# Modular Synthesis of Ru(II)–Ir(III) Complexes Bridged by a Ditopic Carbanionic NHC Ligand: Influence of Heterodinuclear Architecture on Tandem Catalysis

**DOI:** 10.1021/acs.inorgchem.6c00615

**Published:** 2026-06-14

**Authors:** Genesis Ramos-Guzmán, Juan Olguín

**Affiliations:** Departamento de Química, Centro de Investigación y de Estudios Avanzados del Instituto Politécnico Nacional (Cinvestav), Avenida IPN 2508, Col. San Pedro Zacatenco, Ciudad de México 07360, México

## Abstract

The development of polymetallic complexes incorporating N-heterocyclic carbene (NHC) ligands is a promising strategy for achieving enhanced performance that may arise from the presence of multiple metal centers in chemical transformations and/or physicochemical properties. However, quantifying cooperativity in these systems remains a significant challenge. We report a modular synthetic strategy to obtain Ru­(II)–Ir­(III) heterodinuclear complexes bridged by a bis-bidentate ditopic carbanionic NHC (dc-NHC) ligand (L^2^). Our synthetic methodology allowed selective metal coordination at the C2 and C4 imidazolium positions. A series of homo- and heterodinuclear complexes were synthesized, fully characterized, and evaluated as catalysts in tandem α-alkylation/transfer hydrogenation of ketones with alcohols under mild, neat, and base-limited conditions. Notably, the heterodinuclear complexes [Ru-L^2^-Ir]­PF_6_ and [Ir-L^2^-Ru]­PF_6_ exhibited the highest catalytic activity for tandem products, compared to the corresponding monometallic Ru­(II) and Ir­(III) analogues under the same metal percentage loadings. Additionally, a possible contribution of the heterodinuclear arrangement was examined by kinetic experiments (*E*_a_), which indicate lowered activation barriers for [Ru-L^2^-Ir]­PF_6_.

## Graphical abstract

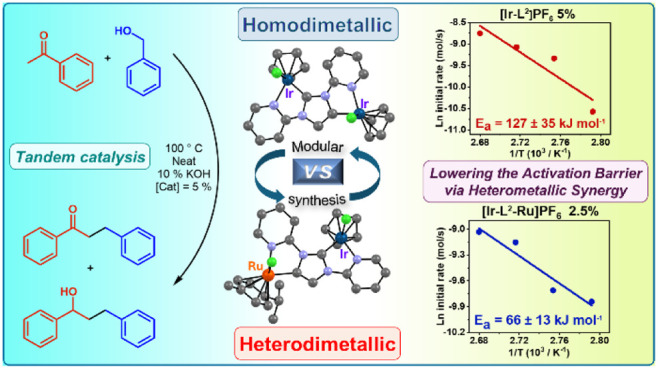

## Introduction

N-heterocyclic carbene (NHC) ligands have become cornerstone scaffolds in organometallic chemistry. Compared to traditional phosphines or nitrogen-based ligands, NHCs form significantly stronger metal–ligand bonds, providing superior thermal and air stability.^1^ Furthermore, their electronic and steric profiles are easily tuned through substituent modification, making them potentially superior candidates for a wide range of catalytic applications.^2–8^

The typical coordination mode for imidazole-based NHCs occurs via the C2-carbon (normal or nNHC, Figure 1a). However, coordination can also occur at the C4/C5 positions to produce abnormal NHC^9^ (aNHC, Figure 1b) also known as mesoionic carbenes (MICs).^10,11^ These two modes possess distinct electronic properties: while nNHCs can be represented by at least one neutral canonical resonance structure, aNHCs are inherently zwitterionic. Consequently, these exhibit markedly enhanced σ-donor strength, a property effectively utilized to boost activity in various catalytic applications.^12–16^

**Figure 1:**
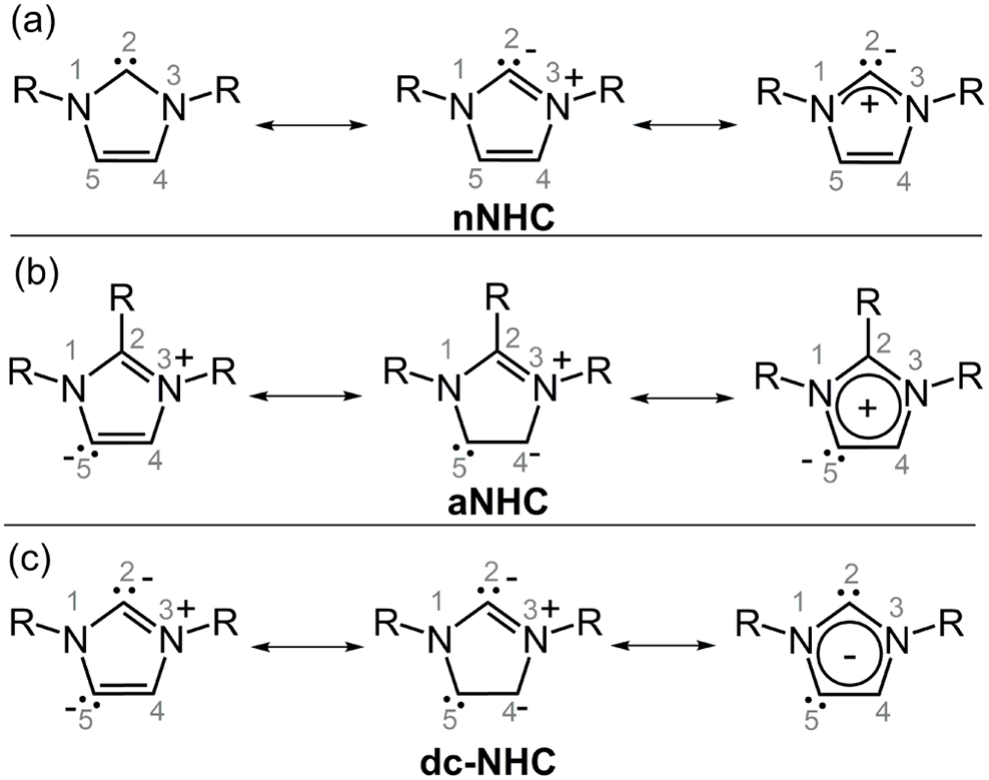
Resonance structures of imidazole-based N-heterocyclic carbenes: (a) nNHC, (b) aNHC, and (c) dc-NHC.

Considering that most of the reported NHC based complexes are monometallic, a compelling frontier lies in ditopic carbanionic NHCs (dc-NHCs), which bridge two metal centers via the C2 and C4/C5 positions simultaneously (Figure 1c). This framework introduces a controlled electronic dissymmetry, allowing each metal center to experience a distinct electronic environment. Such systems offer a new paradigm in ligand design, where bridging-mediated metal communication opens new perspectives in tandem catalysis and multifunctional materials.^7,17^

Polymetallic systems are highly valued for their potential to exhibit cooperative effects due to metal–metal interaction, that create special features impossible or difficult to achieve for monometallic complexes.^7^ It can be confirmed when the total energy, or a physical property, is determined by more than pairwise contributions of its components.^18,19^ The result of cooperativity could have a positive or beneficial impact in the reactions or properties, nevertheless this is not always the case.^20^ On the other hand, heterometallic complexes could show cooperativity and execute tandem catalysis, that is each metal center could be responsible for different transformations.^21–28^

Despite polynuclear systems could combine the properties of NHC ligands and metal–metal cooperativity,^29–31^ only a limited number of polymetallic metal complexes, particularly dinuclear ones containing a single NHC ligand bridging the metal centers have been reported.^32–46^ For more details on these types of systems and their use in tandem catalysis see Figure S1 and Table S1 in the Supporting Information.

In this work, with the aim of evaluating whether the proximity of two metal centers mediated by a single dc-NHC ligand influences catalytic behavior, we designed a modular synthetic route to obtain Ru­(II)–Ir­(III) heterodinuclear complexes bridged by a single bis-bidentate dc-NHC ligand **L** ^**2**^, Figure 2c. Our strategy enables precise control over metal placement at either the C2 or C4 positions, granting straightforward access to both dimetallic complexes and their mononuclear counterparts, Figure 2a–c.

**Figure 2:**
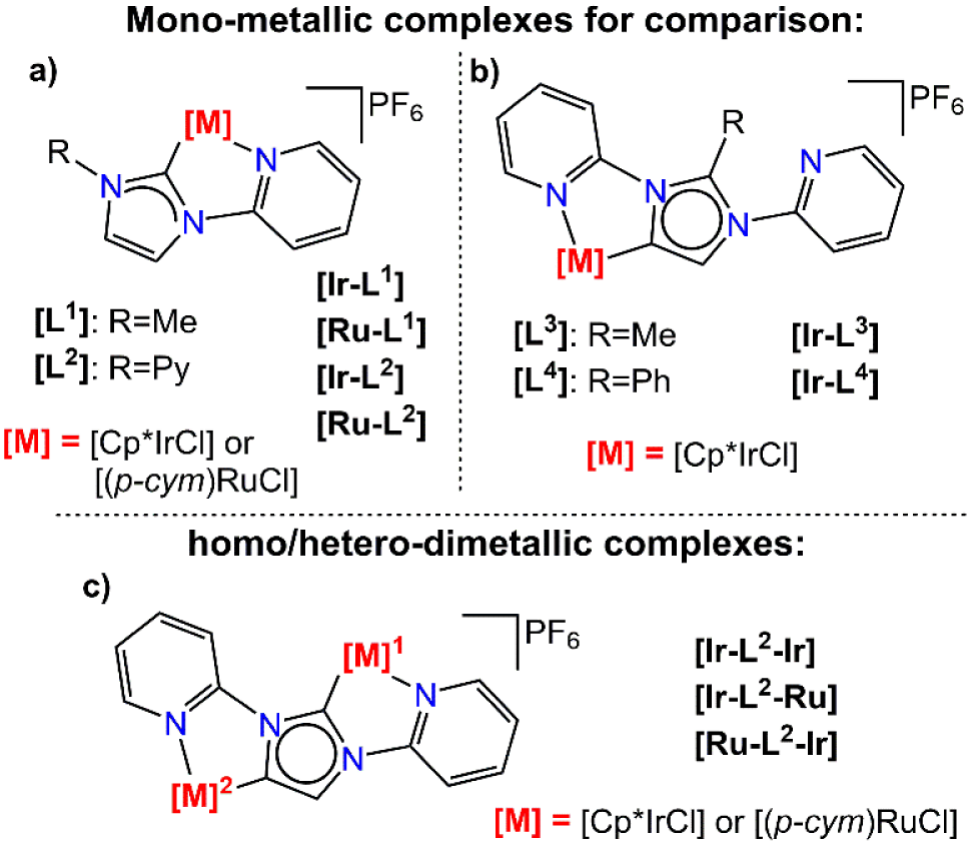
Mono- and homo/heterodimetallic complexes obtained in this work, (a) monometallic complexes using **[L^1^**] and **[L^2^**] ligands, (b) monometallic complexes using **[L^3^**] and **[L^4^**] ligands and (c) homo/heterodimetallic complexes using **[L^2^**] ligand.

The complexes were characterized by standard techniques (^1^H and ^13^C NMR, MS, EA, UV–vis), cyclic voltammetry and for some complexes single crystal X-ray diffractometry. The mono and dinuclear systems were tested as catalysts in the α-alkylation and transfer hydrogenation tandem reaction, using only 10% mol of base (KOH), 5% mol catalysts (metal based), and in neat conditions. More importantly, the influence of the dinuclear architecture was examined by kinetic experiments, suggesting a beneficial effect for **[Ru-L** ^**2**^ **-Ir]­PF**_**6**_ and a negative for **[Ir-L** ^**2**^ **-Ir]­PF**_**6**_.

## Results and Discussion

### Synthesis and Characterization

Ligand precursors **[HL** ^**1**^ **]­PF**_**6**_ ^47,48^ and **[HL** ^**2**^ **]­PF**_**6**_ ^49–51^ were prepared according to reported procedures, while **[HL** ^**3**^ **]­PF**_**6**_ and **[HL** ^**4**^ **]­PF**_**6**_ were synthesized in two steps (see Supporting Information). Mononuclear complexes **[Ir–L** ^**1**^ **]­PF**_**6**_ and **[Ru–L** ^**1**^ **]­PF**_**6**_ were synthesized as previously described,^52–55^ and complexes **[Ir-L** ^**2**^ **]­PF**_**6**_ and **[Ru-L** ^**2**^ **]­PF**_**6**_ were obtained via a onepot Ag_2_O mediated protocol using tetramethylammonium chloride as a phase-transfer catalyst^56^ and [Cp*IrCl_2_]_2_ or [(*p-cymene*)­RuCl_2_]_2_, in 77 and 93% yield respectively, Scheme 1. Attempts to isolate the corresponding Ag–NHC intermediate were unsuccessful since the silver complex is unstable after filtration.

**Scheme 1:**
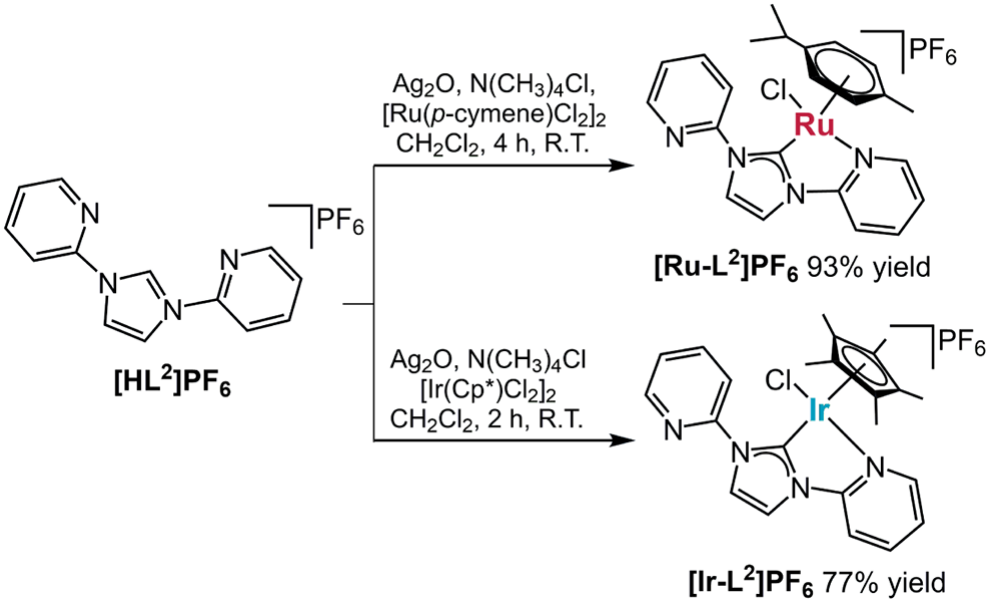
Synthesis of Mononuclear nNHC Complexes **[Ir-L** ^**2**^ **]­PF**_**6**_ and **[Ru-L** ^**2**^ **]­PF**_**6**_

Mononuclear iridium aNHC complexes, **[Ir-L** ^**3**^ **]­PF**_**6**_ and **[Ir-L** ^**4**^ **]­PF**_**6**_, were synthesized by direct reaction of the corresponding ligands with [Cp*IrCl_2_]_2_ under reflux of dichlorobenzene in moderate yields (62 and 58% respectively, Scheme 2). Attempts to isolate the analogous ruthenium complex of **L** ^**4**^ resulted in low yields and rapid decomposition during purification, precluding further investigation.

**Scheme 2:**
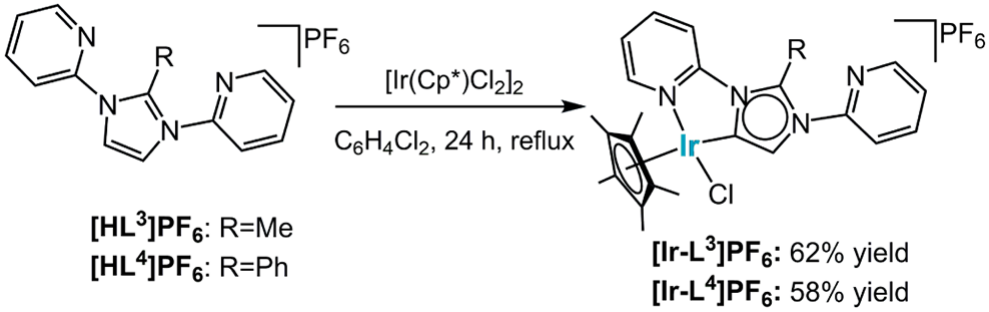
Synthesis of Mononuclear aNHC Complexes **[Ir-L** ^**3**^ **]­PF**_**6**_ and **[Ir-L** ^**4**^ **]­PF**_**6**_

Complex formation was confirmed by ^1^H and ^13^C­{^1^H} NMR spectroscopy and ESI-MS. In all mononuclear complexes, the ^1^H NMR spectra show the disappearance of the C2–H or C4(5)-H imidazolium proton of the ligand precursor and an increase in the number of signals for the pyridyl moieties, consistent with chelation-induced symmetry lowering. Characteristic alkyl and aromatic signals associated with Cp* (Me at δ ≈ 1.4–1.9 ppm) or *p*-cymene (aromatic CH at δ ≈ 5.1–6.1 ppm) further corroborate metal coordination. In addition, for the latter ligand, a diastereotopic pattern is observed due to coordination.

In the^13^C­{^1^H} NMR spectra of the mononuclear nNHC complexes, the M–C_C2‑NHC_ resonances appear in the expected downfield region at δ = 167.07 ppm for Ir and δ = 186.17 ppm for Ru (Table 1, Figures S7 and S9). For the abnormal NHC iridium complexes **[Ir-L** ^**3**^ **]­PF**_**6**_ and **[Ir-L** ^**4**^ **]­PF**_**6**_, coordination at the C4 position gives rise to Ir–C_C4–NHC_ resonances in a distinctly upfield region (δ ≈ 143–148 ppm, Table 1, Figures S12 and S14), consistent with this coordination mode.^57–59^

**Table 1:** Relevant ^13^C­{^1^H} NMR Chemical Shift (ppm) of Mono- and Di-Nuclear Complexes

	Compound						
	^13^C NMR chemical shift (ppm)						
Bond	[Ir-L^2^]^a^	[Ru-L^2^]^b^	[Ir-L^3^]^c^	[Ir-L^4^]^c^	[Ir-L^2^-Ir]^a^	[Ir-L^2^-Ru]^a^	[Ru-L^2^-Ir]^b^
M-C_C2‑NHC_	167.07	186.17			165.52^d^	164.42^d^	183.86^d^
					165.04^e^	165.01^e^	183.18^e^
M-C_C4‑NHC_			143.83	147.70	145.92^d^	160.20^d^	146.85^d^ ^e^
					148.68^e^	162.47^e^	
ipso-Cp*	93.91		90.67	91.01	93.70^d^ ^f^	93.72^d^ ^f^	90.67^d^ ^g^
					93.87^e^ ^f^	93.77^e^ ^f^	90.67^e^ ^g^
					91.00^d^ ^g^		
					90.89^e^ ^g^		
Me-Cp*	9.16		9.17	9.26	9.27^d^ ^f^	9.36^d^ ^f^	9.17^d^ ^g^
					9.30^e^ ^f^	9.27^e^ ^f^	8.81^e^ ^g^
					9.29^d^ ^g^		
					9.32^e^ ^g^		
CH-*p*-cymene		84.33–92.90				83.45–91.83^d^ ^g^	85.92–93.00^d^ ^f^
						83.44–90.00^e^ ^g^	84.64–92.70^e^ ^f^

a Acetonitrile-*d*_*3*_.

b Acetone-*d*_*6*_.

c Chloroform-*d.*

d Isomer A.

e Isomer B.

f M­(nNHC).

g M­(aNHC).

Dinuclear complexes were accessed in a modular way from the mononuclear precursors using Ag_2_O and NaOAc, except for **[Ir-L** ^**2**^ **-Ir]­PF**_**6**_ that only the latter was necessary, Scheme 3. Homo and heterodinuclear complexes **[Ir-L** ^**2**^ **-Ir]­PF**_**6**_, **[Ir-L** ^**2**^ **-Ru]­PF**_**6**_ and **[Ru-L** ^**2**^ **-Ir]­PF**_**6**_ were obtained in moderate to good yields, 53–89%. The corresponding diruthenium analogue could not be isolated due to the thermal instability of **[Ru-L** ^**2**^ **]­PF**_**6**_. All dinuclear complexes were obtained as mixtures of [R-R], [S–S], [R-S] and [S-R] diastereomers due to the chirality at both metal centers.^59^ Only in the case of **[Ir-L** ^**2**^ **-Ru]­PF**_**6**_ was it possible to separate the diastereomer mixture by slow vapor diffusion of diethyl ether into a saturated solution of the mixture in acetonitrile. One of the enantiomer pairs, isomer A, precipitated from the mixture, whereas the other pair, isomer B, remained in the mother liquor, however, once the purified complex is dissolved, conversion to the diastereomer mixture occurs within hours.

**Scheme 3:**
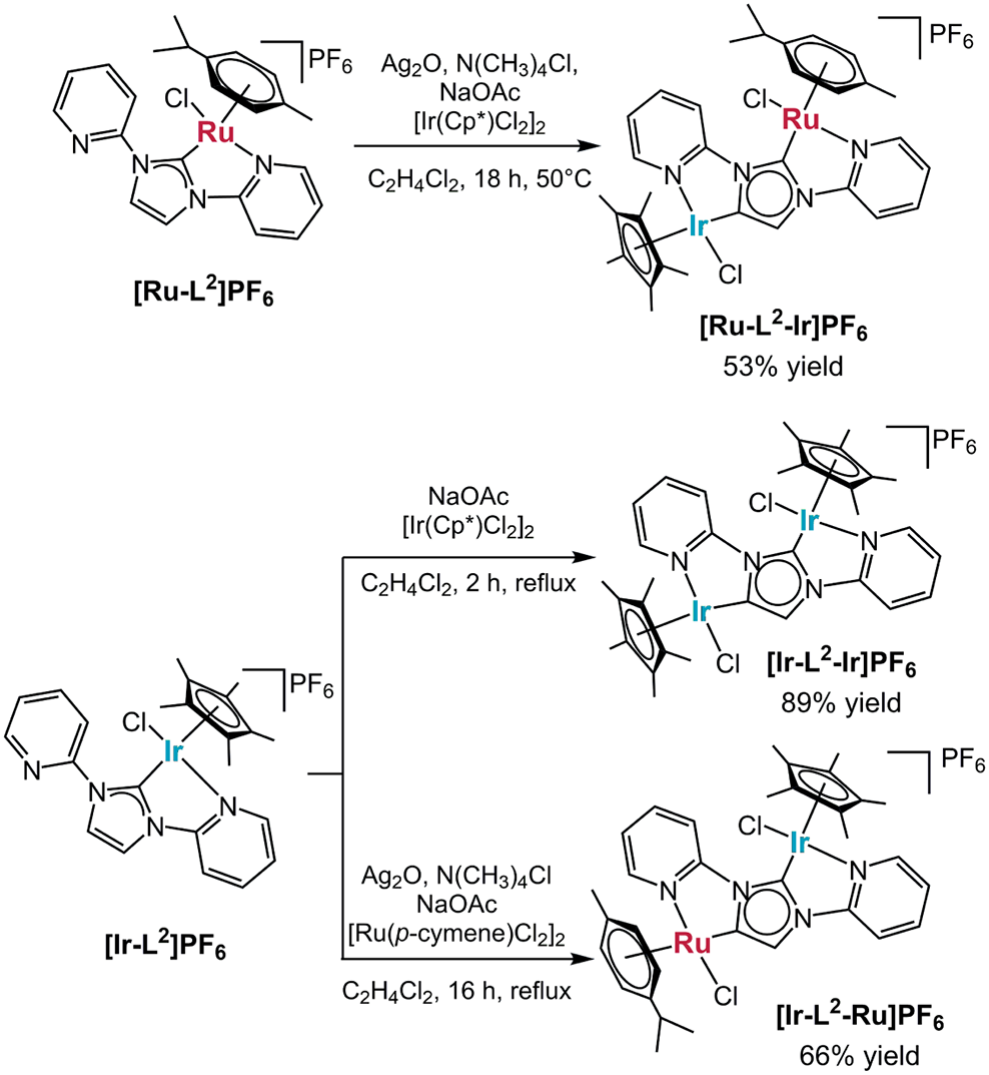
Synthesis of Homo- **[Ir-L** ^**2**^ **-Ir]­PF**_**6**_ and Hetero-Dinuclear **[Ir-L** ^**2**^ **-Ru]­PF**_**6**_, **[Ru-L** ^**2**^ **-Ir]­PF**_**6**_ Complexes

Spectroscopically, dinuclear complex formation is evidenced by disappearance of the resonance for the proton at the 4-position of the imidazole ring in the corresponding mononuclear complex precursor (δ­(^1^H) ≈ 7.9–8.6 ppm), increased number of Cp* and diastereotopic *p*-cymene resonances in the ^1^H NMR spectra, and the emergence of two sets of M–C_C4‑NHC_ signals in the ^13^C­{^1^H} NMR spectra, due to diastereomer mixture, except for **[Ru-L** ^**2**^ **-Ir]­PF**_**6**_ in which only one Ir–C_C4‑NHC_ resonance is observed, Table 1.

Importantly, in all dinuclear systems the M–C_C2–NHC_ resonances exhibit systematic upfield shifts (1.5–3.0 ppm) relative to the corresponding mononuclear complexes due to a second metalation at the ligand unit.^60^ These shifts are independent of homo or heterodinuclear composition and become more pronounced when a second, electronically distinct metal center is introduced. This behavior is consistent with perturbation of the electronic environment of one metal center upon coordination of a second metal, as observed in other heterodimetallic systems.^61^

In an attempt to obtain the analogous abnormal mononuclear complexes containing **L** ^**2**^ ligand, the dinuclear complexes, **[Ir-L** ^**2**^ **-Ir]­PF**_**6**_ and **[Ir-L** ^**2**^ **-Ru]­PF**_**6,**_ were dissolved in CH_3_CN and treated with trifluoroacetic acid to protonate and demetalate the C2 position at room temperature, however in both cases, the ^1^H NMR spectra did not suffer any change, demonstrating high stability of the dinuclear systems, see Supporting Information. Upon more addition of acid equivalents (up to 10) and heating to 80 °C for several days, the ^1^H NMR spectrum for **[Ir-L** ^**2**^ **-Ir]­PF**_**6**_ did not show any change (Figure S31 and S32). However, under these conditions the spectrum for complex **[Ru-L** ^**2**^ **-Ir]­PF**_**6**_ showed transformation to the homo dimetallic complex, **[Ir-L** ^**2**^ **-Ir]­PF**_**6**_, suggesting a high thermodynamic stability of the latter homometallic complex (Figures S33 and S34).

To further characterize the complexes ESI–MS experiments and UV–vis spectra were collected. The mass spectra displayed the expected ion peaks corresponding to the mono and dinuclear cationic species.

The absorption spectra of ligands, mononuclear and dinuclear complexes were recorded in acetonitrile (Figures S77–S80) and the key absorption bands are summarized in Table S14. The mononuclear complexes exhibit intense absorption bands at 217 to 279 nm (ε = 14901–30772 M^–1^ cm^–1^, Figure S78) and one additional moderately strong absorption band at 273–350 nm (ε = 1583–5943 M^–1^ cm^–1^). Finally, the dinuclear complexes exhibit the same absorption bands as the ligands and the mononuclear complexes in addition to one weak absorption band observed at 435–437 nm (ε = 1583–5943 M^–1^ cm^–1^, Figure S79).

Due to the extensive investigation in the literature on related complexes,^53,62–65^ it is possible to assign the intense absorption bands in the UV range (<300 nm) to ligand-centered electronic π → π* transitions of the aromatic groups of the ligands. The absorption bands observed for complexes in the visible region in the 340–437 nm range are assigned to spin-allowed metal-to-ligand charge-transfer (MLCT) transitions {Ru/Ir (dπ) → (π*) ligand}.

### X-ray Crystallography Analysis

Crystals suitable for X-ray crystallography were obtained by slow vapor diffusion of diethyl ether or pentane into a solution of the complexes in a suitable solvent (see Experimental Section and Figures S35–S39). The X-ray crystallographic data were acquired at room temperature for all complexes; relevant bond parameters are found in Table 2, and crystallographic data are given in Table S2. In some cases, the complexes crystallized as a mixture of enantiomers with the chiral center located at the metal.

**Table 2:** Selected Bond Lengths (Å) and Angles (°) for **[Ir-L^2^]­PF_6_**, **[Ru-L^2^]­B­(Ph)_4_**, **[Ir-L^3^]­PF_6_**, **[Ir-L^2^-Ir]­PF_6_,** and **[Ir-L^2^-Ru]­PF_6_**

NHC position		[Ir-L^2^]PF_6_	[Ru-L^2^]B(Ph)_4_	[Ir-L^3^]PF_6_	[Ir-L^2^-Ir]PF_6_	[Ir-L^2^-Ru]PF_6_
C2-NHC	Bond Length (Å)					
	M-Cl	2.3923(8)	2.3834(16)		2.390(4)	2.391(4)
	M-C_NHC_	2.010(3)	2.012(5)		2.004(7)	2.017(14)
	M-N_Py_	2.092(3)	2.103(4)		2.112(9)	2.087(12)
	Bond Angle (°)					
	C_NHC_-M-N_Py_	76.46(12)	76.51(19)		75.1(4)	75.7(6)
	Cl-M-N_Py_	84.17(8)	87.41(14)		88.4(3)	87.0(3)
	Cl-M-C_NHC_	89.67(9)	89.92(15)		91.9(3)	90.0(4)
C4-NHC	Bond Length (Å)					
	M-Cl			2.4225(18)	2.398(4)	2.396(4)
	M-C_NHC_			2.018(7)	2.006(8)	2.028(16)
	M-N_Py_			2.110(6)	2.082(7)	2.086(12)
	Bond Angle (°)					
	C_NHC_-M-N_Py_			76.5(3)	77.1(4)	78.0(5)
	Cl-M-N_Py_			82.69(18)	83.9(3)	83.7(3)
	Cl-M-C_NHC_			91.0 (2)	88.5(3)	87.6(5)

In our hands, crystals suitable for X-ray diffraction of the complex **[Ru-L** ^**2**^ **]­PF**_**6**_ could not be obtained, therefore PF_6_^–^ was replaced by B­(Ph)_4_^–^, obtaining suitable crystals for **[Ru-L** ^**2**^ **]­B­(Ph)**_**4**_. A perspective view for complexes **[Ir-L** ^**2**^ **]­PF**_**6**_, **[Ru-L** ^**2**^ **]­B­(Ph)**_**4**_ and **[Ir-L** ^**3**^ **]­PF**_**6**_ is shown in Figure 3. The structure obtained for the latter confirmed that the iridium center is indeed coordinated to the 4-position of the imidazole ring. The Ir–C_C2‑NHC_ bond lengths for **[Ir-L** ^**2**^ **]­PF**_**6**_ and Ir–C_C4‑NHC_ for **[Ir-L** ^**3**^ **]­PF**_**6**_ are 2.010(3) and 2.018(7) Å respectively, which are in agreement with similar reported complexes, for both C2 and C4 modes.^52,66–69^

**Figure 3:**
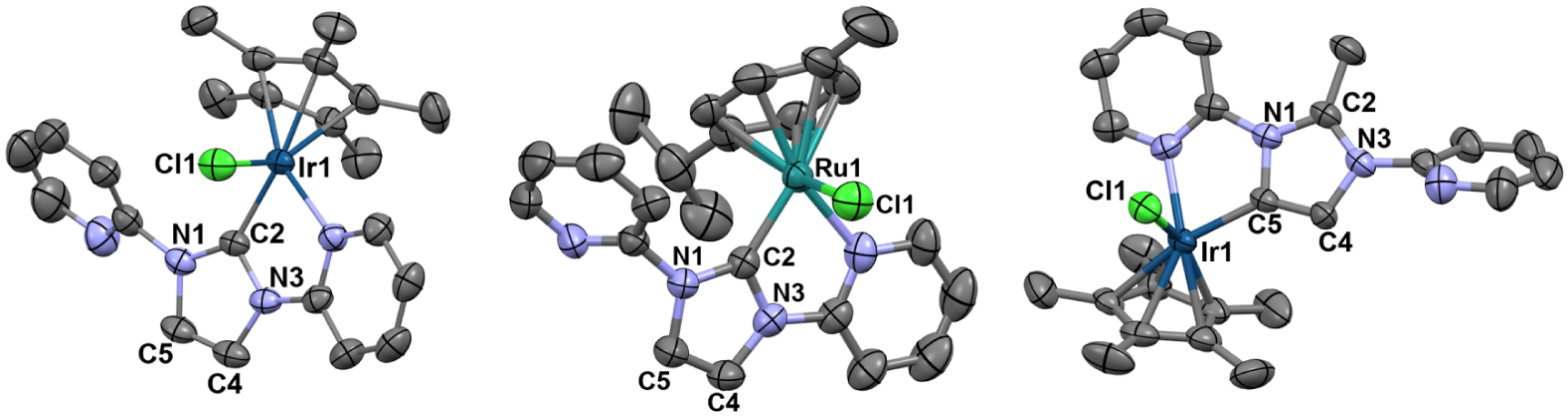
Perspective view of **[Ir-L^2^]­PF_6_** (left), **[Ru-L^2^]­B­(Ph)_4_** (middle) and **[Ir-L^3^]­PF_6_** (right), drawing with 30% probability thermal ellipsoids. The counterion and all hydrogen atoms have been omitted for clarity.

The structure of the dinuclear complexes **[Ir-L** ^**2**^ **-Ir]­PF**_**6**_ and **[Ir-L** ^**2**^ **-Ru]­PF**_**6**_ is shown in Figure 4. In the case of the former, it is possible to observe two [Cp*IrCl] fragments in a *syn* conformation, similar to other dinuclear iridium complexes.^32^ The Ir1 center is linked to the C2-position, while Ir2 is bond to the C4-position of the same imidazole ring, with a Ir···Ir distance of 6.046 Å. Whereas, for **[Ir-L** ^**2**^ **-Ru]­PF**_**6**_ a [Cp*IrCl] fragment is coordinated to the C2-position, while the [*p-cymene*)­RuCl] moiety is coordinated to the C4-position. The Ir···Ru distance is 6.051 Å, statistically identical to Ir···Ir distance. Unfortunately, all efforts so far to obtain single crystals of **[Ru-L** ^**2**^ **-Ir]­PF**_**6**_ have been unsuccessful.

**Figure 4:**
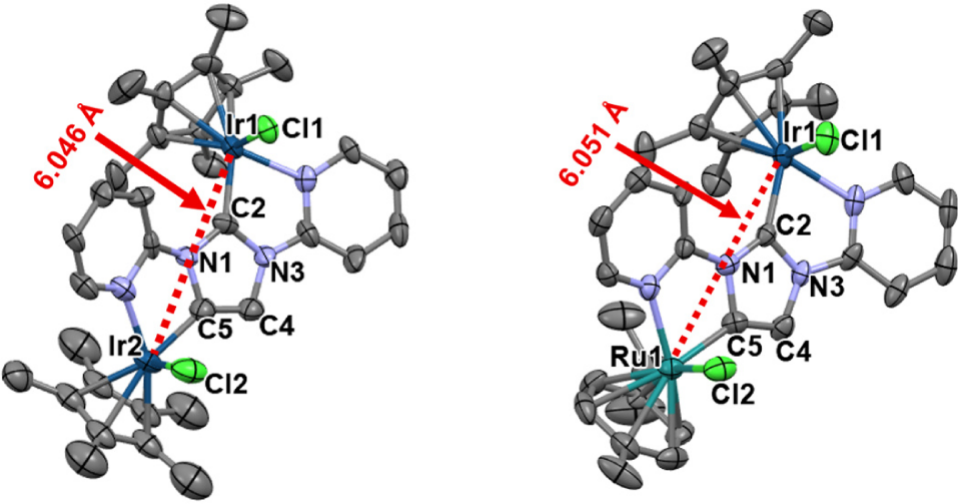
Perspective view of **[Ir-L^2^-Ir]­PF_6_** (left) and **[Ir-L^2^-Ru]­PF_6_** (right), drawing with 30% probability thermal ellipsoids. The **PF_6_** counterion and all hydrogen atoms have been omitted for clarity.

Comparing the M-C_NHC_ of the mononuclear and dinuclear complexes, Table 2, it is possible to observe that C2 and C4 coordination modes produce statistically indistinguishable M-C_NHC_ bond lengths, with only minor variations across mono and dinuclear systems. Nonetheless, it is possible to observe a significative difference in the M-Cl bond length distance in the mononuclear complexes. In all cases the longest M-Cl bond length is observed in the C4 position coordination mode indicating a stronger bonding and a higher σ-donation of the C4-NHC side, weakening the M-Cl bond.^70^ Interestingly for the homo and hetero dimetallic complexes, **[Ir-L** ^**2**^ **-Ir]­PF**_**6**_ and **[Ir-L** ^**2**^ **-Ru]­PF**_**6**_, both C2 and C4 positions show M-Cl bond length distances similar among them. The latter is consistent with redistribution of electronic effects across the dc-NHC framework.

### Catalytic Tandem α-Alkylation/Transfer Hydrogenation Reactions

The catalytic tandem α-alkylation and transfer hydrogenation reaction (Scheme 4) was selected as the benchmark experiment to investigate how the presence of two different metal centers, coordinated to the dc-NHC bridging ligand, influences catalytic performance.

**Scheme 4:**
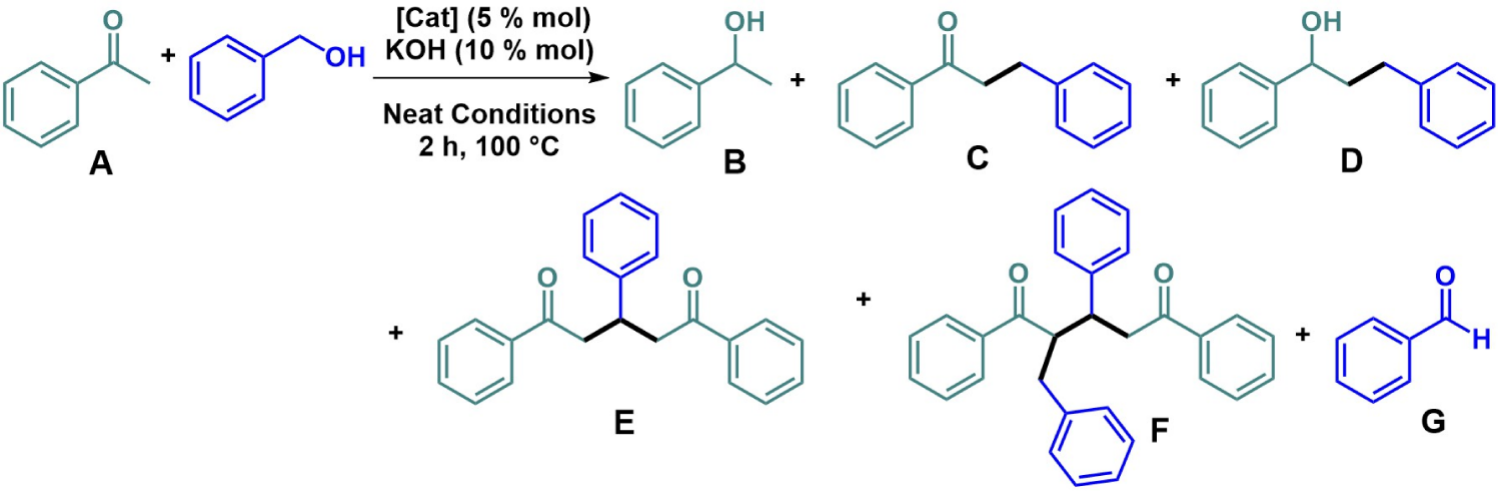
Tandem α-Alkylation and Transfer Hydrogenation Reaction

In our experiments, acetophenone (**A**, Scheme 4) and benzyl alcohol were used as substrates in a 1:3 molar ratio, leading to possible formation of six products (**B–G**, Scheme 4). Compound **C** is a tandem product formed through two consecutive steps: aldol condensation following hydrogenation. Product **D** is another tandem product obtained from the hydrogenation of carbonyl group in **C** (for details of the mechanism, see the Supporting Information). In addition, the formation of the aldol self-condensation of acetophenone (Figures S40–S41) was not detected in the experiments performed in this work, for more details see Supporting Information.

Several parameters were screened to find the optimal reaction conditions (Table S4 and analysis in Supporting Information). The best conditions for obtaining compounds **C** and **D** were 10% mol KOH as base, 5% mol catalysts (metal based), in neat conditions at 100 °C for 2 h.

The results showed that the monometallic complexes coordinated to the C2 position, **[Ir-L** ^**1**^ **]­PF**_**6**_, **[Ru-L** ^**1**^ **]­PF**_**6**_, **[Ir-L** ^**2**^ **]­PF**_**6**_ and **[Ru-L** ^**2**^ **]­PF**_**6**_ (Table 3, entries 1–2 and 4–5), obtained good conversion for **A** ∼98% and good yield for product **C** ∼70%, however low yield for the double hydrogenated product **D** ∼16%. While, interestingly, the iridium complexes coordinated to the C4-position, **[Ir-L** ^**3**^ **]­PF**_**6**_ and **[Ir-L** ^**4**^ **]­PF**_**6**_ (Table 3, entries 7–8) did not show activity for the tandem or transfer hydrogenation processes, but only very low activity for benzyl alcohol dehydrogenation, producing **G** in 6–8%. The latter suggests that neither electron-withdrawing nor donating substituents (phenyl and methyl, respectively) impact the catalytic performance of the metal center.

**Table 3:** Average^1^H NMR Yields of at Least Three Runs for the Tandem α-Alkylation and Transfer Hydrogenation Reaction^a^

			^1^H NMR Yield %							
Entry	Precatalyst	^1^H NMR Conv A %	B	C	D	E	F	G^b^	TOF^c^ (h^–1^)	TON^c^
1	**[Ir-L** ^**1**^ **]PF**_**6**_	99	7	69	18	Traces	Traces	8	-	-
2	**[Ru-L** ^**1**^ **]PF**_**6**_	94	Traces	66	17	Traces	Traces	10	-	-
3	**[Ir-L** ^**1**^ **]PF**_**6**_ + **[Ru-L** ^**1**^ **]PF**_**6**_	26	6	18	-	Traces	-	36	-	-
4	**[Ir-L** ^**2**^ **]PF**_**6**_	96	8	69(55)^d^	16(11)^d^	Traces	Traces	Traces	101	51
5	**[Ru-L** ^**2**^ **]PF**_**6**_	99	6	70(58)^d^	14(7)^d^	Traces	Traces	Traces	144	72
6	**[Ir-L** ^**2**^ **]PF**_**6**_ + **[Ru-L** ^**2**^ **]PF**_**6**_	50	20	21	9	-	-	7	-	-
7	**[Ir-L** ^**3**^ **]PF**_**6**_	15	17	-	-	-	-	6	-	-
8	**[Ir-L** ^**4**^ **]PF**_**6**_	13	9	Traces	-	-	-	8	-	-
9	**[Ir-L** ^**2**^ **-Ir]PF**_**6**_	17	10	6	-	-	-	Traces	-	-
10	**[Ir-L** ^**2**^ **-Ru]PF**_**6**_	95	Traces	55(45)^d^	34(22)^d^	Traces	Traces	Traces	125	63
11	**[Ru-L** ^**2**^ **-Ir]PF**_**6**_	92	Traces	58(49)^d^	28(19)^d^	Traces	Traces	Traces	165	82
12^e^	**[Ir-L** ^**2**^ **-Ru]PF**_**6**_	94	Traces	65	23	Traces	-	Traces	-	-
13	[Ru(*p-cym*)Cl_2_]_2_	20	14	5	-	-	-	6	-	-
14	[IrCp*Cl_2_]_2_	27	23	2	-	Traces	-	7	-	-
15	Blank 10% base	11	Traces	-	-	Traces	Traces	-	-	-
16	Blank 50% base	99	17	25	57	-	-	Traces	-	-

a Reaction conditions: Acetophenone (1 mmol), benzyl alcohol (3 mmol), base (10% mol), catalyst (5% mol, metal based), 100 °C for 2 h and neat conditions. Conversion and yield measured by ^1^H NMR spectra analysis. The results are the average of at least three independent runs.

b Benzaldehyde yield was measured based on benzyl alcohol.

c TOF determined at 30 min, when the catalyst is in full action. For more details of TOF and TON calculation, see Supporting Information.

d Isolated yield in parentheses.

e One drop of mercury added.

The homometallic complex **[Ir-L** ^**2**^ **-Ir]­PF**_**6**_ (Table 3, entry 9) showed analogous behavior as for the C4-mononuclear **[Ir-L** ^**3**^ **]­PF**_**6**_ and **[Ir-L** ^**4**^ **]­PF**_**6**_ complexes (Table 3, entries 7 and 8). Suggesting a negative effect between both Ir centers and the high stability of the homodinuclear complex, which was expected based on the formation of this species when adding acid to the heterometallic complexes (see above).

The heterodinuclear complexes **[Ir-L** ^**2**^ **-Ru]­PF**_**6**_ and **[Ru-L** ^**2**^ **-Ir]­PF**_**6**_ (Table 3, entries 10 and 11) showed the highest activity for the tandem processes, obtaining compounds **C** and **D** in 55 and 34% respectively for **[Ir-L** ^**2**^ **-Ru]­PF**_**6**_, and 58 and 28% respectively for **[Ru-L** ^**2**^ **-Ir]­PF**_**6**_, this is the highest yield observed for compound **D** in all the series of compounds tested. The latter behavior and the fact that mononuclear Ir–C4–NHC complexes do not show activity in the tandem process, is consistent with a beneficial interaction between the metal centers under these conditions mediated by the ditopic dc-NHC ligand (**L** ^**2**^). In addition, mercury drop tests^71,72^ were performed, confirming the homogeneous nature of the catalytic system (Table 3, entry 12).

Experiments were conducted using an equimolar mixture of acetophenone and benzyl alcohol to promote a hydrogen borrowing process. The complexes **[Ir-L** ^**2**^ **]­PF**_**6**_, **[Ru-L** ^**2**^ **]­PF**_**6**_ and **[Ir-L** ^**2**^ **-Ru]­PF**_**6**_ were evaluated as catalysts (see Supporting Information for experimental details). While **[Ir-L** ^**2**^ **]­PF**_**6**_ afforded moderate conversion of **A** to products **C** and **E** (60% and 13% respectively), **[Ru-L** ^**2**^ **]­PF**_**6**_ exhibited no catalytic activity under the same conditions. Interestingly, the heterodinuclear complex **[Ir-L** ^**2**^ **-Ru]­PF**_**6**_ showed high conversion of **A**, yielding product **C** exclusively (83%). These results suggest that the presence of different metal centers bridged by the dc-NHC exhibit a positive influence on the yield and selectivity of the reaction, indicating that the heterodinuclear architecture favorably impacts reaction selectivity. In addition, the results show that the formation of product **D** proceeds via a hydrogen transfer mechanism rather than a hydrogen borrowing process. Consequently, an excess of benzyl alcohol is required to ensure an efficient transformation toward **D**, demonstrating that the selectivity of the catalytic process can be governed by stoichiometric control.

To evaluate the effect of the bridging ligand, and to verify that the higher activity is not a product of the mere presence of both metal centers in the reaction, a mixture of the mononuclear Ru and Ir complexes was used in the catalytic system (Table 3, entries 3 and 6). Remarkably, the catalytic activity for the tandem process plummeted, obtaining *c.a.* 20% for product **C** and negligible yield for product **D**. Further substoichiometric experiments using the mixture of mononuclear Ir and Ru complexes demonstrated that the mixture transforms into the homodinuclear iridium complex **[Ir-L** ^**2**^ **-Ir]­PF**_**6**_, (Figure S44), which is in good agreement with the lack of catalytic activity observed (*cf.* Table 3, entry 9), and confirming the thermodynamic stability of the homodinuclear Ir complex.

Control experiments were performed; one to determine the activity of [Ru­(*p-cym*)­Cl_2_]_2_ and [IrCp*Cl_2_]_2_ precursors as precatalysts, showing no tandem catalytic activity (Table 3, entries 13–14). A second control experiment performed in the absence of metal catalyst using 10% mol of KOH resulting in negligible formation of compounds **E** and **F** (<5% yield; Table 3, entry 15).

Recent literature examples have shown that some catalytic systems have utilized 50% mol of KOH, employing precatalysts containing the same metals as in this work^73–76^ or other metals as nickel^77^ or rhodium.^78^ Under our reaction conditions and using 50% mol of KOH, that is no metal complex was added, both tandem products **C** and **D** are produced (Table 3, entry 16). This outcome serves as a cautionary note to avoid using more than 10% mol of base, as higher concentrations may promote base-mediated pathways rather than metal-centered catalysis.

The catalytic performance of the heterodinuclear complexes **[Ir-L** ^**2**^ **-Ru]­PF**_**6**_ and **[Ru-L** ^**2**^ **-Ir]­PF**_**6**_ were compared with other active metal-based systems for the α-alkylation/transfer hydrogenation reaction. While Ru-, Ir- and even Fe- or Ni-based complexes typically require 50–200% mol of base, elevated temperatures, and prolonged reaction times (Table S3). The present system operates efficiently at 100 °C, under solvent- free and base-limited conditions, with lower catalyst loading, achieving comparable yields of products **C** and **D**.

The fact that the heterometallic systems produce higher yields of product **D** compared to the mononuclear counter parts, and that under stoichiometric conditions the mononuclear nNHC Ru complex does not show catalytic activity for the tandem reaction, suggests that the heterodinuclear architecture provides a beneficial effect relative to the mononuclear systems. This observation motivated evaluation using the cooperativity index “*a”* proposed by Jones and James^79^ (see eqs S3–S9 in Supporting Information) for **[Ru-L** ^**2**^ **-Ir]­PF**_**6**_ and **[Ir-L** ^**2**^ **-Ir]­PF**_**6**_ complexes. In this study, A_O_ and A_P_ were defined as the percentage yield of product **D**, obtained from the reactions catalyzed by the dinuclear and mononuclear systems, respectively. The cooperativity index value was positive for heterodinuclear complex (*a*_**[Ru‑L2‑Ir]PF6**_ = 2), and negative for homodinuclear complex (*a*_**[Ir‑L2‑Ir]PF6**_ = −2). These results are consistent with a beneficial influence arising from the proximity of two different metal centers under the applied reaction conditions.

To study the catalytic reaction scope and the electronic effect of substituents at either the ketone or alcohol units, a variety of substrates were evaluated under optimal conditions using **[Ir-L** ^**2**^ **-Ru]­PF**_**6**_ as precatalyst (see Supporting Information and Table S5). Aryl ketones with *para* electron-donating substituents afforded product **C** in good yields, while the fully hydrogenated product **D** varied depending on the substituent. For example, *p*-Me gave yields comparable to the parent system (21%), whereas *p*-NH_2_ reduced **D** formation to 7% due to side reactivity of intermediate **C** with benzaldehyde **G**, producing a secondary amine in 40% yield (confirmed by NMR and MS, Figures S45–S46). Electron-withdrawing substituents (*p*-I, *p*-Br) gave moderate conversions (69–73%) and lower yields of **D**. An *ortho*-OH substituent led to diminished conversion (54%), likely due to steric hindrance and reduced electrophilicity of the carbonyl group, and/or by consumption of the base by deprotonation of the OH-moiety.

Variation at the alcohol unit showed similar trends: *p*-OMe benzyl alcohol improved conversion (82%) compared to *p*-NO_2_ (53%), consistent with electronic effects on alcohol dehydrogenation. Piperonyl alcohol, a biologically relevant substrate, gave good conversion (81%) with similar yields of **C** (35%) and **D** (25%). Additionally, it is plausible that the alcohol substrate competes for the base, particularly when electron-withdrawing substituents lower the p*K*_a_, which may also account for the reduced product yield observed for the *p*-nitro-substituted alcohol.

Overall acetophenone and benzyl alcohol substrates bearing electron-donating substituents at *para* positions allow a slight increase in the yield of **C** and **D**, due to further stabilization of the enolate, and the facilitated dehydrogenation of the alcohol to produce the corresponding benzaldehyde.

### Mechanistic Investigations

Mechanistic studies confirmed that the system follows the established α-alkylation/transfer hydrogenation pathway (see Supporting Information for details on the established mechanism and Scheme S3 for experimental details on the mechanistic experiments). Heating benzyl alcohol with base and **[Ir-L** ^**2**^ **]­PF**_**6**_ produced benzaldehyde (δ = 10.04 ppm) and a hydride-Ir­(III) species (δ = −13.93 ppm, Scheme S3a and Figure S47) consistent with reported [Ir]–H complexes.^80,81^ Attempts to isolate the [Ir]–H species were unsuccessful, and no [Ru]–H intermediate was detected, likely due to instability. Substitution of benzyl alcohol with benzaldehyde resulted in no product formation, confirming the essential role of benzyl alcohol as hydrogen donor (Scheme S3 and Figure S48).

To confirm the roles of acetophenone and benzyl alcohol in the tandem process, we conducted a series of deuterium labeling experiments under standard reaction conditions, see Scheme S3c-f in Supporting Information for more details, observing deuteration of the products in agreement with previously reported mechanism.^78,80,82,83^

Using C_6_D_5_CD_2_OH led exclusively to product **C** with deuterium incorporation at the β-position, consistent with alcohol-derived alkylation and a kinetic isotope effect suppressing hydrogenation and production of **D**. When C_6_H_5_CHDOH was used, both **C** and **D** were formed, showing moderate isotope effect (∼1.1) and deuterium incorporation at the alcohol position in **D** (26%), confirming the role of metal hydride in carbonyl reduction. Experiments with C_6_H_5_CH_2_OD showed deuterium incorporation at α and β positions of **C** but no **D** formation. Finally, we performed an experiment using unlabeled benzyl alcohol and C_6_H_5_COCD_3_. This experiment yielded the highest observed deuterium incorporation at the α-position, as expected considering the mechanism reported in previous studies, Schemes S1 and S3c-f.

### Activation Energy Determination

To quantify the energetic benefits of the heterobimetallic architecture, activation energies (*E*_a_) were determined from Arrhenius plots based on initial reaction rates for the formation of product **C** (Figure 5 and Supporting Information). A critical comparison between the mononuclear systems reveals that while the iridium aNHC complexes **[Ir-L** ^**3**^ **]­PF**_**6**_ and **[Ir-L** ^**4**^ **]­PF**_**6**_ (Table 3, entries 7 and 8) were inactive for the tandem process, the mononuclear nNHC complex **[Ir-L** ^**2**^ **]­PF**_**6**_ exhibited clear catalytic activity, albeit with a relatively high energy barrier 127 ± 35 kJ mol^–1^ compared to **[Ru-L** ^**2**^ **]­PF**_**6**_ (102 ± 17 kJ mol^–1^). This distinction demonstrates that the iridium center possesses inherent catalytic potential within the ditopic framework, provided it is coordinated at the appropriate position.

**Figure 5:**
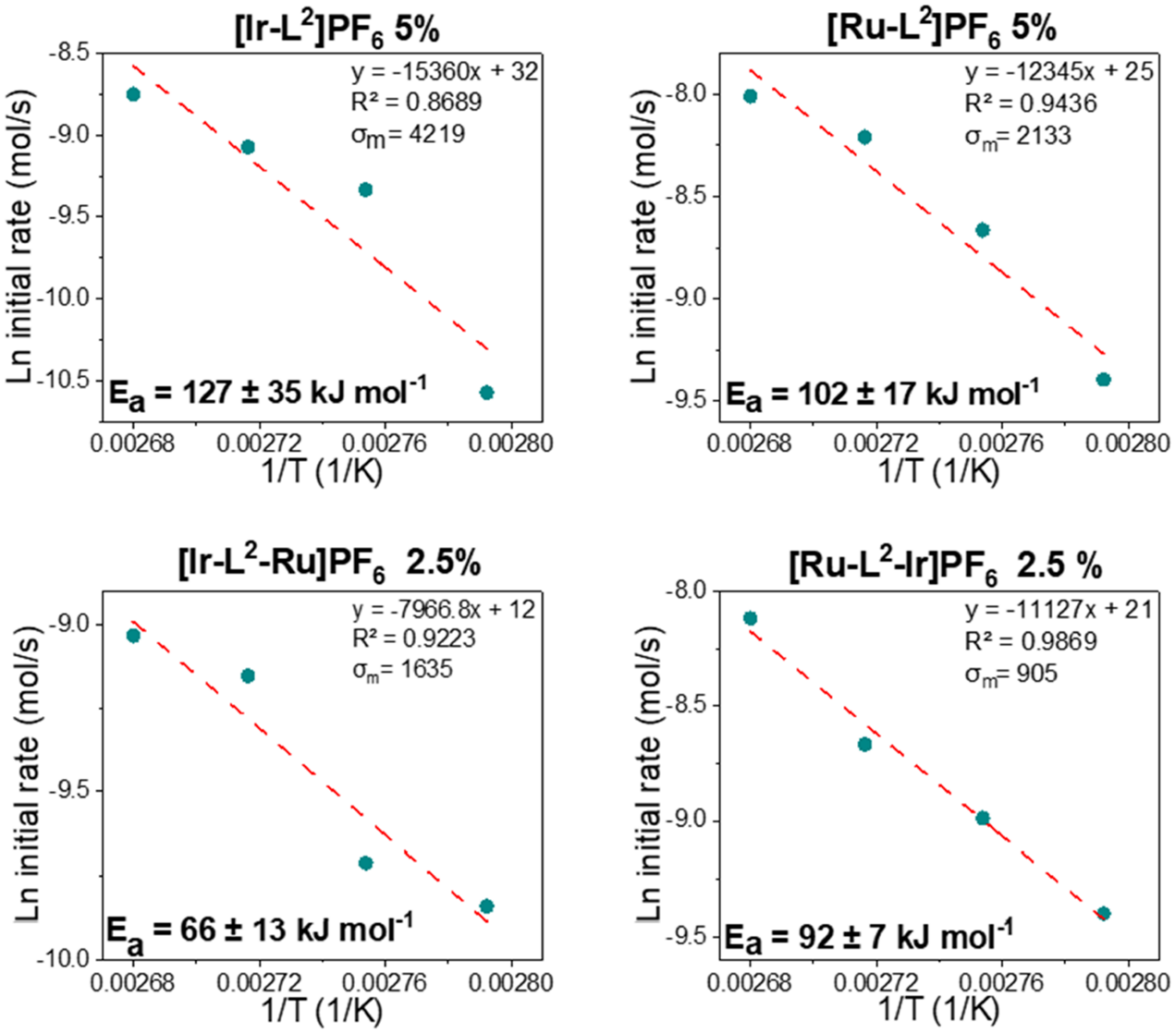
Arrhenius plots for activation energy determination of the catalytic reaction.

The transition from mononuclear to heterodinuclear systems resulted in a significant attenuation of the kinetic barrier. The *E*_a_ values for **[Ir-L** ^**2**^ **-Ru]­PF**_**6**_ (66 ± 13 kJ mol^–1^) and **[Ru-L** ^**2**^ **-Ir]­PF**_**6**_ (92 ± 7 kJ mol^–1^) are notably lower than those of their mononuclear ruthenium and iridium precursors. Specifically, the introduction of ruthenium to the **[Ir-L** ^**2**^ **]­PF**_**6**_ scaffold led to a dramatic decrease in *E*_a_ of 61 kJ mol^–1^, whereas the introduction of iridium to **[Ru-L** ^**2**^ **]­PF**_**6**_ resulted in a relatively small decrease of 10 kJ mol^–1^. These results suggest that both metal centers actively participate in the catalytic cycle, likely through a communication facilitated by the π-conjugated dc-NHC bridge.

The possible synergy was further evaluated by comparing the *E*_a_ of the heterodinuclear systems against the isolated mononuclear subsystems.^19,20^ Given that the aNHC iridium complexes require significantly higher energy for the formation of **C** (Ea **[Ir-L** ^**3 or 4**^ **]­PF**_**6**_ > **[Ru-L** ^**2**^ **]­PF**_**6**_), the calculated cooperative kinetic energy difference δ*E*(kin) was determined to be 10 ± 19 kJ mol^–1^ (eq S14–S15). While the associated uncertainty necessitates a cautious interpretation, the consistent trend of lowered activation energies on the dinuclear systems, which are significantly below the 133–175 kJ mol^–1^ range reported for similar systems,^84,85^ supports the view that the heterodinuclear arrangement may promote a more efficient catalytic pathway.

### Electrochemistry

To investigate the electronic properties and potential electronic coupling between the metal centers, cyclic voltammetry (CV) studies were performed for the free ligand and the corresponding mono- and dinuclear complexes (Table 4 and see the Supporting Information for full experimental details and voltammograms). The mononuclear complexes, **[Ru-L** ^**2**^ **]­PF**_**6**_, **[Ir-L** ^**2**^ **]­PF**_**6**_ and **[Ir-L** ^**3**^ **]­PF**_**6**_, exhibited a chemically irreversible oxidation process in agreement with potentials reported in the literature for Ru^2+/3+^ or Ir^3+/4+^ process.^53,62,86,87^ Notably, a significant shift in the oxidation potential is observed between the iridium mononuclear isomers; the C2-coordinated complex oxidizes at 1.55 V, whereas the C4-coordinated analog shifts to a lower potential of 1.22 V. This 0.33 V cathodic shift provides direct experimental evidence of the significantly stronger σ-donating capability of the imidazole ring when coordinated through the C4 position compared to the C2 site. This electronic trend is maintained in the dinuclear systems **[Ir-L** ^**2**^ **-Ir]­PF**_**6**_ and **[Ru-L** ^**2**^ **-Ir]­PF**_**6**_, which display two distinct, sequential metal-centered oxidation peaks corresponding to the independent oxidation of the metal centers. However, because all observed anodic and cathodic processes across both mono- and dinuclear series are entirely chemically irreversible, a reliable quantitative evaluation of electronic coupling or cooperative intramolecular behavior between the metal centers remains precluded.

**Table 4:** Selected Oxidation and Reduction Peak Potentials for Mono and Dinuclear Complexes^a^

Compound	*E*_ox_ (V vs SCE)		*E*_red_ (V vs SCE)	
**[HL** ^**2**^ **]PF**_**6**_			–1.29	
**[Ru-L** ^**2**^ **]PF**_**6**_		1.46	–1.17	
**[Ir-L** ^**2**^ **]PF**_**6**_		1.55	–1.24	
**[Ir-L** ^**3**^ **]PF**_**6**_	1.22		–1.41	–1.67
**[Ir-L** ^**2**^ **-Ir]PF**_**6**_	1.16	1.53	–1.25	–1.83
**[Ir-L** ^**2**^ **-Ru]PF**_**6**_	1.13	1.33	–1.29	–1.81
**[Ru-L** ^**2**^ **-Ir]PF**_**6**_	1.12	1.47	–1.23	–1.90

a CV were acquired at scan rate of 100 mV/s, at concentration of 2 mM in CH_3_CN with 0.1 M NBu_4_PF_6_ electrolyte, glassy carbon as working electrode, platinum mesh as counter electrode and a Saturated Calomel Electrode (SCE) as reference electrode (calibrated by Fc/Fc+). The *E*_ox_ and *E*_red_ are anodic and cathodic peak potentials.

## Conclusions

We have developed a robust and modular synthetic strategy for the preparation of heterodinuclear complexes bridged by a ditopic carbanionic NHC (dc-NHC) ligand, leveraging regioselective coordination at the asymmetric C2 and C4 positions. The ability to precisely construct such heterobimetallic architectures remains uncommon. Structural and electrochemical analyses confirmed distinct electronic profiles for each site, revealing enhanced σ-donation at the C4 position in mononuclear species and electronic changes upon di­(hetero)­metalation mediated by a π-conjugated bridge.

Catalytic evaluation in the tandem α-alkylation/transfer hydrogenation of ketones demonstrated the clear functional advantages of this heterodinuclear architecture. The heterodimetallic complexes outperformed their mononuclear counterparts by successfully driving the tandem sequence to the fully reduced saturated alcohol with lower activation energies, even under low base loadings that suppress background base-catalyzed pathways. Control experiments confirmed that this enhanced performance arises from the activity of both metal centers.

These findings provide a rational framework for designing cooperative catalysts in tandem transformations using known and readily available imidazolium synthons. The modularity of the dc-NHC scaffold offers future opportunities to tailor electronic communication and reactivity by selecting complementary metal ions, opening avenues in areas such as small molecule activation, photocatalysis, and sustainable synthetic methodologies.

## Experimental Section

**Caution!** The procedures described in this work involve hazardous chemicals and specialized laboratory techniques. Trifluoroacetic acid (TFA) is highly corrosive and can cause severe skin burns and eye damage; it should be handled with extreme care in a well-ventilated fume hood. Diethyl ether and tetrahydrofuran (THF) are highly flammable and prone to the formation of explosive peroxides; these solvents must be tested for peroxides before use and kept away from heat or ignition sources. Dichloromethane (DCM) and acetonitrile are toxic and should be handled with appropriate personal protective equipment (PPE). All reactions using standard Schlenk and high-vacuum techniques involve pressurized or evacuated glassware, which poses a risk of implosion or explosion; users should be properly trained and use safety shielding where appropriate. No unexpected or unusually high safety risks were encountered during the synthesis of the Ru­(II) and Ir­(III) complexes.

### Materials and Methods

#### General Procedure

All reactions were performed under an argon atmosphere using standard Schlenk techniques and high vacuum, unless otherwise specified. Anhydrous solvents were dried according to established procedures.^88^ [Cp*IrCl_**2**_]_**2**_,^89–91^ **[(** *p-cymene* **)­RuCl**_**2**_]_**2**_ ^92^ precursors, **[HL** ^**1**^],^47^ **[HL** ^**2**^] ^49,50^ proligands, **[Ir-L** ^**1**^] ^52,55^ and **[Ru-L** ^**1**^] ^53,54^ complexes, were synthesized according to published methods. All other reagents were used as received from commercial suppliers (Sigma- Aldrich, Merck, and J.T. Baker).

#### NMR Spectroscopy

^1^H and ^13^C­{^1^H} NMR spectra were acquired on either a Bruker Advance III HD 400 MHz or a Jeol ECA 500 MHz spectrometer at 25 °C, unless otherwise noted, and were referenced to residual solvent signals.^93^ Spectral assignments were based on COSY, HSQC, and HMBC experiments.

#### Mass Spectrometry and Elemental Analysis

Mass spectra were obtained using an Agilent 6120 Single Quadrupole LC/MS spectrometer. Although the instrument provides data with one decimal precision, values are reported as integers (*m*/*z* nominal mass) in accordance with low-resolution spectrometry standards. Elemental analyses were performed on a Thermo-Finnigan Flash 1112 instrument.

#### Single Crystal X-ray Diffraction

Single crystals suitable for X-ray diffraction analysis were grown via slow diffusion of diethyl ether into a saturated solution of the complex. The data collection was carried out in a Bruker D8 Venture diffractometer, using graphite-monochromated Mo Kα radiation (λ = 0.71073 Å) and a Photon 100 CMOS detector. Data were collected at room temperature. Determination of unit cell parameters, and integration of frames were performed using the Bruker APEX4 software. The structure was solved by the dual space method using SHELXT and refined by full-matrix least-squares methods against *F* ^2^ by SHELXL 2019/2. Absorption corrections were performed by Multi-Scan. Modeling of **PF**_**6**_^–^ disordered moieties was done using the DSR plugin tool installed in shelXle Qt5.,^94,95^ by restraining bond lengths and displacement parameter. All non-hydrogen atoms were refined with anisotropic thermal displacement coefficients. WinGX was used for publication routines (Farrugia, 2012). Graphical representations of the crystal structures were created using Mercury (version 2025.1.0). CCDC 2471790–2471794.

#### Absorption Spectroscopy

UV–vis spectra were acquired in an Agilent Technologies Cary 8454 spectrophotometer. Quartz cuvettes 1 cm × 1 cm were used. Solutions of complexes and ligands were prepared in 1 × 10^–5^, 2 × 10^–5^, 3 × 10^–5^, 4 × 10^–5^ and 5 × 10^–5^ M to obtain ε (M^–1^ cm^–1^). The graphical representations were created with Originpro 2021.

#### Cyclic Voltammetry

The cyclic voltammograms were recorded in a conventional three-electrode cell under argon atmosphere. The work electrode was a 3 mm diameter glassy carbon disc, which was polished with alumina powder and ultrasonically rinsed with ethanol before each run. The counter electrode was a platinum mesh and the reference electrode a Saturated Calomel Electrode (SCE). A salt bridge, containing 0.1 M Bu_4_NPF_6_ (recrystallized from ethanol), acetonitrile anhydrous grade (99.8%), connected to the cell with the reference electrode. An Autolab 302N (Metrohm) potentiostat with positive feedback resistance compensation was used for the measurements, controlled via PC using software Nova 2.1.

#### General Conditions for the Catalytic Tandem α-Alkylation/Transfer Hydrogenation Reactions

In a pressure tube, the catalyst (0.025 mmol for dinuclear complexes or 0.05 mmol for mononuclear complexes) and base (0.1 mmol) were added. Acetophenone (116.65 μL, 120.15 mg, 1 mmol) and benzyl alcohol (311.94 μL, 324.42 mg, 3 mmol) in 1:3 ratio was measured using a micropipette and added to the pressure tube. A suspension was observed. A magnetic stirrer bar was added to the pressure tube. The tube was heated in a sand bath at indicated temperature, a sample was taken and analyzed by ^1^H NMR in CDCl_3_.

### Synthesis and Characterization

#### Complex **[Ir-L^2^]­PF_6_**

A mixture of **[HL** ^**2**^ **]­PF**_**6**_ (0.3 g, 0.81 mmol), Ag_2_O (0.11 g, 0.49 mmol), N­(CH_3_)_4_Cl (0.12 g, 1.2 mmol), and [Cp*IrCl_2_]_2_ (0.39 g, 0.49 mmol) was suspended in anhydrous dichloromethane (40 mL) and stirred at room temperature in the dark for 2 h. The mixture was filtered off and the filtrate solvent was removed under vacuum. The resulting solid was washed with dichloromethane (3 × 5 mL) and dried in vacuo to afford a yellow solid. Yield: 77% (0.45 g, 0.62 mmol). Crystals suitable for X-ray crystallography were obtained by slow vapor diffusion of diethyl ether into a solution of **[Ir-L** ^**2**^ **]­PF**_**6**_ in acetonitrile.

^1^H NMR (400 MHz, acetonitrile-*d*_*3*_): δ (ppm) 8.71 (ddd, *J* = 4.8, 1.8, 0.9 Hz, 1H, H15), 8.66 (ddd, *J* = 5.7, 1.6, 0.7 Hz, 1H, H10), 8.26–8.19 (m, 2H, H12, H8), 8.14 (d, *J* = 2.4 Hz, 1H, H4), 8.09 (td, *J* = 7.8, 1.8 Hz, 1H, H13), 8.03 (dt, *J* = 8.3, 1.0 Hz, 1H, H7), 7.88 (d, *J* = 2.4 Hz, 1H, H5), 7.63 (ddd, *J* = 7.5, 4.8, 1.1 Hz, 1H, H14), 7.57 (ddd, *J* = 7.3, 5.8, 1.3 Hz, 1H, H9), 1.44 (s, 15H, H16).

^13^C­{^1^H} NMR (101 MHz, acetonitrile-*d*_*3*_): δ (ppm) 167.07 (C2), 153.02 (C6), 152.92 (C10), 151.50 (C11), 150.68 (C15), 143.39 (C8), 141.04 (C13), 126.32 (C14), 125.82 (C5), 125.79 (C9), 121.89 (C12), 118.78 (C4) 113.54 (C7), 93.91 (C17), 9.16 (C16).

MS (SQ+) *m*/*z* calculated for [C_23_H_25_ClN_4_Ir]^+^: 585, found: 585.

Elemental analysis (%) calculated: C: 37.84, N: 7.67, H: 3.45; found: C: 37.69, N: 7.41, H: 3.46.

#### Complex **[Ru-L^2^]­PF_6_**

A mixture of **[HL** ^**2**^ **]­PF**_**6**_ (0.3 g, 0.81 mmol), Ag_2_O (0.11 g, 0.49 mmol), N­(CH_3_)_4_Cl (0.12 g, 1.2 mmol) and [(*p-cymene*)­RuCl_2_]_2_ (0.3 g, 0.49 mmol) was suspended in a solution of anhydrous dichloromethane (40 mL) and stirred at room temperature in the dark for 4 h. The mixture was filtered off through Celite and the solvent was removed under vacuum to afford a wine-red solid. Yield: 93% (0.48 g, 0.75 mmol). Crystals suitable for X-ray crystallography were obtained by slow vapor diffusion of diethyl ether into a solution of **[Ru-L** ^**2**^ **]­B­(Ph)**_**4**_ in acetone.

^1^H NMR (400 MHz, acetone-*d*_*6*_): δ (ppm) 9.44 (dd, *J* = 5.7, 0.9 Hz, 1H, H15), 8.85 (dd, *J* = 4.2, 1.1 Hz, 1H, H10), 8.55 (d, *J* = 2.4 Hz, 1H, H5), 8.35 (ddd, *J* = 8.3, 7.5, 1.5 Hz, 1H, H13), 8.30–8.24 (m, 3H, H7, H8, H12), 8.17 (d, *J* = 2.4 Hz, 1H, H4), 7.79 (ddd, *J* = 6.2, 4.8, 2.2 Hz, 1H, H9), 7.62 (ddd, *J* = 7.2, 5.7, 1.3 Hz, 1H, H14), 6.05 (dd, *J* = 6.2, 1.7 Hz, 1H, H18a), 5.83 (dd, *J* = 6.2, 1.3 Hz, 1H, H19a), 5.46 (dd, *J* = 6.2, 1.3 Hz, 1H, H19), 5.10 (dd, *J* = 6.1, 1.4 Hz, 1H, H18), 2.45 (sept, *J* = 6.9 Hz, 1H, H21), 2.22 (s, 3H, H16), 0.93 (dd, *J* = 29.9, 6.9 Hz, 6H, H22, H22a).

^13^C­{^1^H} NMR (101 MHz, acetone-*d*_*6*_): δ (ppm) 186.17 (C2), 156.83 (C15), 152.20 (C11), 150.77 (C10), 142.62 (C13), 140.58 (C8), 129.73 (C6), 126.36 (C9), 125.86 (C4), 124.56 (C14), 122.00 (C7), 118.38 (C5), 113.98 (C12), 111.15 (C20), 108.34 (C17), 92.90 (C18), 89.09 (C19), 88.50 (C19a), 84.33 (C18a), 31.95 (C21), 22.23 (C22), 22.16 (C22a), 19.08 (C16)

MS (SQ+) *m*/*z* calculated for [C_23_H_24_ClN_4_Ru^+^]: 493, found: 493.

Elemental analysis (%) calculated: C: 43.30, N: 8.78, H: 3.79; found: C: 43.29, N: 7.33, H: 4.16.

#### Complex **[Ir-L^3^]­PF_6_**

A mixture of **[HL** ^**3**^ **]­PF**_**6**_ (0.2 g, 0.52 mmol) and [Cp*IrCl_2_]_2_ (0.21 g, 0.26 mmol) was suspended in dichlorobenzene (40 mL) and refluxed for 24 h. The mixture was cooled to room temperature, and the subsequent addition of diethyl ether gave a dark yellow precipitate which was filtered off and dried. Yield: 62% (0.24 g, 0.32 mmol). Crystals suitable for X-ray crystallography were obtained by slow vapor diffusion of pentane into a solution of **[Ir-L** ^**3**^ **]­PF**_**6**_ in chloroform.

^1^H NMR (400 MHz, chloroform-*d*): δ (ppm) 8.71 (dd, *J* = 5.6, 1.6 Hz, 1H, H10), 8.66 (dd, *J* = 4.9, 1.8 Hz, 1H, H15), 8.25 (d, *J* = 8.6 Hz, 1H, H7), 8.17 (t, *J* = 7.7 Hz, 1H, H8), 8.07 (t, *J* = 7.8 Hz, 1H, H13), 7.86 (d, *J* = 8.0 Hz, 1H, H12), 7.56 (dd, *J* = 7.6, 4.8 Hz, 1H, H14), 7.46 (t, *J* = 6.6 Hz, 1H, H9), 7.06 (s, 1H, H4), 3.24 (s, 3H, H16), 1.78 (s, 15H, H17).

^13^C­{^1^H} NMR (101 MHz, chloroform-*d*): δ (ppm) 152.51 (C6), 152.21 (C10), 150.05 (C15), 147.94 (C11), 143.83 (C5), 143.79 (C2), 141.76 (C8), 140.21 (C13), 125.73 (C14), 125.24 (C9), 121.09 (C12), 120.10 (C4), 115.50 (C7), 90.67 (C18), 14.50 (C16), 9.17 (C17).

MS (SQ+) *m*/*z* calculated for [C_24_H_27_ClN_4_Ir^+^]: 599, found: 599.

Elemental analysis (%) calculated: C: 38.74, N: 7.53, H: 3.66; found: C: 40.04, N: 7.12, H: 3.51.

#### Complex **[Ir-L^4^]­PF_6_**

A mixture of **[HL** ^**4**^ **]­PF**_**6**_ (0.2 g, 0.45 mmol) and [Cp*IrCl_2_]_2_ (0.21 g, 0.26 mmol) was suspended in dichlorobenzene (20 mL) and refluxed for 24 h. The mixture was cooled to room temperature, and the subsequent addition of hexanes gave a mustard yellow precipitate which was filtered off and dried. Yield: 58% (0.26 g, 0.32 mmol).

^1^H NMR (400 MHz, chloroform-*d*): δ (ppm) 8.70 (dd, *J* = 5.6 Hz, 1H, H10), 8.49 (dd, *J* = 4.8, 1.8 Hz, 1H, H15), 8.19 (d, *J* = 7.6 Hz, 1H, H19), 7.78–7.67 (m, 3H, H20, H18, H8), 7.64 (t, *J* = 7.5 Hz, 1H, H13), 7.50–7.40 (m, 4H, H21, H17, H14, H12), 7.38 (s, 1H, H4), 7.36–7.30 (m, 1H, H9), 6.93 (d, *J* = 8.5 Hz, 1H, H7), 1.84 (s, 15H, H22).

^13^C­{^1^H} NMR (101 MHz, chloroform-*d*): δ (ppm) 152.46 (C6), 152.28 (C10), 149.55 (C15), 147.70 (C5), 145.04 (C2), 143.00 (C16), 140.70 (C8), 139.47 (C13), 133.05 (C19), 130.51 (C17, C21), 129.77 (C18, C20), 125.54 (C14), 125.34 (C9), 122.84 (C11), 121.43 (C12), 121.14 (C4), 114.70 (C7), 91.01 (C23), 9.26 (C22).

MS (SQ+) *m*/*z* calculated for [C_29_H_29_ClN_4_Ir^+^]: 661, found: 661.

Elemental analysis (%) calculated: C: 43.20, N: 6.95, H: 3.63; found: C: 45.70, N: 6.71, H: 3.69.

#### Complex **[Ir-L^2^-Ir]­PF_6_**

A mixture of **[Ir-L** ^**2**^ **]­PF**_**6**_ (0.2 g, 0.27 mmol), NaOAc (0.03 g, 41 mmol) and [Cp*IrCl_2_]_2_ (0.13 g, 0.16 mmol) in dichloroethane (40 mL) was stirred at reflux for 2 h. The mixture was filtered, and the solvent was removed under vacuum to obtain the crude product. Purification by flash column chromatography on silica gel using dichloromethane/methanol (10:0.5) as eluent afford a yellow solid as an isomer mixture. Yield: 89% (0.26 g, 0.24 mmol). Crystals suitable for X-ray crystallography were obtained by slow vapor diffusion of diethyl ether into a solution of **[Ir-L** ^**2**^ **-Ir]­PF**_**6**_ in acetonitrile.

**Isomer mixture:** ^1^H NMR (400 MHz, acetonitrile-*d*_*3*_): δ (ppm) 8.75–8.61 (m, 6H, H10, H12, H15, H10′, H12′, H15,), 8.21–8.13 (m, 4H, H8, H13, H8′, H13′), 8.08–7.99 (m, 2H, H7, H7′), 7.60 (s, 1H, H4′), 7.57–7.46 (m, 5H, H4, H9, H14, H9′, H14′) 1.78 (s, 15H, H18), 1.77 (s, 15H, H18′), 1.57 (s, 15H, H16), 1.56 (s, 15H, H16′).

**Isomer mixture** ^13^C­{^1^H} NMR (101 MHz, acetonitrile-*d*_*3*_): δ (ppm) 165.52 (C2), 165.04 (C2′), 154.72 (C6′), 153.85 (C10), 153.80 (C6), 153.24 (C10′), 152.96 (C15), 152.64 (C11′), 152.60 (C11), 152.19 (C15′), 148.68 (C5′), 145.92 (C5), 143.32 (C8), 143.31 (C8′), 141.96 (C13), 141.21 (C13′), 126.26 (C14), 126.04 (C14′), 124.86 (C9), 124.72 (C9′), 118.16 (C4), 116.58 (C12′), 116.53 (C4′), 116.14 (C12), 113.58 (C7), 113.44 (C7′), 93.87 (C17′), 93.70 (C17), 91.00 (C19), 90.89 (C19′), 9.32 (C18′), 9.30 (C16′), 9.29 (C18), 9.27 (C16).

MS (SQ+) *m*/*z* calculated for [C_33_H_39_Cl_2_N_4_Ir_2_^+^]: 947, found: 947.

Elemental analysis (%) calculated: C: 36.30, N: 5.13, H: 3.60; found: C: 35.66, N: 5.04, H: 3.79.

#### Complex **[Ir-L^2^-Ru]­PF_6_**

A mixture of **[Ir-L** ^**2**^ **]­PF**_**6**_ (0.2 g, 0.27 mmol), Ag_2_O (0.04 g, 0.16 mmol), N­(CH_3_)_4_Cl (0.04 g, 0.41 mmol), NaOAc (0.03 g, 0.41 mmol) and [(*p-cymene*)­RuCl_2_]_2_ (0.1 g, 0.16 mmol) in dichloroethane (40 mL) was stirred and refluxed in the dark for 18 h. The mixture was filtered, and the solvent was removed under vacuum to obtain the crude product. Purification by flash column chromatography on silica gel using dichloromethane/methanol (10:0.5) as eluent afforded a dark green solid as an isomer mixture. Yield: 89% (0.26 g, 0.24 mmol). The isomer mixture was separated *via* slow diffusion of diethyl ether into a saturated solution of acetonitrile, where isomer A precipitated, and isomer B remained in the mother liquor. Crystals suitable for X-ray crystallography were obtained by slow vapor diffusion of diethyl ether into a solution of **[Ir-L** ^**2**^ **-Ru]­PF**_**6**_ in acetone/dichloromethane mixture.

**Isomer A**. ^1^H NMR (400 MHz, acetonitrile-*d*_*3*_): δ (ppm) 9.26 (d, *J* = 5.5 Hz, 1H, H15), 8.63 (d, *J* = 5.7 Hz, 1H, H10), 8.51 (d, *J* = 8.2 Hz, 1H, H12), 8.19 (t, *J* = 8.0 Hz, 1H, H8), 8.11 (t, *J* = 7.9 Hz, 1H, H13), 7.95 (s, 1H, H4), 7.94 (d, *J* = 7.9 Hz, 1H, H7), 7.54–7.44 (m, 2H, H9, H14), 5.91 (t, *J* = 6.1 Hz, 2H, H20a, H21a), 5.55 (d, *J* = 6.0 Hz, 1H, H21), 5.29 (d, *J* = 6.1 Hz, 1H, H20), 2.60 (sept, *J* = 7.2 Hz, 1H, H23), 2.11 (s, 3H, H18), 1.57 (s, 15H, H16), 1.05 (dd, *J* = 61.3, 6.9 Hz, 6H, H24, H24a)

**Isomer A** ^13^C­{^1^H} NMR (101 MHz, acetonitrile-*d*_*3*_): δ (ppm) 164.42 (C2), 160.20 (C5), 157.07 (C15), 153.82 (C6), 153.19 (C12), 152.35 (C11), 143.40 (C8), 141.44 (C13), 125.02 (C14), 124.92 (C9), 120.62 (C7), 116.24 (C10), 113.44 (C4), 105.18 (C19), 104.89 (C22), 93.72 (C17), 91.83 (C21a), 88.46 (C20a), 83.87 (C21), 83.45 (C20), 31.64 (C23), 22.95 (C24), 22.09 (C24a), 18.86 (C18), 9.36 (C16).

**Isomer B**. ^1^H NMR (400 MHz, acetonitrile-*d*_*3*_): δ (ppm) 9.27 (d, *J* = 4.8 Hz, 1H, H15), 8.61 (d, *J* = 5.6 Hz, 1H, H10), 8.55 (d, *J* = 8.2 Hz, 1H, H12), 8.19 (t, *J* = 7.5 Hz, 1H, H8), 8.11 (t, *J* = 7.5 Hz, 1H, H13), 8.00 (s, 1H, H4), 7.96 (d, *J* = 7.9 Hz, 1H, H7), 7.51–7.42 (m, 2H, H9, H14), 5.99 (d, *J* = 6.0 Hz, 1H, H21a), 5.86 (d, *J* = 6.0 Hz, 1H, H20a), 5.43 (d, *J* = 5.5 Hz, 1H, H21), 5.20 (d, *J* = 6.0 Hz, 1H, H20), 2.61 (sept, *J* = 7.0 Hz, 1H, H23), 2.04 (s, 3H, H18), 1.52 (s, 15H, H16), 1.00 (dd, *J* = 72.9, 6.9 Hz, 6H, H24, H24a)

**Isomer B** ^13^C­{^1^H} NMR (101 MHz, acetonitrile-*d*_*3*_): δ (ppm) 165.01 (C2), 162.47 (C5), 156.01 (C15), 154.29 (C6), 153.23 (C12), 152.40 (C11), 143.36 (C8), 141.06 (C13), 124.90 (C14), 124.71 (C9), 119.66 (C7), 116.34 (C10), 113.20 (C4), 106.09 (C19), 103.29 (C22), 93.77 (C17), 90.00 (C21a), 89.93 (C20a), 85.09 (C20), 83.44 (C21), 31.61 (C23), 22.58 (C24), 22.07 (C24a), 18.86 (C18), 9.27 (C16).

MS (SQ+) *m*/*z* calculated for [C_33_H_38_Cl_2_N_4_IrRu^+^]: 855, found: 855.

Elemental analysis (%) calculated: C: 39.64, N: 5.60, H: 3.83; found: C: 39.14, N: 5.42, H: 3.86.

#### Complex **[Ru-L^2^-Ir]­PF_6_**

A mixture of **[Ru-L** ^**2**^ **]­PF**_**6**_ (0.2 g, 0.31 mmol), Ag_2_O (0.04 g, 0.19 mmol), N­(CH_3_)_4_Cl (0.05 g, 0.47 mmol), NaOAc (0.04 g, 0.47 mmol) and [Cp*IrCl_2_]_2_ (0.15 g, 0.19 mmol) in dichloroethane (40 mL) was stirred at 50 °C, in an oil bath, in the dark for 18 h. The mixture was filtered off, the solvent was removed under vacuum to obtain the crude product. Purification by flash column chromatography on silica gel using dichloromethane/methanol (10:0.5) as eluent afforded a dark green solid as an isomer mixture. Isomer separation was attempted by slow vapor diffusion of diethyl ether into a saturated solution of acetone, but it was unsuccessful. Nevertheless, the precipitate was pure **[Ru-L** ^**2**^ **-Ir]­PF**_**6**_ isomer mixture. Yield: 53% (0.16 g, 0.16 mmol).

**Isomer mixture:** ^1^H NMR (400 MHz, acetone-*d*_*6*_): δ (ppm) 9.37 (d, *J* = 4.2 Hz, 1H, H15), 8.96 (d, *J* = 5.6 Hz, 1H, H15F), 8.88 (d, *J* = 5.8 Hz, 1H, H10), 8.84 (d, *J* = 5.1 Hz, 1H, H10′), 8.38–8.18 (m, 8H, H7, H7′, H8, H8′, H12, H12′, H13, H13′), 7.94 (s, 1H, H4′), 7.91 (s, 1H, H4), 7.64 (t, *J* = 6.3 Hz, 1H, H9′), 7.59 (t, *J* = 6.5 Hz, 1H, H9), 7.58–7.49 (m, 2H, H14, H14′), 6.44 (d, *J* = 6.0 Hz, 1H, H18a), 6.29 (d, *J* = 6.0 Hz, 1H, 18a′), 6.20 (d, *J* = 6.1 Hz, 1H, H18), 6.07 (d, *J* = 6.1 Hz, 1H, 18′), 5.78 (d, *J* = 6.0 Hz, 1H, H19a), 5.72 (d, *J* = 6.0 Hz, 1H, H19a′), 5.65–5.59 (m, 2H, H19, H19′), 2.66–2.53 (m, 1H, H21′), 2.49–2.37 (m, 1H, H21), 2.33 (s, 3H, H16), 2.24 (s, 3H, H16′), 1.85 (s, 15H, H23), 1.83 (s, 15H, H23′), 1.06 (d, *J* = 6.8 Hz, 3H, H22′), 0.96 (d, *J* = 6.8 Hz, 3H, H22), 0.88 (m, 6H, H22a, H22a′).

**Isomer mixture** ^13^C­{^1^H} NMR (101 MHz, acetone-*d*_*6*_): δ (ppm) 183.86 (C2′), 183.18 (C2), 156.70 (C15) 156.64 (C11), 155.45 (C10′), 154.99 (C11′), 153.83 (C10), 152.73 (C15′), 152.26 (C6), 152.22 (C6′), 151.74 (C12), 151.63 (C12′), 146.85 (C5), 146.85 (C5′), 142.33 (C8), 142.31 (C13), 141.90 (C8′), 141.46 (C13′), 125.67 (C9), 125.63 (C9′), 123.65 (C14), 123.57 (C14′), 117.46 (C4), 117.42 (C4′), 116.43 (C7), 116.03 (C7′), 113.84 (C20), 113.81 (C20′), 113.65 (C17), 113.44 (C17′), 93.00 (C19a), 92.70 (C19a′), 90.67 (C24′), 90.67 (C24), 90.19 (C18a′), 89.37 (C18a), 88.07 (C18), 86.44 (C18′), 85.92 (C19), 84.64 (C19′), 32.03 (C21′), 32.00 (C21), 22.90 (C22′), 22.78, 22.53 (C22′), 22.40 (C22a), 21.97 (C22a′), 18.97 (C16), 18.97 (C16a′), 9.17 (C23), 8.81 (C23′).

MS (SQ+) *m*/*z* calculated for [C_33_H_38_Cl_2_N_4_IrRu^+^]: 855, found: 855.

Elemental analysis (%) calculated: C: 39.64, N: 5.60, H: 3.83; found: C: 39.58, N: 5.38, H: 3.86.

## Supplementary Material



## Notes

Literature review; synthetic procedures and characterization of ligands and complexes, experimental details on catalytic and kinetic experiments, absorption spectroscopy and electrochemistry (PDF)

## References

[1] Hopkinson M. N., Richter C., Schedler M., Glorius F. (2014). An Overview of N-Heterocyclic
Carbenes. Nature.

[2] Herrmann W. A., Köcher C. (1997). N-Heterocyclic Carbenes. Angew.
Chem., Int. Ed..

[3] Herrmann W. A. (2002). N-Heterocyclic
Carbenes: A New Concept in Organometallic Catalysis. Angew. Chem. Int..

[4] de Frémont P., Marion N., Nolan S. P. (2009). Carbenes: Synthesis,
Properties,
and Organometallic Chemistry. Coord. Chem. Rev..

[5] Díez-González S., Nolan S. P. (2007). Stereoelectronic Parameters Associated with N-Heterocyclic
Carbene (NHC) Ligands: A Quest for Understanding. Coord. Chem. Rev..

[6] Gómez-Suárez A., Nelson D. J., Nolan S. P. (2017). Quantifying and Understanding the
Steric Properties of N-Heterocyclic Carbenes. Chem. Commun..

[7] Peris E. (2018). Smart N-Heterocyclic
Carbene Ligands in Catalysis. Chem. Rev..

[8] Koy M., Bellotti P., Das M., Glorius F. (2021). N-Heterocyclic Carbenes
as Tunable Ligands for Catalytic Metal Surfaces. Nat. Catal..

[9] Gründemann S., Kovacevic A., Albrecht M., Faller J. W. R., Crabtree H. (2001). Abnormal Binding
in a Carbene Complex Formed from an Imidazolium Salt and a Metal Hydride
Complex. Chem. Commun..

[10] Vivancos Á., Segarra C., Albrecht M. (2018). Mesoionic and Related Less Heteroatom-Stabilized
N-Heterocyclic Carbene Complexes: Synthesis, Catalysis, and Other
Applications. Chem. Rev..

[11] Crabtree R. H. (2013). Abnormal,
Mesoionic and Remote N-Heterocyclic Carbene Complexes. Coord. Chem. Rev..

[12] Arnold P. L., Pearson S. (2007). Abnormal N-Heterocyclic Carbenes. Coord. Chem. Rev..

[13] Albrecht M. (2008). C4-Bound Imidazolylidenes:
From Curiosities to High-Impact Carbene Ligands. Chem. Commun..

[14] Aldeco-Perez E., Rosenthal A. J., Donnadieu B., Parameswaran P., Frenking G., Bertrand G. (2009). Isolation of a C5-Deprotonated Imidazolium,
a Crystalline “Abnormal” N-Heterocyclic Carbene. Science.

[15] Melaimi M., Soleilhavoup M., Bertrand G. (2010). Stable Cyclic Carbenes
and Related
Species beyond Diaminocarbenes. Angew. Chem.,
Int. Ed..

[16] Albrecht M. (2014). Normal and
Abnormal N-Heterocyclic Carbene Ligands. Similarities and Differences
of Mesoionic C-Donor Complexes. Adv. Organomet.
Chem..

[17] Mercs L., Albrecht M. (2010). Beyond Catalysis: N-Heterocyclic Carbene Complexes
as Components for Medicinal, Luminescent, and Functional Materials
Applications. Chem. Soc. Rev..

[18] Mück-Lichtenfeld C., Grimme S. (2012). Theoretical Analysis
of Cooperative Effects of Small
Molecule Activation by Frustrated Lewis Pairs. Dalton Trans..

[19] Becker S. (2024). Understanding
Cooperativity in Homo- and Heterometallic Complexes: From Basic Concepts
to Design. ChemPluschem.

[20] Tebben L., Mück-Lichtenfeld C., Fernández G., Grimme S., Studer A. (2017). From Additivity to Cooperativity
in Chemistry: Can Cooperativity Be Measured?. Chem. Eur. J..

[21] Allen A. E., MacMillan D. W. C. (2012). Synergistic Catalysis: A Powerful
Synthetic Strategy
for New Reaction Development. Chem. Sci..

[22] Van Der Vlugt J. I. (2012). Cooperative
Catalysis with First-Row Late Transition Metals. Eur. J. Inorg. Chem..

[23] Park J., Hong S. (2012). Cooperative Bimetallic Catalysis in Asymmetric Transformations. Chem. Soc. Rev..

[24] Mankad N. P. (2016). Selectivity
Effects in Bimetallic Catalysis. Chem. Eur.
J..

[25] Powers I. G., Uyeda C. (2017). Metal-Metal Bonds in Catalysis. ACS Catal.

[26] Maity R., Birenheide B. S., Breher F., Sarkar B. (2021). Cooperative Effects
in Multimetallic Complexes Applied in Catalysis. ChemCatchem.

[27] Stevens M.
A., Colebatch A. L. (2022). Cooperative
Approaches in Catalytic Hydrogenation and
Dehydrogenation. Chem. Soc. Rev..

[28] Buchwalter P., Rosé J., Braunstein P. (2015). Multimetallic
Catalysis Based on
Heterometallic Complexes and Clusters. Chem.
Rev..

[29] Charra V., Frémont P. D., Braunstein P. (2017). Multidentate N-Heterocyclic Carbene
Complexes of the 3d Metals: Synthesis, Structure, Reactivity and Catalysis. Coord. Chem. Rev..

[30] Czégéni C. E., Joó F., Kathó Á., Papp G. (2023). Heterobimetallic Complexes
of Bi- or Polydentate N-Heterocyclic Carbene Ligands and Their Catalytic
Properties. Catalysts.

[31] Tiwari, C. S. ; Dey, A. ; Rit, A. Synthesis and Catalytic Applications of Heterobimetallic Complexes Involving Bis-N-Heterocyclic Carbenes. In Organometallic Chemistry; Royal Society of Chemistry, 2024; Vol. 45, pp. 1–34. 10.1039/9781837676200-00001.

[32] Zanardi A., Corberán R., Mata J. A., Peris E. (2008). Homo- And Heterodinuclear
Complexes with Triazolyl-Diylidene. An Easy Approach to Tandem Catalysts. Organometallics.

[33] Zanardi A., Mata J. A., Peris E. (2009). Well-Defined Ir/Pd Complexes with
a Triazolyl-Diylidene Bridge as Catalysts for Multiple Tandem Reactions. J. Am. Chem. Soc..

[34] Leung J. N., Huynh H. V. (2024). Mesoionic Janus-Type Dicarbene: Complexes,
Adducts,
and Catalytic Studies. Chem. Eur. J..

[35] Leung J. N., Huynh H. V. (2025). Dinuclear Gold­(I) and Gold­(III) Complexes of Janus
Di-N-Heterocyclic Carbenes. Inorg. Chem..

[36] Boydston A. J., Williams K. A., Bielawski C. W. (2005). A Modular
Approach to Main-Chain
Organometallic Polymers. J. Am. Chem. Soc..

[37] Tennyson A. G., Rosen E. L., Collins M. S., Lynch V. M., Bielawski C. W. (2009). Bimetallic
N-Heterocyclic Carbene-Lridium Complexes: Investigating Metal-Metal
and Metal-Ligand Communication via Electrochemistry and Phosphorescence
Spectroscopy. Inorg. Chem..

[38] Poyatos M., Peris E. (2021). Insights into the Past and Future
of Janus-Di-N-Heterocyclic Carbenes. Dalton
Trans..

[39] Sabater S., Mata J. A., Peris E. (2012). Dual Catalysis with
an Ir III −Au
I Heterodimetallic Complex: Reduction of Nitroarenes by Transfer Hydrogenation
using Primary Alcohols. Chem. Eur. J..

[40] Sabater S., Mata J. A., Peris E. (2013). Hydrodefluorination of Carbon-Fluorine
Bonds by the Synergistic Action of a Ruthenium-Palladium Catalyst. Nat. Commun..

[41] Grineva A. A., Filippov O. A., Canac Y., Sortais J. B., Nefedov S. E., Lugan N., César V., Valyaev D. A. (2021). Experimental and
Theoretical Insights into the Electronic Properties of Anionic N-Heterocyclic
Dicarbenes through the Rational Synthesis of Their Transition Metal
Complexes. Inorg. Chem..

[42] Bitzer M. J., Pöthig A., Jandl C., Kühn F. E., Baratta W. (2015). Ru-Ag and Ru-Au Dicarbene Complexes from an Abnormal
Carbene Ruthenium System. Dalton Trans..

[43] Rottschäfer D., Ebeler F., Strothmann T., Neumann B., Stammler H. G., Mix A., Ghadwal R. S. (2018). The Viability
of C5-Protonated- and C4,C5-Ditopic Carbanionic
Abnormal NHCs: A New Dimension in NHC Chemistry. Chem. Eur. J..

[44] Ellul C. E., Mahon M. F., Saker O., Whittlesey M. K. (2007). Abnormally
Bound N-Heterocyclic Carbene Complexes of Ruthenium: C-H Activation
of Both C4 and C5 Positions in the Same Ligand. Angew. Chem., Int. Ed..

[45] Hu Z., Ma X., Wang J., Wang H., Han X., Shi M., Zhang J. (2019). Six-Membered Janus-Type Ditopic N-Heterocyclic Carbene Coinage Metal
Complexes. Organometallics.

[46] Leung J. N., Huynh H. V. (2024). Design of a Mesoionic Janus-Type
Dicarbene. J. Am. Chem. Soc..

[47] Catalano V. J., Etogo A. O. (2005). Luminescent Coordination Polymers
with Extended Au­(I)-Ag­(I)
Interactions Supported by a Pyridyl-Substituted NHC Ligand. J. Organomet. Chem..

[48] Peters M., Breinbauer R. (2010). A Simple Synthesis of Functionalized
3-Methyl-1-Pyridinyl-1H-Imidazolium
Salts as Bidentate N-Heterocyclic-Carbene Precursors and Their Application
in Ir-Catalyzed Arene Borylation. Tetrahedron
Lett..

[49] Chen J. C.
C., Lin I. J. B. (2000). Palladium
Complexes Containing a Hemilabile Pyridylcarbene
Ligand. Organometallics.

[50] Lee K. M., Chen J. C. C., Lin I. J. B. (2001). Helical Mono
and Dinuclear Mercury­(II)
N-Heterocyclic Carbene Complexes. J. Organomet.
Chem..

[51] Riener K., Bitzer M. J., Pöthig A., Raba A., Cokoja M., Herrmann W. A., Kühn F. E. (2014). On the Concept of Hemilability: Insights
into a Donor-Functionalized Iridium­(I) NHC Motif and Its Impact on
Reactivity. Inorg. Chem..

[52] Xiao X. Q., Jin G. X. (2008). Functionalized N-Heterocyclic Carbene
Iridium Complexes:
Synthesis, Structure and Addition Polymerization of Norbornene. J. Organomet. Chem..

[53] Leigh V., Ghattas W., Lalrempuia R., Müller-Bunz H., Pryce M. T., Albrecht M. (2013). Synthesis, Photo-,
and Electrochemistry
of Ruthenium Bis­(Bipyridine) Complexes Comprising a N-Heterocyclic
Carbene Ligand. Inorg. Chem..

[54] Movassaghi S., Singh S., Mansur A., Tong K. K. H., Hanif M., Holtkamp H. U., Söhnel T., Jamieson S. M. F., Hartinger C. G. (2018). (Pyridin-2-Yl)-NHC
Organoruthenium Complexes: Antiproliferative Properties and Reactivity
toward Biomolecules. Organometallics.

[55] Feng X., Huang M. (2021). Effect of the Ancillary Ligand in
N-Heterocyclic Carbene Iridium­(III)
Catalyzed N-Alkylation of Amines with Alcohols. Polyhedron.

[56] Lin I. J. B., Vasam C. S. (2007). Preparation and Application of N-Heterocyclic
Carbene
Complexes of Ag­(I). Coord. Chem. Rev..

[57] Valencia M., Müller-Bunz H., Gossage R. A., Albrecht M. (2016). Enhanced Product
Selectivity
Promoted by Remote Metal Coordination in Acceptor-Free Alcohol Dehydrogenation
Catalysis. Chem. Commun..

[58] Petronilho A., Woods J. A., Mueller-Bunz H., Bernhard S., Albrecht M. (2014). Iridium Complexes
Containing Mesoionic C Donors: Selective C­(Sp3)-H versus C­(Sp2)-H
Bond Activation, Reactivity towards Acids and Bases, and Catalytic
Oxidation of Silanes and Water. Chem. Eur. J..

[59] Petronilho A., Woods J. A., Bernhard S., Albrecht M. (2014). Bimetallic Iridium–Carbene
Complexes with Mesoionic Triazolylidene Ligands for Water Oxidation
Catalysis. Eur. J. Inorg. Chem..

[60] Meier M., Tan T. T. Y., Hahn F. E., Huynh H. V. (2017). Donor Strength Determination
of Benzoxazolin-2-Ylidene, Benzobisoxazolin-2-Ylidene, and Their Isocyanide
Precursors by 13C NMR Spectroscopy of Their PdII and AuI Complexes. Organometallics.

[61] Kumar S., Patra D. K., Rit A. (2023). Heterobimetallic Complexes Bridged
by an Unsymmetrical Bis­(NHC) Ligand: Study of Enhanced Catalytic Activity
in Tandem Transformations and Understanding of Cooperativity between
the Metal Centers. Chem. Eur. J..

[62] Barbante G. J., Doeven E. H., Francis P. S., Stringer B. D., Hogan C. F., Kheradmand P. R., Wilson D. J. D., Barnard P. J. (2015). Iridium­(III) N-Heterocyclic
Carbene Complexes: An Experimental and Theoretical Study of Structural,
Spectroscopic, Electrochemical and Electrogenerated Chemiluminescence
Properties. Dalton Trans..

[63] Siek S., Burks D. B., Gerlach D. L., Liang G., Tesh J. M., Thompson C. R., Qu F., Shankwitz J. E., Vasquez R. M., Chambers N., Szulczewski G. J., Grotjahn D. B., Webster C. E., Papish E. T. (2017). Iridium and Ruthenium
Complexes of N-Heterocyclic Carbene- and Pyridinol-Derived Chelates
as Catalysts for Aqueous Carbon Dioxide Hydrogenation and Formic Acid
Dehydrogenation: The Role of the Alkali Metal. Organometallics.

[64] Sawkmie M., Bhattacharyya M., Banothu V., Kaminsky W., Gannon P. M., Majaw S., Kollipara M. R. (2023). Ruthenium, Rhodium, and Iridium Complexes
Featuring Fluorenyl Benzohydrazone Derivatives: Synthesis and Preliminary
Investigation of Their Anticancer and Antibacterial Activity. J. Mol. Struct..

[65] Zahirović A., Fetahović S., Feizi-Dehnayebi M., Višnjevac A., Bešta-Gajević R., Kozarić A., Martić L., Topčagić A., Roca S. (2024). Dual Antimicrobial-Anticancer
Potential, Hydrolysis, and DNA/BSA Binding Affinity of a Novel Water-Soluble
Ruthenium-Arene Ethylenediamine Schiff Base (RAES) Organometallic. Spectrochim. Acta - Part A Mol. Biomol. Spectrosc..

[66] Hintermair U., Campos J., Brewster T. P., Pratt L. M., Schley N. D., Crabtree R. H. (2014). Hydrogen-Transfer Catalysis with
Cp*IrIII Complexes:
The Influence of the Ancillary Ligands. ACS
Catal.

[67] Illam P. M., Donthireddy S. N. R., Chakrabartty S., Rit A. (2019). Heteroditopic Ru­(II)-And
Ir­(III)-NHC Complexes with Pendant 1,2,3-Triazole/Triazolylidene Groups:
Stereoelectronic Impact on Transfer Hydrogenation of Unsaturated Compounds. Organometallics.

[68] Petronilho A., Mueller-Bunz H., Albrecht M. (2015). Iridium, Ruthenium, and Palladium
Complexes Containing a Mesoionic Fused Imidazolylidene Ligand. J. Organomet. Chem..

[69] Maji B., Bhandari A., Bhattacharya D., Choudhury J. (2022). Reusable Single
Homogeneous Ir­(III)-NHC Catalysts for Bidirectional Hydrogenation-Dehydrogenation
of N-Heteroarenes in Water. Organometallics.

[70] Olguín J., Müller-Bunz H., Albrecht M. (2014). Springloaded Porphyrin NHC Hybrid
Rhodium­(III) Complexes: Carbene Dissociation and Oxidation Catalysis. Chem. Commun..

[71] Anton D. R., Crabtree R. H. (1983). Dibenzo­[a,e]­Cyclooctatetraene in a Proposed Test for
Heterogeneity in Catalysts Formed from Soluble Platinum Group Metal
Complexes. Organometallics.

[72] Widegren J. A., Bennett M. A., Finke R. G. (2003). Is It Homogeneous
or Heterogeneous
Catalysis? Identification of Bulk Ruthenium Metal as the True Catalyst
in Benzene Hydrogenations Starting with the Monometallic Precursor,
Ru­(II)­(Η6-C6Me 6)­(OAc)­2, plus Kinetic Characterization of the
Heterogeneous Nucle. J. Am. Chem. Soc..

[73] Roy B. C., Chakrabarti K., Shee S., Paul S., Kundu S. (2016). Bifunctional
RuII-Complex-Catalysed Tandem C–C Bond Formation: Efficient
and Atom Economical Strategy for the Utilisation of Alcohols as Alkylating
Agents. Chem. Eur. J..

[74] Dehury N., Mishra S. R., Laha P., Patra S. (2020). Tandem α/β-Alkylation
and Transfer Hydrogenation by Heterodimetallic Ruthenium-Iridium Complex. Inorg. Chim. Acta.

[75] Yadav S., Rao Kuram M. (2023). Ir-Catalyzed
Transfer Hydrogenation and Alkylation
of Aldehydes and Ketones Using Ethanol as the Hydrogen Source. Eur. J. Org. Chem..

[76] Mechrouk V., Maisse-François A., Bellemin-Laponnaz S., Achard T. (2023). β-Alkylation through Dehydrogenative Coupling
of Primary Alcohols and Secondary Alcohols Catalyzed by Thioether-Functionalized
N-Heterocyclic Carbene Ruthenium Complexes. Eur. J. Inorg. Chem..

[77] Das J., Vellakkaran M., Banerjee D. (2019). Nickel-Catalyzed Alkylation of Ketone
Enolates: Synthesis of Monoselective Linear Ketones. J. Org. Chem..

[78] Wang D., McBurney R. T., Pernik I., Messerle B. A. (2019). Controlling
the
Selectivity and Efficiency of the Hydrogen Borrowing Reaction by Switching
between Rhodium and Iridium Catalysts. Dalton
Trans..

[79] Jones N. D., James B. R. (2002). Homo- and Heterobimetallic
Precursor Catalysts for
the Heck Reaction, and a Proposal for a General Catalytic Cooperativity
Index. Adv. Synth. Catal..

[80] Kaur M., Din Reshi N., Patra K., Bhattacherya A., Kunnikuruvan S., Bera J. K. (2021). A Proton-Responsive Pyridyl­(Benzamide)-Functionalized
NHC Ligand on Ir Complex for Alkylation of Ketones and Secondary Alcohols. Chem. Eur. J..

[81] Wong C. M., McBurney R. T., Binding S. C., Peterson M. B., Gonçales V. R., Gooding J. J., Messerle B. A. (2017). Iridium­(III)
Homo- and Heterogeneous
Catalysed Hydrogen Borrowing C-N Bond Formation. Green Chem..

[82] Liu J., Li W., Li Y., Liu Y., Ke Z. (2021). Selective C-Alkylation
Between Alcohols Catalyzed by N-Heterocyclic Carbene Molybdenum. Chem. - An Asian J..

[83] Martínez R., Ramón D. J., Yus M. (2006). Easy α-Alkylation of Ketones
with Alcohols through a Hydrogen Autotransfer Process Catalyzed by
RuCl2­(DMSO)­4. Tetrahedron.

[84] Nurmakanova A., Salischeva A., Chudinova A., Ivashkina E., Syskina A. (2014). Comparison between Alkylation and
Transalkylation Reactions
Using Ab Initio Approach. Proc. Chem..

[85] Kirinde Arachchige P. T., Handunneththige S., Talipov M. R., Kalutharage N., Yi C. S. (2021). Scope and Mechanism
of the Redox-Active 1,2-Benzoquinone Enabled
Ruthenium-Catalyzed Deaminative α-Alkylation of Ketones with
Amines. ACS Catal.

[86] Cavazzini M., Quici S., Scalera C., Puntoriero F., La Ganga G., Campagna S. (2009). Synthesis, Characterization, Absorption
Spectra, and Luminescence Properties of Multinuclear Species Made
of Ru­(II) and Ir­(III) Chromophores. Inorg. Chem..

[87] Tripathy S. K., De U., Dehury N., Pal S., Kim H. S., Patra S. (2014). Dinuclear
[{(p-Cym)­RuCl}­2­(μ-Phpy)]­(PF6)­2 and Heterodinuclear (Ppy)­2Ir­(μ-Phpy)­Ru­(p-Cym)­Cl]­(PF6)­2
Complexes: Synthesis, Structure and Anticancer Activity. Dalton Trans..

[88] Armarego, W. L. F. Purification of Laboratory Chemicals; Elsevier: Australia, 2017.

[89] Kang J. W., Moseley K., Maitiis P. M. (1969). Pentamethylcyclopentadienylrhodium
and -Iridium Halides. I. Synthesis and Properties. J. Am. Chem. Soc..

[90] White C., Yates A., Maitlis P. M., Heinekey D. M. (1992). (Η5 -Pentamethylcyclopentadienyl)­Rhodium
and -Iridium Compounds. Inorg. Synth..

[91] Ball R. G., Graham W. A. G., Heinekey D. M., Hoyano J. K., McMaster A. D., Mattson B. M., Michel S. T. (1990). Synthesis and Structure
of Dicarbonylbis­(.Eta.-Pentamethylcyclopentadienyl)­Diiridium. Inorg. Chem..

[92] De La Cruz-Cruz J. I., Paz-Sandoval M. A. (2015). Ruthenium
Complexes Containing Hexamethylbenzene and
Butadienesulfonyl Ligands: Synthesis and Reactivity toward CO, Nitrogen
and Phosphine Ligands. J. Organomet. Chem..

[93] Fulmer G. R., Miller A. J. M., Sherden N. H., Gottlieb H. E., Nudelman A., Stoltz B. M., Bercaw J. E., Goldberg K. I. (2010). NMR Chemical
Shifts
of Trace Impurities: Common Laboratory Solvents, Organics, and Gases
in Deuterated Solvents Relevant to the Organometallic Chemist. Organometallics.

[94] Kratzert D., Holstein J. J., Krossing I. (2015). DSR: Enhanced Modelling
and Refinement
of Disordered Structures with SHELXL. J. Appl.
Crystallogr..

[95] Kratzert D., Krossing I. (2018). Recent Improvements
in DSR. J.
Appl. Crystallogr..

