# Supplementary material for: Modular Synthesis of Ru(II)–Ir(III) Complexes Bridged by a Ditopic Carbanionic NHC Ligand: Influence of Heterodinuclear Architecture on Tandem Catalysis
Source: Inorg Chem. 2026 Jun 14;65(25):13839–51. doi: 10.1021/acs.inorgchem.6c00615 (PMC13321304; doi:10.1021/acs.inorgchem.6c00615)
Supplement: Supplementary file 1 [file ic6c00615_si_001.pdf]

SUPPORTING INFORMATION

for

Modular Synthesis of Ru(II)-Ir(III) Complexes Bridged by a Ditopic Carbanionic NHC Ligand: Influence of Heterodinuclear Architecture on Tandem Catalysis

Genesis Ramos-Guzmán[a], Juan Olguín[a]*

[a] Departamento de Química, Centro de Investigación y de Estudios Avanzados del Instituto Politécnico Nacional (Cinvestav), Avenida IPN 2508, Col. San Pedro Zacatenco, Ciudad de México 07360, México. e-mail jolguin@cinvestav.mx

Contents

Examples of dinuclear metal complexes containing different ditopic NHC-based ligands, showing cooperativity in tandem catalysis ... 4

Synthesis and characterization ... 8

2-methyl-1,3-bis(2-pyridyl) imidazolium hexafluorophosphate $[HL^3]PF_6$ ... 8

2-phenyl-1,3-bis(2-pyridyl) imidazolium hexafluorophosphate $[HL^4]PF_6$ ... 10

^1H and ^{13}C NMR spectra of complexes ... 12

$[Ir\text{-}L^2]PF_6$ ... 12

$[Ru\text{-}L^2]PF_6$ ... 13

$[Ru\text{-}L^2]B(Ph)_4$ ... 14

$[Ir\text{-}L^3]PF_6$ ... 15

$[Ir\text{-}L^4]PF_6$ ... 16

$[Ir\text{-}L^2\text{-}Ir]PF_6$ ... 17

$[Ir\text{-}L^2\text{-}Ru]PF_6$ ... 18

$[Ru\text{-}L^2\text{-}Ir]PF_6$ ... 20

Synthesis of deuterated substrates ... 21

Experiment with trifluoroacetic acid ... 28

Single crystal X ray crystallography ... 31

Structural refinement details for complexes ... 35

Catalytic studies ... 37

Mechanism for the α-alkylation/transfer hydrogenation reaction proposed in previous reports ... 37

General conditions for the catalytic tandem α-alkylation/transfer hydrogenation reactions ... 40

Analysis of optimization of reaction conditions for tandem α-alkylation/transfer hydrogenation ... 41

Self-condensation product of acetophenone ... 41

Optimization of reaction conditions for tandem α-alkylation/transfer hydrogenation ... 43

TOF ... 45

TON ... 45

Experiments 1:1 ratio of acetophenone and benzyl alcohol ... 46

Cooperative index ($\boldsymbol{a}$)[49] ... 48

Scope of ketones and alcohols ... 49

Byproduct observed in Table S5 Entry 2: ... 51

Mercury test to probe homogeneous vs heterogeneous catalysis ... 52

Control experiments and deuterium labeling studies. ... 52

Hydride species 52
Benzaldehyde instead of benzyl alcohol 54
Deuterated experiments 54
Calculating deuterium incorporation in products 55
Analysis of control experiments and deuterium labeling studies. 67
Arrhenius analysis 68
Analysis of the kinetic results to determine the activation energy Ea 72
Calculation of activation energy 78
Equations to quantify cooperativity using Ea: [51,52] 81
Absorption spectroscopy 82
Electrochemistry 86
References 93

Examples of dinuclear metal complexes containing different ditopic NHC-based ligands, showing cooperativity in tandem catalysis

Some examples are illustrated in Figure S1, (references for each example: **A**[1–4], **B**[5,6], **C**[7,8], **D**[9], **E**[10–12], **F**[13–15]), however, the majority of examples in the literature correspond to dinucleating ligands containing two different NHC-sites separated by a bridge. Some examples of this latter type of ligands that are capable of maintaining the metal centers in close proximity are shown in Figure S1, ligands **G**[16,17] and **H**[18]. While cooperative catalysis has been observed in some complexes with these bridging ligands, the quantification of this cooperativity remains unreported.

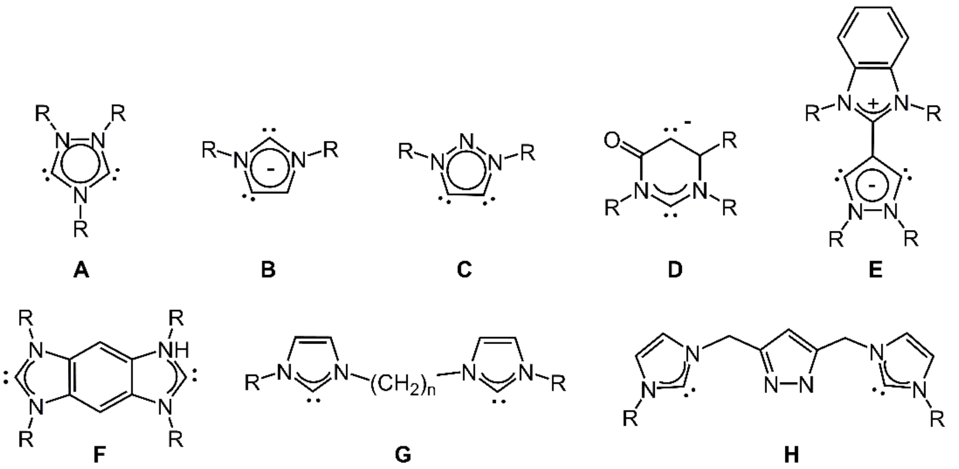

Figure S1 Selected examples of ditopic NHC-based ligands utilized for the synthesis of dimetallic complexes

Table S1 Examples of dinuclear metal complexes containing different ditopic NHC-based ligands, showing cooperativity in tandem catalysis.[a]

Entry	Tandem Catalysis	Catalyst: Mono / homodinuclear (% Yield) [b]	Catalyst: Heterodinuclear (% Yield)	Ref
1	Suzuki−Miyaura coupling / transfer hydrogenation of p-bromoacetophenone 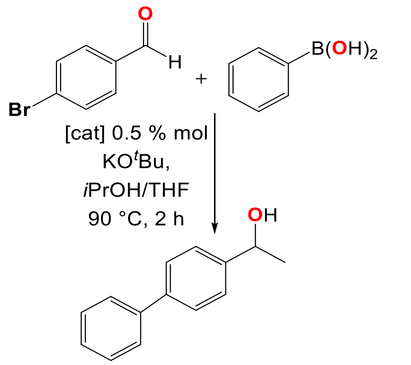	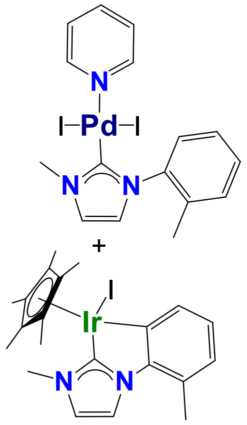 **(39)**	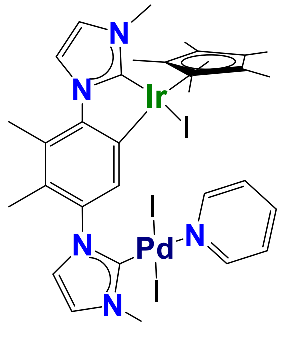 **(85)**	19
2	Hydrodefluorination / transfer hydrogenation of p-fluoroacetophenonea 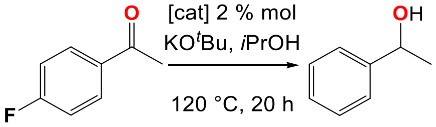	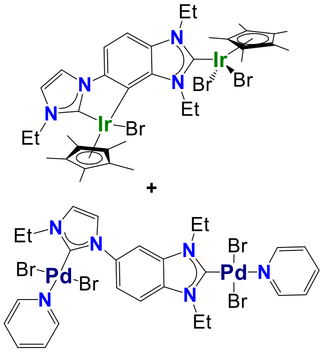 **(80)**	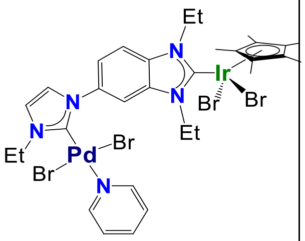 **(92)**	20
3	Condensation of nitrobenzene with benzyl alcohol / imine reduction 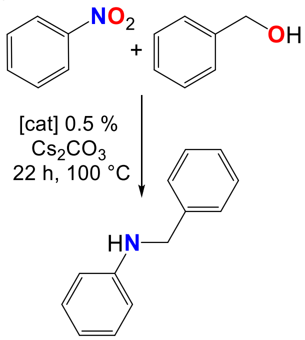	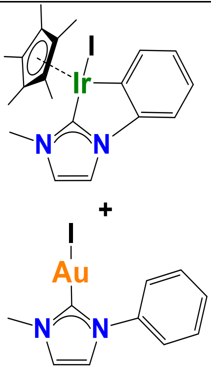 **(8)**	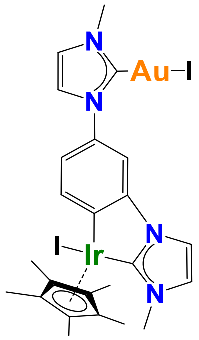 **(82)**	21

4	Condensation of nitrobenzene with benzyl alcohol / imine reduction 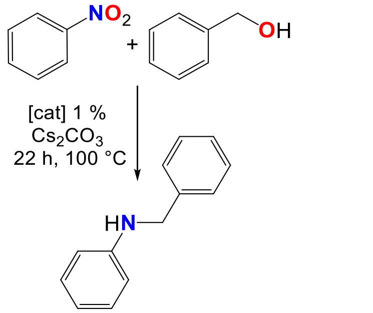	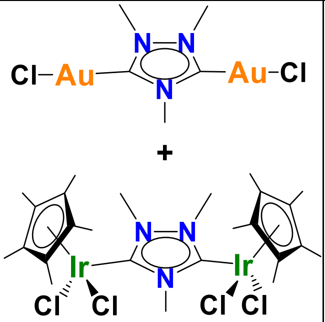 **(81)**	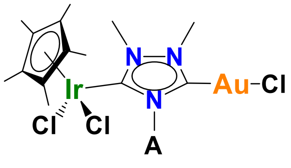 **(96)**	3
5	Cyclization of 2-(ortho-aminophenyl)ethanol / addition of an alkynyl alcohol. 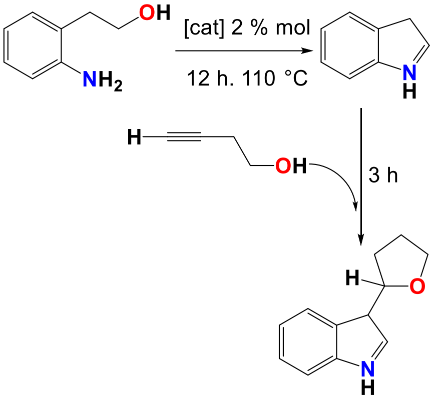	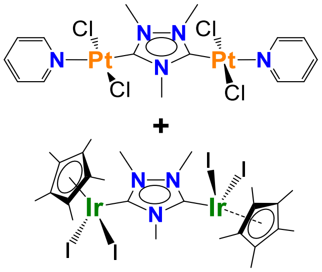 **(16)**	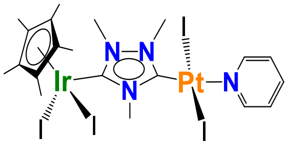 **(60)**	22
6	Dehalogenation / transfer hydrogenation of haloacetophenones 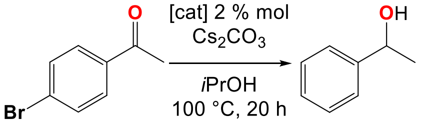	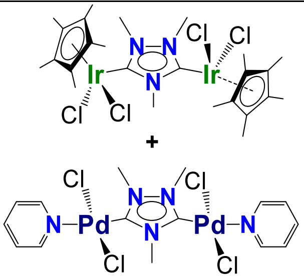 **(25)**	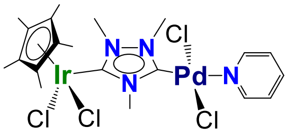 **(>99)**	2
7	Suzuki-Miyaura coupling / transfer hydrogenation 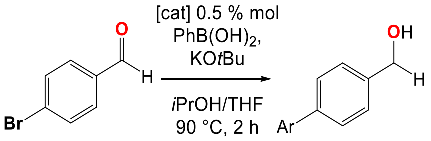	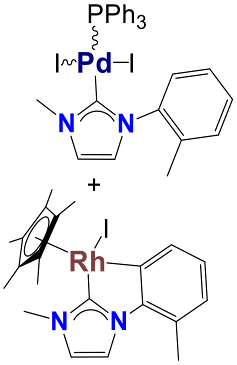 **(7)**	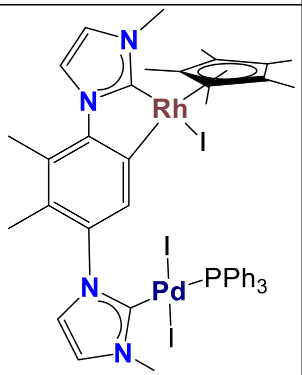 **(44)**	23

8	Hydrodefluorination of fluoroarene[c] 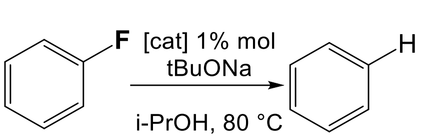	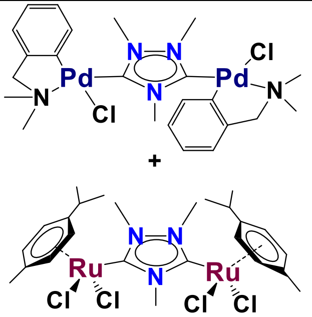 **(49)**	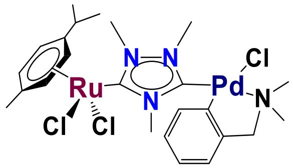 **(100)**	4

[a] According to the original papers, cooperativity has not been measured in any of these compounds.
[b] Metal based catalyst loading.
[c] Not tandem catalysis.

Synthesis and characterization

Caution! The procedures described in this work involve hazardous chemicals and specialized laboratory techniques. Trifluoroacetic acid (TFA) is highly corrosive and can cause severe skin burns and eye damage; it should be handled with extreme care in a well-ventilated fume hood. Diethyl ether and tetrahydrofuran (THF) are highly flammable and prone to the formation of explosive peroxides; these solvents must be tested for peroxides before use and kept away from heat or ignition sources. Dichloromethane (DCM) and acetonitrile are toxic and should be handled with appropriate personal protective equipment (PPE). All reactions using standard Schlenk and high-vacuum techniques involve pressurized or evacuated glassware, which poses a risk of implosion or explosion; users should be properly trained and use safety shielding where appropriate. No unexpected or unusually high safety risks were encountered during the synthesis of the Ru(II) and Ir(III) complexes.

2-methyl-1,3-bis(2-pyridyl) imidazolium hexafluorophosphate [HL^3]PF_6

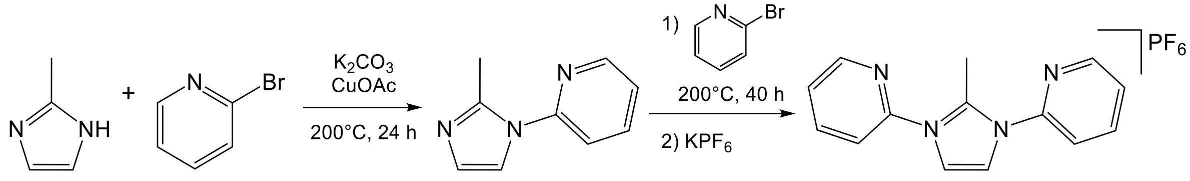

In a pressure tube, 2-methylimidazole (3 g, 36.5 mmol), 2-bromopyridine (8.6 g, 54.8 mmol), K_2CO_3 (5.04 g, 36.5 mmol) and CuOAc (0.45 g, 3.65 mmol) as catalyst, were added. The mixture was heated neat while stirring at 200°C for 24 h. The resulting mixture was dissolved in dichloromethane, filtered and extracted with 100 mL of an aqueous solution of EDTA (2 g, 6.8 mmol) and K_2CO_3.(2 g, 14.4 mmol) The organic layer was dried over $NaSO_4$, filtered and the solution was concentrated under vacuum. Subsequent addition of diethyl ether yielded a brown precipitate, which was filtered and dried, producing the monosubstituted compound **mono-[L^3]** used in the next reaction. Yield: 83 % (4.8 g, 30.3 mmol).

In a pressure tube, **mono-[L^3]** (4.6 g, 28.9 mmol) and 2-bromopyridine (6.8 g, 43.3 mmol) were added. The mixture was heated neat while stirring at 200°C for 40 h. The resulting mixture was dissolved in dichloromethane and precipitated by the addition of diethyl ether. The resulting brown solid was filtered and dissolved in H_2O and treated with an aqueous solution of KPF_6 (7.9 g, 43.3 mmol) yielding a brown precipitate which was filtered and dried under vacuum. Yield: 65 % (7.18 g, 18.78 mmol).

^{1}H NMR (500 MHz, chloroform-*d*): δ (ppm) 8.43 (dd, *J* = 4.4, 2.1 Hz, 1H, H10), 7.88 (td, *J* = 7.8, 1.7 Hz, 1H, H8), 7.72 (d, *J* = 1.2 Hz, 1H, H4/5), 7.57 (dt, *J* = 8.0, 0.9 Hz, 1H, H7), 7.41 – 7.37 (m, 1H, H9), 2.67 (s, 1.5H, H11).

^{13}C NMR (126 MHz, chloroform-*d*): δ (ppm) 149.41 (C10), 146.63 (C6), 144.58 (C2), 139.97 (C8), 125.63 (C9), 121.33 (C4/5), 119.18 (C7), 12.74 (C11).

MS (SQ+) m/z calculated for [$C_{14}H_{13}N_4^+$]: 237, found: 237

Elemental analysis (%) calculated: C: 43.99, N: 14.66, H: 3.43; found: C: 44.15, N: 15.66, H: 2.93.

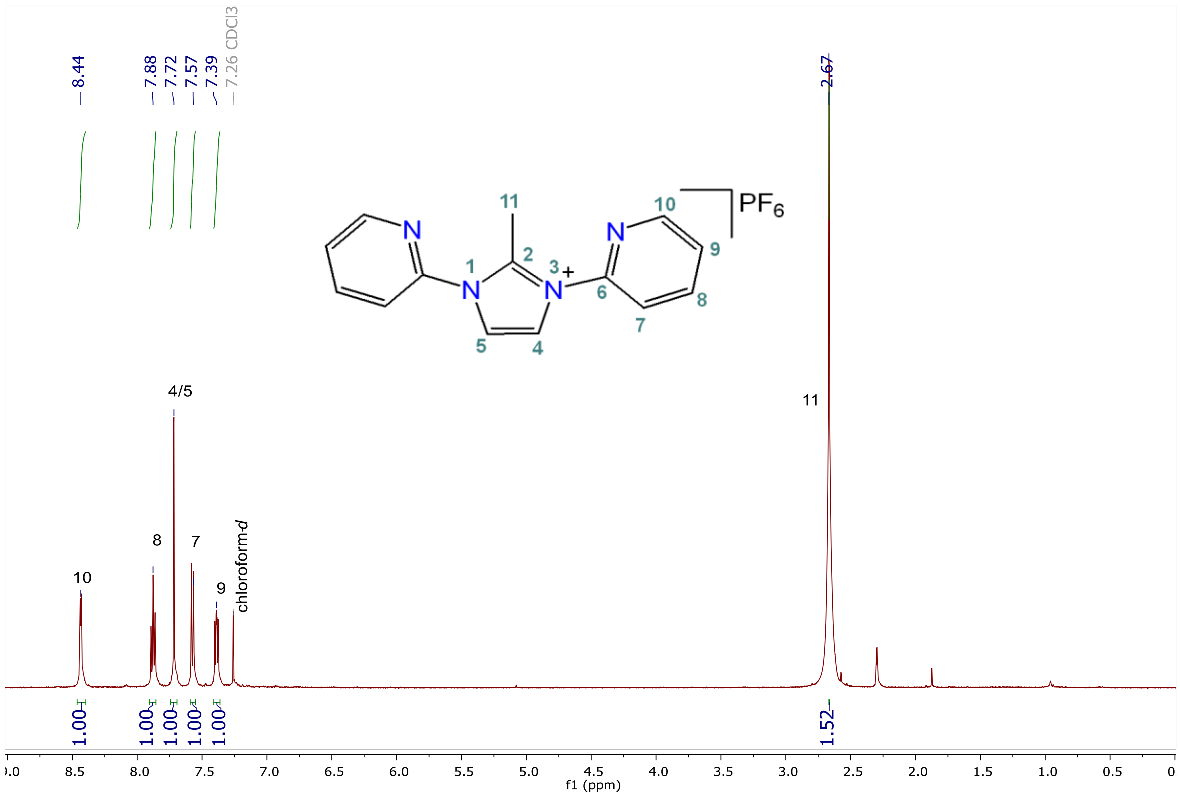

Figure S2 ^{1}H NMR spectrum for **[HL^{3}]PF$_6$** in chloroform-*d*/DMSO-d_6

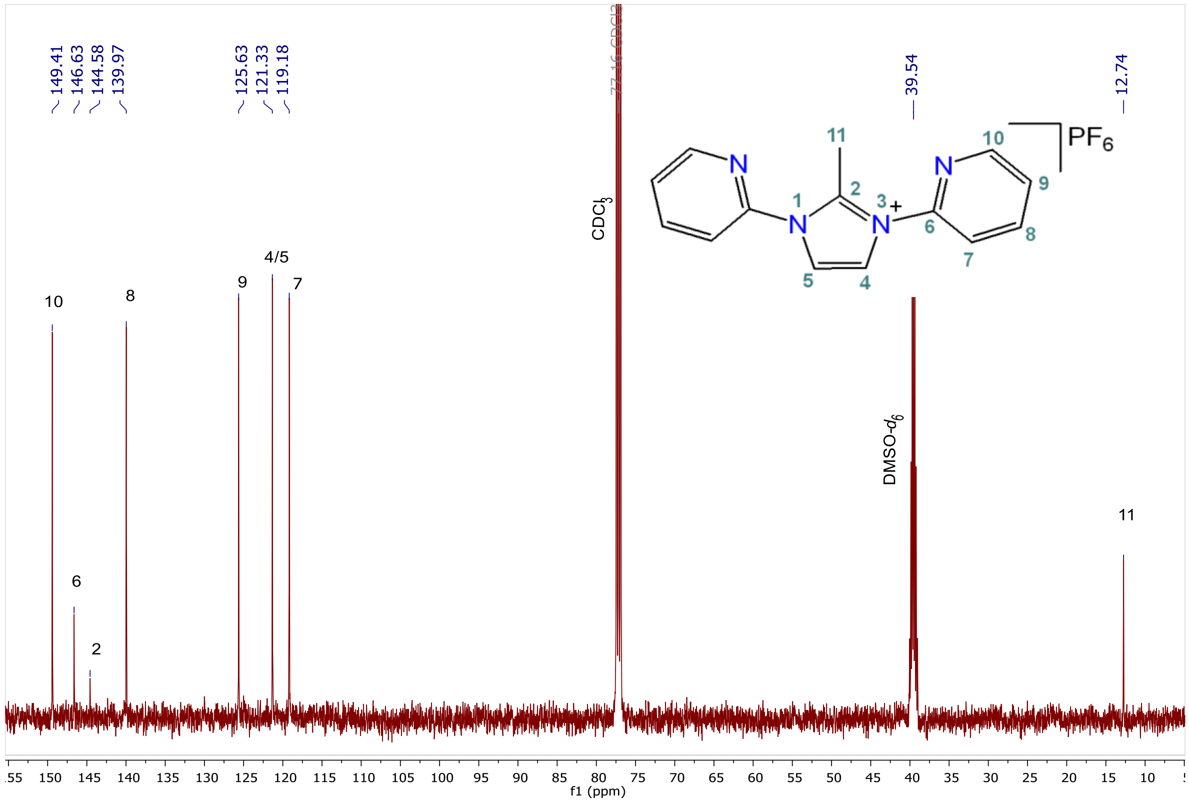

Figure S3 ^{13}C NMR spectrum for **[HL^{3}]PF$_6$** in chloroform-*d*/DMSO-d_6

2-phenyl-1,3-bis(2-pyridyl) imidazolium hexafluorophosphate [HL[4]]PF_6

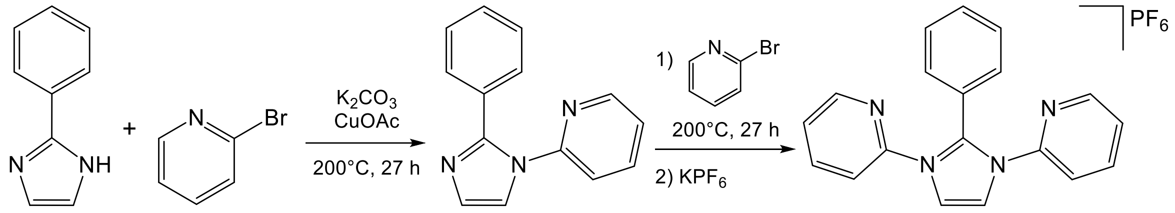

mono-[L[4]] **[L[4]]**

In a pressure tube, 2-phenylimidazole (2 g, 13.9 mmol), 2-bromopyridine (3.2 g, 20.8 mmol), K_2CO_3 (1.9 g, 13.9 mmol) and CuOAc (0.17 g, 1.39 mmol) as catalyst, were added. The mixture was heated neat while stirring at 200°C for 27 h. The resulting mixture was dissolved in dichloromethane, filtered and extracted with 100 mL of an aqueous solution of EDTA (2 g, 6.8 mmol) and K_2CO_3 (2 g, 14.4 mmol). The organic layer was dried over $NaSO_4$, filtered and the solution was concentrated under vacuum. Subsequent addition of diethyl ether yielded a brown precipitate which was filtered and dried, producing the monosubstituted compound **mono-[L[4]]**, used in the next reaction. Yield: 77 % (2.4 g, 10.7 mmol).

In a pressure tube, **mono-[L[4]]** (2 g, 9.04 mmol) and 2-bromopyridine (2.14 g, 13.5 mmol) were added. The mixture was heated neat while stirring at 200°C for 27 h. The resulting mixture was dissolved in dichloromethane and precipitated by the addition of diethyl ether. The resulting brown solid was filtered, dissolved in H_2O and treated with an aqueous solution of KPF_6 (2.5 g, 13.5 mmol) yielding a brown precipitated which was filtered and dried under vacuum. Yield: 85 % (3.41 g, 7.7 mmol).

^{1}H NMR (500 MHz, chloroform-*d*): δ (ppm) 8.34 (dd, *J* = 4.8, 2.2 Hz, 1H, H10), 7.89 (d, *J* = 1.2 Hz, 1H, H4/5), 7.64 (tt, *J* = 7.9, 2.0 Hz, 1H, H8), 7.37 – 7.27 (m, 1.5H, H13, H14), 7.16 (td, *J* = 7.9, 2.0 Hz, 1H, H12), 7.15 – 6.97 (m, 2H, H7, H9).

^{13}C{^{1}H} RMN (126 MHz, chloroform-*d*) δ (ppm) 149.43 (C10), 146.47 (C6), 143.80 (C2), 139.28 (C8), 132.36 (C14), 130.19 (C9), 129.00 (C7), 125.58 (C13), 122.43 (C4/5), 120.45 (C11), 119.92 (C12).

MS (SQ+) m/z calculated for [$C_{19}H_{15}N_4^+$]: 299, found: 299.

Elemental analysis (%) calculated: C: 60.17, N: 14.77, H: 3.99; found: C: 59.52, N: 16.73, H: 3.66.

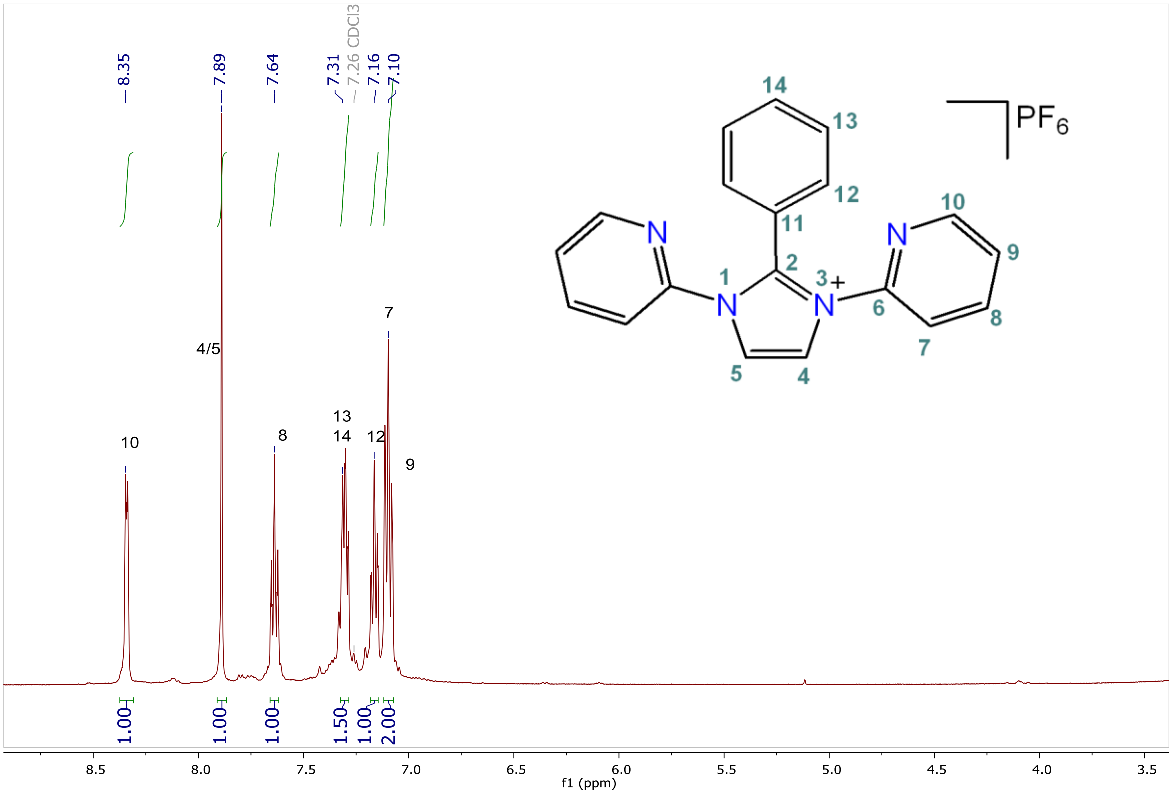

Figure S4 ^{1}H NMR spectrum for **[HL^{4}]PF$_6$** in chloroform-*d*/DMSO-*d*$_6$

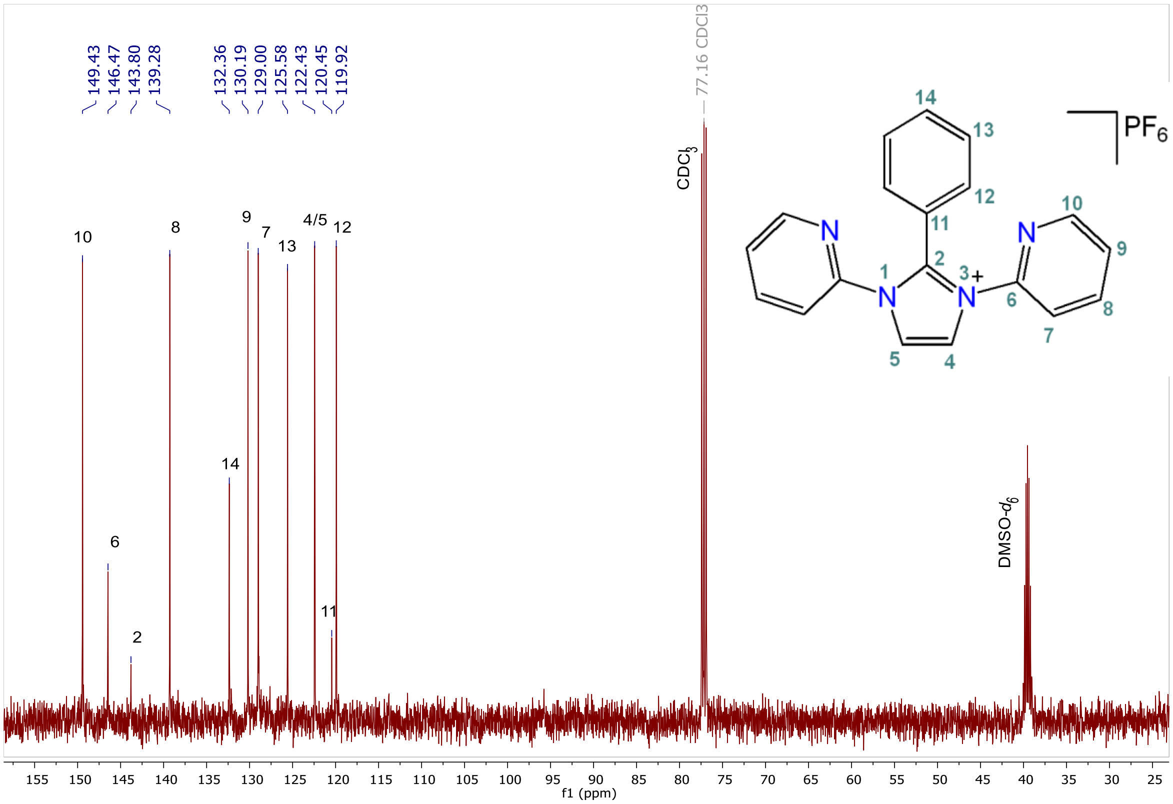

Figure S5 ^{13}C NMR spectrum for **[HL^{4}]PF$_6$** in chloroform-*d*/DMSO-*d*$_6$

^{1}H and ^{13}C NMR spectra of complexes

[Ir-L^{2}]PF$_6$

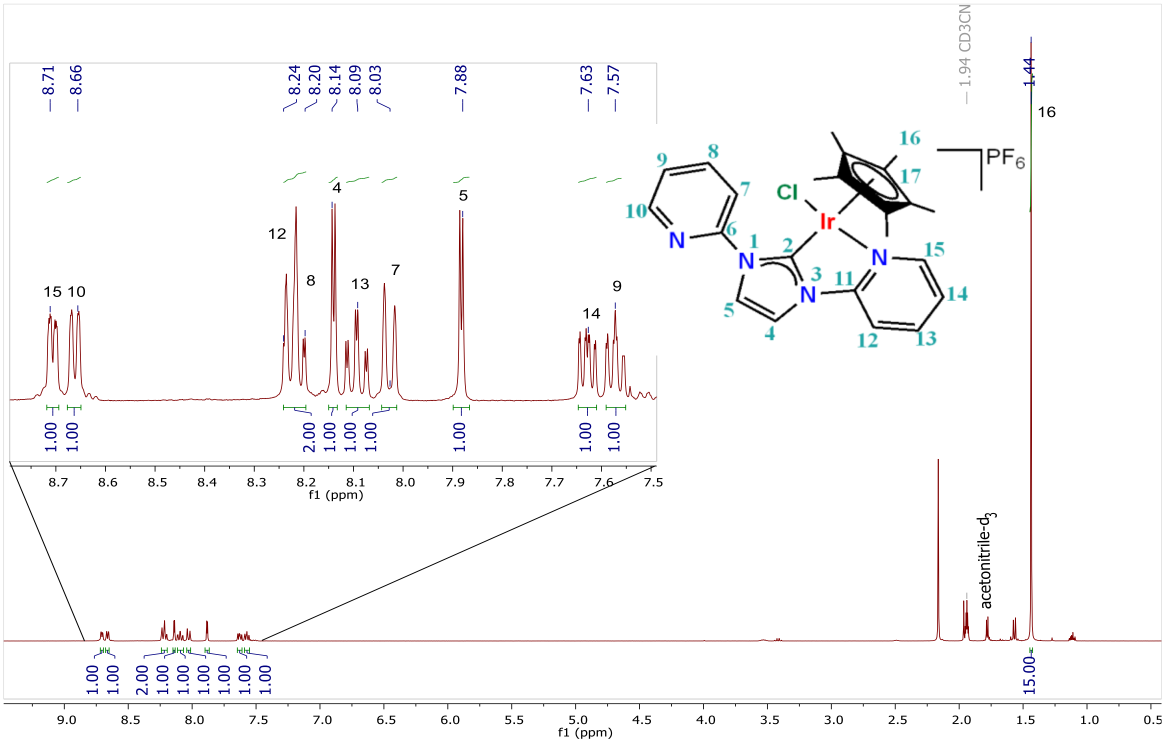

Figure S6 ^{1}H NMR spectrum for **[Ir-L^{2}]PF$_6$** in acetonitrile-d_3

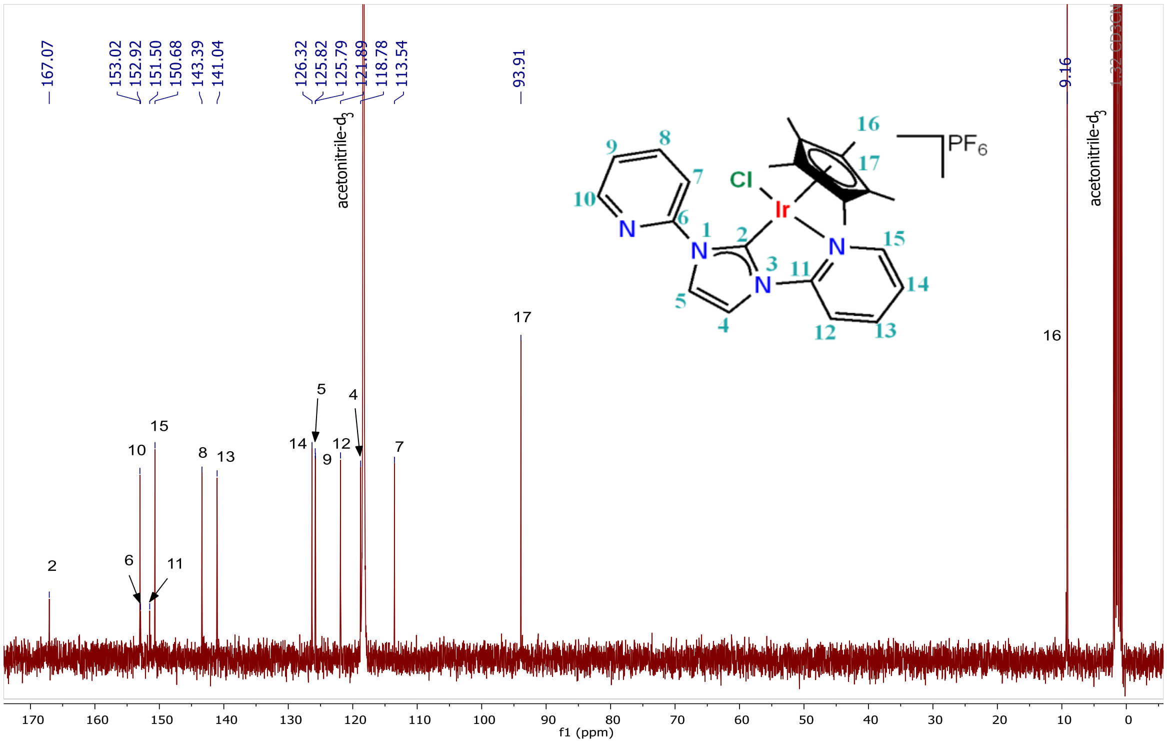

Figure S7 ^{13}C NMR spectrum for **[Ir-L^{2}]PF$_6$** in acetonitrile-d_3

[Ru-L^2]PF$_6$

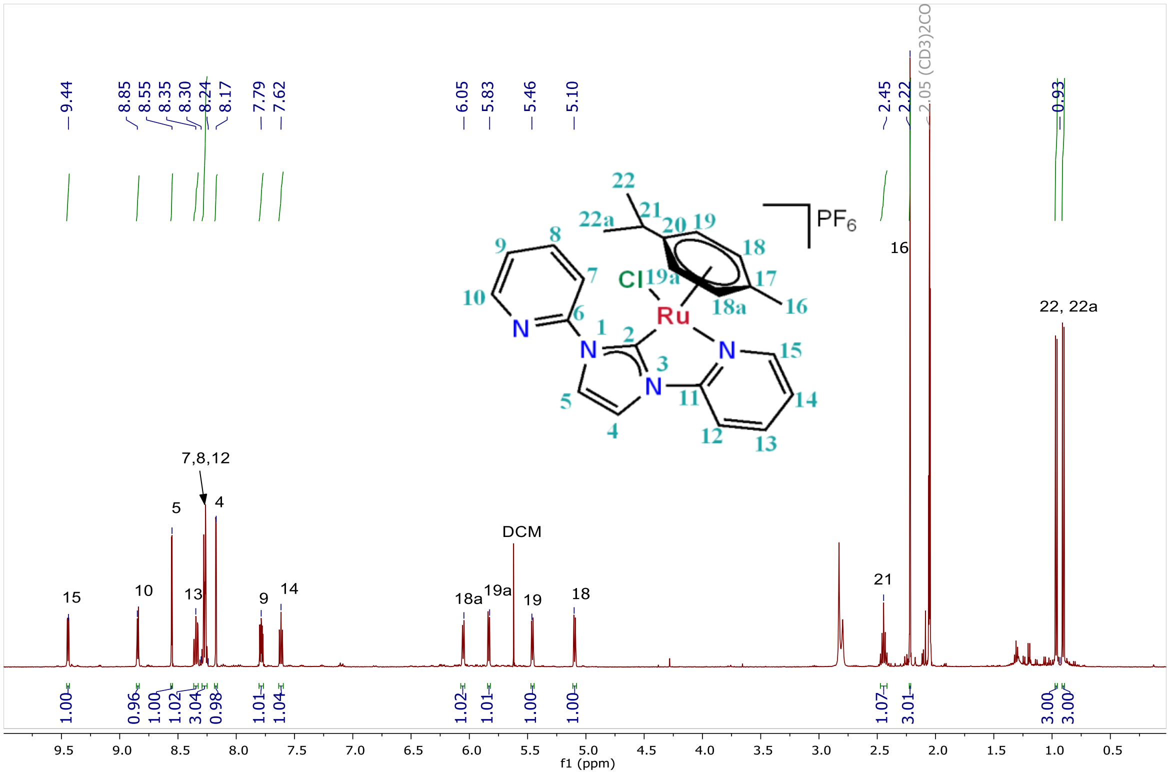

Figure S8 ^{1}H NMR spectrum for **[Ru-L^2]PF$_6$** in acetone-d_6

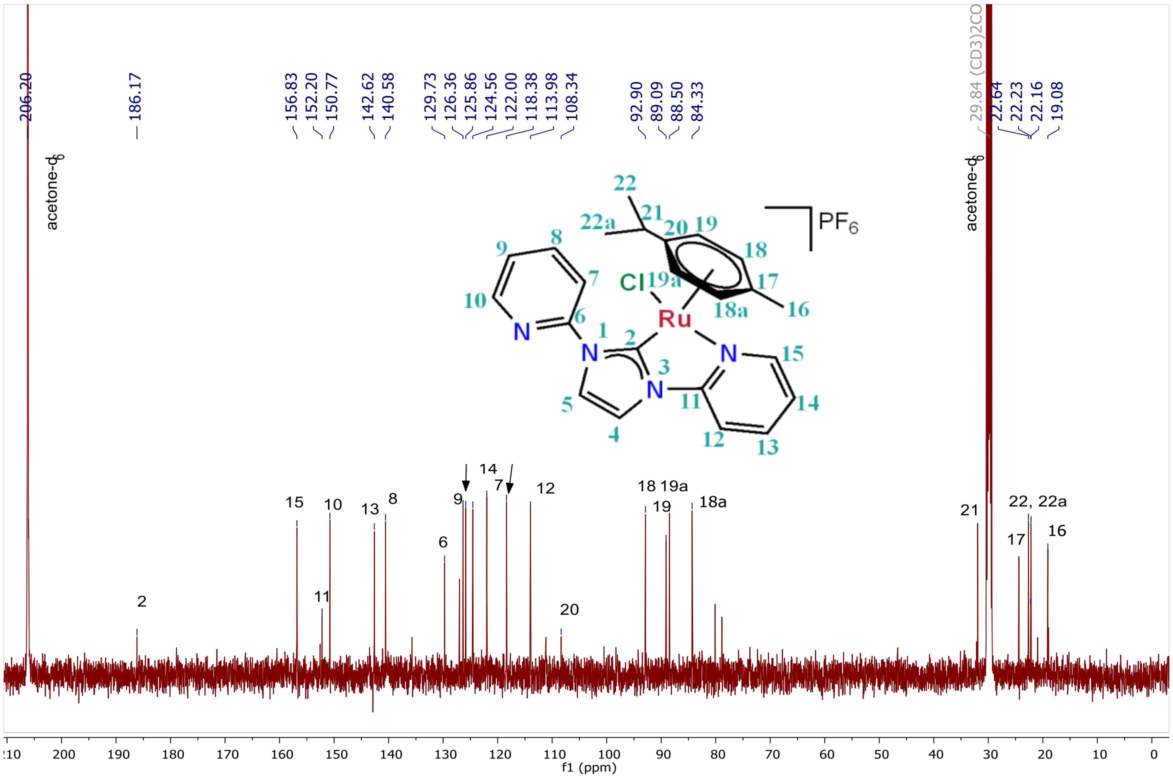

Figure S9 ^{13}C NMR spectrum for **[Ru-L^2]PF$_6$** in acetone-d_6

$[Ru\text{-}L^2]B(Ph)_4$

A solution of $NaB(Ph)_4$ (0.45 g, 1.3 mmol) in 20 mL of H_2O was added to an aqueous solution of **$[HL^2]Br$** (0.4 g, 1.3 mmol) yielding a white precipitate, which was filtered and dried under vacuum to afford **$[HL^2]B(Ph)_4$** as a white solid. Yield: 77 % (0.55 g, 1.01 mmol).

A mixture of **$[HL^2]B(Ph)_4$** (0.1 g, 0.18 mmol), Ag_2O (0.03 g, 0.11 mmol), $N(CH_3)_4Cl$ (0.03 g, 0.28 mmol) and $[(p\text{-}cymene)RuCl_2]_2$ (0.067 g, 0.11 mmol) was suspended in a solution of anhydrous dichloromethane (40 mL) and stirred at room temperature in the dark for 3 h. The resulting mixture was filtered through Celite, and the solvent was removed under vacuum to afford a wine-red solid. Yield: 96 % (0.15 g, 0.18 mmol). Crystals suitable for X-ray crystallography were obtained by slow vapor diffusion of diethyl ether into a solution of **$[Ru\text{-}L^2]B(Ph)_4$** in acetone.

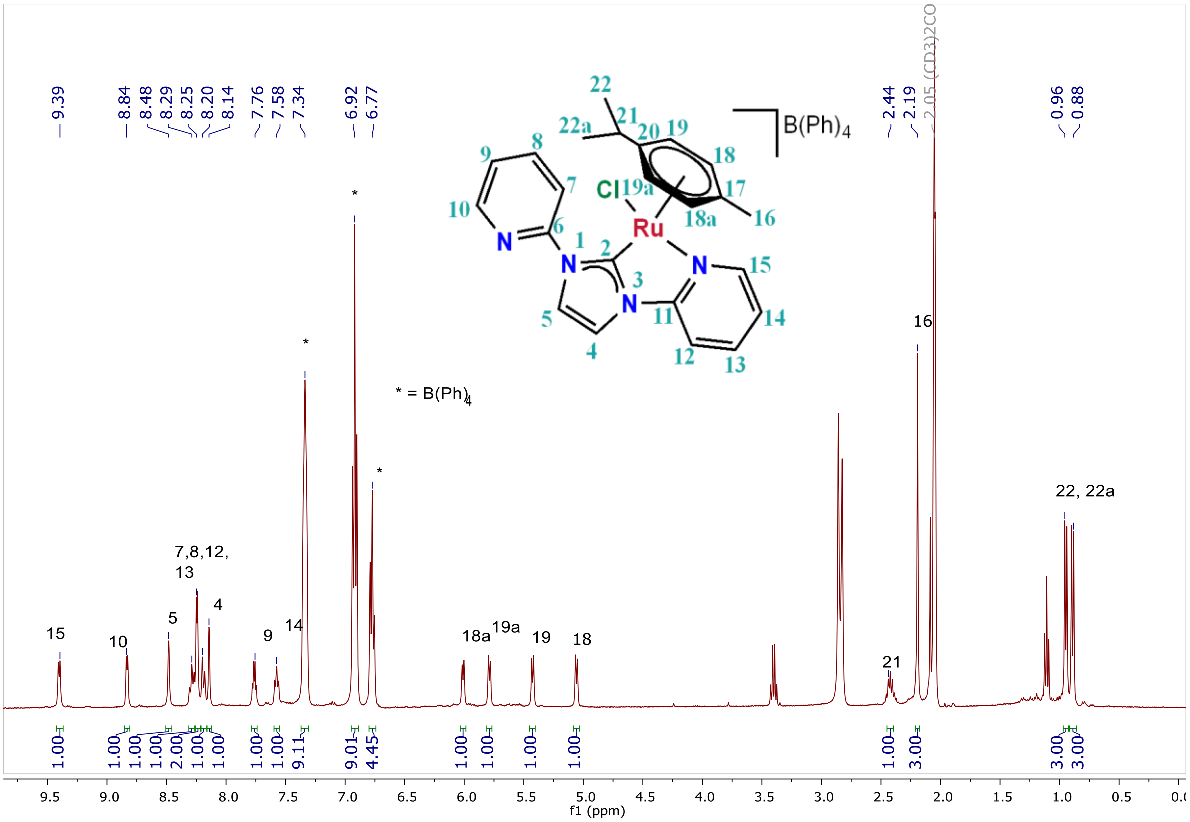

Figure S10 ^{1}H NMR spectrum for **$[Ru\text{-}L^2]B(Ph)_4$** in acetone-d_6

[Ir-L³]PF₆

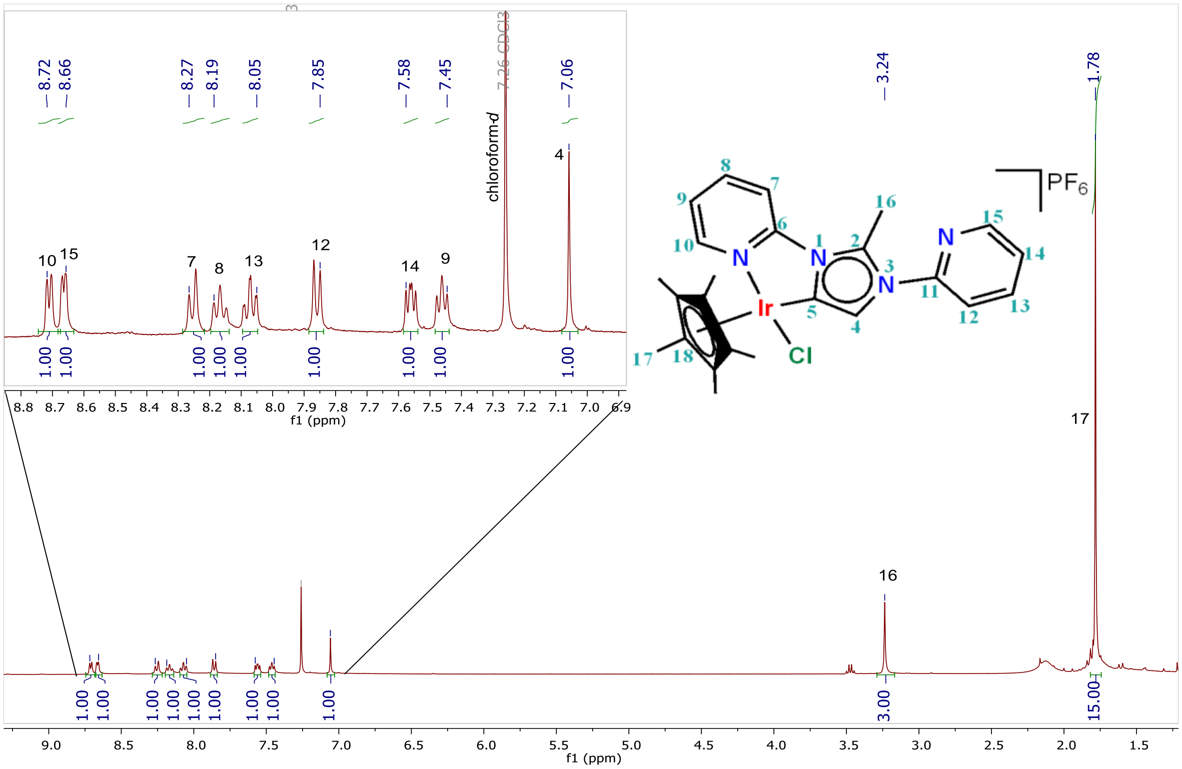

Figure S11 ^{1}H NMR spectrum for **[Ir-L³]PF₆** in chloroform-*d*

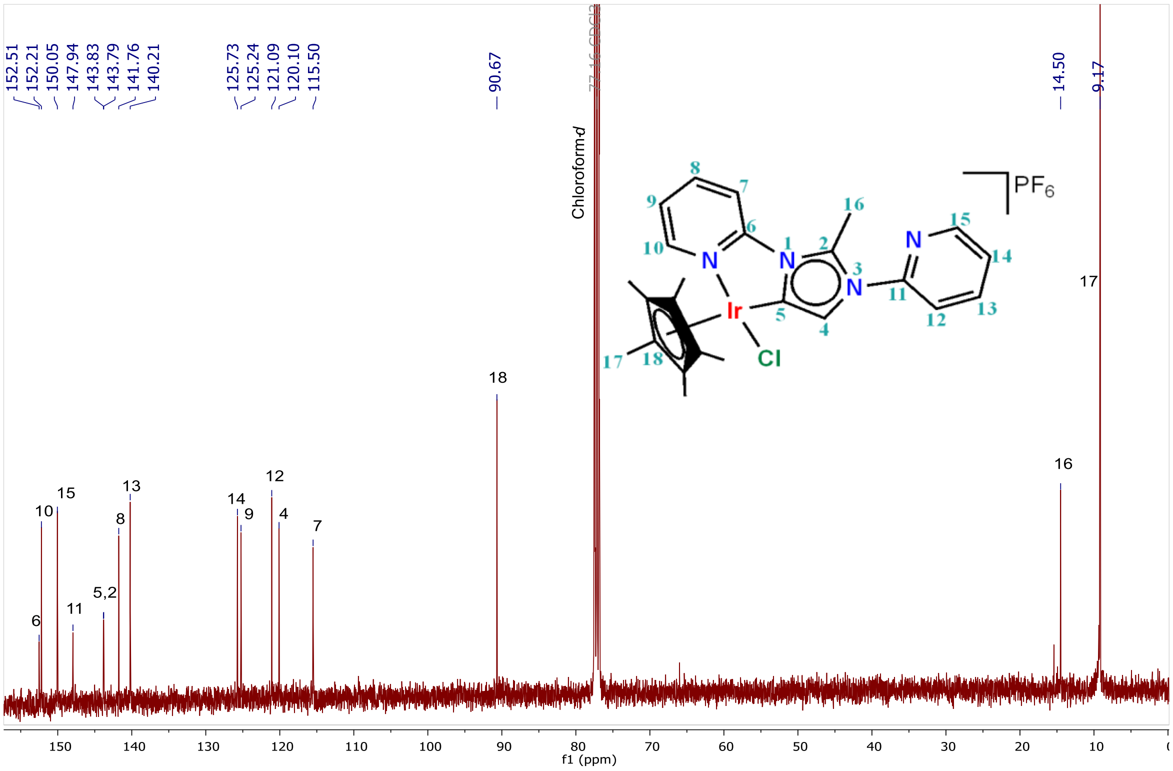

Figure S12 ^{13}C NMR spectrum for **[Ir-L³]PF₆** in chloroform-*d*

[Ir-L⁴]PF₆

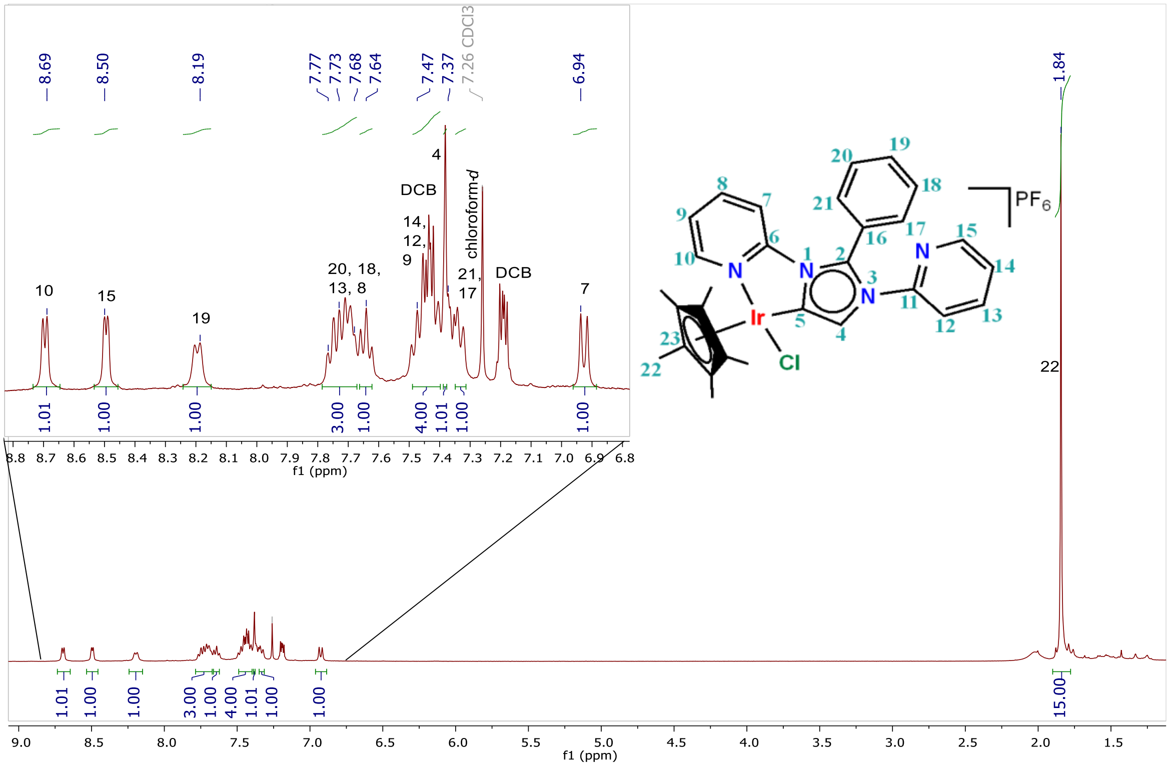

Figure S13 ^{1}H NMR spectrum for **[Ir-L^{4}]PF$_{6}$** in chloroform-*d*

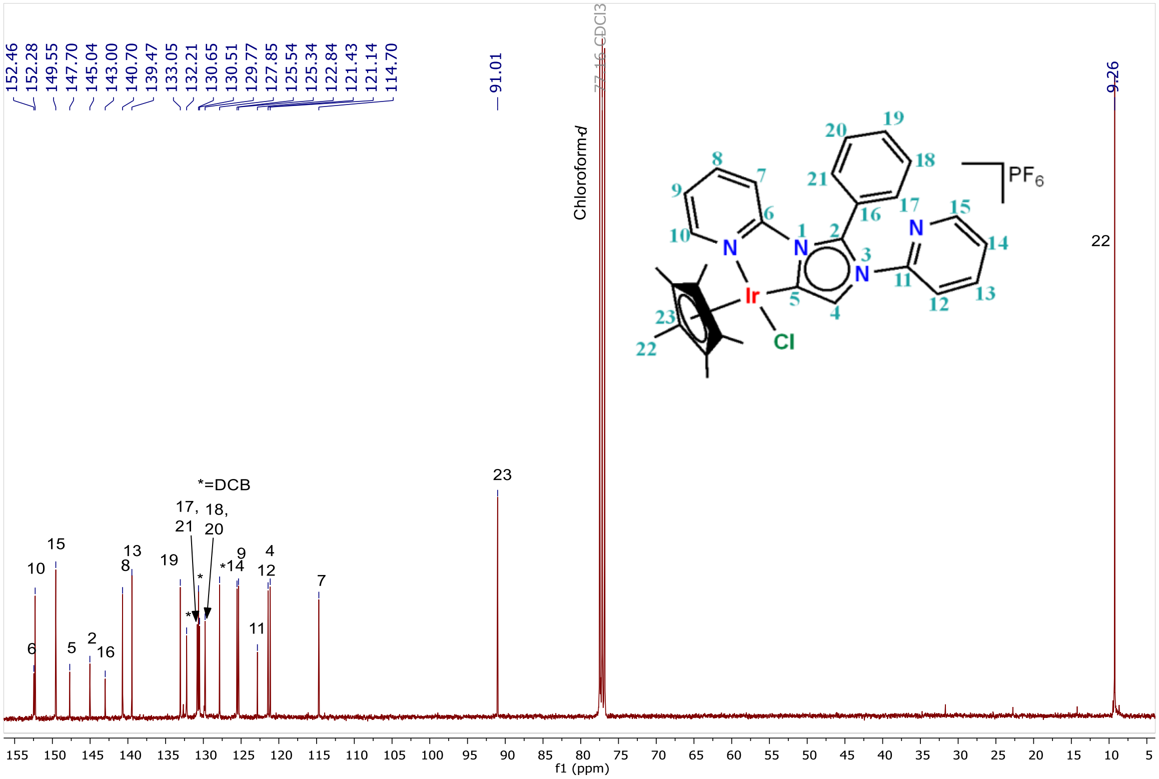

Figure S14 ^{13}C NMR spectrum for **[Ir-L^{4}]PF$_{6}$** in chloroform-*d*

[Ir-L²-Ir]PF₆

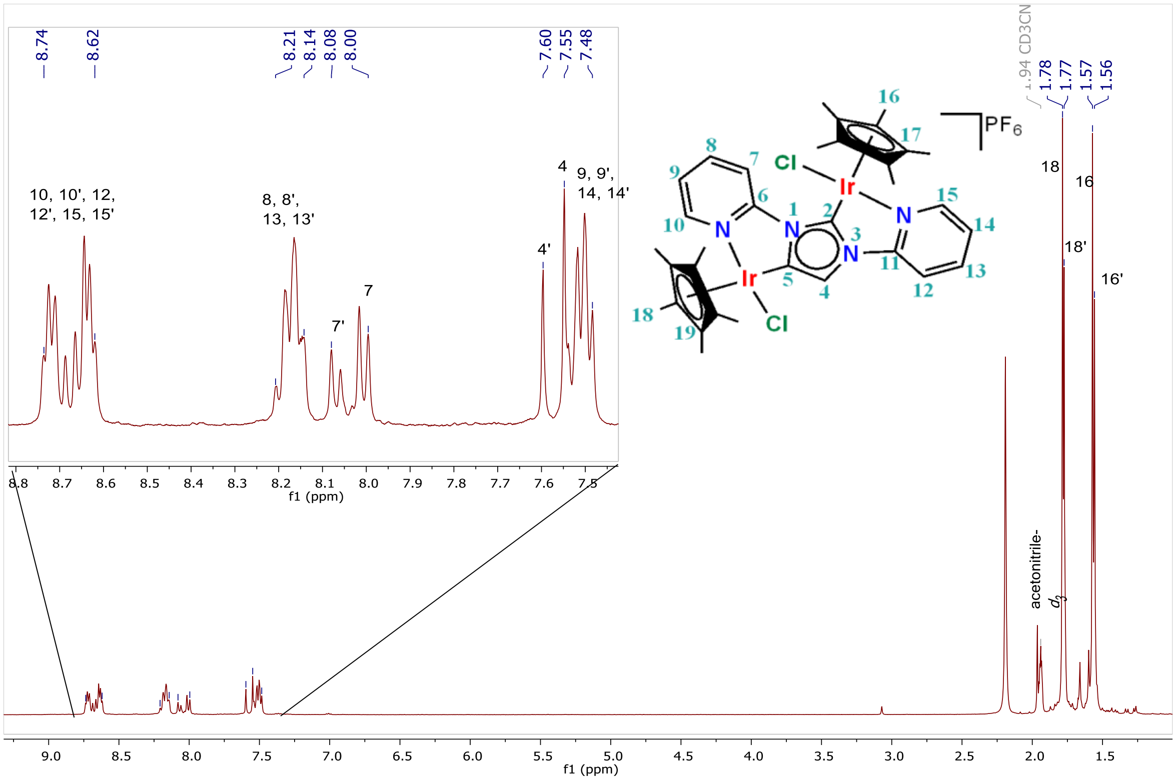

Figure S15 ^{1}H NMR spectrum for **[Ir-L^{2}-Ir]PF$_6$** in acetonitrile-*d*$_3$

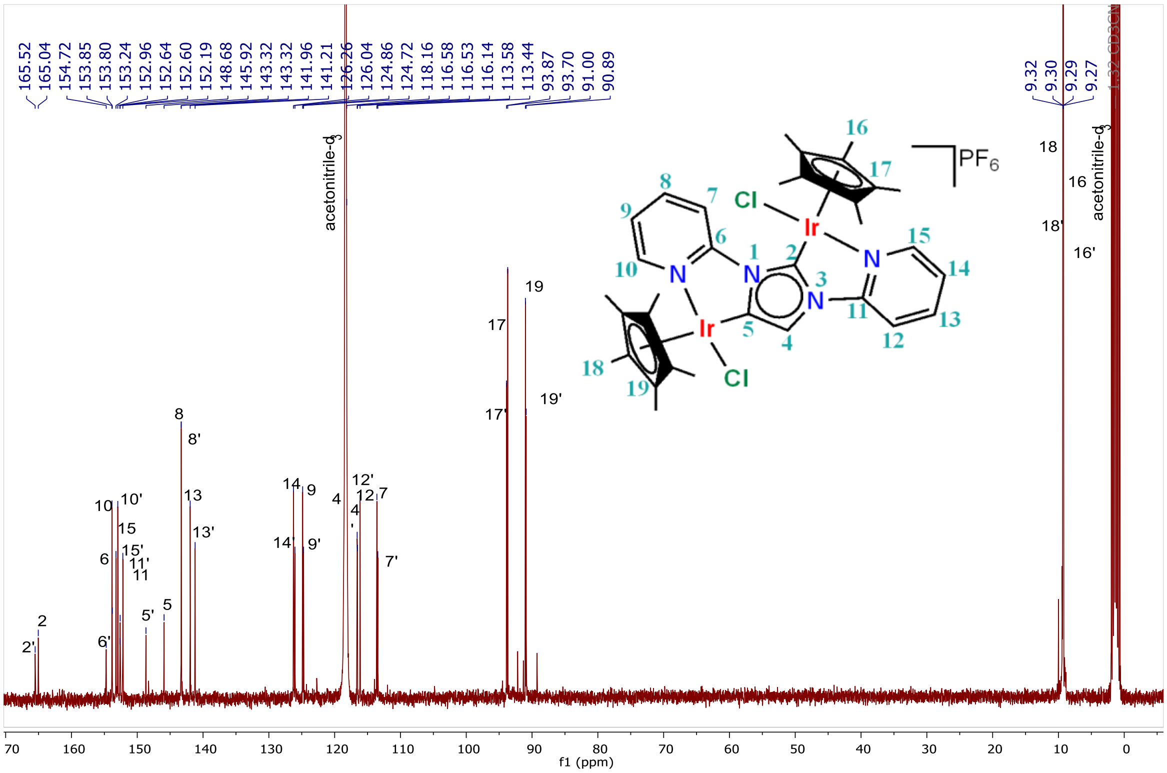

Figure S16 ^{13}C NMR spectrum for **[Ir-L^{2}-Ir]PF$_6$** in acetonitrile-*d*$_3$

[Ir-L^2-Ru]PF$_6$

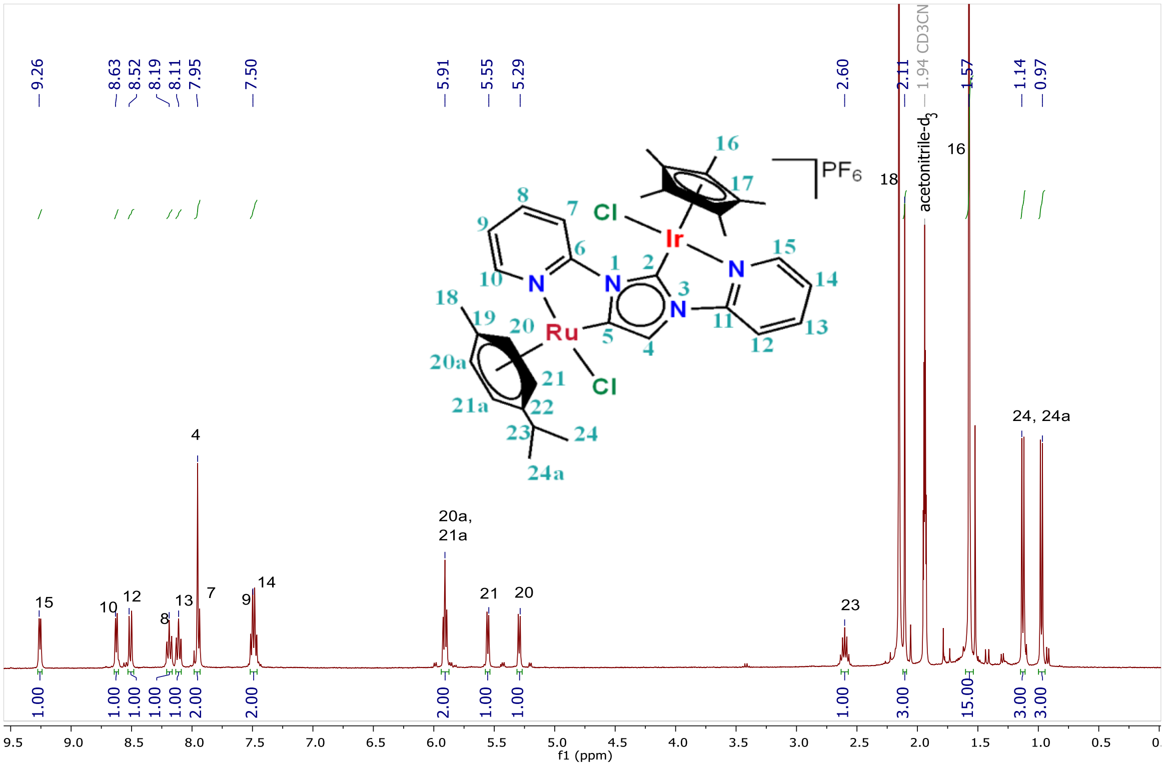

Figure S17 ^{1}H NMR spectrum for **[Ir-L^2-Ru]PF$_6$** (**Isomer A**) in acetonitrile-d_3

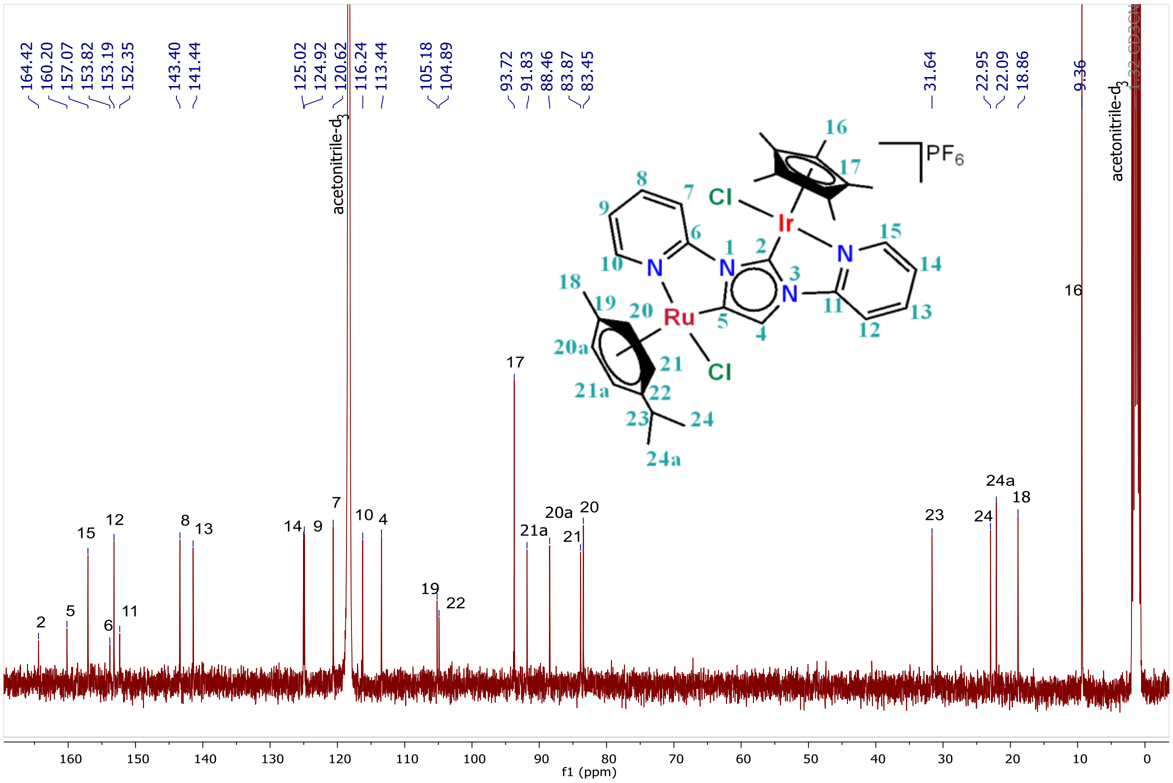

Figure 18 ^{13}C NMR spectrum for **[Ir-L^2-Ru]PF$_6$** (**Isomer A**) in acetonitrile-d_3

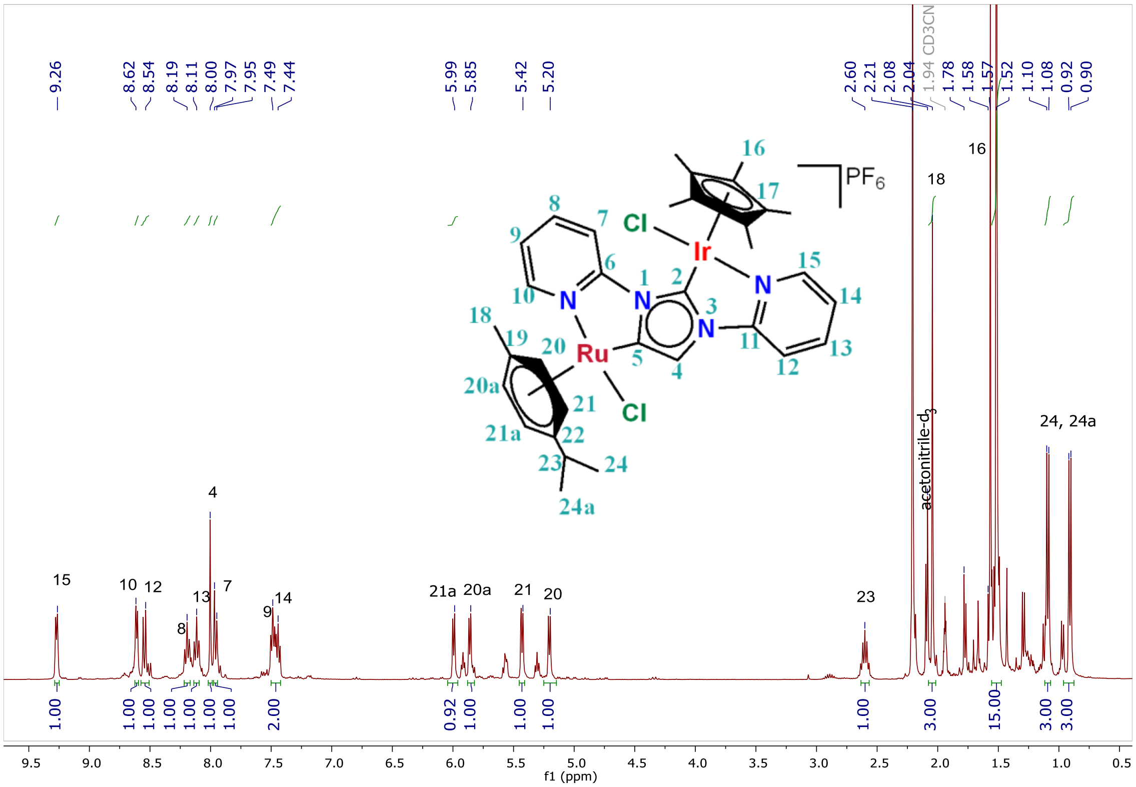

Figure S19 ^{1}H NMR spectrum for **[Ir-L^{2}-Ru]PF$_6$** (**Isomer B**) in acetonitrile-d_3

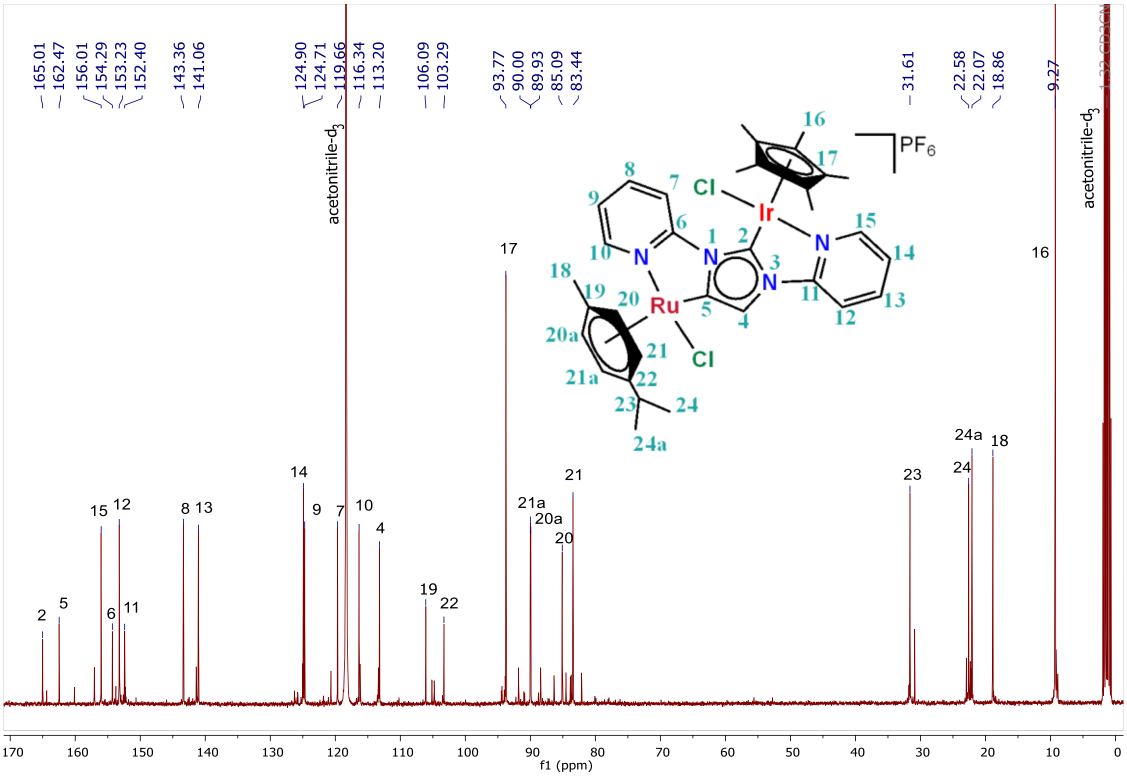

Figure S20 ^{13}C NMR spectrum for **[Ir-L^{2}-Ru]PF$_6$** (**Isomer B**) in acetonitrile-d_3

[Ru-L^2-Ir]PF$_6$

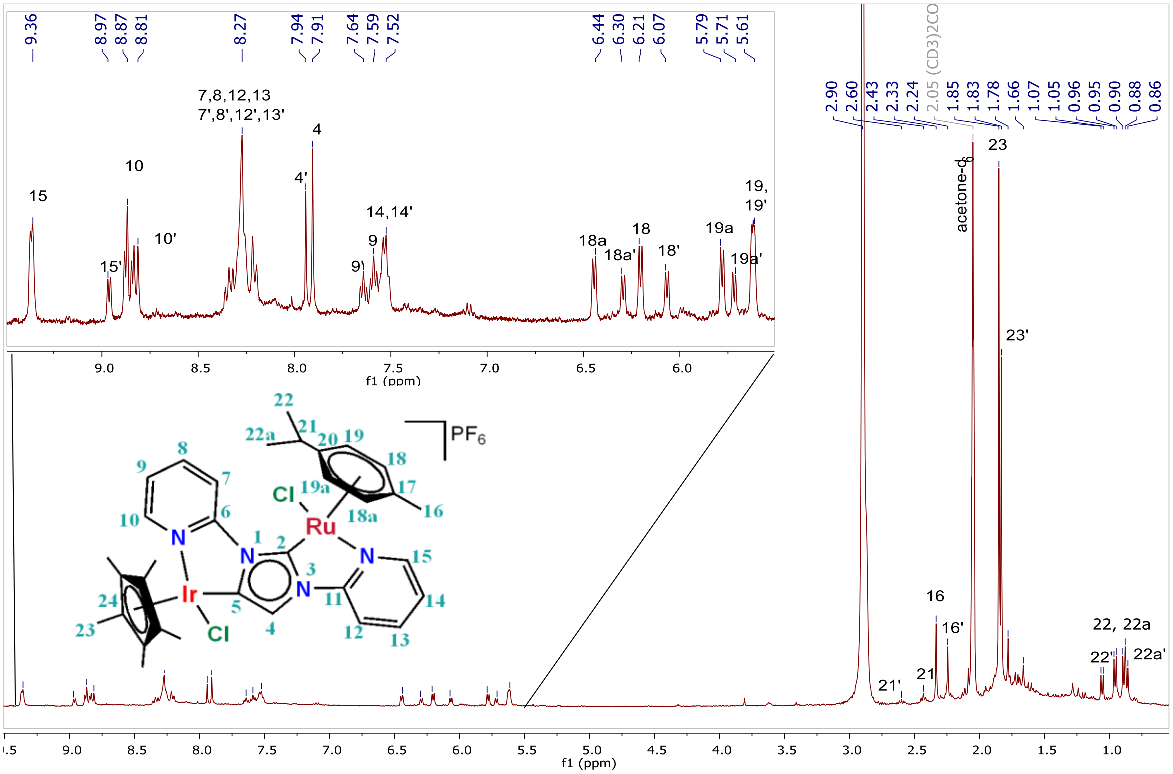

Figure S21 ^{1}H NMR spectrum for **[Ru-L^2-Ir]PF$_6$** in acetone-*d$_6$*

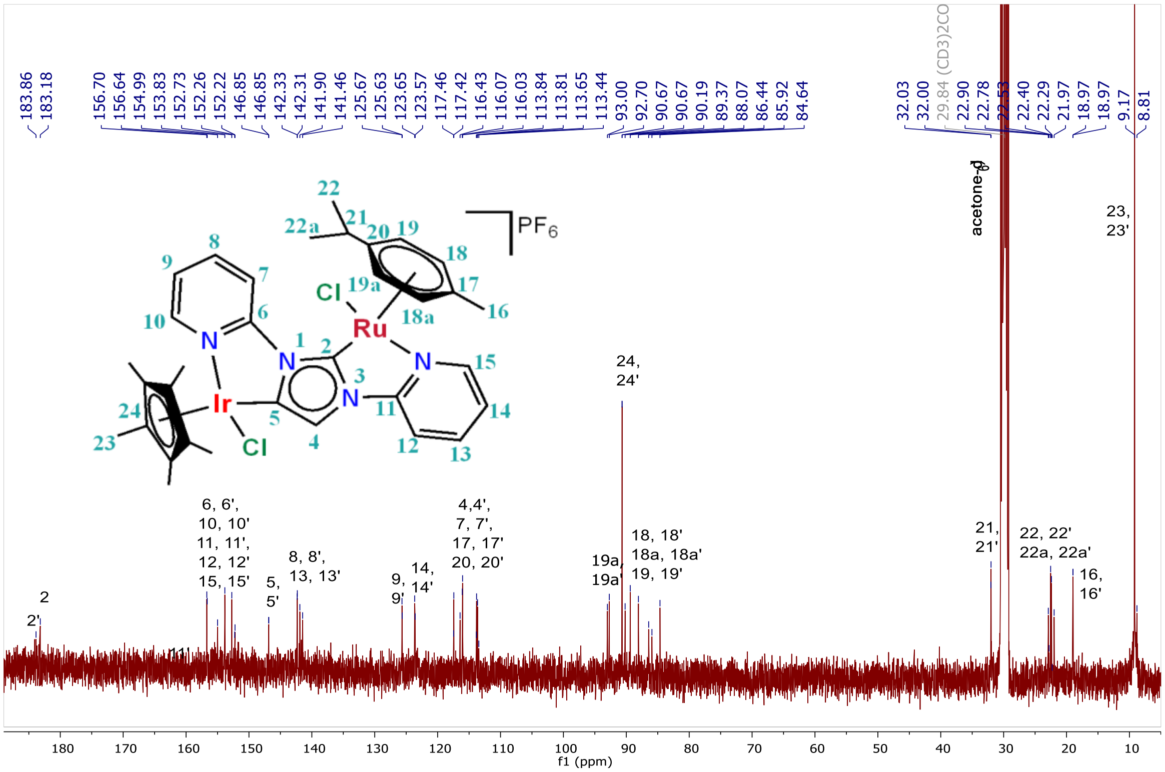

Figure 22 ^{13}C NMR spectrum for **[Ru-L^2-Ir]PF$_6$** in acetone-*d$_6$*

Synthesis of deuterated substrates

Benzyl alcohol-*d*$_7$ ($C_6D_5CD_2OH$)[24–26]

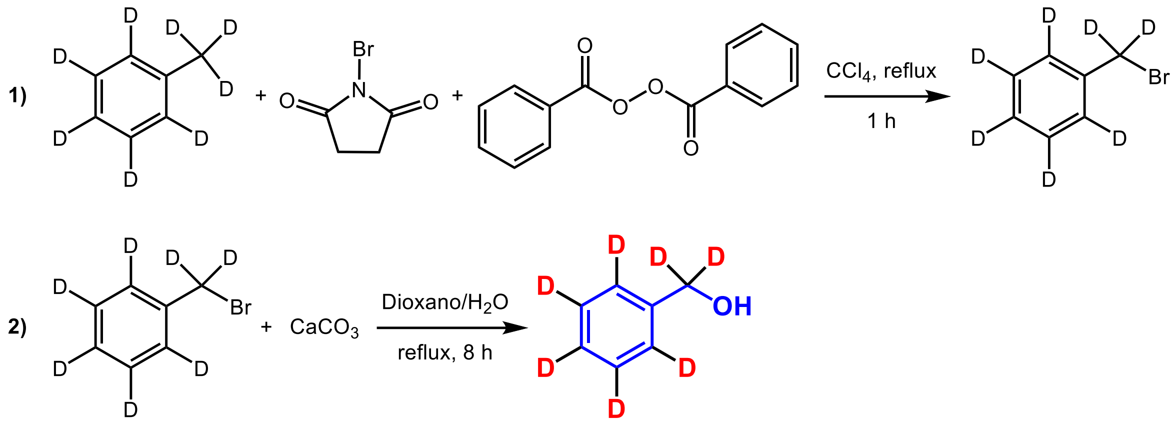

$C_6D_5CD_2OH$

Toluene-*d*$_8$ (4 g, 40 mmol) and *N*-bromosuccinimide (7.1 g, 40 mmol) were added to a flask and dissolved in CCl_4 (100 mL), then benzoyl peroxide (0.12 g, 0.5 mmol) was added to the solution and refluxed for 1 h. The solution was cooled to room temperature and filtered, the solvent was removed under vacuum, the benzyl bromide-*d*$_7$ was obtained as a colorless liquid and used in the next step without any purification.

A solution of $CaCO_3$ (20 g, 200 mmol) in water (100 mL) was added to a solution of benzyl bromide-*d*$_7$ (7 g, 40 mmol) in dioxane (100 mL) and refluxed for 8 h. The solution was cooled to room temperature and dioxane was removed under vacuum, a white precipitate was observed, then CH_2Cl_2 (100 mL) was added. A solution of HCl was gradually added until all solids had dissolved, then the phases were separated, and the aqueous layer was extracted five times with CH_2Cl_2. The organic layer was washed with a $NaHCO_3$ solution, dried over $NaSO_4$ and filtered. The solvent was removed under vacuum resulting in yellow oil and purified by Kugelrohr distillation to give a colorless oil, identified as benzyl alcohol-*d*$_7$ (2.6 g, 22.4 mmol, 56% overall yield).

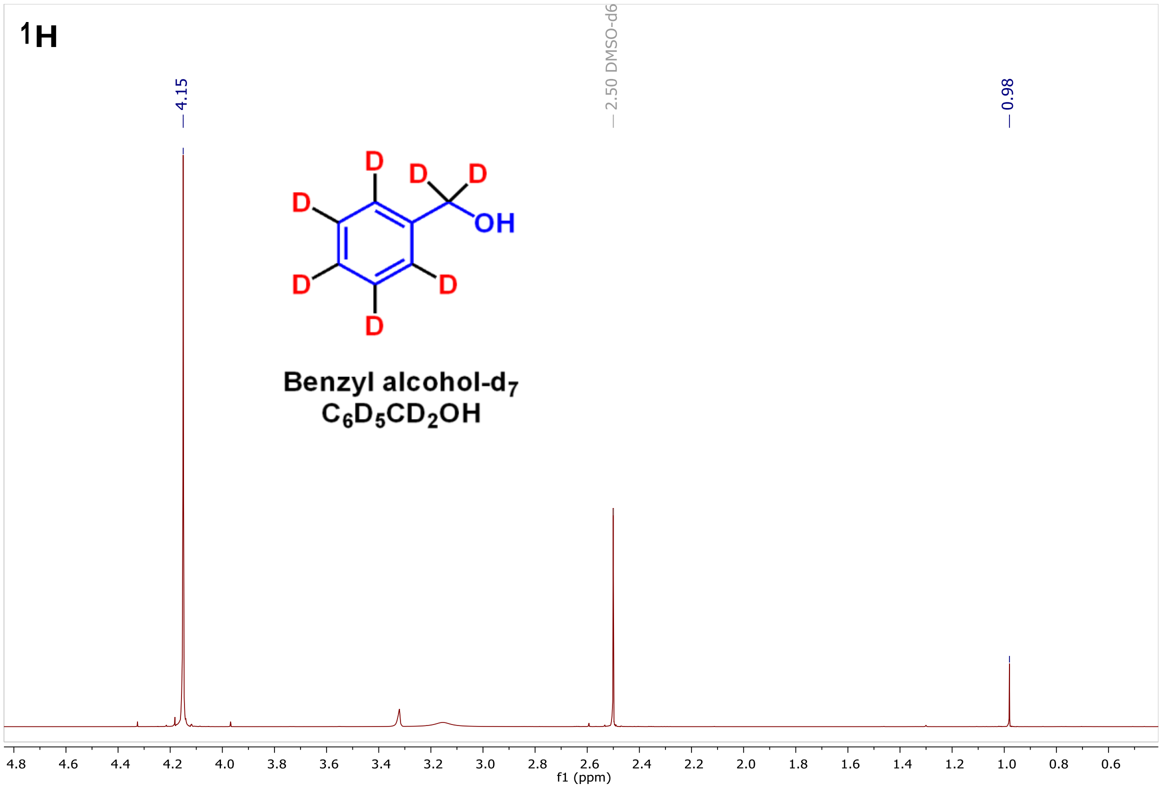

Figure S23 ^{1}H NMR spectrum for **benzyl alcohol-*d*$_7$ ($C_6D_5CD_2OH$)** in DMSO-*d*$_6$

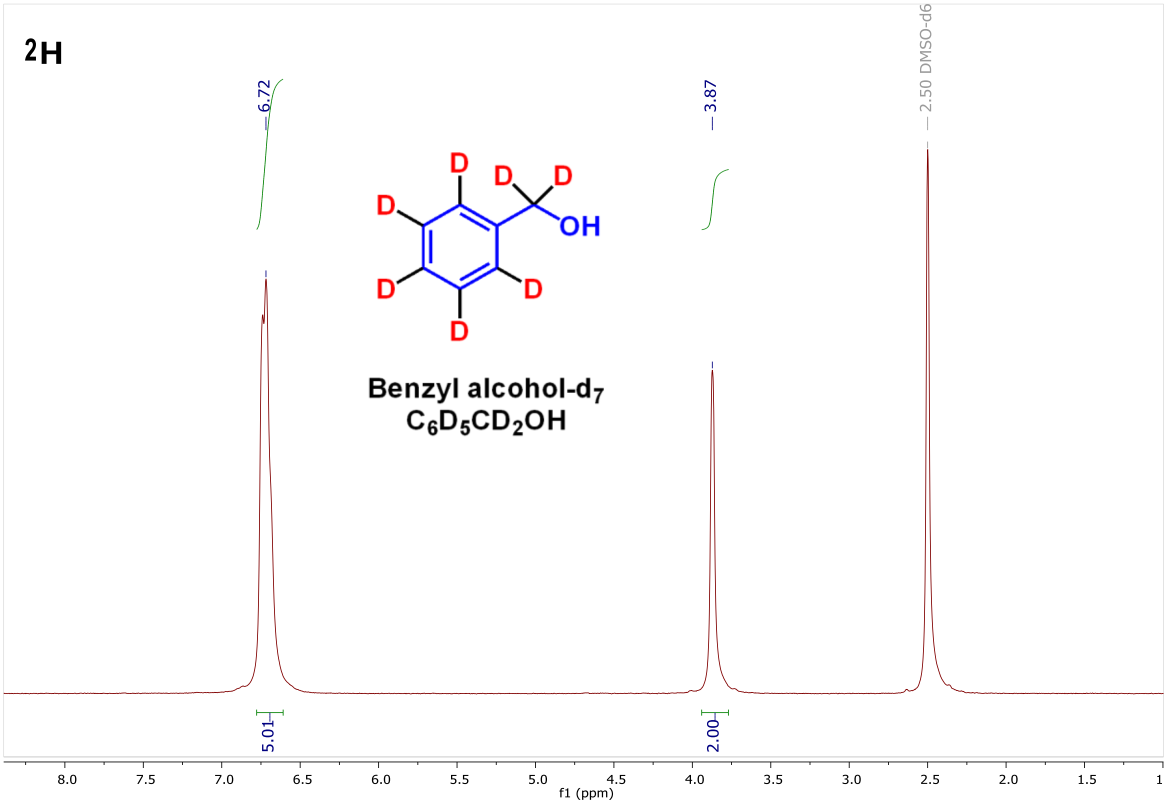

Figure S24 ^{2}H NMR spectrum for **benzyl alcohol-*d*$_7$ ($C_6D_5CD_2OH$)** in DMSO-*d*$_6$

Benzyl alcohol-*d₁* (C_6H_5CHDOH)[27]

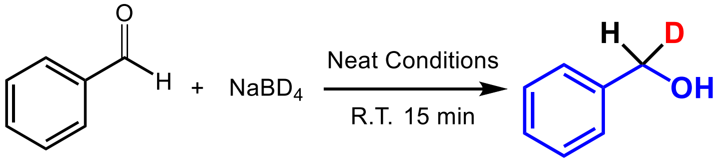

Benzaldehyde (1.06 g, 10 mmol) was added to a flask, cooled to 0°C in an ice bath and $NaBD_4$ (0.5 g, 12 mmol) was gradually added, the mixture was stirred for 15 minutes at room temperature before it was cooled at 0°C (ice bath) and the mixture was quenched with water (20 mL) and extracted with CH_2Cl_2 (20 mL). The phases were separated, and the aqueous layer was extracted three times with CH_2Cl_2. The organic layer was dried over $NaSO_4$ and filtered. The solvent was removed under vacuum, resulting in a pale-yellow oil which was submitted to Kugelrohr distillation to give a colorless oil, identified as benzyl alcohol-*d₁* (0.98 g, 9 mmol, 90% yield).

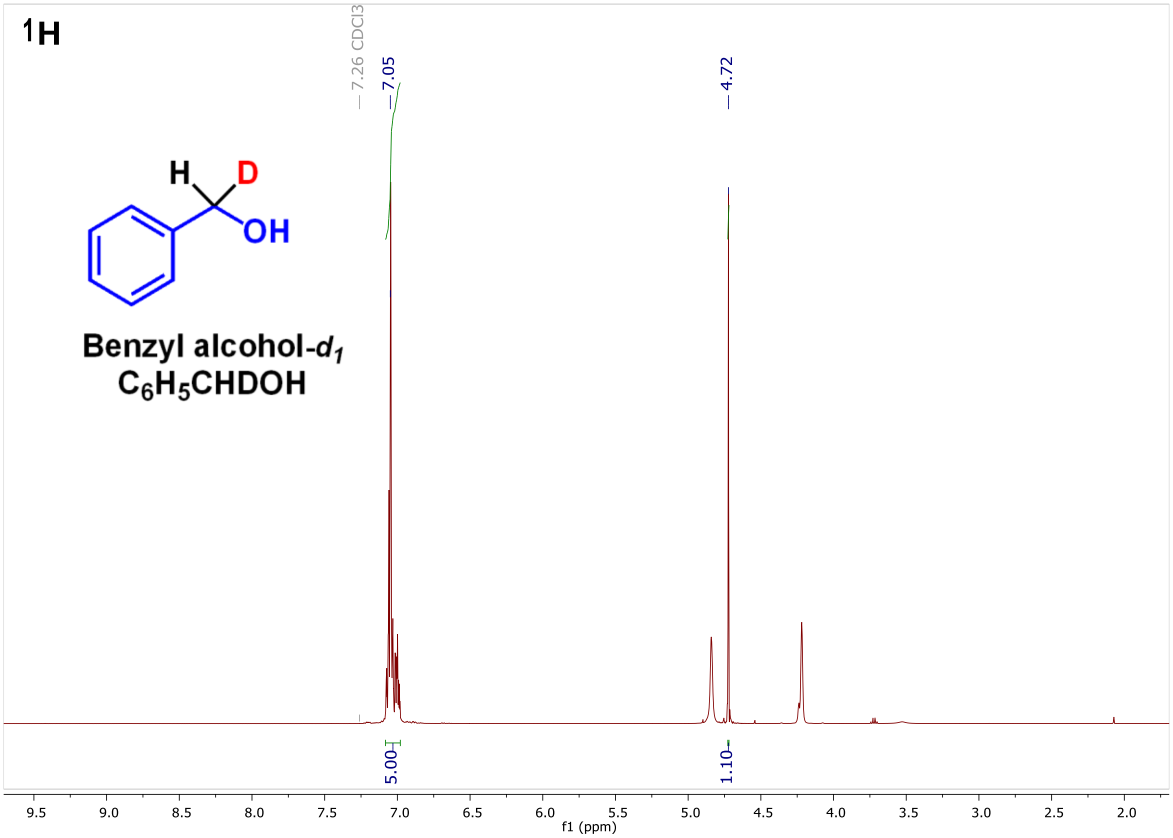

Figure S25 ^{1}H NMR spectrum for **benzyl alcohol-*d₁* (C_6D_5CHDOH)** in chloroform-*d*

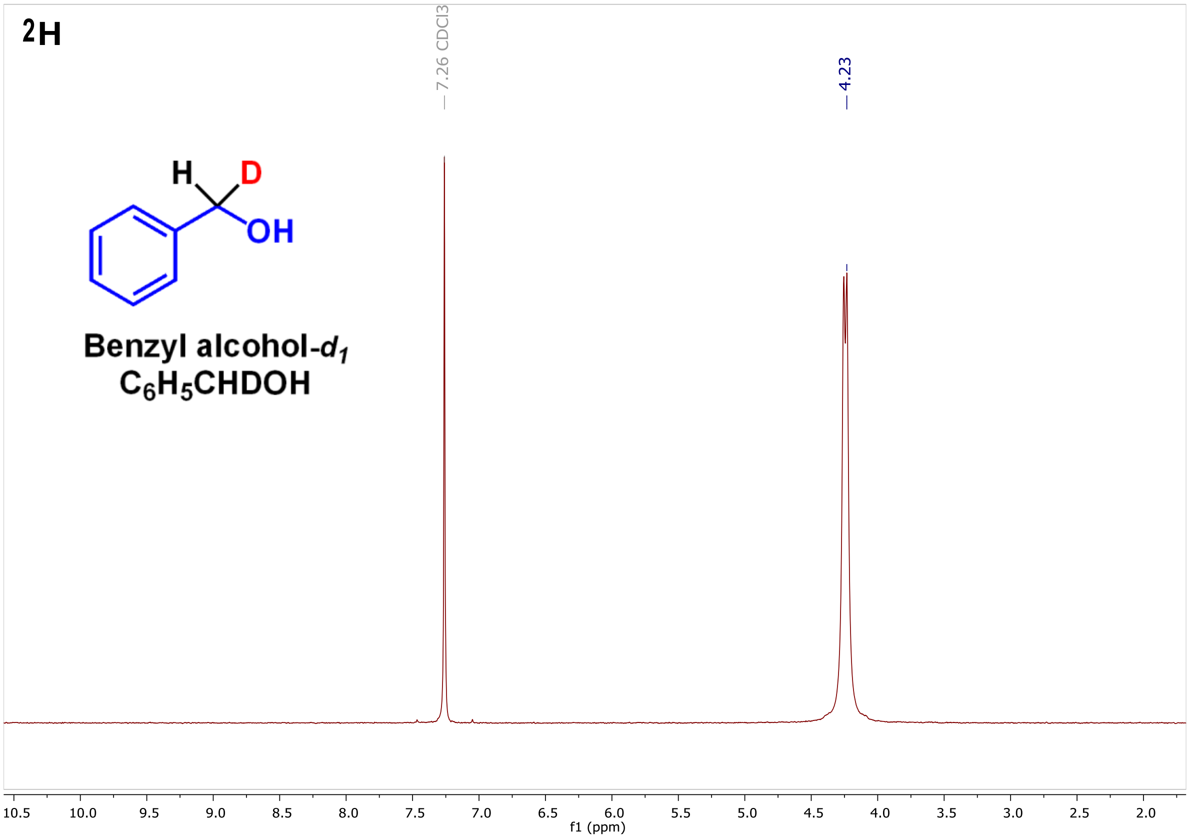

Figure S26 ^{2}H NMR spectrum for **benzyl alcohol-*d₁* (C_6D_5CHDOH)** in chloroform-*d*

Benzyl alcohol-OD ($C_6H_5CH_2OD$)[28,29]

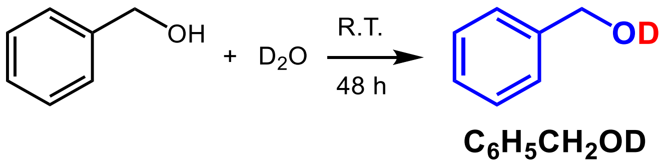

Benzyl alcohol (1.030 mL, 1.08 g, 10 mmol) was added to a flask and dissolved in D_2O (10 mL, 554 mmol), the solution was stirred for 48 h at room temperature. Then, the mixture was Kugelrohr distilled, the resulting liquid was dissolved in CH_2Cl_2, dried over $NaSO_4$, filtered and the solvent removed under vacuum to afford a colorless liquid, identified as benzyl alcohol-OD (0.65 g, 6 mmol, 60% yield).

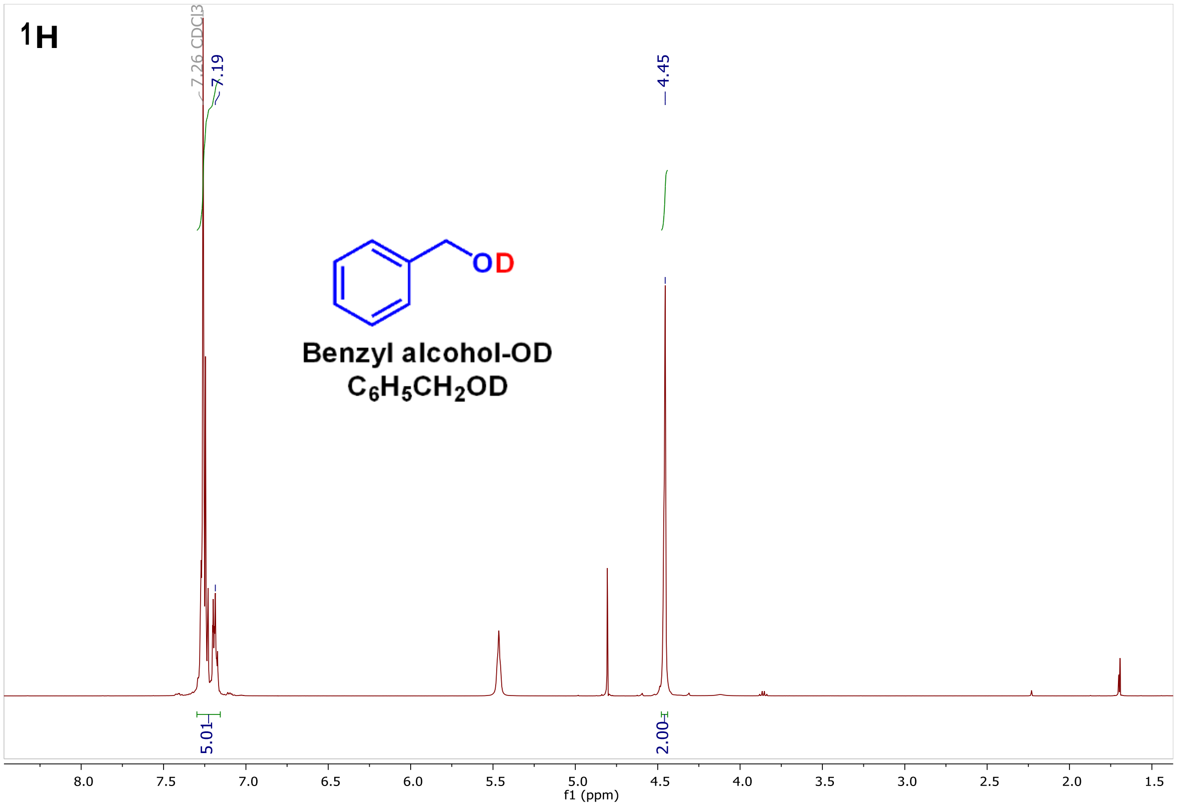

Figure S27 ¹H NMR spectrum for **benzyl alcohol-OD ($C_6H_5CH_2OD$)** in chloroform-*d*

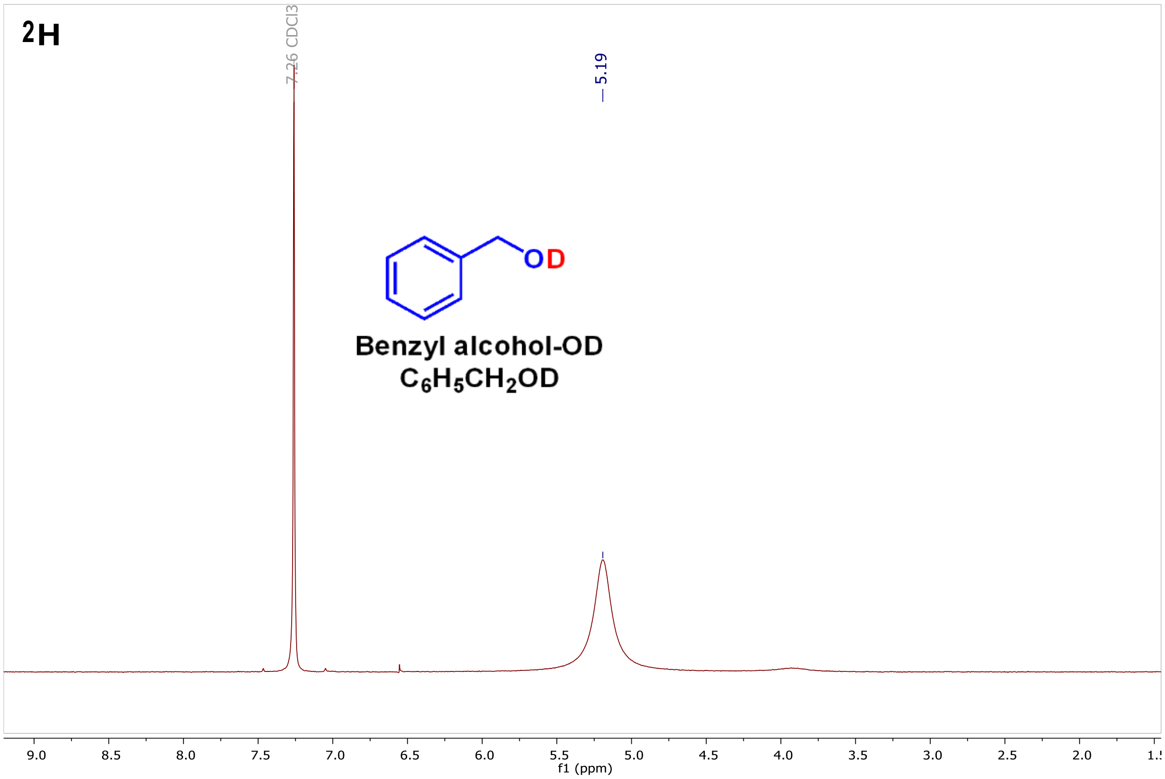

Figure S28 ²H NMR spectrum for **benzyl alcohol-OD ($C_6H_5CH_2OD$)** in chloroform-*d*

Acetophenone-d_3 ($C_6H_5COCD_3$)[30–33]

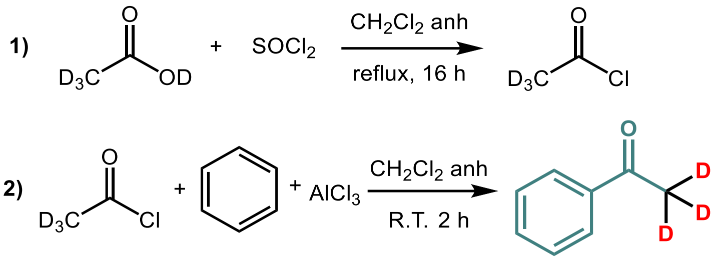

Acetic acid-d_4 (5 g, 78 mmol) was added to a Schlenk flask under argon atmosphere and dissolved in CH_2Cl_2 anh. (40 mL). The solution was cooled to 0 °C in an ice bath before a solution of $SOCl_2$ (11.1 g, 94 mmol) in CH_2Cl_2 anh. (10 mL) was added dropwise using a dropping funnel. Once the dropwise addition was complete, the mixture was refluxed for 16 h. A colorless solution containing the acetylchloride-d_3 was obtained and was used for the following reaction without any purification.

$AlCl_3$ (20.8 g, 156 mmol) was added to a Schlenk flask in a glove bag under argon atmosphere and dissolved in CH_2Cl_2 anh. (30 mL), C_6H_6 anh (4.06 g, 78 mmol) was added to the mixture cooled to 0°C while stirring. The solution of acetylchloride-d_3 from the previous step, was added dropwise using a dropping funnel to the solution. Once the dropwise addition was complete, the mixture was allowed to warm to room temperature and stirred for 2.5 h. Then the dark reaction mixture was cooled to 0°C and carefully quenched with cold water (100 mL) and the resulting precipitate was dissolved by adding concentrate HCl (30 mL) and the phases were separated. The aqueous layer was extracted three times with CH_2Cl_2. The organic layer was washed with a saturated aqueous solution of NaOH and again with water, dried over $NaSO_4$ and filtered. The solvent was removed under vacuum, resulting in a red oil which was submitted to Kugelrohr distillation to give a colorless oil, identified as acetophenone-d_3 (3.5 g, 28.1 mmol, 36% overall yield).

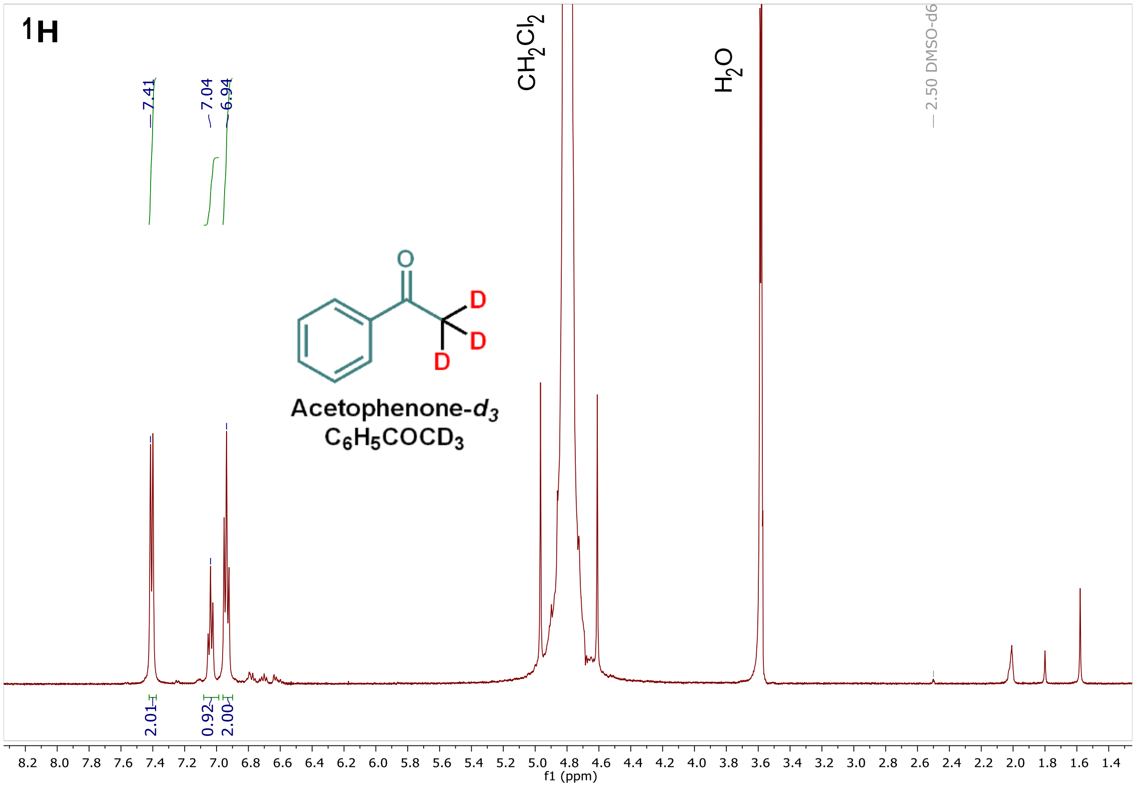

Figure S29 ^{1}H NMR spectrum for **acetophenone-*d*$_3$ ($C_6H_5COCD_3$)** in DMSO-*d*$_6$

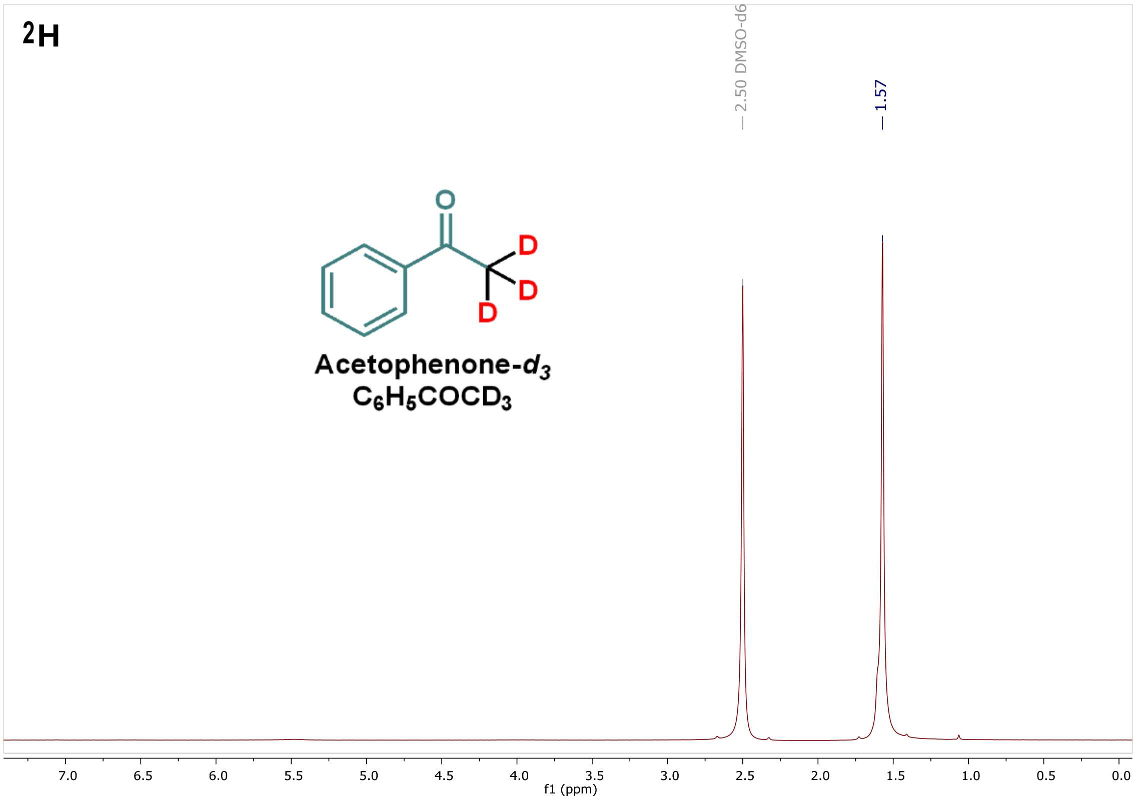

Figure S30 ^{2}H NMR spectrum for **acetophenone-*d*$_3$ ($C_6H_5COCD_3$)** in DMSO-*d*$_6$

Experiment with trifluoroacetic acid

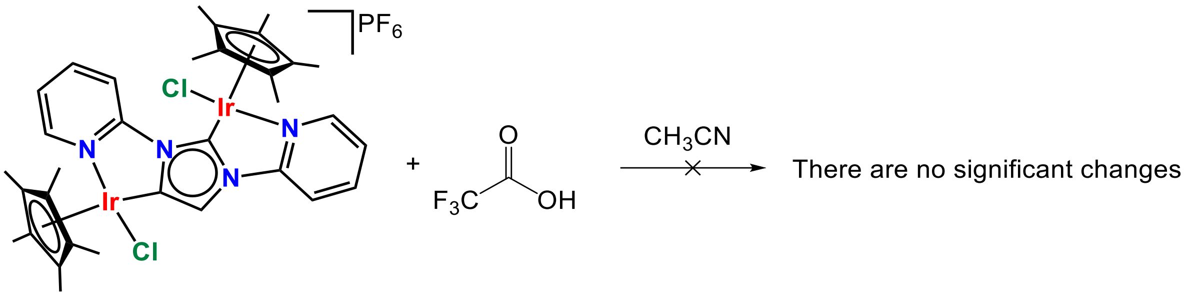

[Ir-L²-Ir]

In a round bottom flask, **[Ir-L²-Ir]PF₆** (100 mg, 0.09 mmol), trifluoroacetic acid (10.44 mg, 0.09 mmol) were added and dissolved in CH_3CN (30 mL). The mixture was stirred at room temperature for 30 minutes, an aliquot was obtained and analyzed by ^1H NMR, no change was observed. Thus 2 equivalents of trifluoroacetic acid were added and the mixture was stirred overnight, after this time, an aliquot was analyzed, resulting in no change. The mixture was heated to 50°C and 22 equivalents of trifluoroacetic acid were gradually added. After 13 days slight changes were observed in the ^1H NMR spectrum. The temperature increased to 72°C (trifluoroacetic acid boiling point) and after 1 day, an aliquot was analyzed, the spectrum indicated recovery of **[Ir-L²-Ir]PF₆**.

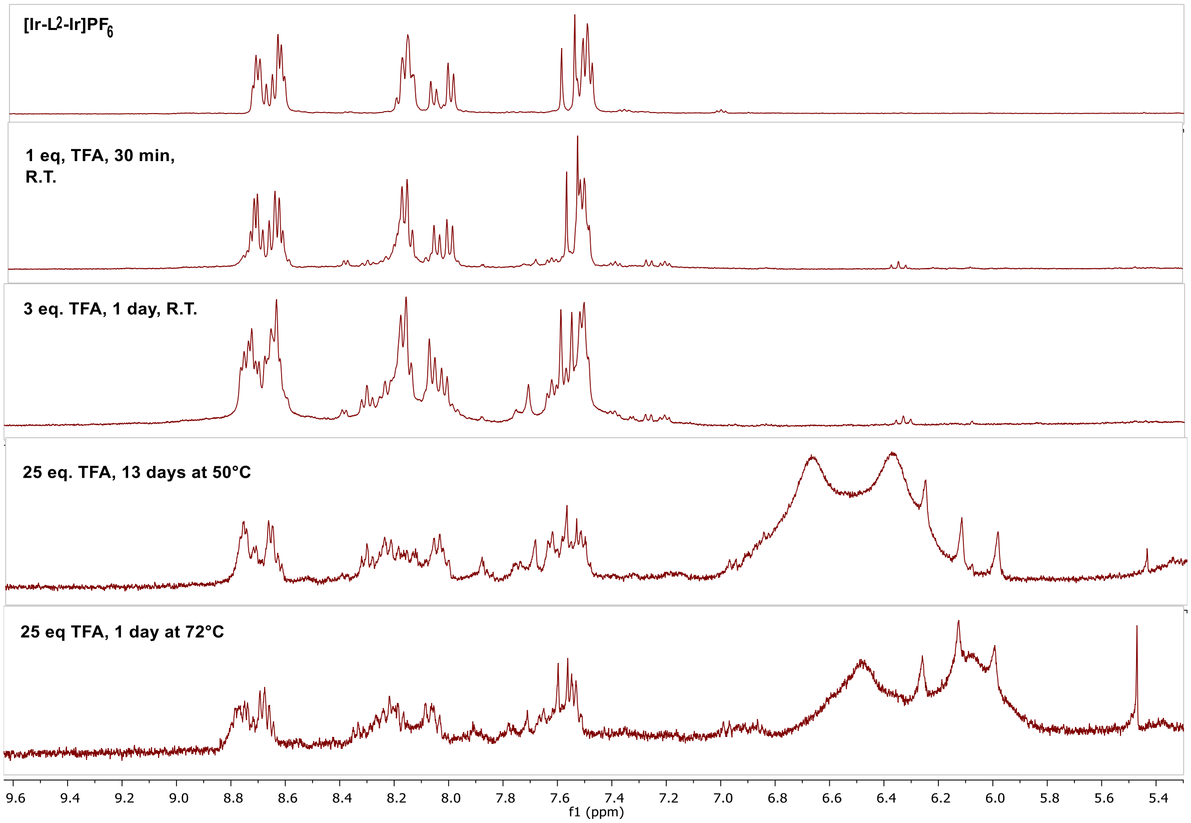

Figure S31. Stacked ^1H NMR spectra for experiments with trifluoroacetic acid and **[Ir-L²-Ir]PF₆** in acetonitrile-d_3 (5.4 - 9.6 ppm)

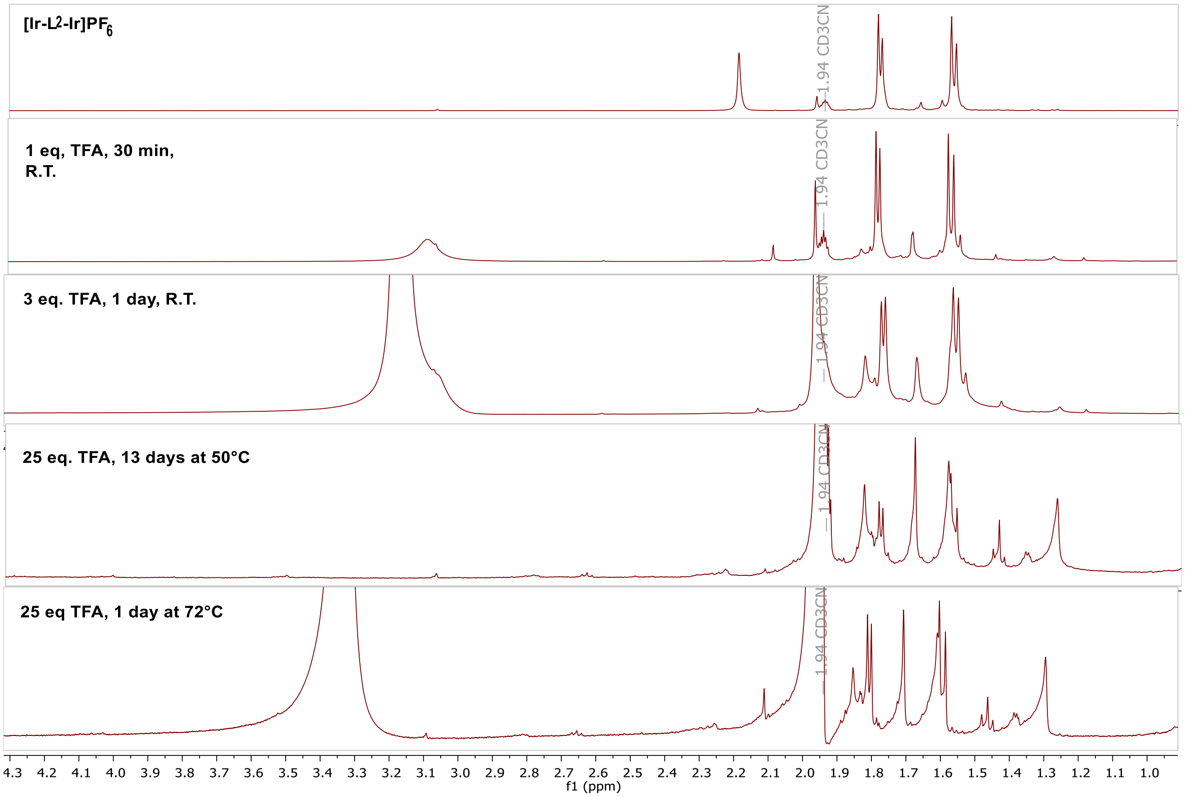

Figure S32 Stacked ^{1}H NMR spectra for experiments with trifluoroacetic acid and **[Ir-L^2-Ir]PF$_6$** in acetonitrile-*d*$_3$ (1 – 4.3 ppm)

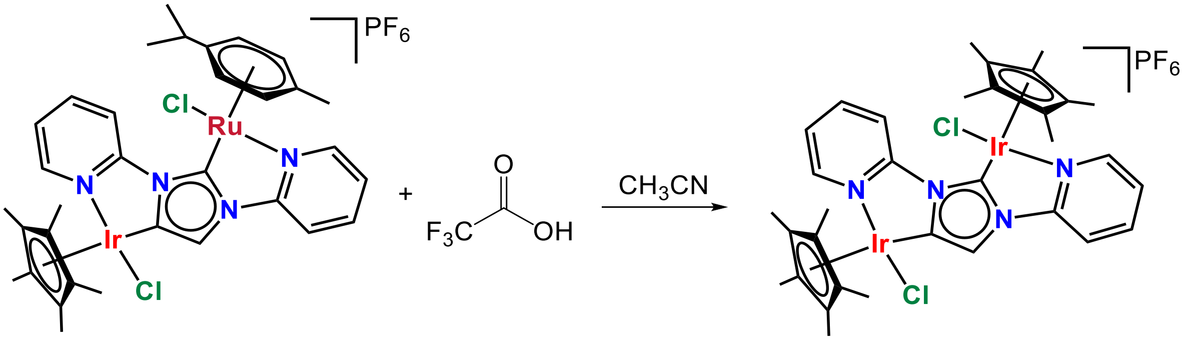

In a round bottom flask, **[Ru-L^2-Ir]PF$_6$** (100 mg, 0.1 mmol), trifluoroacetic acid (11.4 mg, 0.1 mmol) were dissolved in 30 mL of CH_3CN. The mixture was stirred at room temperature for 1 h, an aliquot analyzed by ^{1}H NMR, indicating no change. Further stirring overnight, showed slight changes in the ^{1}H NMR spectrum. The mixture was heated to 55°C and 14 equivalents of trifluoroacetic acid were gradually added. After 10 days under those conditions, we observed that the **[Ru-L^2-Ir]PF$_6$** had been completely consumed. Diethyl ether was added to the resulting mixture to precipitate, however no solid formed. Then the solvent was removed under vacuum, and the resulting oil was dissolved in dichloromethane and extracted with an aqueous solution of Na_2HCO_3. The organic layer was dried over Na_2SO_4, filtered, and the solvent was removed under vacuum producing a black solid, which was analyzed by MS. The solid was identified as **[Ir-L^2-Ir]PF$_6$**.

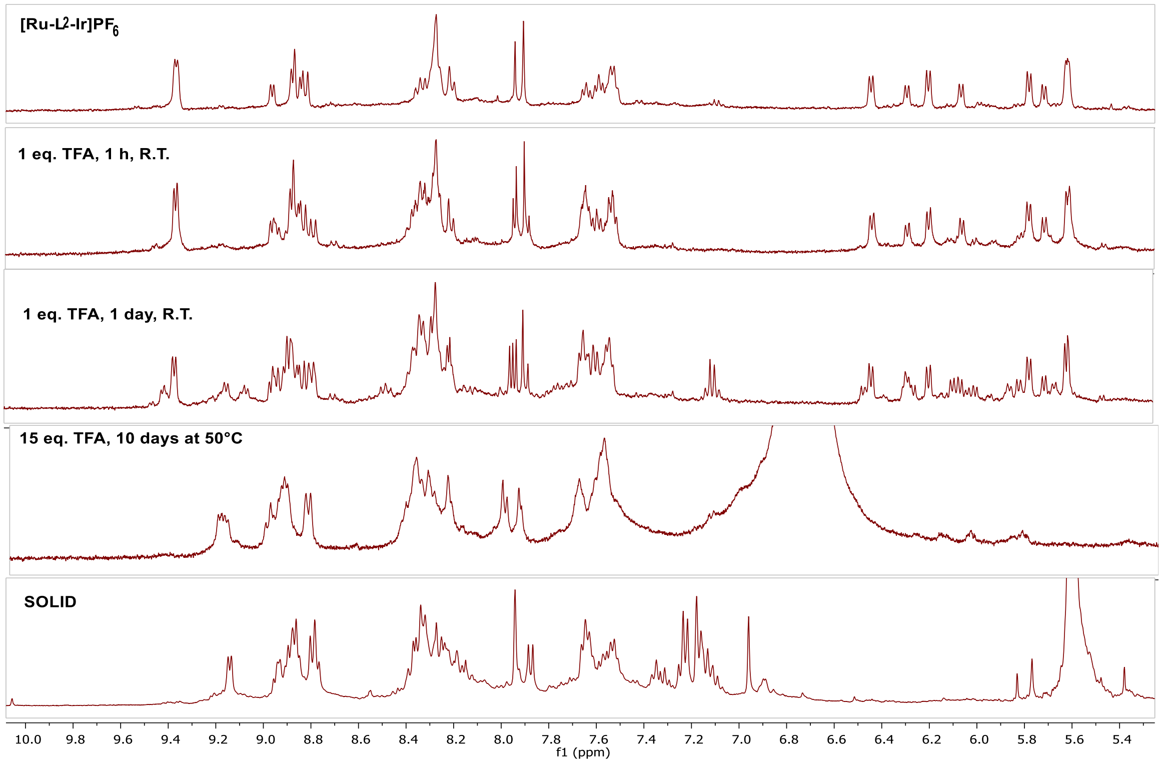

Figure S33 Stacked ^{1}H NMR spectra for experiments with trifluoroacetic acid and **[Ru-L^2-Ir]PF$_6$** in acetone-d_6 (5.4 - 10 ppm)

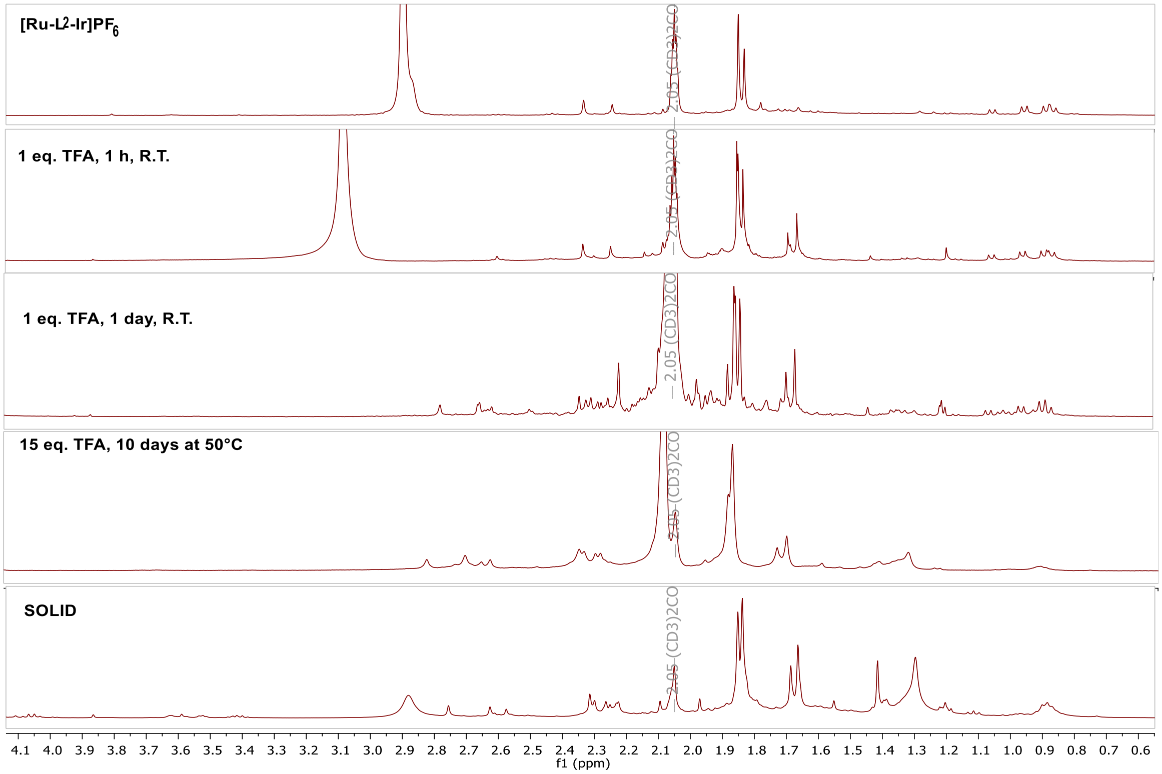

Figure S34 Stacked ^{1}H NMR spectra for experiments with trifluoroacetic acid and **[Ru-L^2-Ir]PF$_6$** in acetone-d_6 (0.6 – 4.1 ppm)

Single crystal X ray crystallography

Mononuclear complexes

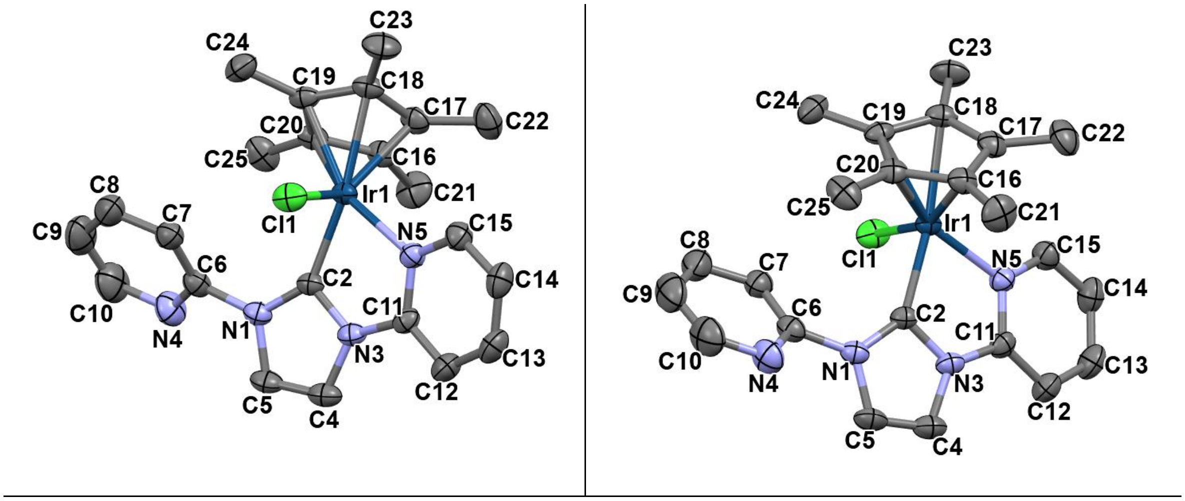

Isomer A **Isomer B**

Figure S35 Crystal structure of **[Ir-L^2]PF$_6$**, thermal ellipsoids drawn at 40 % probability. The **PF$_6$** and all hydrogen atoms have been omitted for clarity.

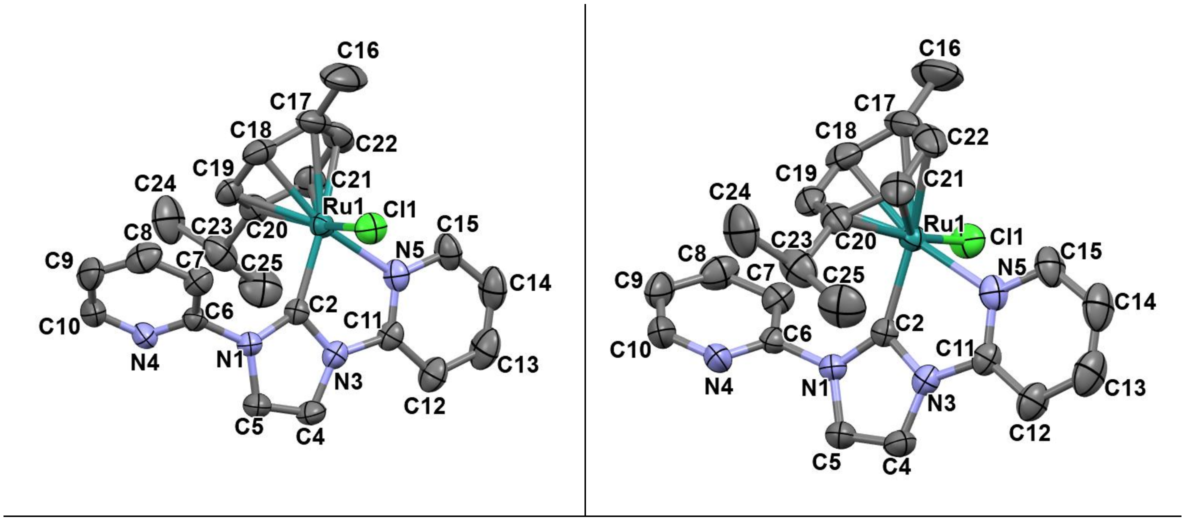

Isomer A **Isomer B**

Figure S36 Crystal structure of **[Ru-L^2]B(Ph)$_4$**, thermal ellipsoids drawn at 40 % probability. The **B(Ph)$_4$** and all hydrogen atoms have been omitted for clarity.

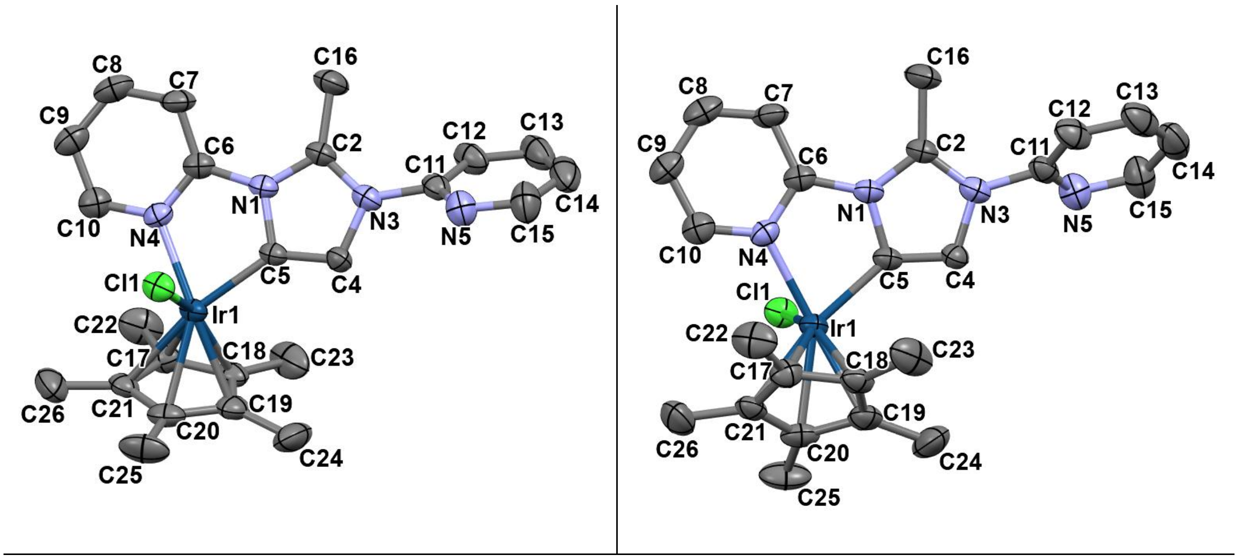

Isomer A **Isomer B**

Figure S37 Crystal structure of **[Ir-L^2]PF$_6$**, thermal ellipsoids drawn at 40 % probability. The **PF$_6$** and all hydrogen atoms have been omitted for clarity.

<u>Dinuclear complexes</u>

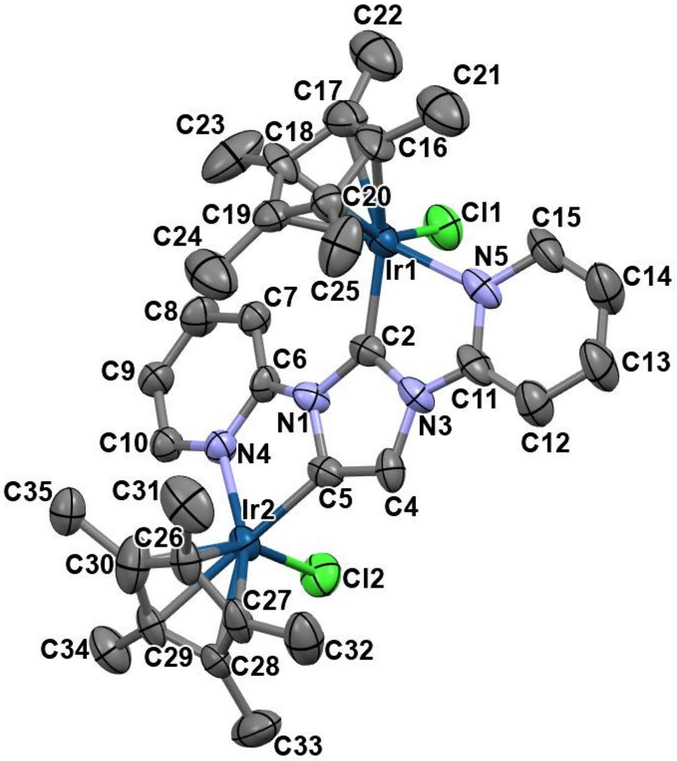

Figure S38 Crystal structure of **[Ir-L^2-Ir]PF$_6$**, thermal ellipsoids drawn at 40 % probability. The **PF$_6$** and all hydrogen atoms have been omitted for clarity.

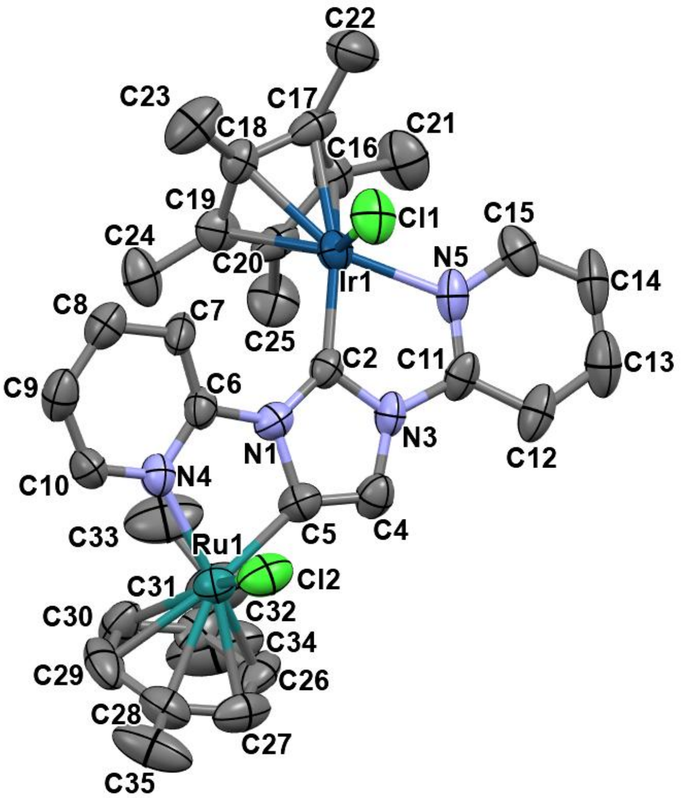

Figure S39 Crystal structure of **[Ir-L^2-Ru]PF$_6$**, thermal ellipsoids drawn at 40 % probability. The **PF$_6$** and all hydrogen atoms have been omitted for clarity.

Table S2 X-ray crystallographic data for all complexes

Complex	**[Ir-L^2]PF$_6$**	**[Ru-L^2]BPh$_4$**	**[Ir-L^3]PF$_6$**	**[Ir-L^2-Ir]PF$_6$**	**[Ir-L^2-Ru]PF$_6$**
Empirical Formula	$C_{25}H_{28}ClN_5IrPF_6$	$C_{47}H_{46}BClN_4Ru$	$C_{25}H_{28}Cl_4N_4IrPF_6$	$C_{33}H_{39}Cl_2N_4Ir_2PF_6$	$C_{33}H_{38}Cl_2N_4IrRuPF_6$
Formula weight	771.14	814.25	863.48	1091.99	999.81
T (K)	294(2)	298(2)	295(2)	294(2)	293(2)
Wavelength (Å)	0.71073	0.71073	0.71073	0.71073	0.71073
Crystal System	Triclinic	Orthorhombic	Triclinic	Monoclinic	Orthorhombic
Space group	P-1	Pna2$_1$	P-1	P 2$_1$/c	Pbca
a (Å)	8.4134(4)	18.433(2)	8.6790(3)	19.7246(9)	8.3860 (17)
b (Å)	13.1977(7)	14.757(2)	10.9992(4)	8.4573(4)	24.075 (5)
c (Å)	14.1274(7)	29.609(4)	16.6390(7)	49.159(2)	35.442 (7)
α (°)	65.007(2)	90	103.444(2)	90	90
β (°)	82.653(2)	90	95.210(2)	100.817(2)	90
γ (°)	88.264(2)	90	99.882(2)	90	90
V (Å^3)	1409.62(12)	8054.3(18)	1507.72(10)	8054.8(7)	7155 (2)
Z	2	8	2	8	8
ρ_{calcd} (g/cm^3)	1.817	1.340	1.902	1.801	1.856
μ (mm^{-1})	4.952	0.494	4.897	6.829	4.39
F(000)	752.0	3360.0	840.0	4176	3904.0
Crystal size (mm) / colour / shape	0.24 x 0.19 x 0.07 / yellow / plate	0.350 x 0.120 x 0.04 / red / plate	0.38 x 0.28 x 0.13 / yellow / plate	0.200 x 0.060 x 0.020 / yellow / plate	0.22 x 0.1 x 0.02 / yellow / needle
Θ range for data collection	2.44 to 38.782	2.240 to 27.204	1.27 to 25.018	2.408 to 18.773	4.22 to 25.079
Index ranges	-14 ≤ h ≤ 14, -23 ≤ k ≤ 23, -23 ≤ l ≤ 24	-23 ≤ h ≤ 23, -18 ≤ k ≤ 18, -30 ≤ l ≤ 38	-10 ≤ h ≤ 10, -13 ≤ k ≤ 13, -19 ≤ l ≤19	-17 ≤ h ≤17, -7 ≤ k ≤ 7, -44 ≤ l ≤ 44	-10 ≤ h ≤ 10, -28 ≤ k ≤ 28, -42 ≤ l ≤ 42
Completeness (Θ)	99.9% (25.242°)	99.9% (25.242°)	99.1% (25.018°)	99.7% (18.774°)	99.5% (25.079°)
Data / restraints / parameters	16082 / 0 / 358	17884 / 1 / 980	5265 / 163 / 376	6261 / 2 / 639	6309 / 2 / 423
R(int)	0.0828	0.1467	0.0482	0.1110	0.0
Goof (F^2)	1.017	1.025	1.064	1.156	1.165
R1, wR$_2$ (I>2sigma(I))	R1 = 0.0445, wR2 = 0.0908	R1 = 0.0408 wR2 = 0.0920	R1 =0.043 wR2 =0.1078	R1 = 0.0443 wR2 = 0.0849	R1 =0.0795 wR2 =0.1679
R1, wR2 (all)	R1 = 0.1036, wR2 = 0.194	R1 = 0.0630 wR2 = 0.1046	R1 = 0.0523 wR2 =0.1132	R1 = 0.0582 wR2 =0.0953	R1 =0.1419 wR2 =0.2052
S(all)	1.026	1.025	1.064	1.111	1.165

Structural refinement details for complexes

[Ir-L³]PF₆

The $\mathbf{PF_6^-}$ anion was disordered over two positions with 41:59 occupancy, hence it was modeled using SIMU, RIGU, SADI, and SAME restraints implemented in the DSR plugin tool installed in shelXle Qt5.[34,35]

[Ir-L²-Ir]PF₆

Disordered solvent could not be modeled successfully, thus the electron density was removed from the reflection data with PLATON SQUEEZE[36] (246 electrons and solvent accessible void volume 1337 Å^3, the electron count suggests the presence of approximately one MeOH molecule per asymmetric unit of **[Ir-L²-Ir]PF₆** complex).

The check cif for **[Ir-L²-Ir]PF₆** complex shows an alert A:

```
THETM01_ALERT_3_A  The value of sine(theta_max)/wavelength is less than 0.550
             Calculated sin(theta_max)/wavelength =     0.4528
```

The low value of sine(theta_max)/wavelength is attributed to the limited diffraction quality of the complex [Ir-L^2-Ir]PF_6 crystals, which resulted in weak high-angle reflections and consequently restricted the data resolution. Despite the limited resolution, all non-hydrogen atoms, particularly the iridium were clearly identified in the Fourier electron density maps, and the molecular connectivity was unambiguously established. Furthermore, the structure exhibited significant solvent disorder that could not be reliably modeled; therefore, it was treated using the SQUEEZE routine implemented in PLATON, which significantly improved the final refinement residuals and the overall structural model.

EADP constraints were used for C1, C2, C3, C5, C6, C8, C10, C11, C12, C15, C31, C45, C46 and C58. DELU command was used to restrain the bonds: N4-C1 and C14-C24.

[Ir-L²-Ru]PF₆

Poor quality of the single crystal obtained for **[Ir-L²-Ru]PF₆**, a preliminary structure was solved and refined. Additionally, the measurement was made before the maintenance of the equipment, which was not aligned correctly and the strategy used in the measurement was not adequate. Unfortunately, after several attempts, it was not possible to crystallize again this compound.

The check cif for **[Ir-L²-Ru]PF₆** complex shows some alerts B:

```
Alert level B
PLAT342_ALERT_3_B Low Bond Precision on  C-C Bonds ...............     0.02483 Ang.
PLAT910_ALERT_3_B Missing FCF Reflection(s) Below Theta(Min)[Deg]=        4.22 Note
                0  2  0,   0  4  0,   1  1  1,   0  2  1,   1  2  1,   1  3  1,
                0  4  1,   1  4  1,   0  0  2,   1  0  2,   1  1  2,   0  2  2,
                1  2  2,   1  3  2,   0  4  2,   1  1  3,   0  2  3,   1  2  3,
                1  3  3,   0  4  3,   0  0  4,   1  0  4,   1  1  4,   0  2  4,
                   (    8 More Missing: see the .ckf listing file)
```

PLAT342_ALERT_3_B Low Bond Precision on C-C Bonds .... 0.02483 Ang.

The low bond precision is attributed to the poor quality of the single crystal obtained for the complex [Ir-L^2-Ru]PF_6. Unfortunately, after numerous crystallization attempts using different solvent mixtures and temperatures, it was not possible to crystallize again this compound. The structure was refined to the best possible model to confirm the atom connectivity and the presence of both metal centers. Given the chemical importance of this specific assembly and the difficulty in reproducing the crystallization, these data are presented as the most reliable representation of the molecular structure. Which are due to the reasons mentioned above.

PLAT910_ALERT_3_B Missing FCF Reflection(s) Below Theta(Min)[Deg]= 4.22

The missing low-angle reflections are attributed to physical shadowing by the beamstop assembly. Due to the small crystals obtained of the complex [Ir-L^2-Ru]PF_6, the collection strategy was optimized to prioritize high-angle data. Their absence is localized to a few low-order reflections and does not compromise the structural solution, the refinement of the metal centers, or the overall reliability of the model. Moreover, EADP constraints were used for C16 and DFIX restrained was used for C31-C32 and C27-C28 bonds.

Catalytic studies

Mechanism for the α-alkylation/transfer hydrogenation reaction proposed in previous reports

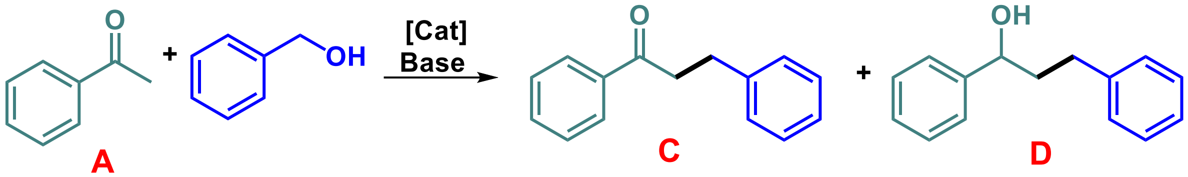

A C D

According to the previously reported mechanisms for the tandem α-alkylation/hydrogen transfer reaction (Scheme S1),[37–40] the first step is the dehydrogenation of benzyl alcohol to the aldehyde **G**. This is achieved through synergy between the base and the catalyst, wherein the chloride ligand dissociates from the metal center to generate a vacant coordination site, this allows for the formation of a metal-hydride species, which is responsible for borrowing hydrogen process; subsequent hydrogenation steps then take place, producing KCl and H_2O as by-products.

The second step consists of an aldol condensation between the aldehyde **G** and the enolate generated from **A** via an enolate-ketone equilibrium in the presence of the base. This results in the formation of a new C-C bond, yielding the chalcone intermediate and H_2O as a by-product.

The third step involves the 1,4-hydrogenation of the chalcone through a process of "return" of hydrogen from the previously formed metal-hydride species, resulting in the alkylated product **C**. The fourth and final step is the reduction of the C=O bond of product **C** through a hydrogen transfer reaction. In this step, a metal-hydride species regenerated by the process described in step 1, ultimately yielding product **D** after three consecutive catalytic reactions.

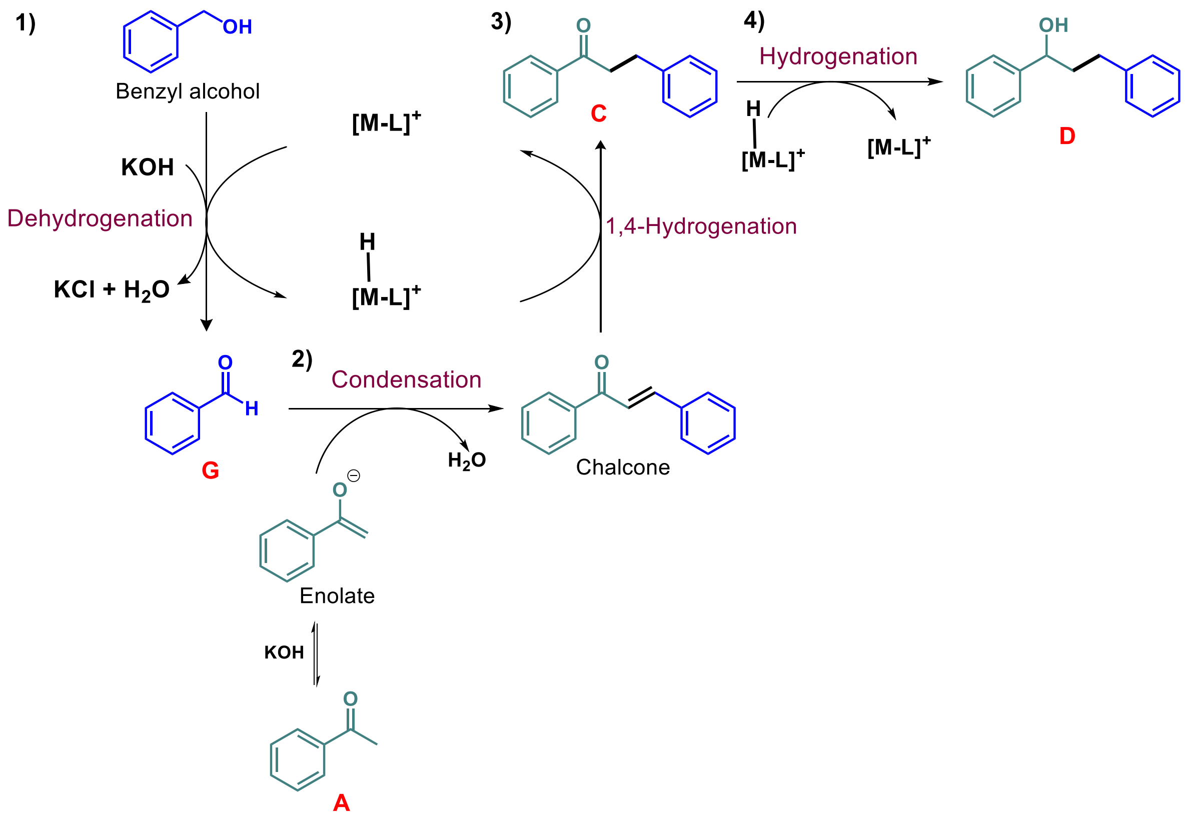

Scheme S1 General mechanism for the tandem reaction of α-alkylation and hydrogen transfer reaction.[37–40]

Similar studies reported in the literature for catalytic tandem α-alkylation/transfer hydrogenation reaction.

Table S3 Similar tandem α-alkylation/transfer hydrogenation catalysis systems reported in the literature

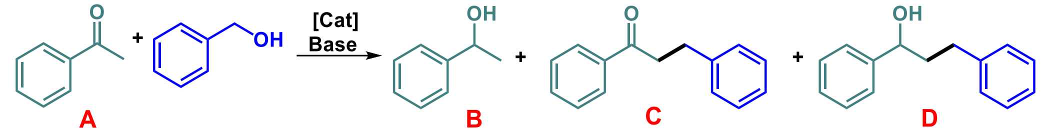

Entry	Catalyst (% mol)	Solvent	T (°C)	Time (h)	Base (% mol)	Conv A %	Yield %		Ref
							C	D	
1	$RuCl_2(PPh_3)_3$ (5)	Dioxane	80	20	KOH (300)			77	41
2	$RuCl_2(DMSO)_4$ (2) PPh_3 (2)	Dioxane	80	24	KOH (100)			82	40
3	$[Ru(DMSO)_4]Cl_2$ (2)	Dioxane	80	24	KOH (100)		72		40
4	$RuCl_2(DMSO)_4$ (2)	Dioxane	80	24	KOH (120)		78	6	40
5	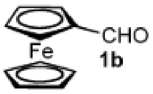 (5)	p-xylene	130	12	NaOH (20)		38	23	42
6	-	Toluene	110	12	LiO*t*Bu (200)		92		29
7	$Rh(acac)_3$ (1) DABCO (50)	Toluene	110	4	KOH (100)			95	43
8		Neat	130	24	NaOH (100)	98	3	95	44
9	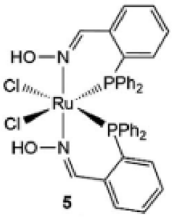 (2)	Toluene	120	3	KOH (100)	>99		87(75)	45
10	NiBr2 (5) 1,10-phenanthroline (6) TON: 16.4 TOF: 0.46 h^{-1}	Toluene	140	36	*t*-BuOK (20)		82		46
11	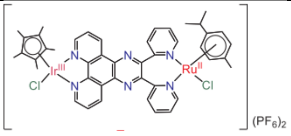 (0.1)	Benzyl alcohol	130	8	KOH (100)			97	47
12	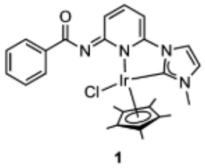 (0.05) TON :1940 TOF: 3880 h^{-1}	Toluene	130	0.5	KOH (10)		97		39

**13**	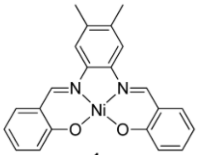 1c (1)	Toluene	135	3	NaOH (5)		87		48

General conditions for the catalytic tandem α-alkylation/transfer hydrogenation reactions

In a pressure tube the catalyst (0.025 mmol for dinuclear complexes or 0.05 mmol for mononuclear complexes) and base (0.1 mmol) were added. Acetophenone (116.65 µL, 120.15 mg, 1 mmol) and benzyl alcohol (311.94 µL, 324.42 mg, 3 mmol) in 1:3 ratio was measured using a micropipette and added to the pressure tube. A suspension was observed. A magnetic stirrer bar was added to the pressure tube. The tube was heated in a sand bath at indicated temperature, a sample was taken and analyzed by ^{1}H NMR in $CDCl_3$.

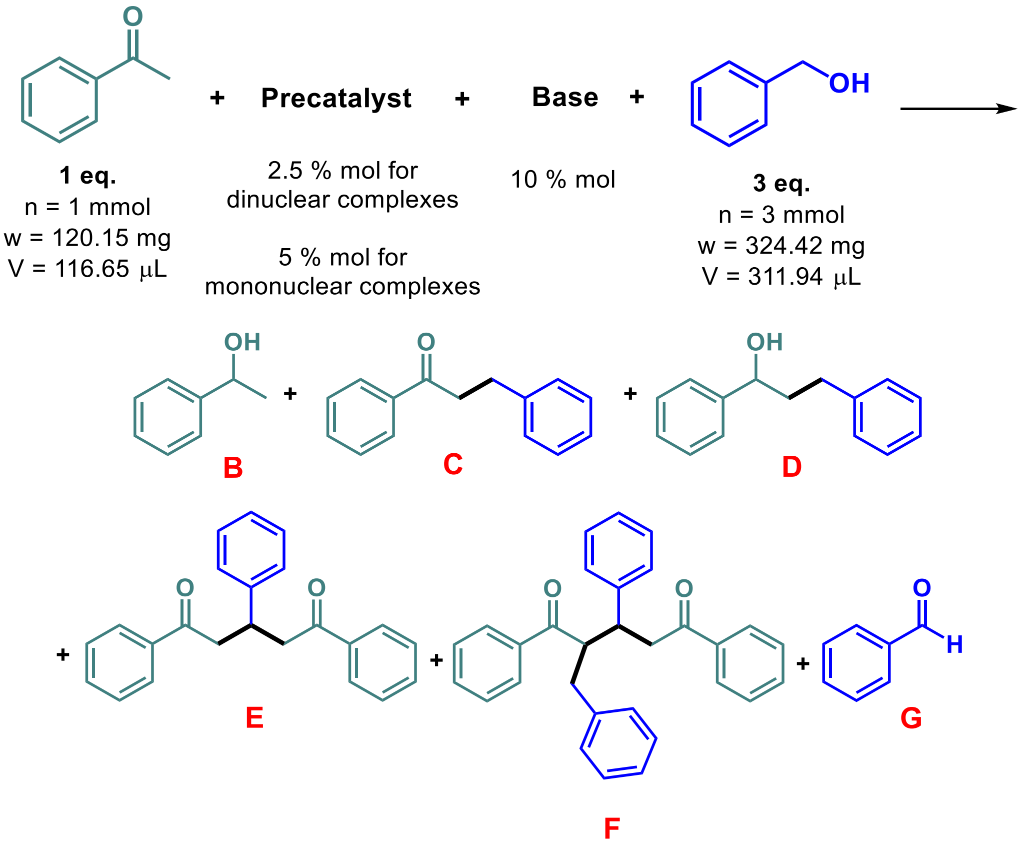

Scheme S2 General conditions for the catalytic tandem α-alkylation/transfer hydrogenation reactions.

Analysis of optimization of reaction conditions for tandem α-alkylation/transfer hydrogenation

The catalytic tandem α-alkylation and transfer hydrogenation reaction was selected as the benchmark experiment to probe the potential for cooperativity between Ru(II)-Ir(III) bridged by one NHC ligand. To find the optimal conditions, several parameters were screened. (Table S4). Initially, we used benzyl alcohol as solvent at reflux and KOH as base (Entry 1, Table S4), obtaining good conversion of **A**, but low yield for our desired tandem product **D** (13% at 2 h and 28% at 20 h), Thus, we employed toluene at reflux (Entry 2, Table S4) nevertheless, we did not observe any product at 2 h. We tried different alcohols as solvents such as isopropanol and methanol (Entries 3 and 4, Table S4), however, we observed other byproducts such as alkylation between acetophenone and the corresponding alcohol. Therefore, we decided to try the reaction in neat conditions evaluating at 130°C, 100°C and 70°C (Entries 5-7, Table S4). The best performance was observed at 100°C for 2 h, on further heating for 20 h the products returned to substrate, decreasing the conversion. Therefore, the following reactions were performed in neat conditions at 100°C for 2 h.

The effect of the coordinated chloride ligands was evaluated by adding silver salt to abstract them. Hence $AgBF_4$ or $AgPF_6$ (5 % mol) was added to the reaction (Entries 8 and 9, Table S4), surprisingly this silver salt interferes in the catalysis, deactivating the catalytic activity.

Interestingly, negligible formation for **C** and **D** products were obtained when 10 % mol catalyst or 5 % mol of base loading was used (Entries 10 and 11, Table S4).

A screening of other bases, such as Li_2CO_3, KO^tBu, Et_3N, $Ba(OH)_2·8H_2O$, NaOH, Cs_2CO_3, NaO^tBu (Entries 12-18, Table S4) did not improve the reaction outcome. Moreover, in some cases, a higher benzaldehyde production was observed, indicating that only the benzyl alcohol oxidation took place.

Self-condensation product of acetophenone

A control experiment was performed using a **1:1** ratio of acetophenone and KOH, in the absence of benzyl alcohol and metal complex, under the same conditions employed for the catalytic reactions. Observing the formation of the β-hydroxyketone product from the acetophenone self-condensation, Figure S40, but not dehydration step to produce the chalcone derivative was observed under these conditions.

Another control experiment was carried out using only acetophenone and 10 % mol of base, in the absence of benzyl alcohol and metal complex, under the same conditions employed for the catalytic reactions. Observing only trace amounts of the reduced acetophenone, while no acetophenone self-condensation product was detected, Figure S41

These results indicate that, although the aldol self-condensation reaction of acetophenone can occur under strongly basic conditions, using 10 % mol of base precludes the self-condensation reaction. Moreover, the self-condensation product was not detected in the catalytic runs using our pre-catalysts.

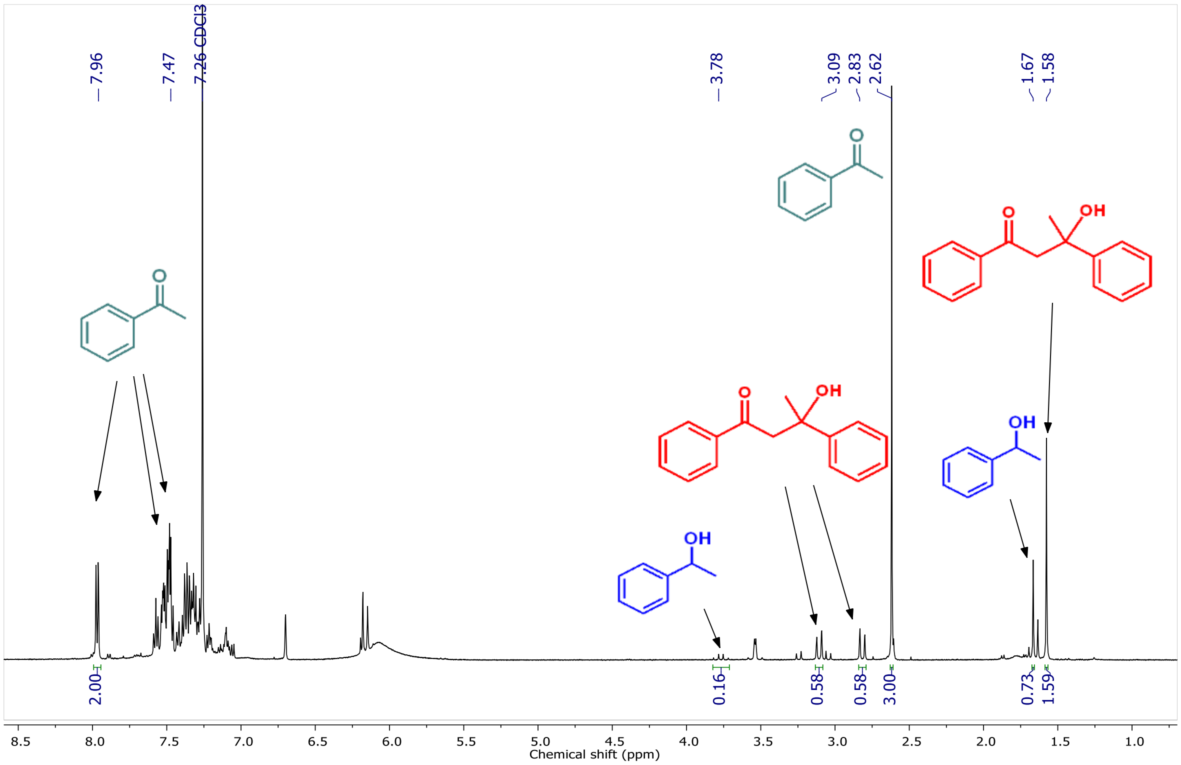

Figure S40 ^{1}H NMR spectrum of the control experiment using acetophenone and KOH in a 1:1 ratio.

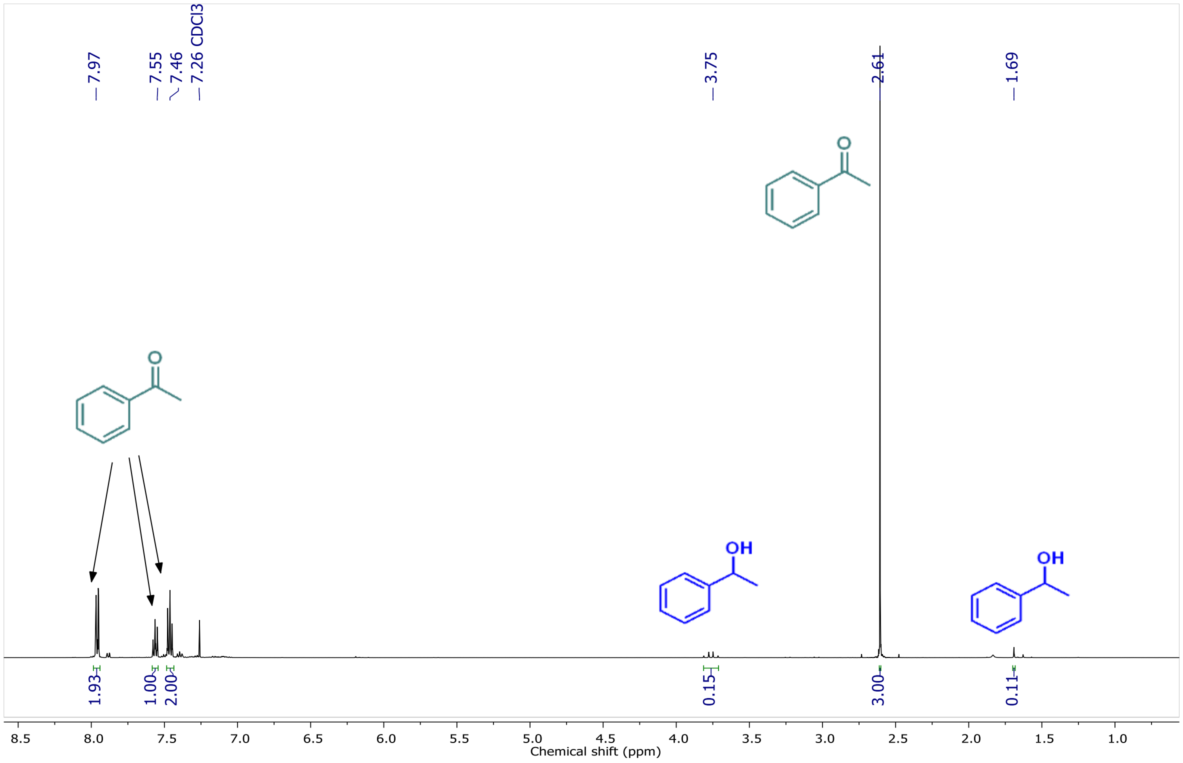

Figure S41 ^{1}H NMR spectrum of the control experiment using acetophenone with 10 mol % KOH in the absence of benzyl alcohol and metal complex

Optimization of reaction conditions for tandem α-alkylation/transfer hydrogenation

Table S4 Optimization of reaction conditions for tandem α-alkylation/transfer hydrogenation[a]

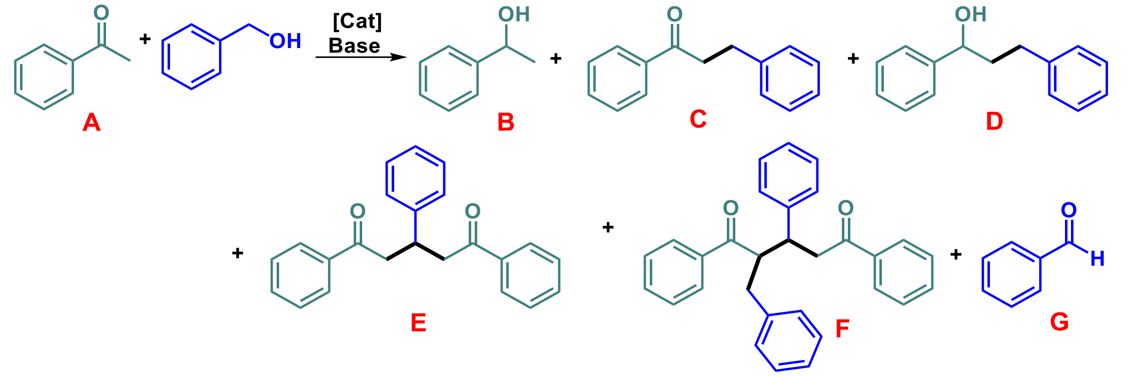

Entry	Solvent	T (°C)	Time (h)	Base	^{1}H NMR %Conv A	^{1}H NMR % Yield					
						B	C	D	E	F	G[b]
1	Benzyl alcohol	205	2	KOH	87	33	40	13	0	0	traces
			20		94	13	53	28	0	0	traces
2	Toluene	110	2	KOH	0	0	0	0	0	0	0
			20		46	24	22	0	0	0	0
3[c]	Isopropanol	82	2	KOH	95	82	traces	11	0	0	0
			20		94	64	0	29	0	0	0
4	Methanol	65	2	KOH	3	traces	0	0	0	0	traces
			20		11	11	0	0	0	0	traces
5	Neat conditions	130	2	KOH	77	traces	44	30	0	0	traces
			20		69	traces	30	37	0	0	traces
6	Neat conditions	100	2	KOH	95	traces	55	34	traces	0	traces
			20		97	traces	53	39	traces	0	6
7	Neat conditions	70	2	KOH	98	8	77	12	traces	traces	traces
			20		97	< 5	64	27	traces	traces	traces
8[d]	Neat conditions	100	2	KOH	18	12	< 5	0	traces	traces	9
			20		24	14	< 5	0	0	6	13
9[e]	Neat conditions	100	2	KOH	16	11	< 5	traces	0	0	6
			20		25	14	7	traces	0	0	6
10[f]	Neat conditions	100	2	KOH	35	23	10	traces	0	0	7
			20		40	22	12	6	0	0	7
11[g]	Neat conditions	100	2	KOH	25	14	11	0	0	0	traces
			20		13	11	traces	0	0	0	traces
12	Neat conditions	100	2	Li_2CO_3	< 5	< 5	0	0	0	0	traces
			20		18	18	0	0	0	0	< 5
13	Neat conditions	100	2	KO^{t}Bu	56	11	32	12	0	0	traces
			20		68	14	26	28	0	0	traces
14	Neat conditions	100	2	Et_3N	7	7	0	0	0	0	traces
			20		11	11	0	0	0	0	< 5
15		100	2		22	16	6	0	0	0	traces

	Neat conditions		20	$Ba(OH)_2 \cdot 8H_2O$	35	28	7	0	0	0	< 5
16	Neat conditions	100	2	NaOH	92	traces	63	18	traces	< 5	traces
			20		83	< 5	64	9	traces	traces	traces
17	Neat conditions	100	2	Cs_2CO_3	94	9	74	10	0	0	traces
			20		98	traces	65	29	0	0	< 5
18	Neat conditions	100	2	NaO^tBu	7	6	0	0	0	0	traces
			20		9	8	traces	0	0	0	13

[a] Reaction conditions: Acetophenone and benzyl alcohol in 1:3 ratio (1 mmol), 10 mol% base, 2.5 mol% **[Ir-L^2-Ru]** catalyst loading and 2 mL solvent. Conversion and yield were measured by ^{1}H NMR spectral analysis
[b] Benzaldehyde yield was calculated based on benzyl alcohol.
[c] Other products were observed
[d] $AgBF_4$ was added as additive
[e] $AgPF_6$ was added as additive
[f] 10 % mol catalyst loading
[g] 5 % mol base

TOF

$$TOF = \frac{n_C}{n_{cat} \ *t} \ [=] \ h^{-1} \qquad \textbf{(Eq. S1)}$$

$n_{\boldsymbol{C}} = moles\ of\ \boldsymbol{C}\ produced$

$n_{cat} = moles\ of\ catalyst$

$t = time = \ determined\ at\ 30\ minutes, when\ the\ catalyst\ is\ in\ full\ action$

TON

It refers to the turnovers of the aldol condensation and 1,4-hydrogenation reactions to obtain **C** at 30 minutes.

$$TON = \frac{n_C}{n_{cat}} \qquad \textbf{(Eq. S2)}$$

$n_{\boldsymbol{C}} = moles\ of\ \boldsymbol{C}\ produced$

$n_{cat} = moles\ of\ catalyst$

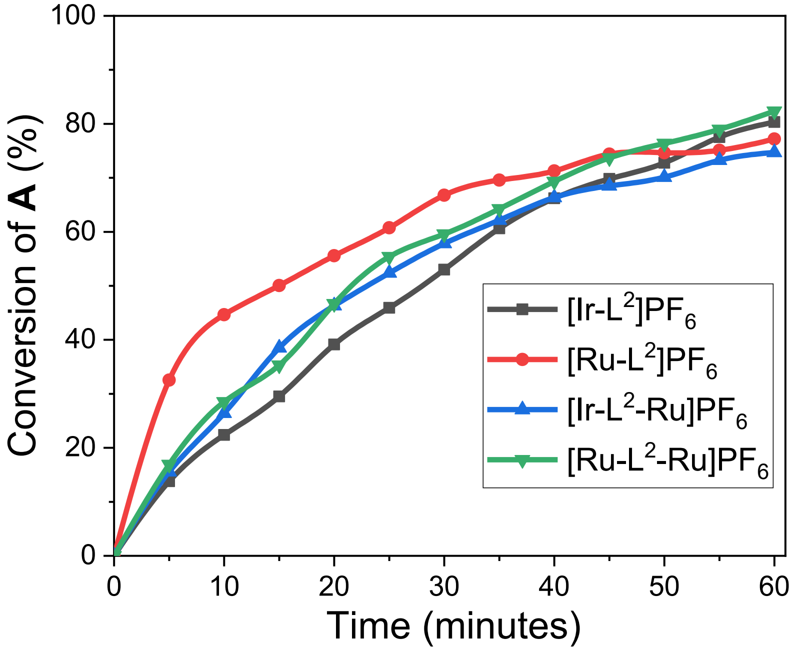

Figure S42 Conversion of **A** vs time. The plot shows that the catalyst reaches its maximum activity at approximately 30 minutes of reaction.

Experiments 1:1 ratio of acetophenone and benzyl alcohol

General conditions:

In a pressure tube the catalyst, (0.025 mmol, 25 mg for **[Ir-L^2-Ru]PF$_6$** complex or 0.05 mmol, 32 mg **[Ru-L^2]PF$_6$** or 37 mg for **[Ir-L^2]PF$_6$** mononuclear complexes) and KOH (0.1 mmol, 5.6 mg) were added. Acetophenone (117 µL, 120.15 mg, 1 mmol) and benzyl alcohol (104 µL, 108 mg, 1 mmol) in 1:1 ratio was measured using a micropipette and added to the pressure tube. A suspension was observed. The tube was heated in a sand bath at 100 °C for 2 h. A sample was taken and analyzed by ^{1}H NMR in CDCl$_3$.

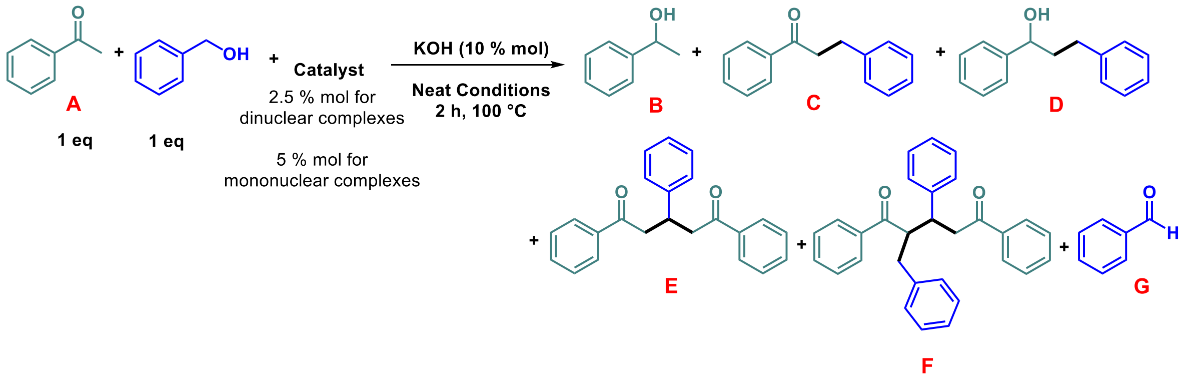

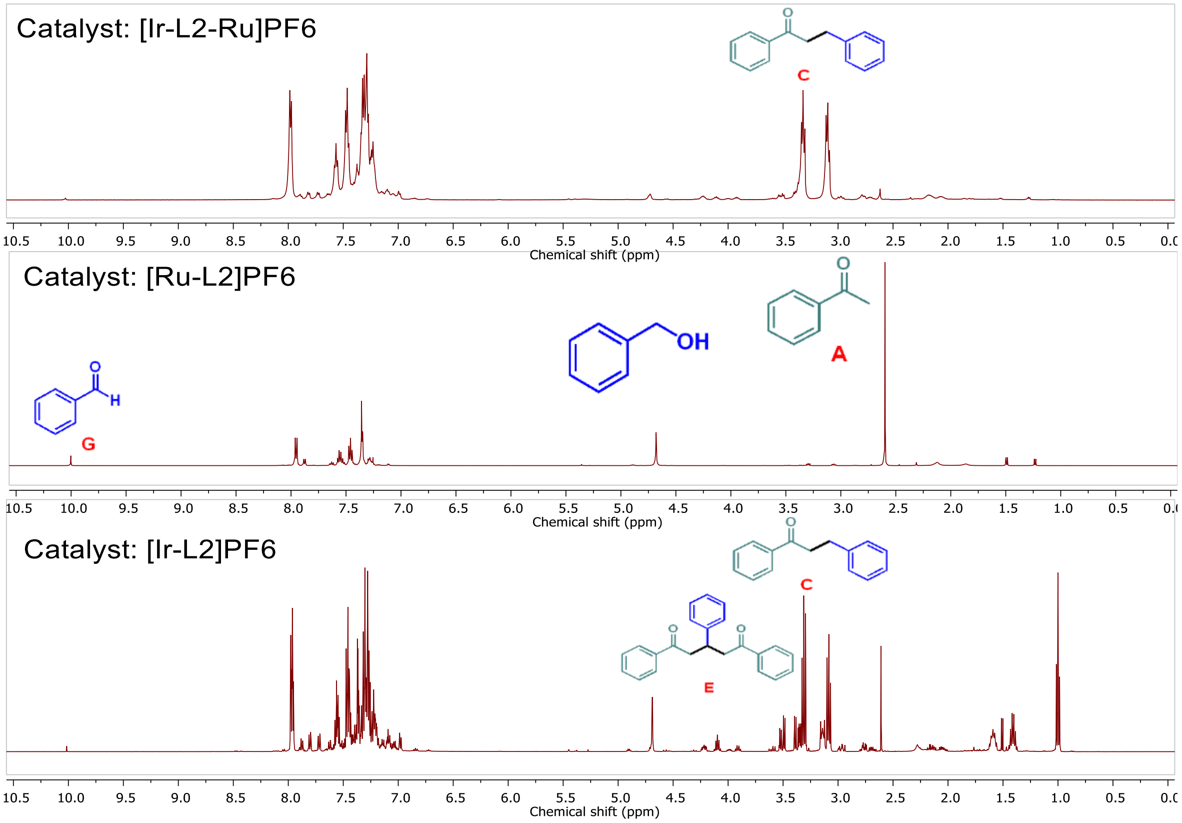

Figure S43 Stacked ^{1}H NMR spectra for the 1:1 ratio of acetophenone and benzyl alcohol reaction

Conversion of the [Ru-L²]PF₆ + [Ir-L²]PF₆ mixture to [Ir-L²-Ir]PF₆

In a pressure tube **[Ru-L²]PF₆** (20 mg, 0.3 mmol, 1 equiv), **[Ir-L²]PF₆** (23 mg, 0.3 mmol, 1 equiv), KOH (3.5 mg, 0.03 mmol 10%mol), acetophenone (10.9 µL, 0.09 mmol, 3 equiv) and benzyl alcohol (19.6 µL, 0.09 mmol, 3 equiv) were added and heated at 100°C for 1 h. After this time, diethyl ether was added and a solid precipitated. The analysis of the solid showed a mixture of **[Ir-L²]PF₆** and **[Ir-L²-Ir]PF₆**.

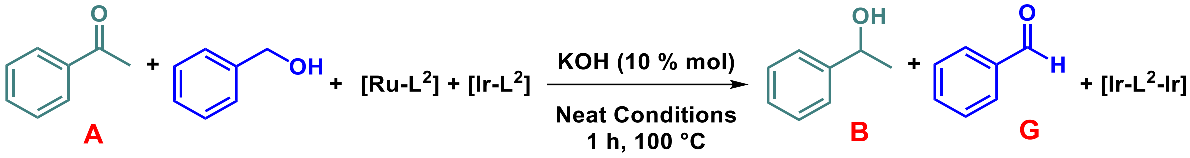

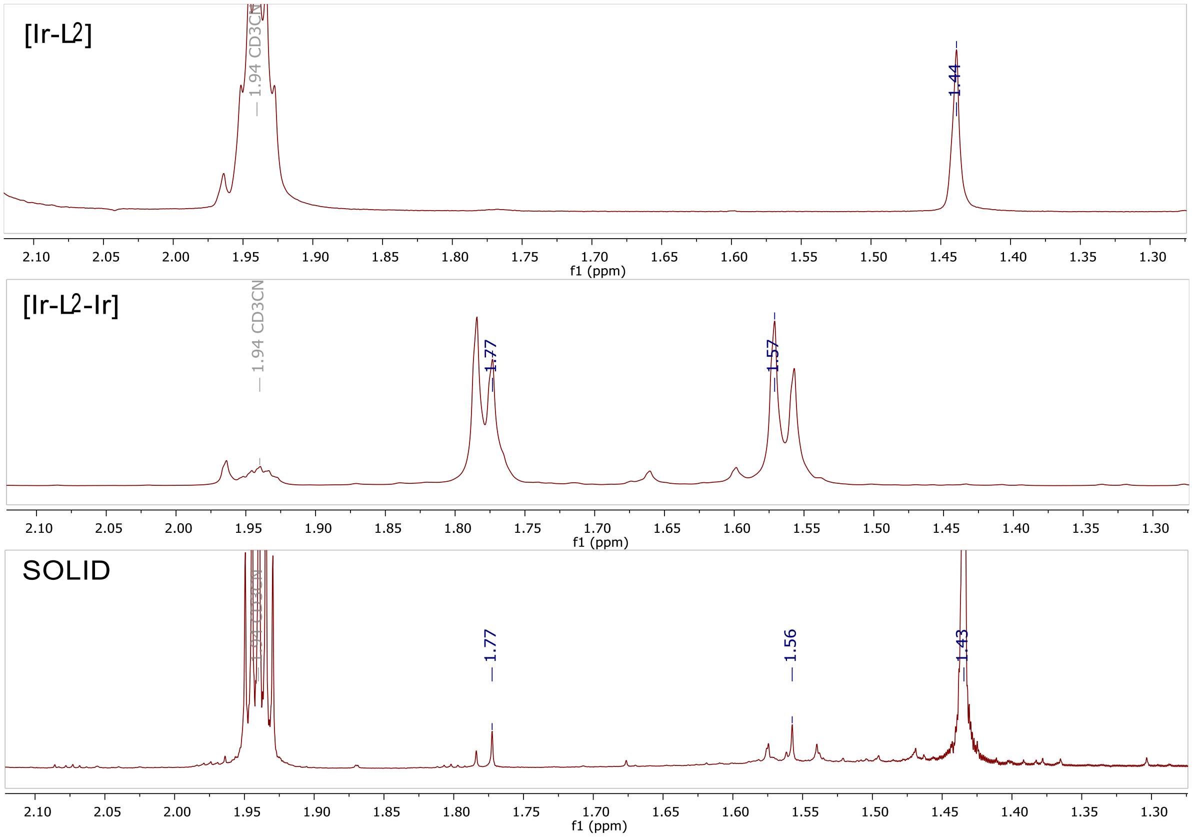

Figure S44 Stacked ¹H NMR spectra for the reaction between **[Ru-L²]PF₆** and **[Ir-L²]PF₆** producing **[Ir-L²-Ir]PF₆**

Cooperative index (a)[49]

$$A_P = \sum_{i=1}^{n} A_i \quad \textbf{(Eq. S3)}$$

$$\bar{A} = \frac{A_P}{n} \quad \textbf{(Eq. S4)}$$

$$a = \frac{A_O - A_P}{\bar{A}} \quad \textbf{(Eq. S5)}$$

A_P= predicted total activity of a polymetallic catalyst

A_i = measured activity of the monometallic complex that most closely mimics the steric and electronic attributes of the *i*th metal center

$\bar{A}$ = average activity

n = metal centers

A_O = observed activity of the polymetallic complex

a = cooperativity index

To our system:

For **[Ru-L^2-Ir]PF$_6$**

A_O = % yield of **D** of **[Ru-L^2-Ir]PF$_6$**

A_P= % yield of **[Ru-L^2]PF$_6$** + % yield of **[Ir-L$^{3/4}$]PF$_6$**

$$\bar{A} = \frac{14}{2} = 7 \quad \textbf{(Eq. S6)}$$

$$a = \frac{28-14}{7} = \mathbf{2} \quad \textbf{(Eq. S7)}$$

For **[Ir-L^2-Ir]PF$_6$**

A_O = % yield of **D** of **[Ir-L^2-Ir]PF$_6$**

A_P= % yield of **D** of **[Ir-L^2]PF$_6$** + % yield of **D** of **[Ir-L$^{3/4}$]PF$_6$**

$$\bar{A} = \frac{16}{2} = 8 \quad \textbf{(Eq. S8)}$$

$$a = \frac{0-16}{8} = \mathbf{-2} \quad \textbf{(Eq. S9)}$$

Scope of ketones and alcohols

For testing the scope of different ketones and alcohols, the standard procedure was utilized as described above, using **[Ir-L^2-Ru]PF$_6$** as catalyst in all reactions. The aliquots were analyzed by ^{1}H NMR. In some cases, the crude reaction mixture was purified by flash column chromatography on silica gel using hexane/ethyl acetate (10:0.5) as eluent to afford the corresponding products.

Analysis of the catalytic reaction scope

To study the catalytic reaction scope and the electronic effect of substituents at either the ketone or alcohol units, a variety of substrates were evaluated under optimal conditions using **[Ir-L^2-Ru]PF$_6$** as precatalyst, Table S5. We found that aryl ketones bearing *para* electron donating substituents can provide product **C**, however the yield for product **D** varies depending on the nature of the substituent. In the case of the *p*-methyl group (Table S5, entry 1) the yield for **D** is like the parent system (21 %), but for the *p*-amino group the yield drops to 7% (Table S5, entry 2). The latter can be explained by further reactivity of product **C**, which condensates with the benzaldehyde **G** to produce an imine moiety that is hydrogenated by the catalytic system to produce the corresponding secondary amine, 1-(4-(benzylamino)phenyl)-3-phenylpropan-1-one in 40 % yield, corroborated by ^{1}H NMR and MS (Figures S40 and S41). On the other hand, moderate conversions were observed for electron withdrawing substituents, 69-73% (Table S5, entries 3-4). The effect of *ortho*-OH electro donating substituent (Table S5, entry 5) was assessed, showing moderate conversion possibly due to steric hindrance and/or the electrodonating effect of the OH to the carbonyl group that diminishes the acidity and electrophilicity of the acetyl group.

Maintaining the unsubstituted acetophenone and varying the substituents at the benzyl alcohol unit, it is possible to observe that the electrodonating *p*-OMe group shows better conversions than the electrowithdrawing *p*-NO_2 group, 82 and 53 % respectively (Table S5, entries 6-7). Finally, piperonyl alcohol, which is a biologically relevant substrate, provided good conversion (81 %) along with 35% and 25% yield for compounds **C** and **D** respectively (Table S5, entry 8).

In both cases, acetophenone and benzyl alcohol substrates bearing electron-donating substituents at para positions allow a slight increase in the yield of **C** and **D**, due to further stabilization of the enolate, and the facilitated dehydrogenation of the alcohol to produce the corresponding benzaldehyde.

Table S5 Scope of ketones and alcohols.[a]

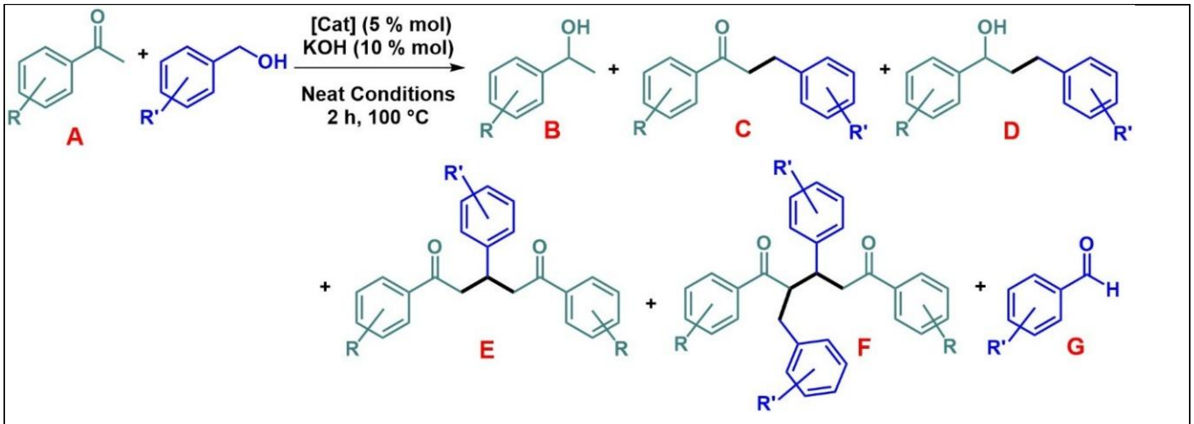

Entry	Ketone<br>R	Alcohol<br>R'	^{1}H NMR Conv **A** %	^{1}H NMR Yield %<br>**B**	**C**	**D**	**E**	**F**	**G**[b]
1	R = 4-Me	R' = H	97	3	65(57)[d]	21(16)[d]	5	3	12
2[c]	R = 4-NH_2	R' = H	66	3	12	7	0	0	6
3	R = 4-I	R' = H	69	16	37	5	7	3	7
4	R = 4-Br	R' = H	73	13	45	11	3	1	4
5	R = 2-OH	R' = H	54	5	47	0	2	0	6
6	R = H	R' = 4-OMe	82	21	62(47)[d]	11(7)[d]	1	0	16
7	R = H	R' = 4-NO_2	53	2	46	0	2	0	6
8	R = H	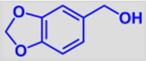 Piperonyl alcohol	81	15	35	25	2	1	20

[a] Reaction conditions: acetophenone (1 mmol), benzyl alcohol (3 mmol), base (10% mol), **[Ir-L^2-Ru]PF$_6$** as catalyst (2.5 % mol), 100°C for 2 h and neat conditions. Conversion and yield measured by ^{1}H NMR spectra analysis. The results are the average of at least two independent runs.
[b] Benzaldehyde yield was measured based on benzyl alcohol.
[c] Byproducts were observed, the condensation of **C** with **G** produced a new compound in 40% yield
[d] Isolated yield in parenthesis.

Byproduct observed in Table S5 Entry 2:

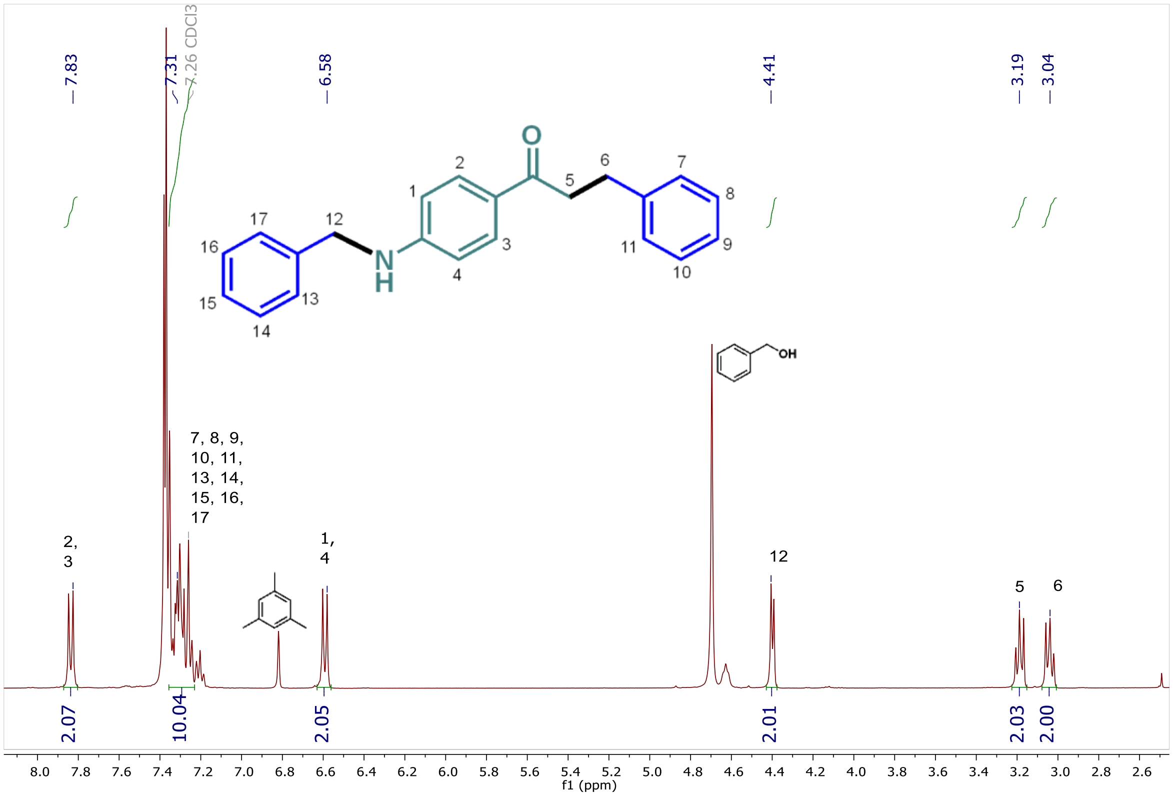

Figure S45 ^{1}H NMR spectrum for byproduct of Table S5 entry 2.

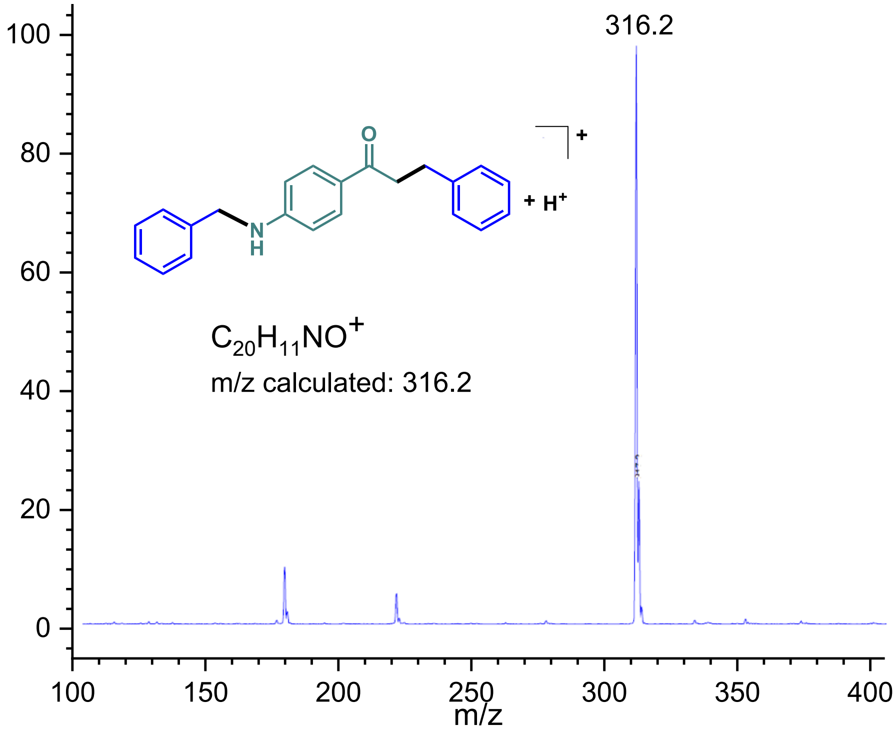

Figure S46 MS spectrum for byproduct of Table S5, entry 2.

Mercury test to probe homogeneous vs heterogeneous catalysis

A reaction under the standard methodology but adding one drop of Hg. The tube was sealed and heated at 100°C for 2 h. A sample was taken and ^{1}H NMR analyzed.

Control experiments and deuterium labeling studies.

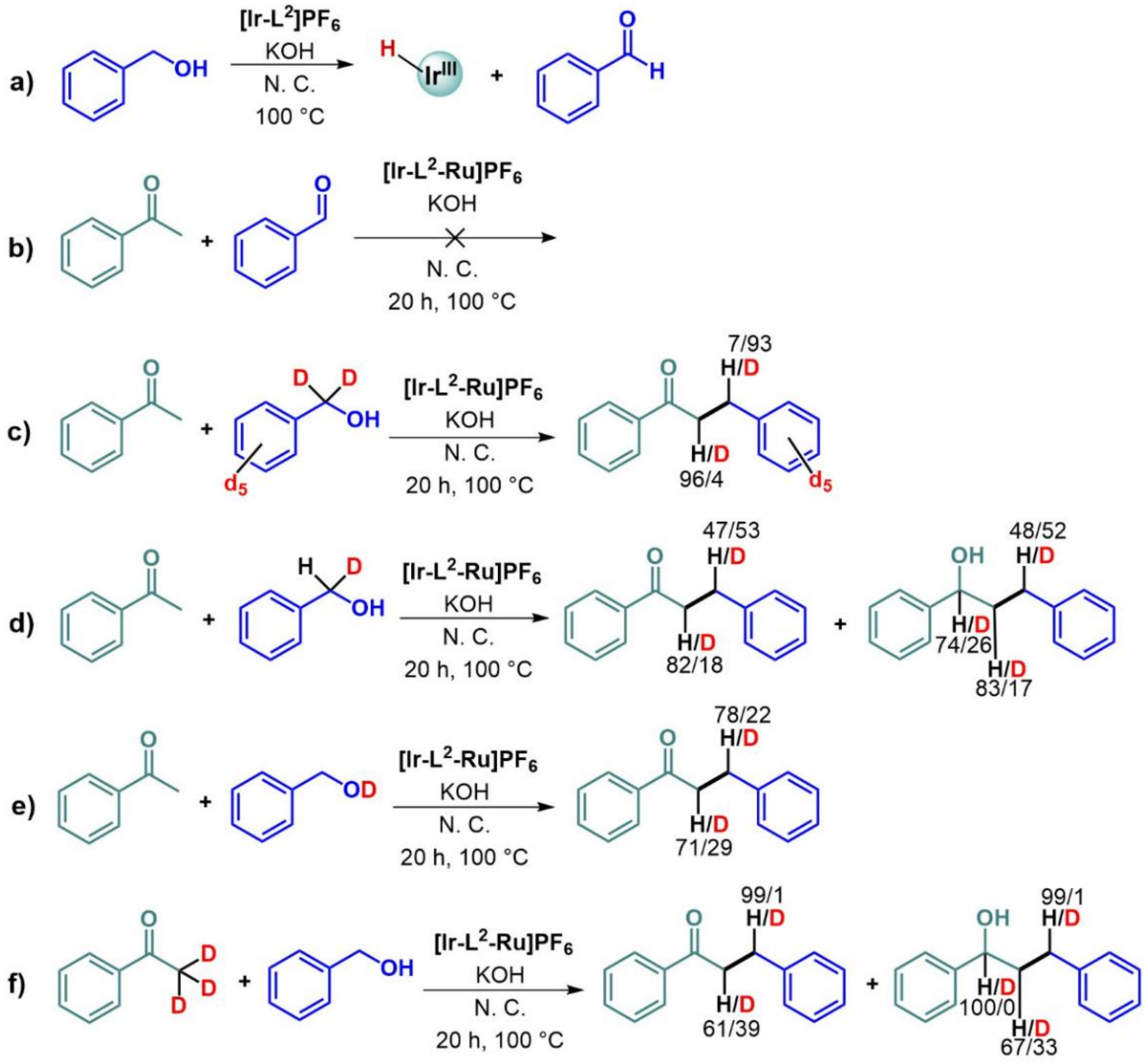

Scheme S3 Control experiments and deuterium labeling studies.

Hydride species

A solution of KOH (54.3 mg, 0.96 mmol) in benzyl alcohol was prepared in a 2 mL volumetric flask and diluted to the mark with benzyl alcohol. In a J. Young NMR tube, **[Ir-L^{2}]PF$_6$** (10 mg, 0.0136 mmol) and 2.8 µL of KOH solution (corresponding to 10 % mol KOH and 2 equiv of benzyl alcohol) were added using a micropipette. Acetonitrile-*d$_3$* (0.3 mL) was used as the deuterated solvent. The mixture was sonicated then the tube was loaded in a 500 MHz NMR

instrument and heated to 75°C. Spectra were recorded every 5 minutes for 1 hour with 64 scans per acquisition.

The hydride species was detected after 5 minutes of reaction, with its signal gradually increasing and reaching a maximum at 45 minutes. The intensity then remained constant until the end of the 1 h experiment.

The characteristic hydride signal was observed at δ = –13.93 ppm, and the benzaldehyde signal at δ = 10.04 ppm. Additionally, in the aromatic region, signals between δ = 8.42 – 8.86 (marked with an asterisk) were detected, suggesting the formation of new species that may correspond to the remaining fragment of the molecule bearing the hydride. It has not been possible to fully characterize the hydride species so far, likely due to its transient nature and short lifetime under the reaction conditions.

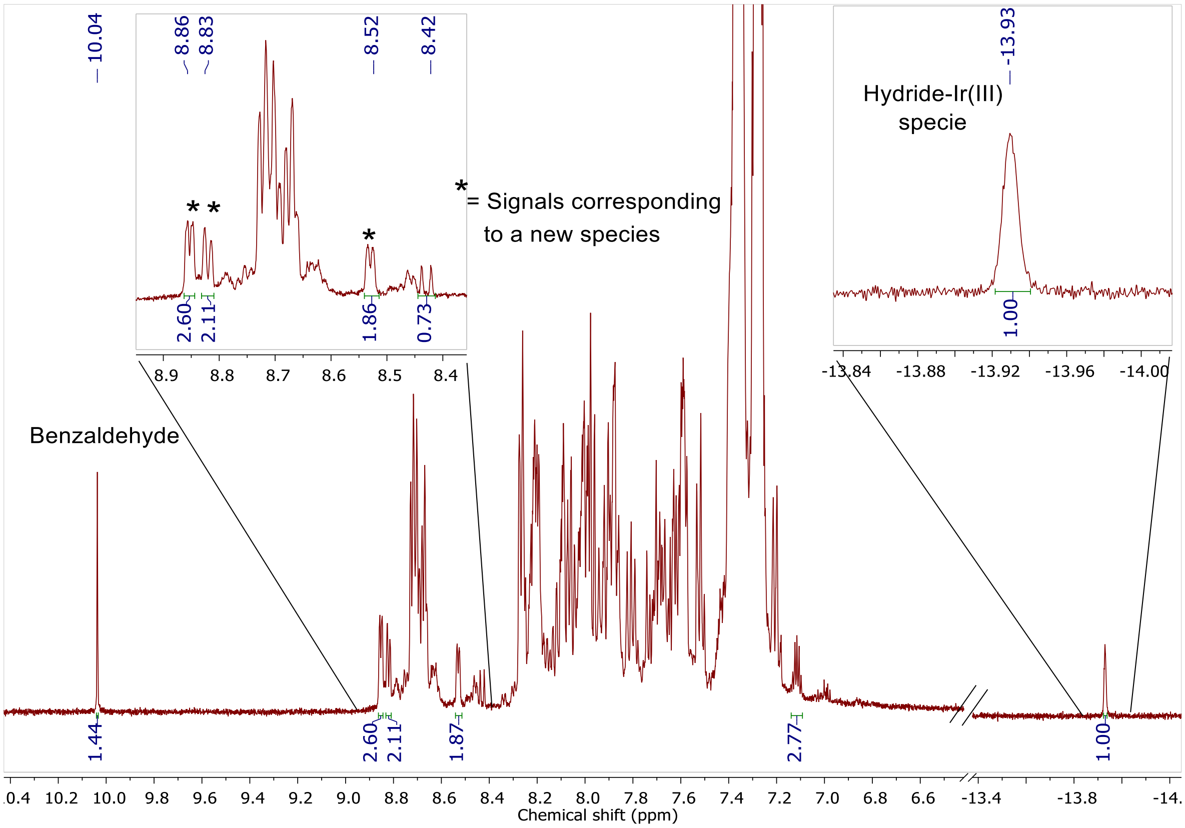

Figure S47 ^{1}H NMR spectrum for the reaction between **[Ir-L^{2}]PF$_{6}$**, benzyl alcohol and KOH at 75 °C to detect the hydride species in acetonitrile-d_3

Benzaldehyde instead of benzyl alcohol

A reaction under the standard methodology but adding benzaldehyde (306 µL, 3 equiv, 3 mmol) instead of benzyl alcohol was performed. A sample was taken at 2 h and 20 h and ^{1}H NMR analyzed.

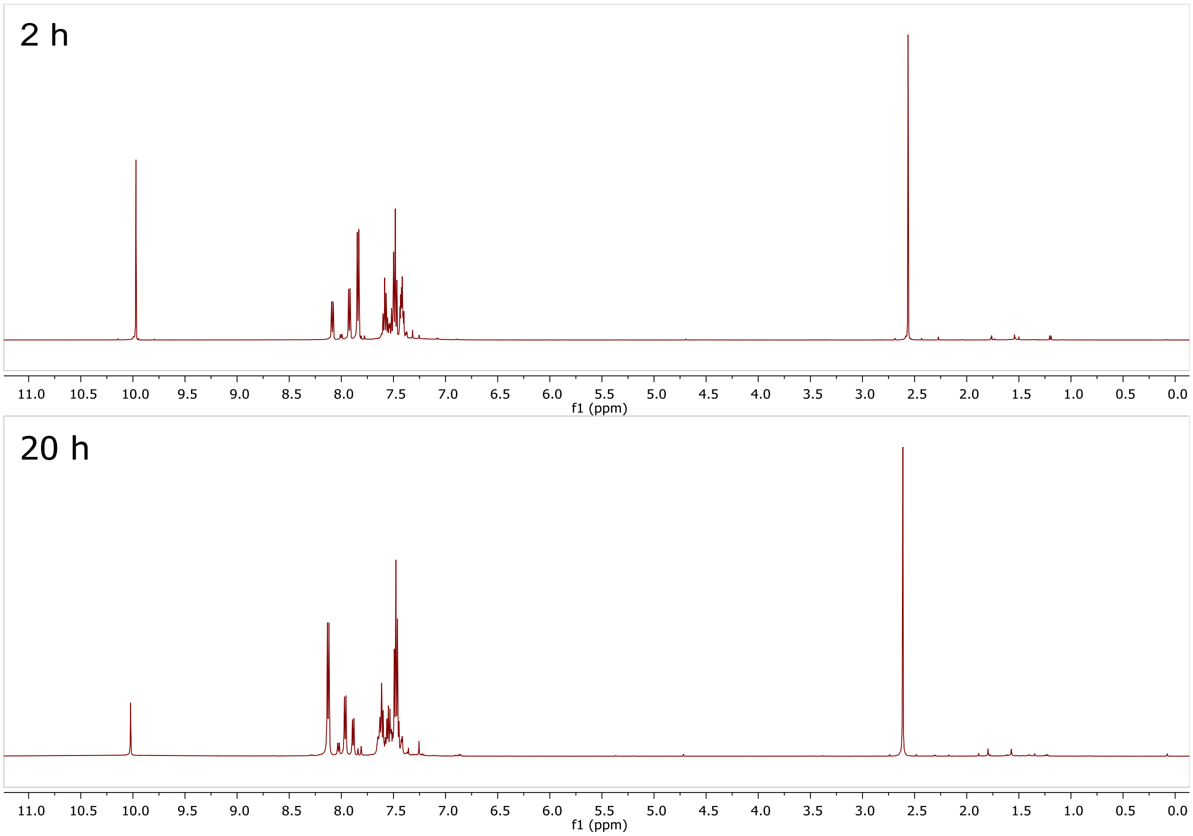

Figure S48 Stacked ^{1}H NMR spectra for the catalysis reaction using benzaldehyde instead of benzyl alcohol at 2 h and 20 h.

Deuterated experiments

The reactions were performed under standard optimized conditions as described above, using **[Ir-L²-Ru]PF₆** as catalyst in all reactions, for 20 h. The crude reaction mixture was purified by flash column chromatography on silica gel using hexane/ethyl acetate (10:0.5) as eluent to afford the corresponding products.

Calculating deuterium incorporation in products

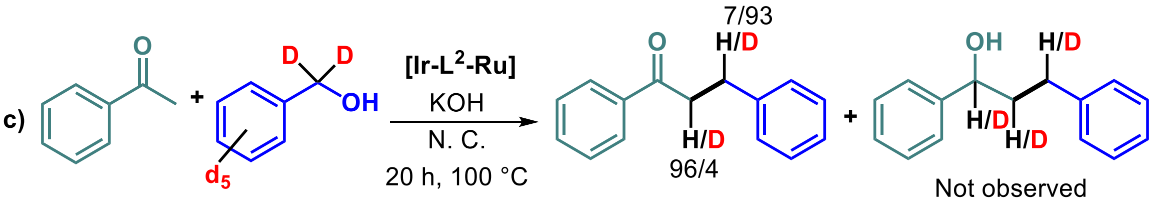

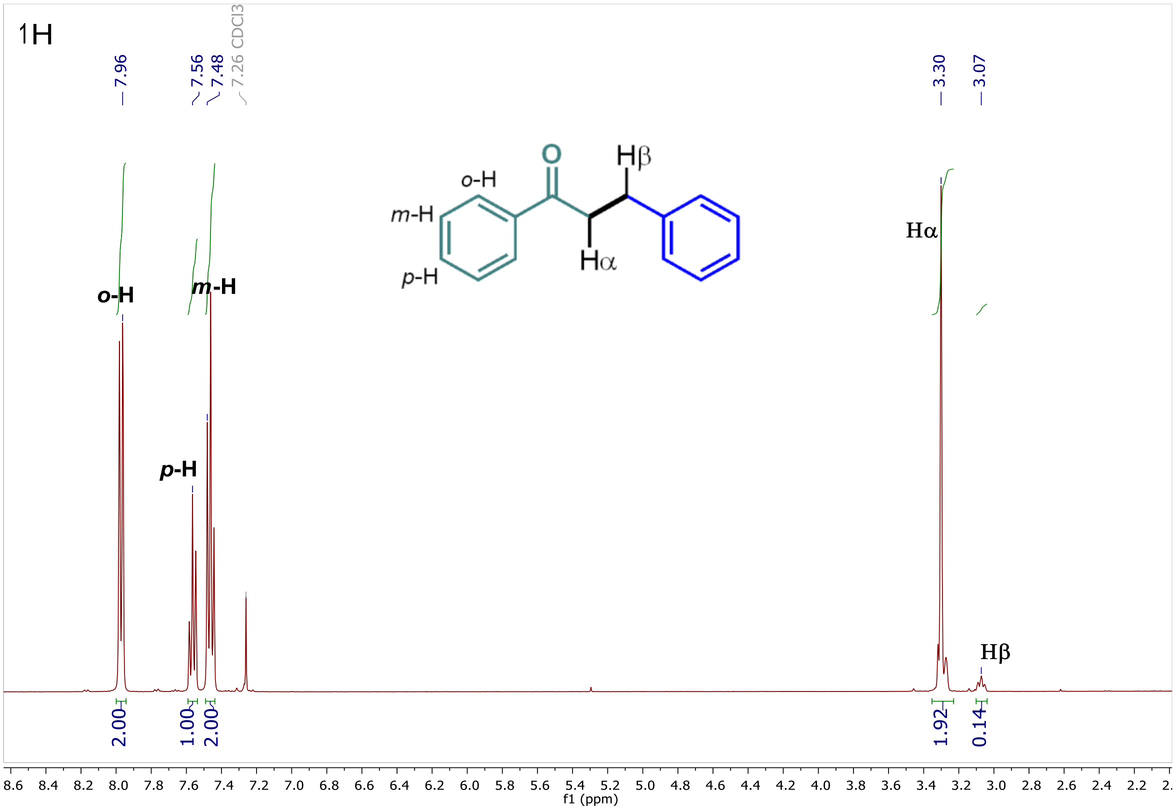

Figure S49 ^{1}H NMR spectrum for the corresponding alkylated product of reaction **c**, Scheme S3.

Deuterium incorporation calculation for alkylation product of reaction **c**, Scheme S3.

	Deuterium incorporation in **α** position	Deuterium incorporation in **b** position
Integral Value	1.92	0.14
Calculated ratio	[(2-1.92) / 2] * 100 = 4%	[(2-0.14) / 2] * 100 = 93%

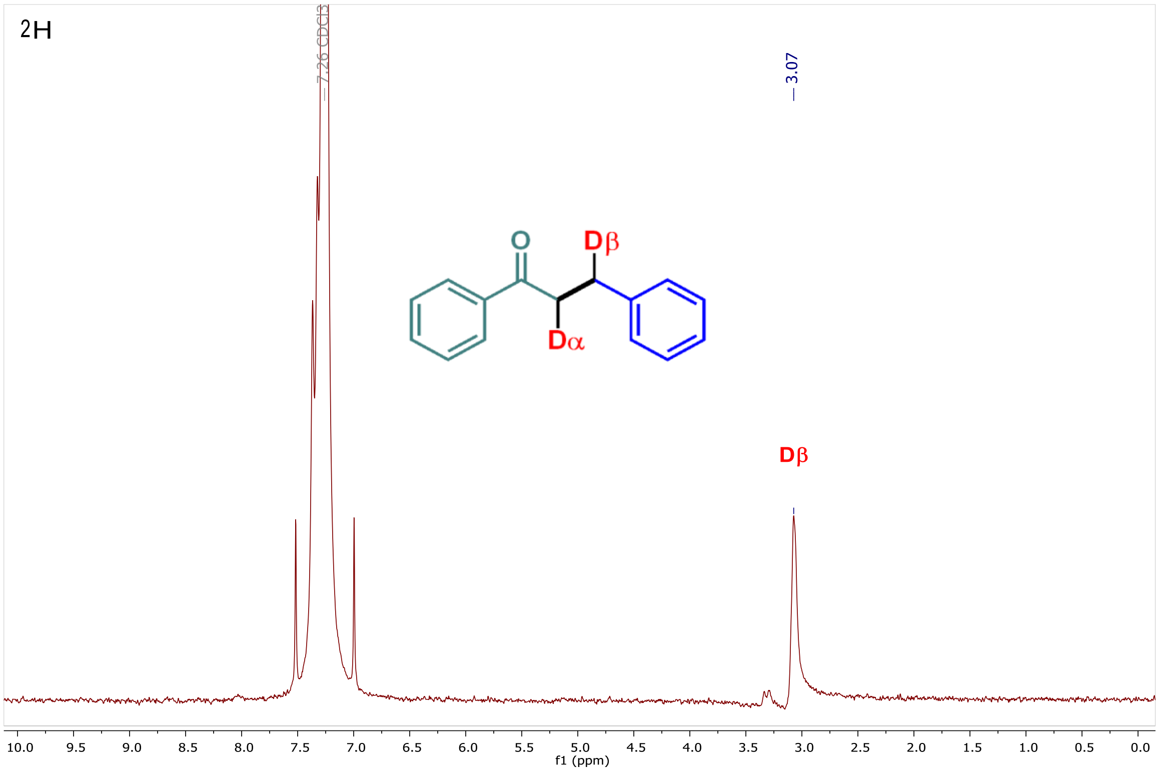

Figure S50 ^{2}H NMR spectrum for the corresponding alkylated product of reaction **c**, Scheme S3.

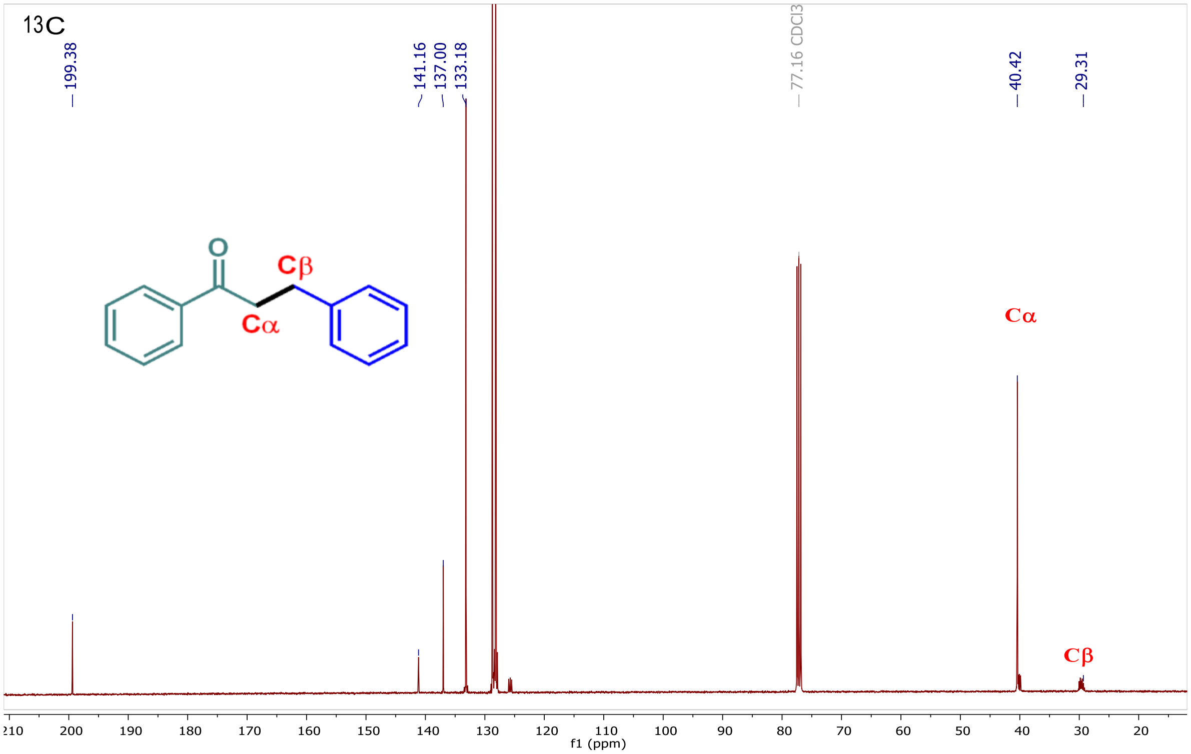

Figure S51 ^{13}C NMR spectrum for the corresponding alkylated product of reaction **c**, Scheme S3.

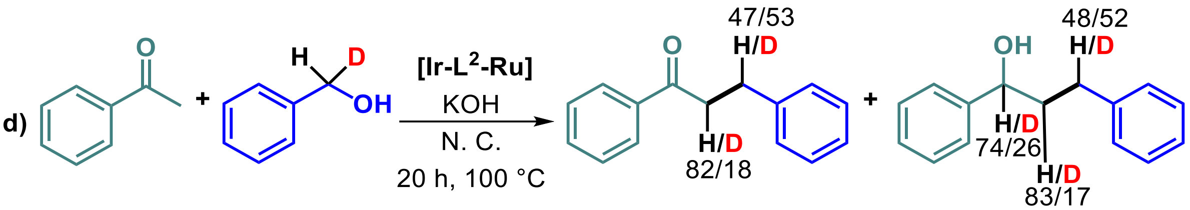

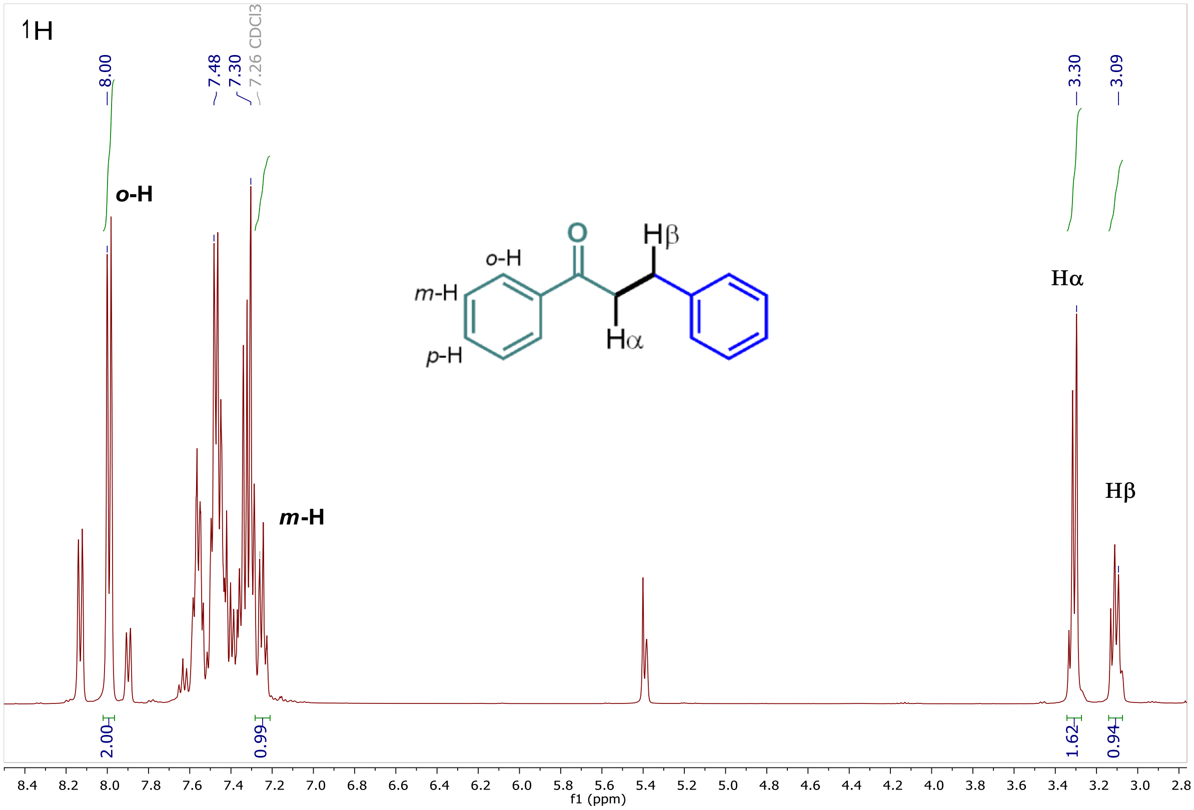

Figure S52 ¹H NMR spectrum for the corresponding alkylated product of reaction **d**, Scheme S3.

Deuterium incorporation calculation for alkylation product of reaction **d**, Scheme S3

	Deuterium incorporation in **α** position	Deuterium incorporation in **b** position
Integral Value	1.62	0.94
Calculated ratio	[(2-1.62) / 2] * 100 = 18%	[(2-0.94) / 2] * 100 = 53%

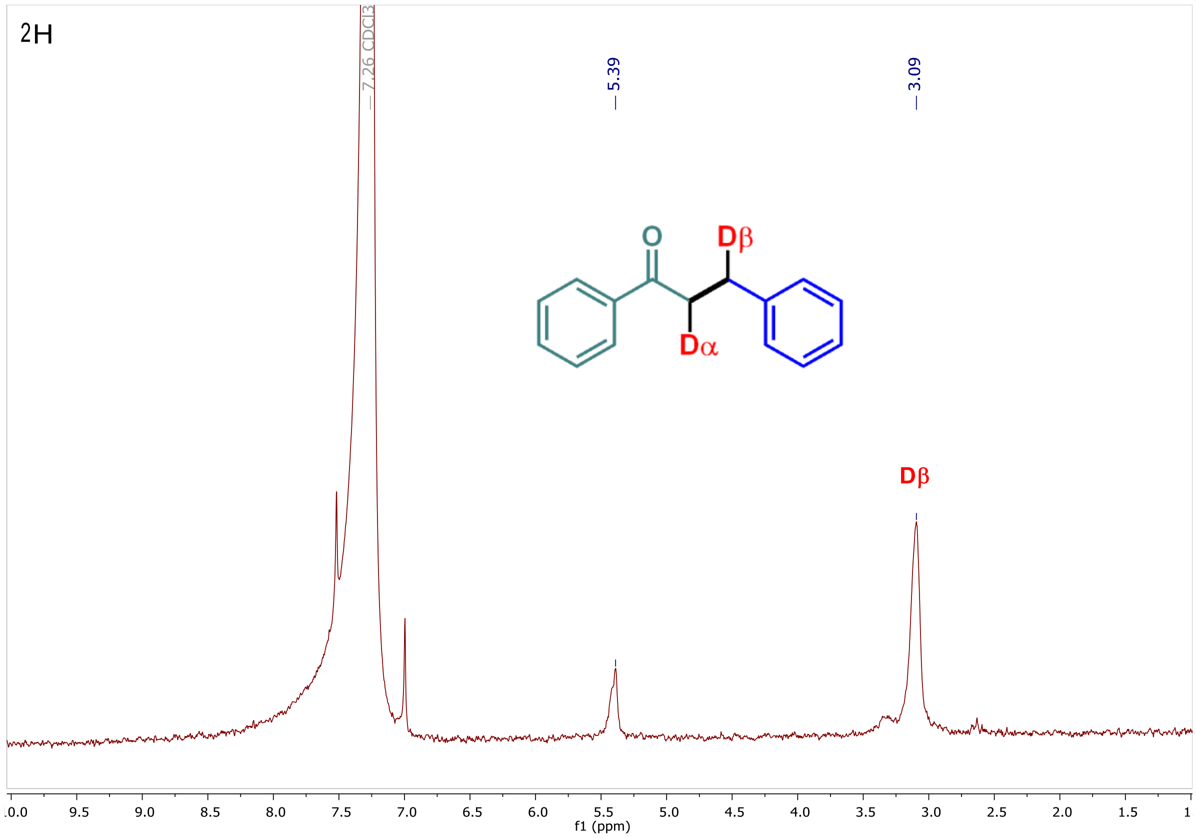

Figure S53 ^{2}H NMR spectrum for the corresponding alkylated product of reaction **d**, Scheme S3.

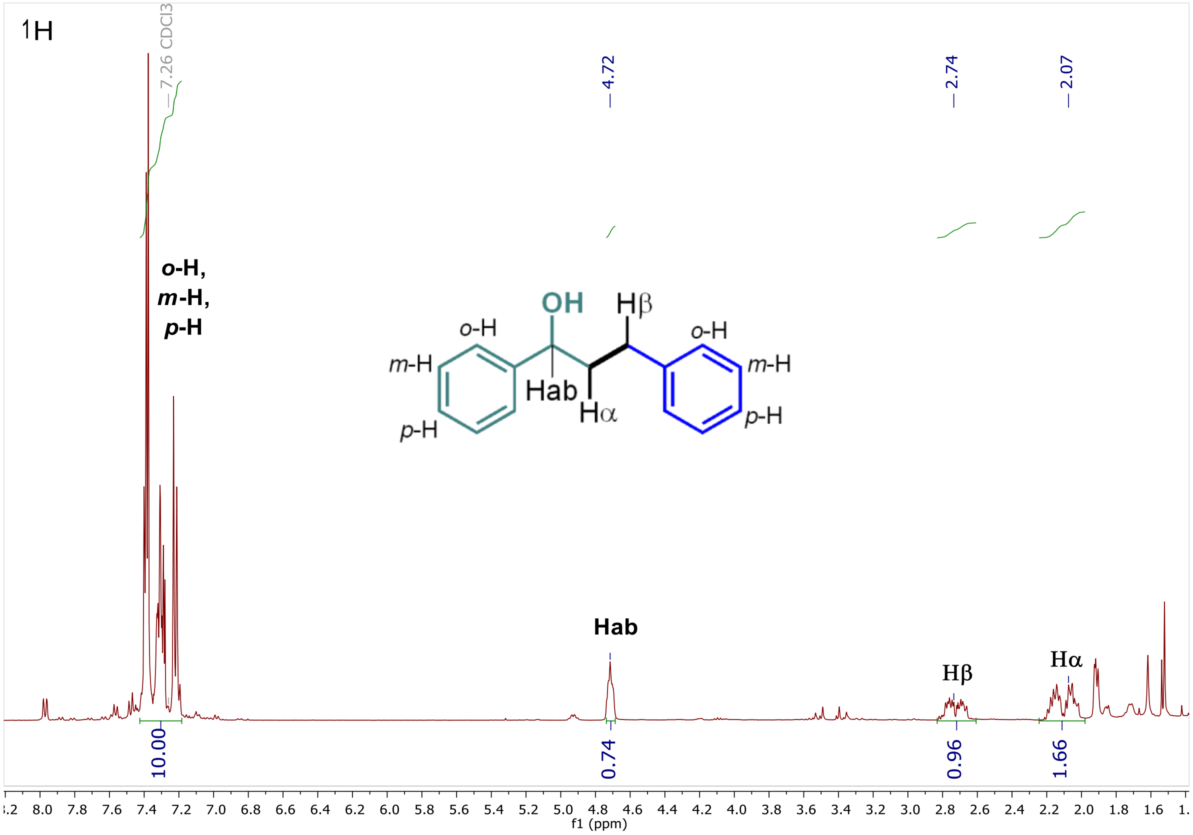

Figure S54 ^{1}H NMR spectrum for the corresponding tandem product of reaction **d**, Scheme S3.

Deuterium incorporation calculation for alkylation product of reaction **d**, Scheme S3

	Deuterium incorporation in **CH** position	Deuterium incorporation in **α** position	Deuterium incorporation in **β** position
Integral Value	0.74	1.66	0.96
Calculated ratio	[1 - (0.74)]*100 = 26%	[(2-1.66)/2]*100 = 17%	[(2-0.96)/2]*100 = 52%

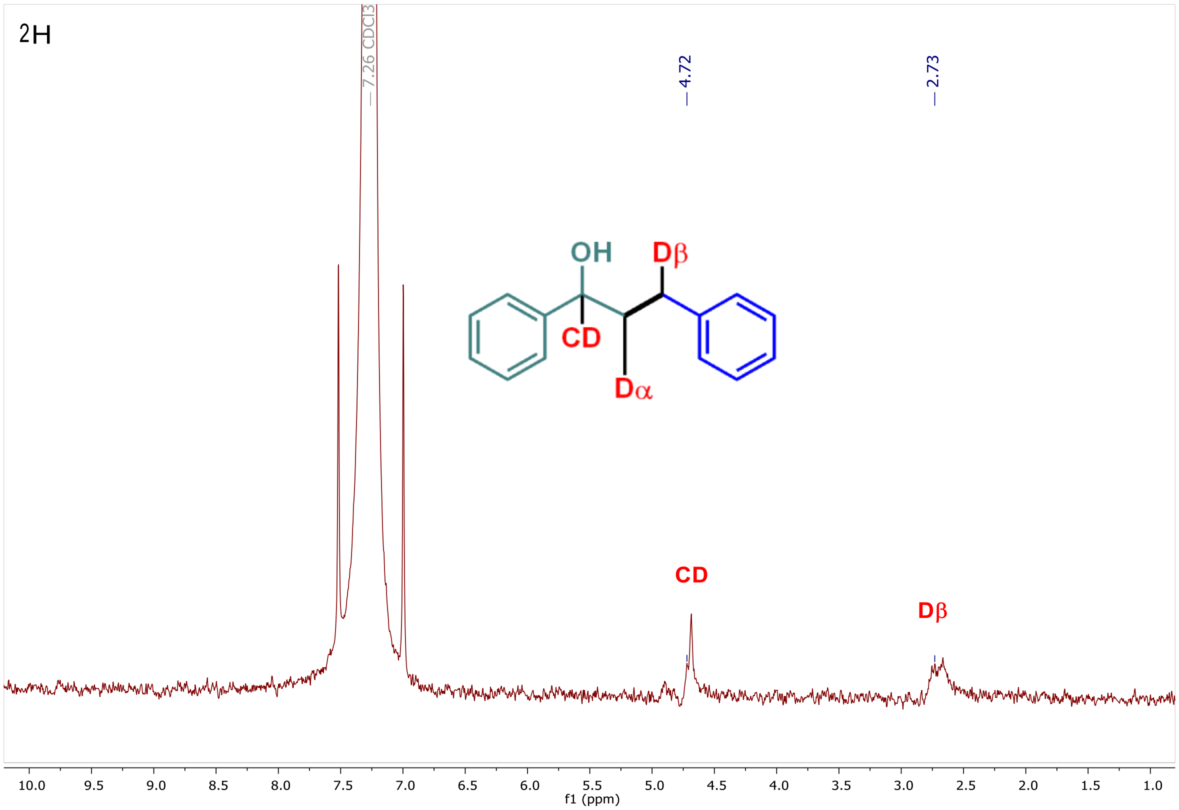

Figure S55 ^{2}H NMR spectrum for the corresponding tandem product of reaction **d**, Scheme S3.

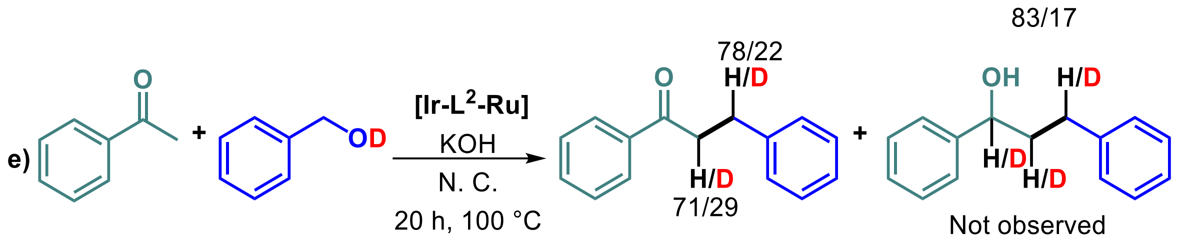

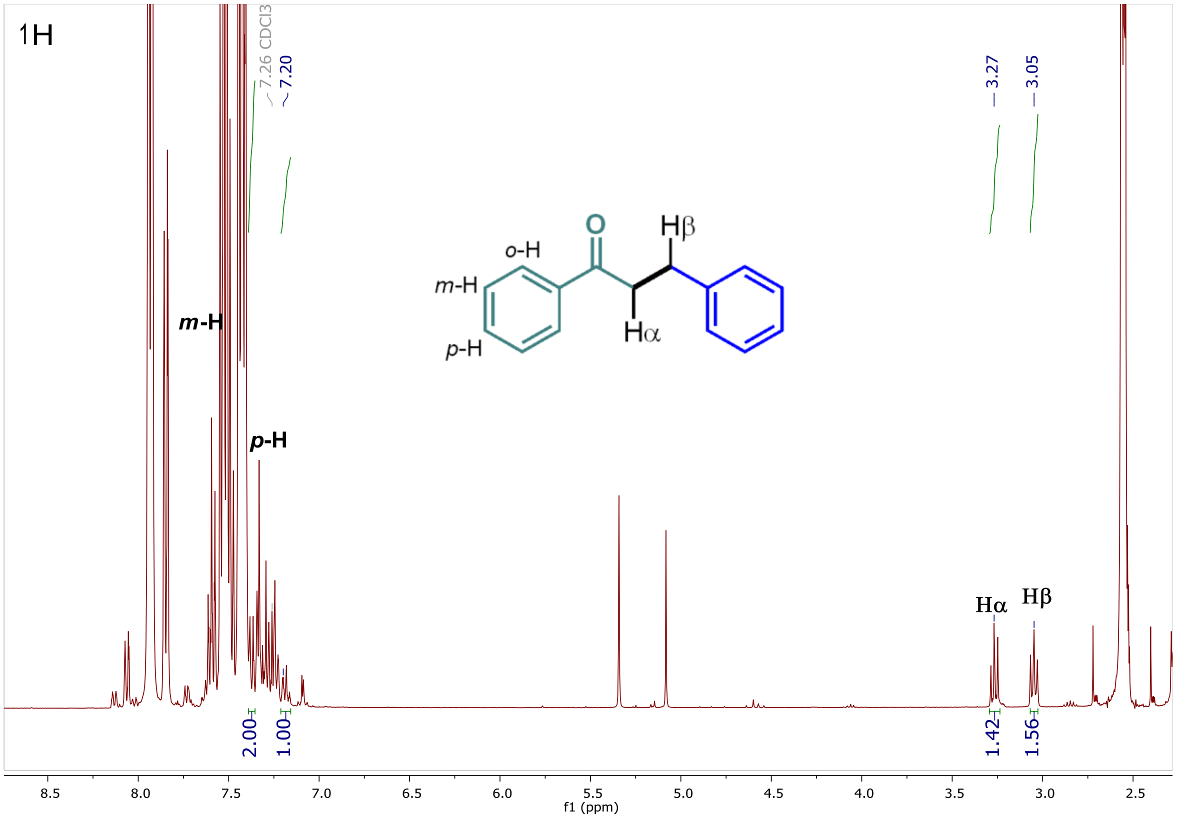

Figure S56 ^{1}H NMR spectrum for the corresponding alkylated product of reaction **e**, Scheme S3.

Deuterium incorporation calculation for alkylation product of reaction **e**, Scheme S3

	Deuterium incorporation in **α** position	Deuterium incorporation in **b** position
Integral Value	1.42	1.56
Calculated ratio	[(2-1.42) / 2] * 100 = 29%	[(2-1.56) / 2] * 100 = 22%

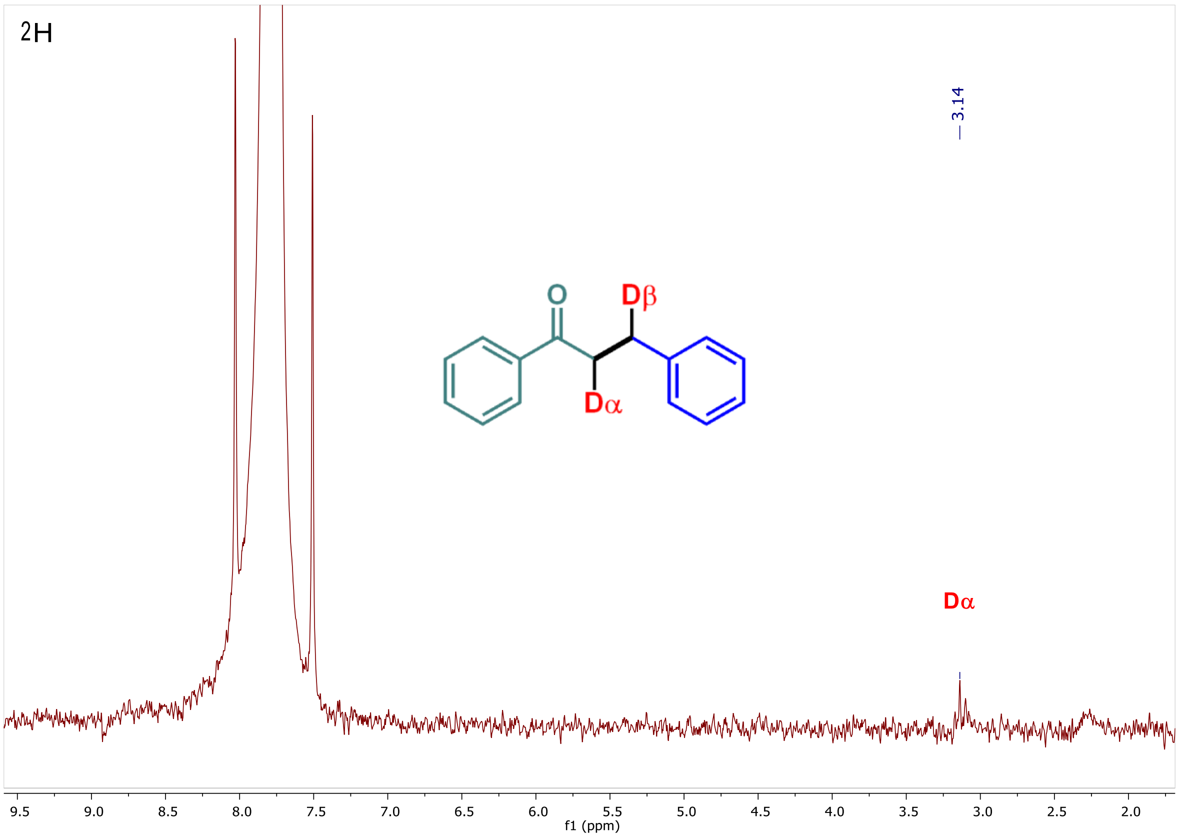

Figure S57 ^{2}H NMR spectrum for the corresponding alkylated product of reaction **e**, Scheme S3.

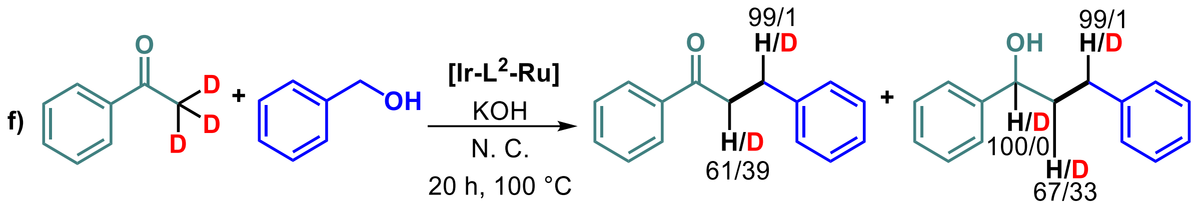

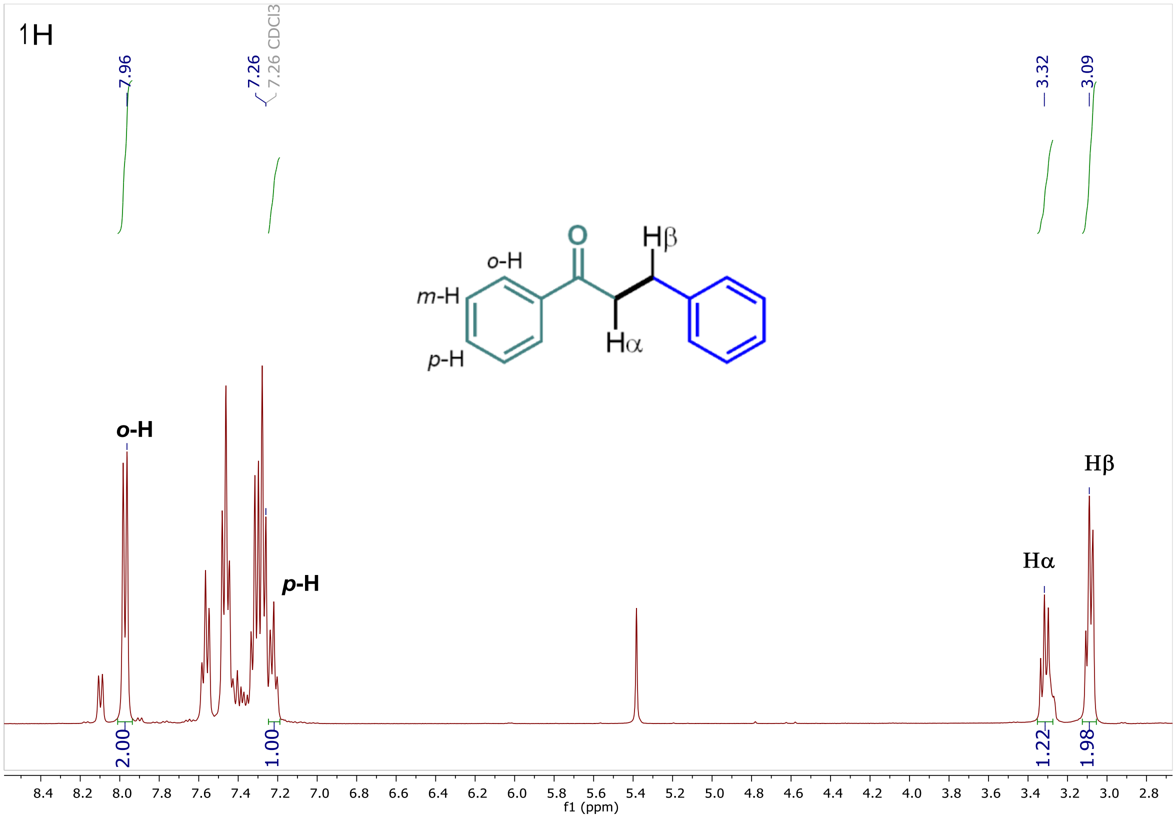

Figure S58 ^{1}H NMR spectrum for the corresponding alkylated product of reaction **f**, Scheme S3.

Deuterium incorporation calculation for alkylation product of reaction **f**, Scheme S3.

	Deuterium incorporation in **α** position	Deuterium incorporation in **b** position
Integral Value	1.22	1.98
Calculated ratio	[(2-1.22) / 2] * 100 = 39%	[(2-1.98) / 2] * 100 = 1%

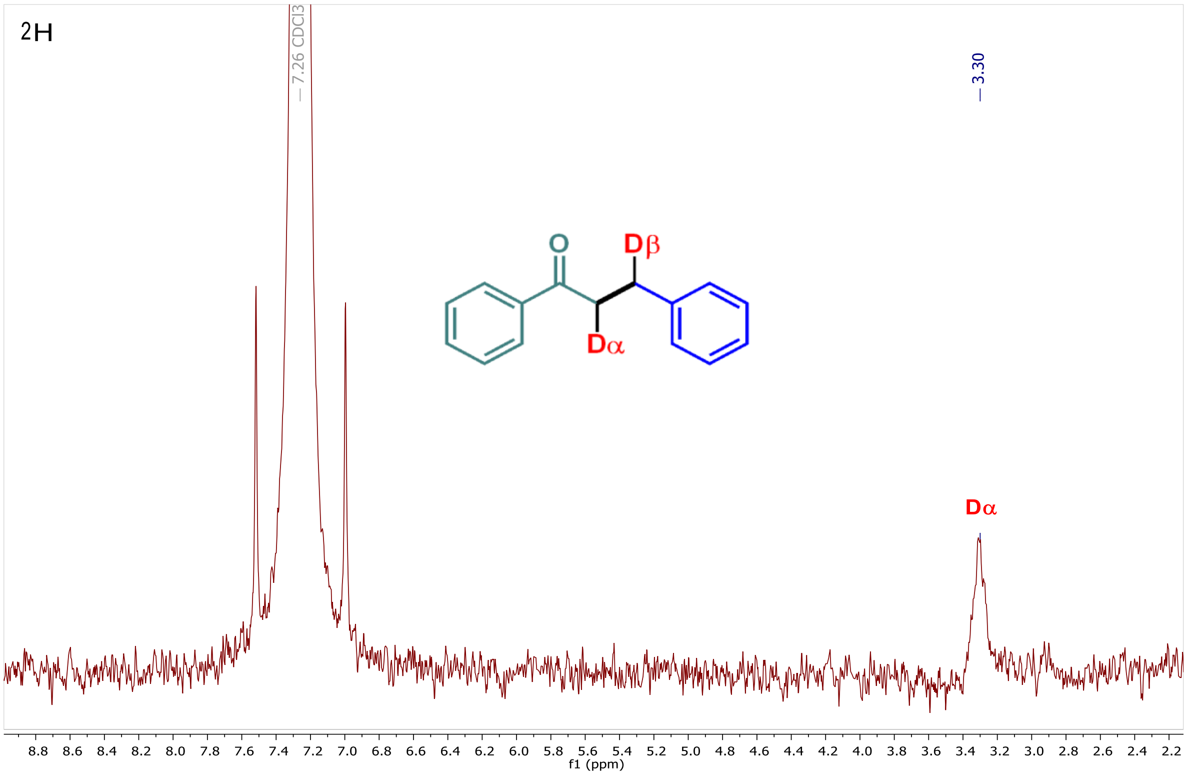

Figure S59 ^{2}H NMR spectrum for the corresponding alkylated product of reaction **f**, Scheme S3.

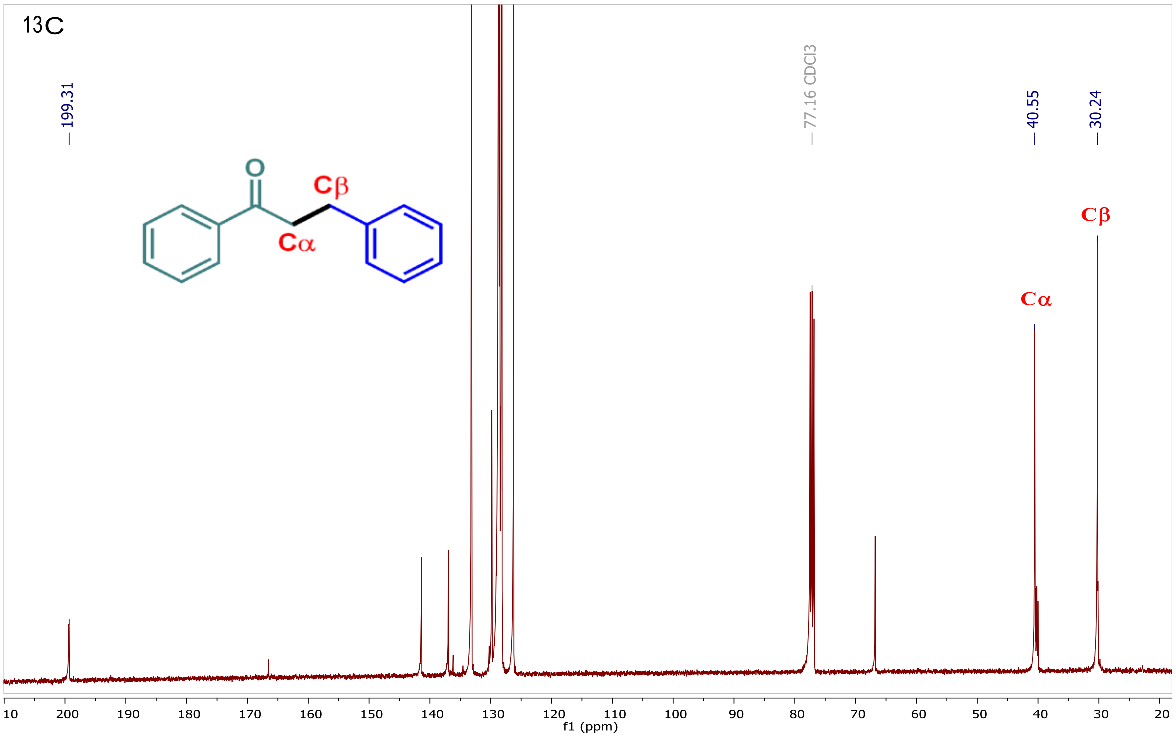

Figure S60 ^{13}C NMR spectrum for the corresponding alkylated product of reaction **f**, Scheme S3.

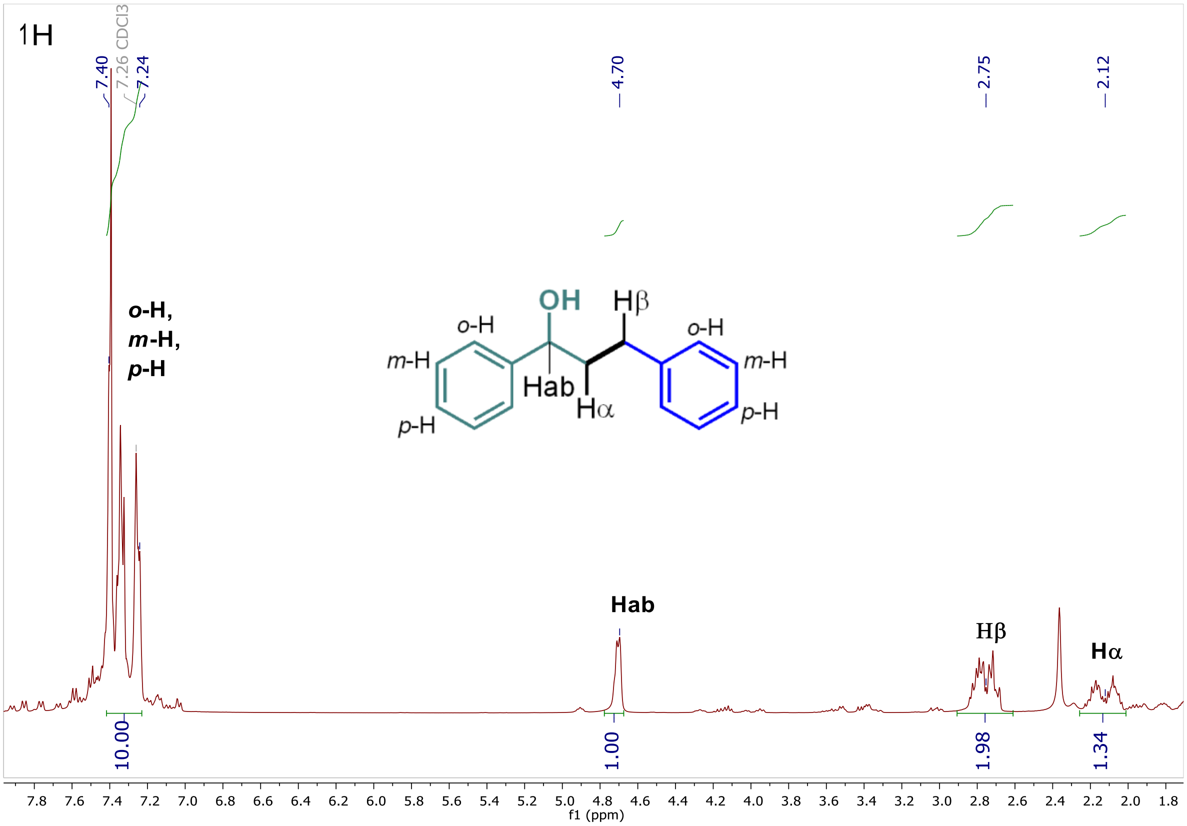

Figure S61 ^{1}H NMR spectrum for the corresponding tandem product of reaction **f**, Scheme S3.

Deuterium incorporation calculation for alkylation product of reaction **f**, Scheme S3.

	Deuterium incorporation in **Hab** position	Deuterium incorporation in **α** position	Deuterium incorporation in **β** position
Integral Value	1	1.34	1.98
Calculated ratio	[1 - (1)] * 100 =0%	[(2-1.34)/2]*100 = 33%	[(2-1.98)/2]*100 = 1%

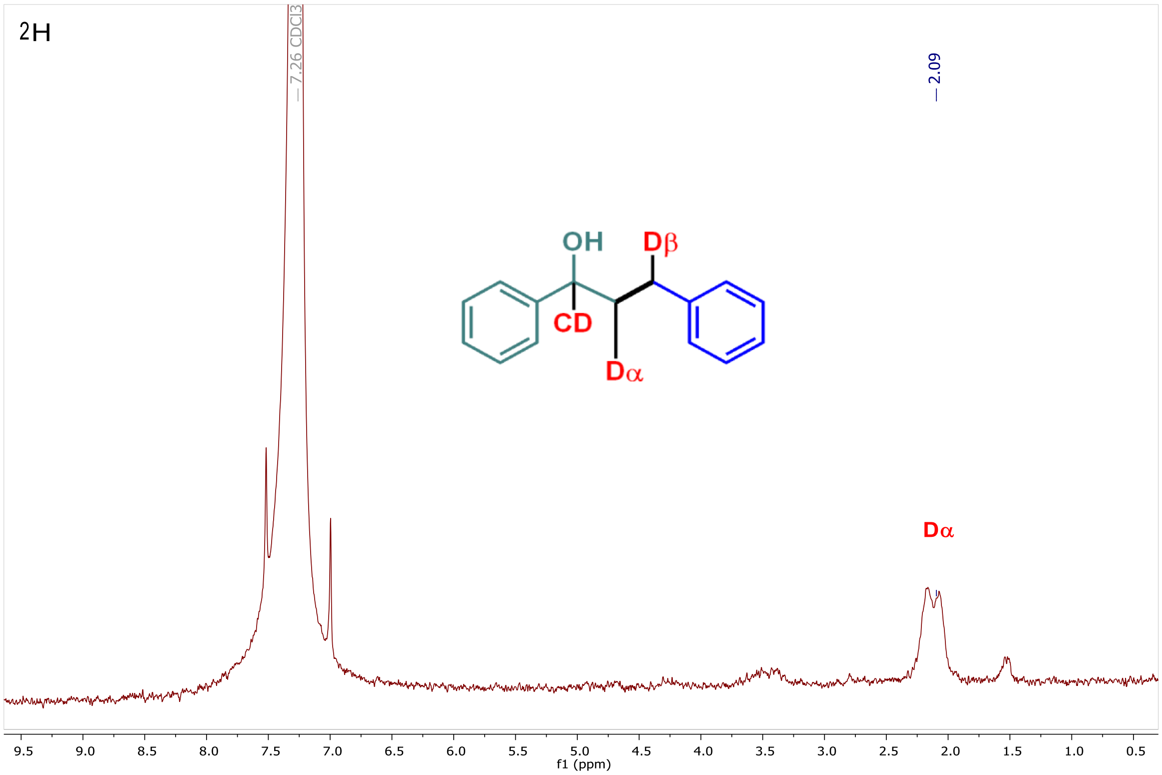

Figure S62 ^{2}H NMR spectrum for the corresponding tandem product of reaction **f**, Scheme S3.

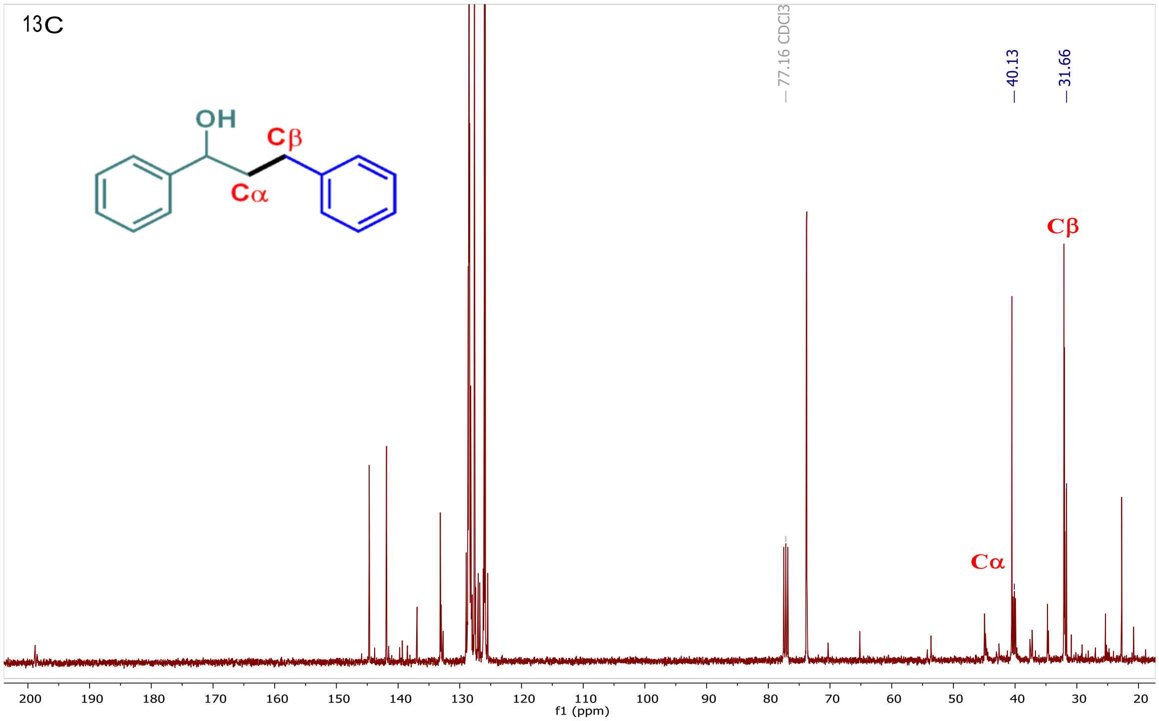

Figure S63 ^{13}C NMR spectrum for the corresponding tandem product of reaction **f**, Scheme S3.

Analysis of control experiments and deuterium labeling studies.

The general mechanism for the α-alkylation/transfer hydrogenation reaction has been established in previous reports.[37–40] In this work, the mechanistic studies were intended to confirm that our system follows the same overall mechanism described (Scheme S3).

Two key experiments were conducted. In the first, a mixture of benzyl alcohol, base, and **[Ir-L^2]PF$_6$** complex was heated at 100°C and monitored by ^{1}H NMR spectroscopy (Scheme S3a). After 5 minutes, signals corresponding to benzaldehyde (δ = 10.04 ppm) and a hydride-Ir(III) species (δ = -13.93 ppm) were observed (Figure S47). These chemical shifts are consistent with previously reported hydride-Ir(III) complexes.[39,50] Despite attempts, crystallization of the Ir-H species proved unsuccessful. Similar experiments with the **[Ru-L^2]PF$_6$** did not reveal a detectable Ru-H species, likely due to its inherent low stability or high reactivity.

The second experiment was performed to confirm the role of benzyl alcohol as the hydrogen source. The use of benzaldehyde in place of benzyl alcohol (Scheme S3b), resulted in no product formation, confirming the essential role of benzyl alcohol in the reaction (Figure S48).

To confirm the roles of acetophenone and benzyl alcohol in the tandem process, we conducted a series of deuterium labeling experiments under standard reaction conditions, Scheme S3, observing deuteration of the products in agreement with previously reported mechanism.[37–40]

Three deuterated benzyl alcohol substrates were synthesized and employed. First, using $C_6D_5CD_2OH$ (Scheme S3c) resulted exclusively in the formation of product **C**, with most of the deuterium incorporation observed at the β-position. This observation confirms that the alkylation originates from the alcohol substrate. Notably, product **D** was not detected; this is likely due to a kinetic isotope effect, which led to a decrease in the deuterium-transfer reaction rate.

Next, we utilized C_6H_5CHDOH (Scheme S3d), which allowed for the observation of tandem product **D**. A moderate isotopic effect of 1.1 was obtained for both **C** and **D** products, by analyzing the deuterium percentage at the β-position. On the other hand, it is evident that benzyl alcohol dehydrogenation produces the metal hydride species responsible for reduction of the carbonyl group of **C** to produce **D**, as 26 % of deuterium incorporation at the alcohol position in **D** was observed.

In a third experiment, $C_6H_5CH_2OD$ (Scheme S3e) was used. This resulted in 29% and 22% deuterium incorporation at the α and β positions, respectively in product **C**. Notably, tandem product **D** was not observed in this case.

Finally, we performed an experiment using unlabeled benzyl alcohol and $C_6H_5COCD_3$ (Scheme S3f). This experiment yielded the highest observed deuterium incorporation at the α-position, as expected considering the mechanism reported in previous studies.

Arrhenius analysis

Experimental procedure to determine the activation energy.

A KOH (5.4 mg, 0.096 mmol) solution in benzyl alcohol was prepared in a 1 mL volumetric flask and diluted to the mark with benzyl alcohol. In a J Young NMR tube the catalyst (5% mol for mononuclear and 2.5% mol for dinuclear complexes), acetophenone (33.7 μL, 0.29 mmol), mesitylene as internal standard (40.1 μL, 0.29 mmol) and 300 μL of KOH solution (0.029 mmol, 10% mol of KOH and 2.886 mmol, 10 equiv of benzyl alcohol)) were added using a micropipette, The mixture was sonicated and a coaxial tube containing DMSO-*d*$_6$ was added to the J Young NMR tube. The tube was loaded on the 400 MHz NMR instrument at temperatures in the 85° to 100°C range, in 5°C steps. A spectrum was taken with 16 scans every 5 minutes.

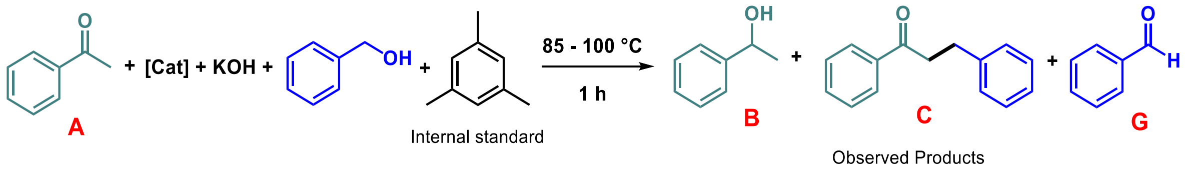

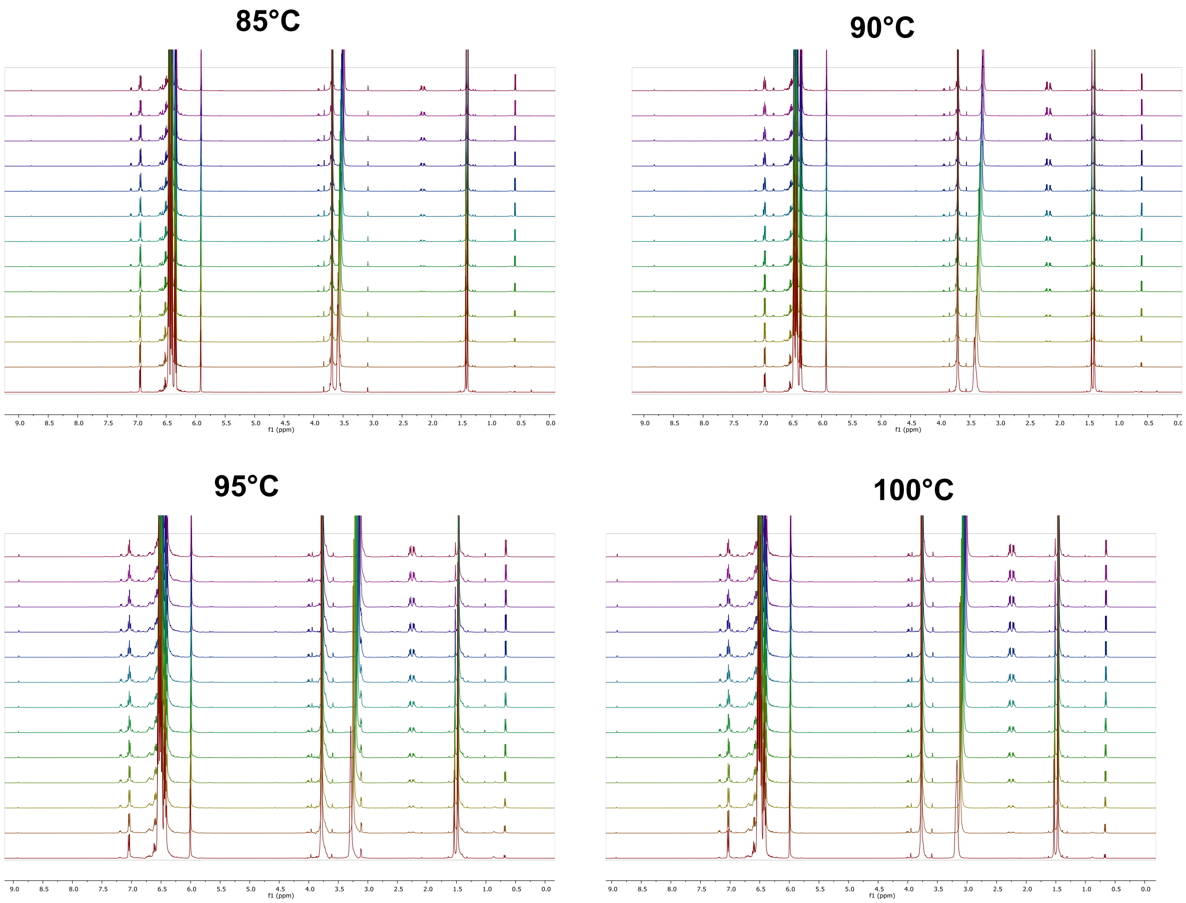

Figure S64 Stacked ^{1}H NMR spectra for kinetic experiments of **[Ir-L^2]PF$_6$** complex, only **B** (δ=0.66 ppm) and **C** (δ=2.28-2.30 ppm) products were observed over 1 hour at varying temperatures.

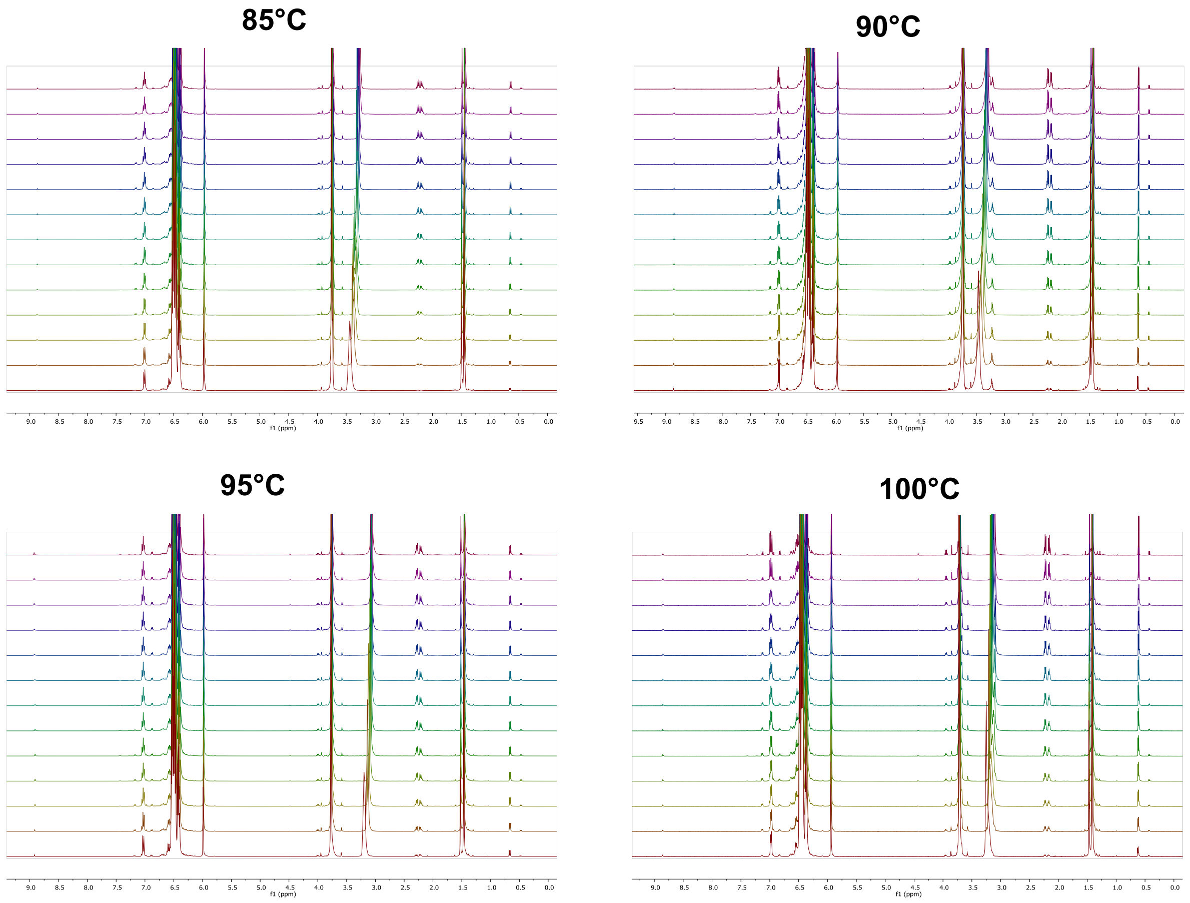

Figure S65 Stacked ^{1}H NMR spectra for kinetic experiments of **[Ru-L^2]PF$_6$** complex, only **B** (δ=0.66 ppm) and **C** (δ=2.28-2.30 ppm) products were observed over 1 hour at varying temperatures

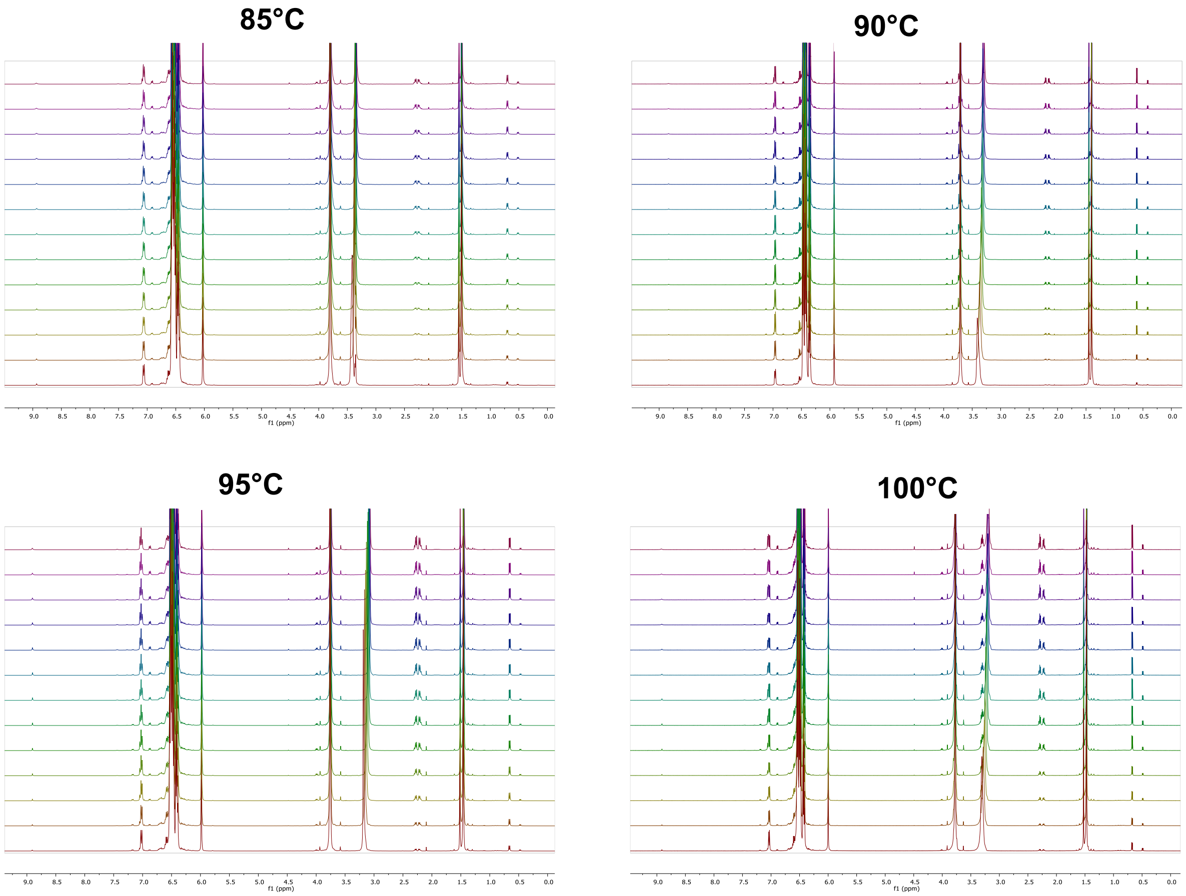

Figure S66 Stacked ^{1}H NMR spectra for kinetic experiments of **[Ir-L^{2}-Ru]PF$_{6}$** complex, only **B** (δ=0.66 ppm) and **C** (δ=2.28-2.30 ppm) products were observed over 1 hour at varying temperatures.

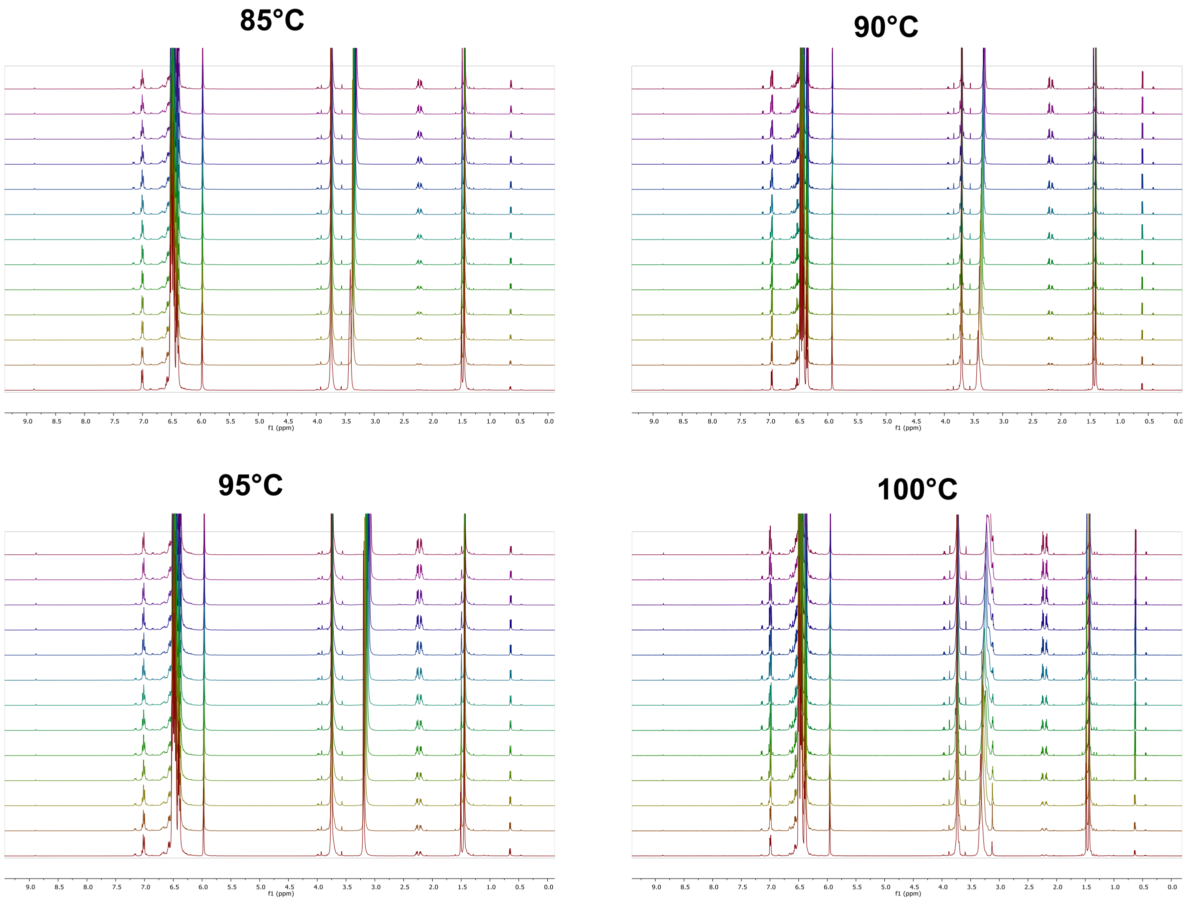

Figure S67 Stacked ^{1}H NMR spectra for kinetic experiments of **[Ru-L^{2}-Ir]PF$_6$** complex, only **B** (δ=0.66 ppm) and **C** (δ=2.28-2.30 ppm) products were observed over 1 hour at varying temperatures.

Analysis of the kinetic results to determine the activation energy Ea

The characteristic signals of products **B** and **C** were observed at a shift of δ = 0.66 ppm (d) and δ = 2.28–2.30 ppm (dd), respectively. These signals are important since they do not overlap with any other signals.

The experiments were conducted for 1 h; however, the reaction rate increased rapidly during the first 15 minutes. Therefore, the initial rate was determined from the data collected within this time interval.

In addition, mesitylene was used as an internal standard, the signals corresponding to the three aromatic protons at δ = 5.99 ppm (s) were used as reference. The yield of product **C** was calculated from the integration ratio between the internal standard and the aliphatic signals of **C** (δ = 2.28-2.30 ppm). Then, the concentration of **C** $[\mathrm{C_C}]$ was obtained:

$$[\mathrm{C_C}] = \frac{[C_A]*\%\ Yield\ \boldsymbol{C}}{100} [=] \frac{\mathrm{mol}}{\mathrm{L}} \qquad \textbf{(Eq. S10)}$$

Where:

$$[\mathrm{C_A}] = \frac{2.885 \times 10^{-4}\ mol}{3.738 \times 10^{-4}\ \mathrm{L}} = 0.7718\ \frac{\mathrm{mol}}{\mathrm{L}}$$

Next, the initial rate v_o was calculated:

$$v_o = \frac{\Delta[\mathrm{C_C}]}{\Delta t} = \frac{mol}{L*s} \qquad \textbf{(Eq. S11)}$$

Which is obtained by linear regression of the time vs [Cc] plot where the slope corresponds to the initial rate at the first 15 minutes of reaction.

Each experiment was performed at least twice, and the reported values represent the average of these measurements.

For example:

The spectrum of the tandem reaction carried out using catalyst **[Ir-L²]PF₆**, at 100 °C, after 15 minutes of reaction is shown in Figure S61. After 15 minutes of reaction, the integral corresponding to **C** was 0.31, corresponding to a 15.5 % yield, therefore, the calculated concentration $[C_C]$ = 0.1196 mol/L, resulting in an initial rate of 1.3 mol/L*s

$$\%\,Yield\ \boldsymbol{C} = \frac{0.31 * 100}{2} = 15.5\,\%$$

$$[C_C] = \frac{0.7718\,\frac{\text{mol}}{\text{L}} * 15.5}{100} = 0.1196\,\frac{\text{mol}}{\text{L}}$$

$$v_o = \frac{0.1196\,\frac{\text{mol}}{\text{L}}}{900\ s} = 1.3x10^{-4}\,\frac{mol}{L * s}$$

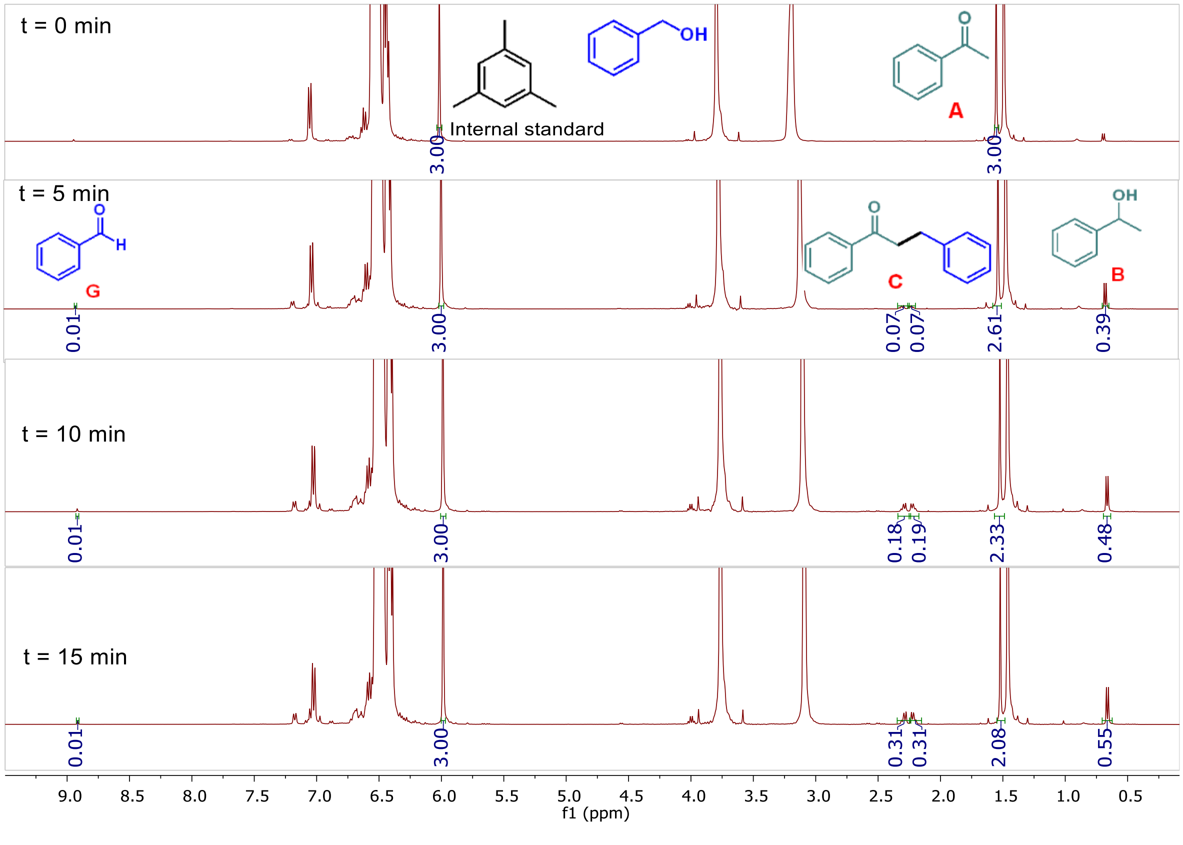

Figure S68. Stacked ^{1}H NMR spectra of the tandem reaction carried out using as catalyst **[Ir-L²]PF₆**, at 100 °C, after 15 minutes of reaction

To calculate the initial rate of [Ir-L^2]PF$_6$

Table S6 Calculation of the initial rate of **[Ir-L^2]PF$_6$** at 85°C

**T = 85°C**			
Time (minutes)	Time (seconds)	% Yield **C**	[Cc] (mol/L)
0	0	0	0.0000
5	300	0.85	0.0066
10	600	1.90	0.0147
15	900	2.98	0.0230

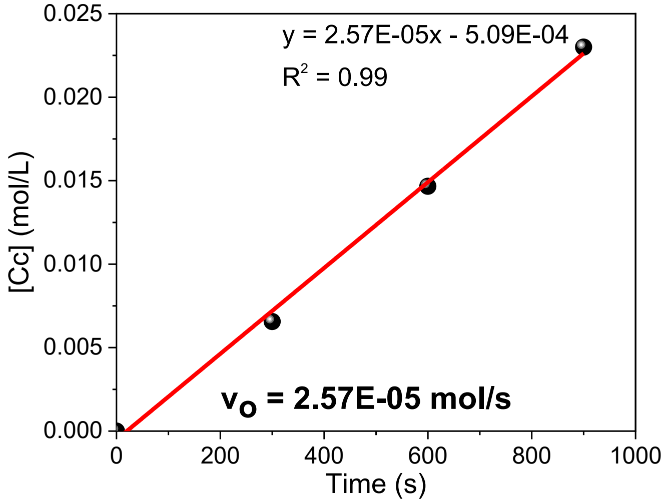

Figure S69 Linear regression for calculating initial rate of **[Ir-L^2]PF$_6$** at 85°C

Table S7 Calculation of the initial rate of **[Ir-L²]PF₆** at 90°C

T = 90°C			
Time (minutes)	Time (seconds)	% Yield **C**	[Cc] (mol/L)
0	0	0	0.0000
5	300	2.35	0.0181
10	600	5.06	0.0390
15	900	10.56	0.0815

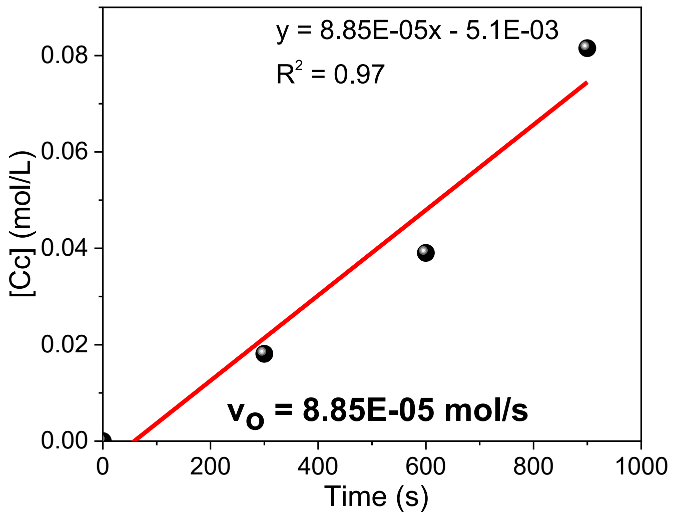

Figure S70 Linear regression for calculating initial rate of **[Ir-L²]PF₆** at 90°C

Table S8 Calculation of the initial rate of **[Ir-L^2]PF$_6$** at 95°C

**T = 95°C**			
Time (minutes)	Time (seconds)	% Yield **C**	[Cc] (mol/L)
0	0	0	0.0000
5	300	3.55	0.0274
10	600	7.89	0.0609
15	900	13.42	0.1036

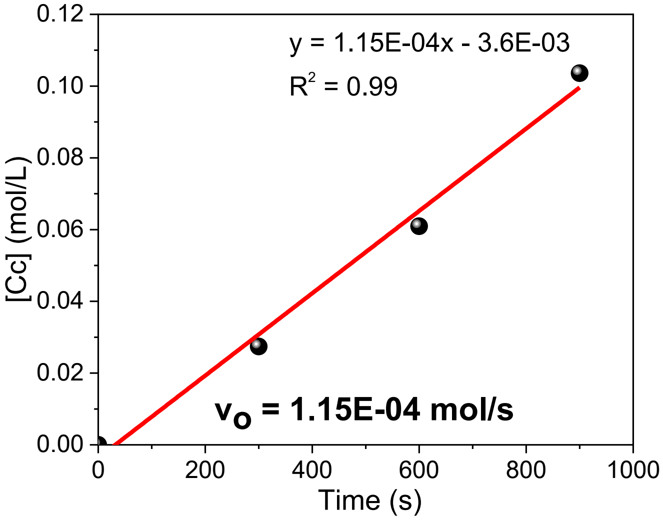

Figure S71. Linear regression for calculating initial rate of **[Ir-L^2]PF$_6$** at 95°C

Table S9 Calculation of the initial rate of **[Ir-L²]PF₆** at 100°C

T = 100°C			
Time (minutes)	Time (seconds)	% Yield **C**	[Cc] (mol/L)
0	0	0	0.0000
5	300	4.84	0.0373
10	600	12.10	0.0934
15	900	18.02	0.1391

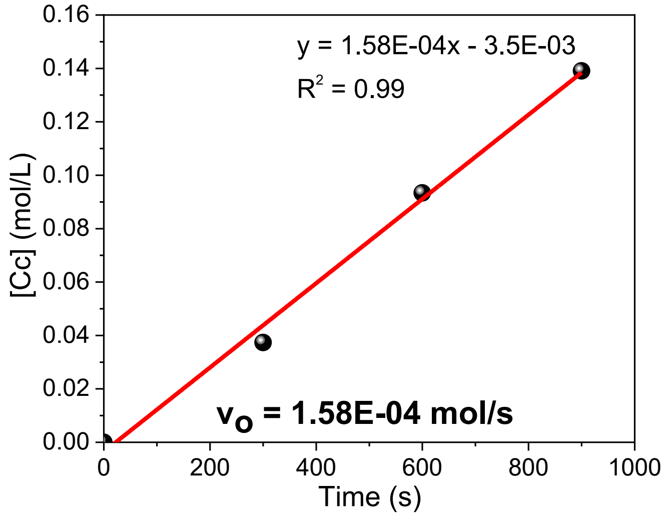

Figure S72 Linear regression for calculating initial rate of **[Ir-L²]PF₆** at 100°C

Once the initial rates at different temperatures (85, 90, 95, and 100 °C) were determined for all catalysts, the data were plotted as ln(vo) vs. 1/T. Linear regression was performed, and the Arrhenius equation was applied in its logarithmic form:

$$\ln v_o = \ln A - \frac{Ea}{RT} \qquad \textbf{(Eq. S12)}$$

Where:

$y = \ln v_o$ $\quad x = \frac{1}{T}$ $\quad m = -\frac{Ea}{R}$ $\quad b = \ln A$

From the slope m, the activation energy Ea was obtained:

$$Ea = -m * R \qquad \textbf{(Eq. S13)}$$

$R = 8.314 \frac{J}{mol\,K}$

Thus, the slope is multiplied by -R and the value of the activation energy was obtained.

Calculation of activation energy

Table S10 Calculation of activation energy of **[Ir-L^2]PF$_6$**

[Ir-L^2]PF$_6$				
Temp (°C)	Temp (K)	v_o (mol/s)	ln v_o	1/T
85	358.14	2.57E-05	-10.57	2.79E-03
90	363.14	8.85E-05	-9.33	2.75E-03
95	368.14	1.15E-04	-9.07	2.72E-03
100	373.14	1.58E-04	-8.75	2.68E-03

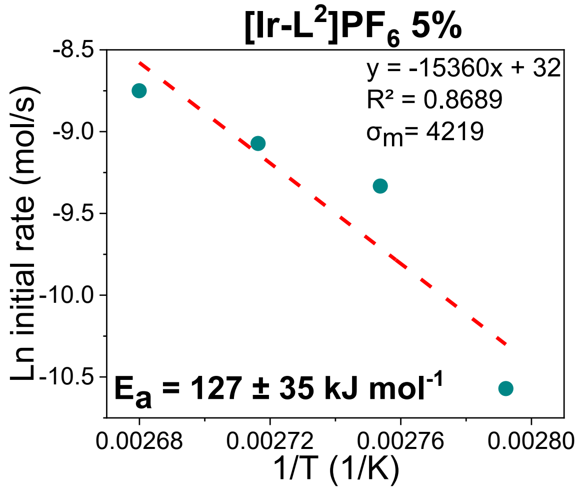

Figure S73. Linear regression for calculating activation energy of **[Ir-L^2]PF$_6$**

Table 11 Calculation of activation energy of **[Ru-L^2]PF$_6$**

**[Ru-L^2]PF$_6$**				
Temp (°C)	Temp (K)	v_o (mol/s)	ln v_o	1/T
85	358.14	8.33E-05	-9.39	2.79E-03
90	363.14	1.73E-04	-8.66	2.75E-03
95	368.14	2.72E-04	-8.21	2.72E-03
100	373.14	3.32E-04	-8.01	2.68E-03

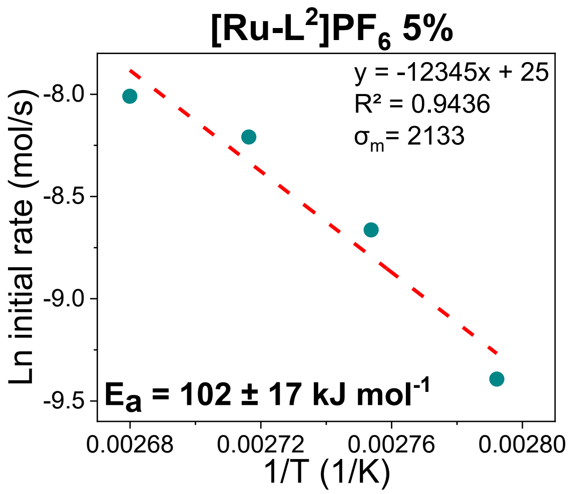

Figure S74 Linear regression for calculating activation energy of **[Ru-L^2]PF$_6$**

Table S12 Calculation of activation energy of **[Ir-L^2-Ru]PF$_6$**

**[Ir-L^2 Ru]PF$_6$**				
Temp (°C)	Temp (K)	v_o (mol/s)	ln v_o	1/T
85	358.14	5.32E-05	-9.84	2.79E-03
90	363.14	6.06E-05	-9.71	2.75E-03
95	368.14	1.06E-04	-9.15	2.72E-03
100	373.14	1.19E-04	-9.03	2.68E-03

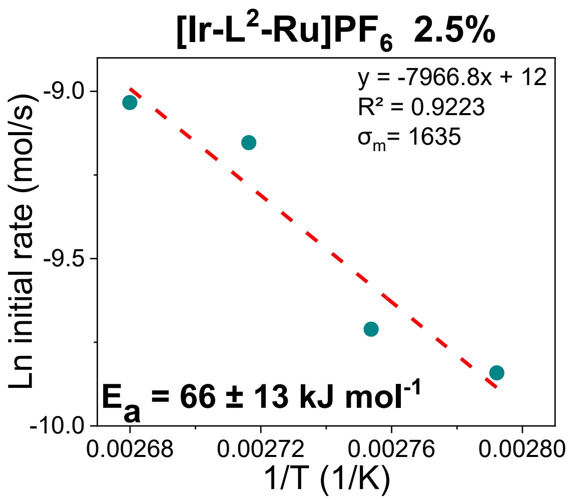

Figure S75 Linear regression for calculating activation energy of **[Ir-L^2-Ru]PF$_6$**

Table S13 Calculation of activation energy of **[Ru-L^2-Ir]PF$_6$**

**[Ru-L^2 Ir]PF$_6$**				
Temp (°C)	Temp (K)	v_o (mol/s)	ln v_o	1/T
85	358.14	8.27E-05	-9.40	2.79E-03
90	363.14	1.25E-04	-8.99	2.75E-03
95	368.14	1.72E-04	-8.67	2.72E-03
100	373.14	2.98E-04	-8.12	2.68E-03

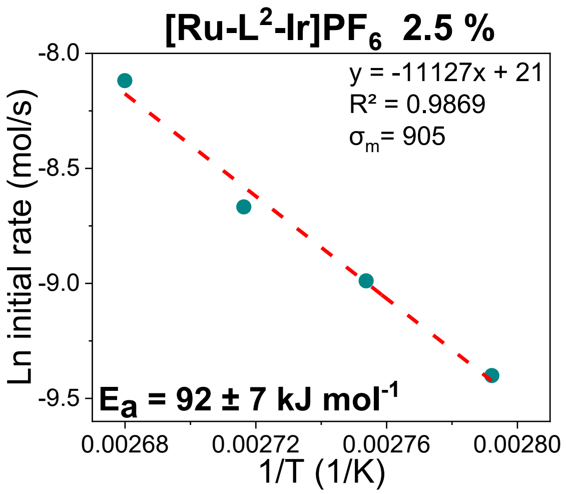

Figure S76 Linear regression for calculating activation energy of **[Ru-L^2-Ir]PF$_6$**

Equations to quantify cooperativity using Ea: [51,52]

$\delta E(kin) = E_a(A) - E_a(AB)$ $\quad$ (if $E_a(A) < E_a(B)$) **(Eq. S14)**

$\delta E(kin) = E_a(B) - E_a(AB)$ $\quad$ (if $E_a(A) > E_a(B)$) **(Eq. S15)**

- If $\delta E(kin) > 0$ ∴ cooperative active
- If $\delta E(kin) = 0$ ∴ $E_a(A) = E_a(B)$
- If $\delta E(kin) < 0$ ∴ not cooperative

Absorption spectroscopy

The absorption spectra of ligands **[HL²]PF₆**, **[HL³]PF₆** and **[HL⁴]PF₆**, mononuclear complexes: **[Ru-L²]PF₆**, **[Ir-L²]PF₆** **[Ir-L³]PF₆** and **[Ir-L⁴]PF₆**, homo- **[Ir-L²-Ir]PF₆** and hetero-dinuclear **[Ir-L²-Ru]PF₆**, **[Ru-L²-Ir]PF₆** complexes were recorded in acetonitrile (Figures 7 and S72 - S75) and the key absorption bands are summarized in Table S13.

The ligands exhibit two intense absorption peaks at 219 to 274 nm (ε = 10589 - 19578 M^{-1} cm^{-1}, Figure S72). The mononuclear complexes exhibit intense absorption peaks at 217 to 279 nm (ε = 14901 – 30772 M^{-1} cm^{-1}, Figure S73) and one additional moderately strong absorption peak at 273 – 350 nm (ε = 1583 – 5943 M^{-1} cm^{-1}). Finally, the dinuclear complexes exhibit the same absorption peaks as the ligands and the mononuclear complexes in addition to one weak absorption peak observed at 435 – 437 nm (ε = 1583 – 5943 M^{-1} cm^{-1}, Figure S74).

Due to the extensive investigation in the literature on related complexes,[53–57] it is possible to assign the intense absorption bands in the UV range (< 300 nm) to ligand-centered electronic $\pi \rightarrow \pi^*$ transitions of the aromatic groups of the ligands. The absorption bands observed for complexes in the visible region in the 340 - 437 nm range are assigned to spin-allowed metal-to-ligand charge-transfer (MLCT) transitions {Ru/Ir (dπ) → (π*) ligand}.

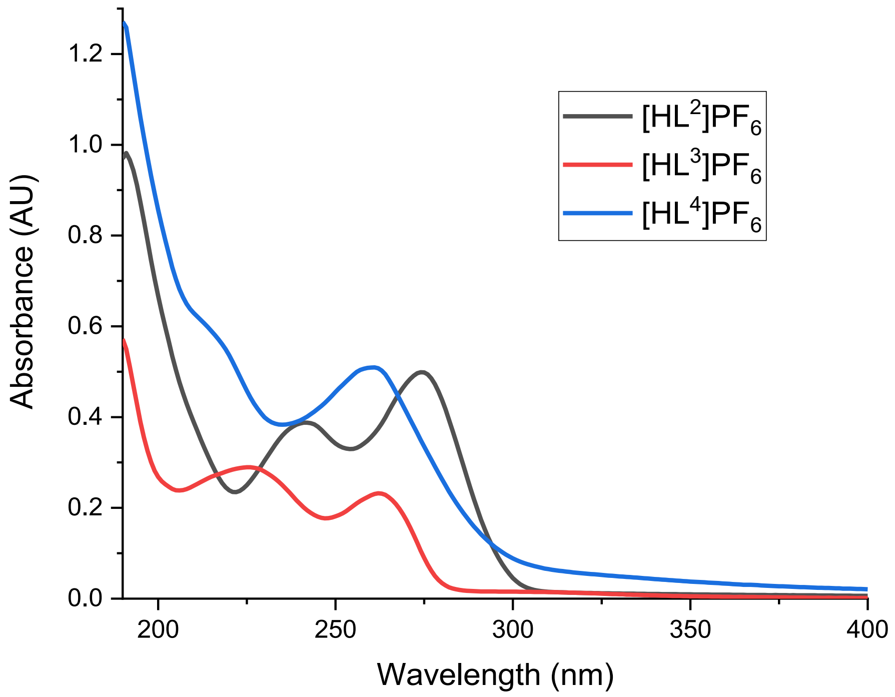

Figure S77 UV–vis spectra of ligands **[HL²]PF₆, [L³]PF₆** and **[L⁴]PF₆** in acetonitrile (3x10^{-3} M)

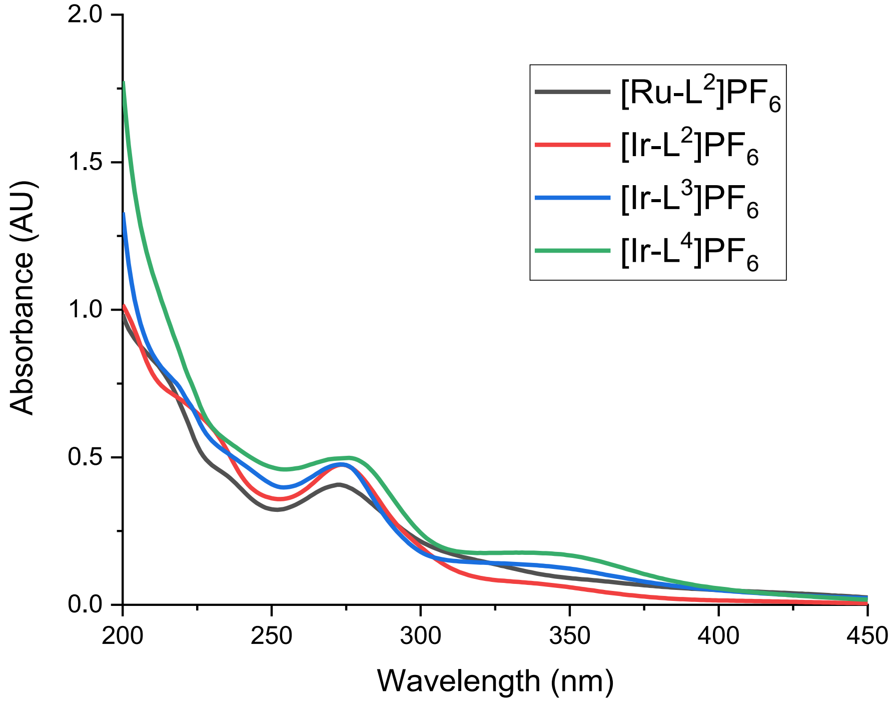

Figure S78 UV–vis spectra of mononuclear complexes **[Ru-L^2]PF$_6$, [Ir-L^2]PF$_6$, [Ir-L^3]PF$_6$** and **[Ir-L^4]** in acetonitrile (3x10^{-3} M)

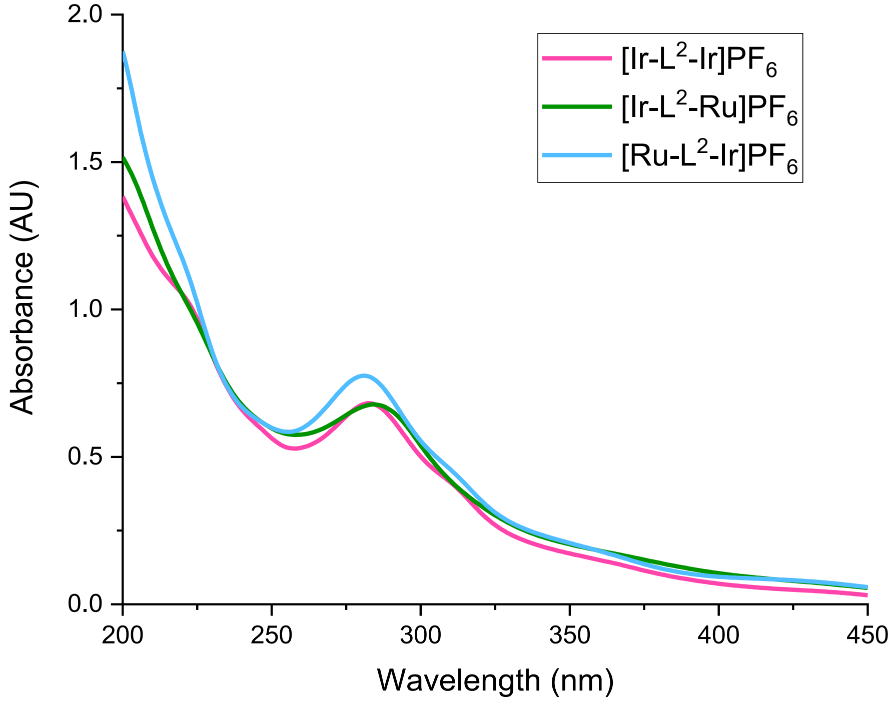

Figure S79 UV–vis spectra of dinuclear complexes **[Ir-L^2-Ir]PF$_6$, [Ir-L^2-Ru]PF$_6$** and **[Ru-L^2-Ir]PF$_6$** in acetonitrile (3x10^{-3} M)

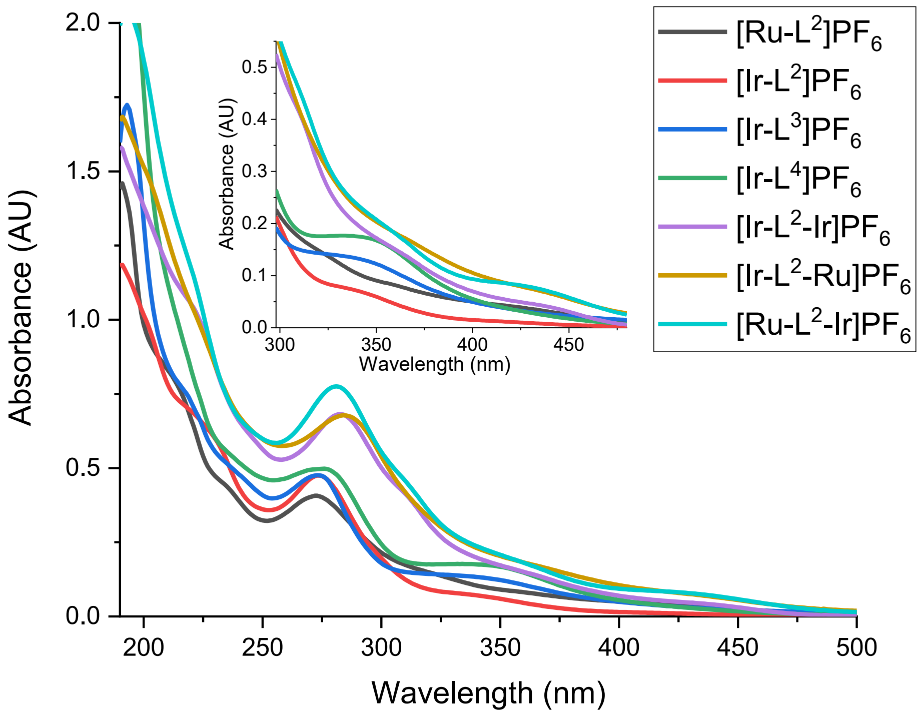

Figure S80 Comparison of UV–vis spectra of mononuclear **[Ru-L[2]]PF6, [Ir-L[2]]PF6, [Ir-L[3]]PF6, [Ir-L[4]]PF6** and dinuclear **[Ir-L[2]-Ir]PF6, [Ir-L[2]-Ru]PF6, [Ru-L[2]-Ir]PF6** complexes in acetonitrile ($3x10^{-3}$ M)

Table S14 UV-vis data for ligands and complexes

Compound	λ, nm (ε, M^{-1} cm^{-1})			
**[L[2]]**	242 (13760)	274 (17590)		
**[L[3]]**	225 (13980)	262 (10590)		
**[L[4]]**	217 (19580)	261 (16710)		
**[Ru-L[2]]**	217 (25950)	235 (15480)	273 (14290)	
**[Ir-L[2]]**	225 (21600)	274 (15800)	340 (2332)	
**[Ir-L[3]]**	219 (24110)	276 (14900)	345 (3884)	
**[Ir-L[4]]**	217 (30770)	279 (16030)	350 (5490)	
**[Ir-L[2]-Ir]**	224 (36320)	283 (24800)	360 (5451)	435 (1583)
**[Ir-L[2]-Ru]**	225 (33570)	287 (23770)	367 (5943)	437 (2447)
**[Ru-L[2]-Ir]**	222 (38330)	284 (25980)	363 (5688)	434 (2425)
CH_3CN solvent, $3x10^{-5}$ M, at 298 K				

Electrochemistry

The cyclic voltammograms of ligand **[HL^2]PF_6**, mononuclear complexes: **[Ru-L^2]PF_6, [Ir-L^2]PF_6 [Ir-L^3]PF_6**, homo- **[Ir-L^2-Ir]PF_6** and hetero-dinuclear **[Ir-L^2-Ru]PF_6**, **[Ru-L^2-Ir]PF_6** complexes were carried out in acetonitrile solution at room temperature, using NBu_4PF_6 (0.1 M) as the supporting electrolyte, with a glassy carbon as working electrode. A selection of the oxidation and reduction peaks potentials vs SCE is given in Table 4 and in Figures S81-S86.

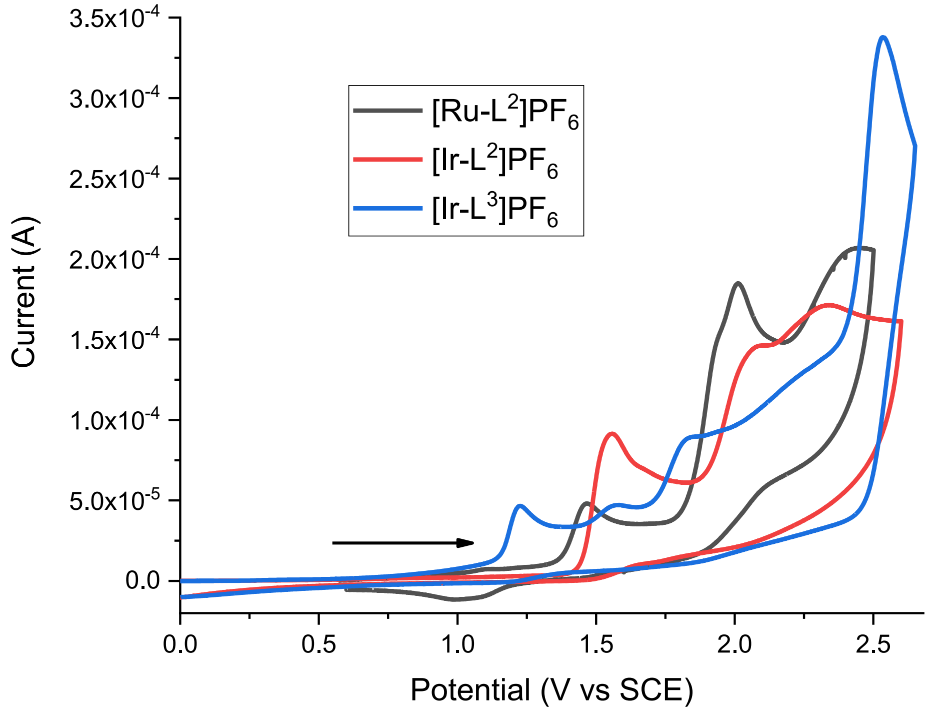

Figure S81 Cyclic voltammograms (oxidation process) of monometallic **[Ru-L^2]PF_6, [Ir-L^2]PF_6, [Ir-L^3]PF_6** complexes, obtained in acetonitrile, at room temperature, using Bu_4NPF_6 (0.1 M) as the supporting electrolyte, with a glassy-carbon electrode, at a scan rate of 100 mV/s.

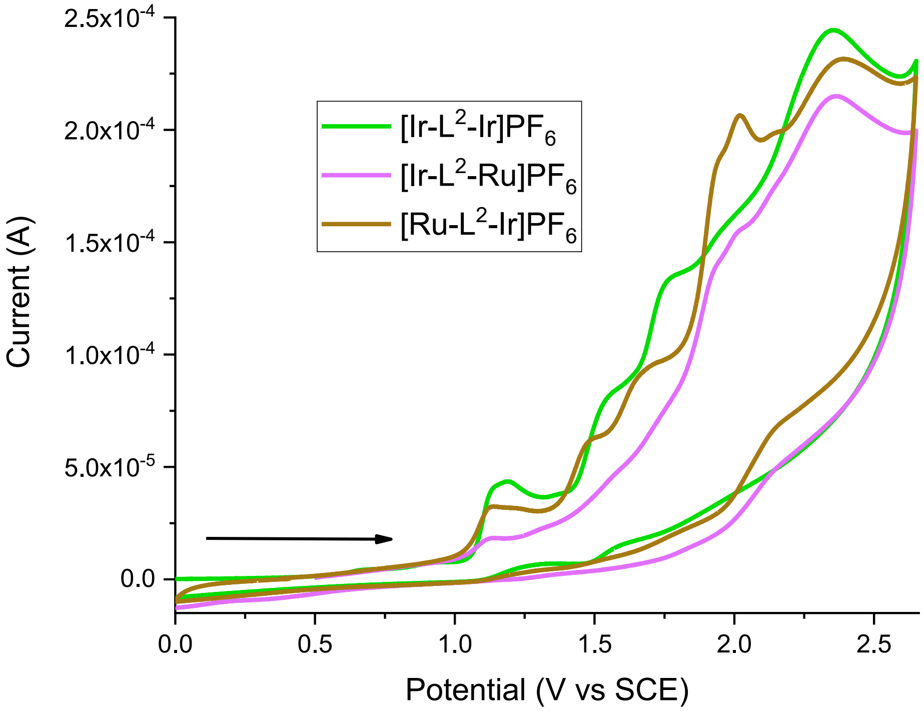

Figure S82 Cyclic voltammograms (oxidation process) of dimetallic **[Ir-L^2-Ir]PF$_6$, [Ir-L^2-Ru]PF$_6$, [Ru-L^2-Ir] PF$_6$** complexes, obtained in acetonitrile, at room temperature, using Bu_4NPF_6 (0.1 M) as the supporting electrolyte, with a glassy-carbon electrode, at a scan rate of 100 mV/s.

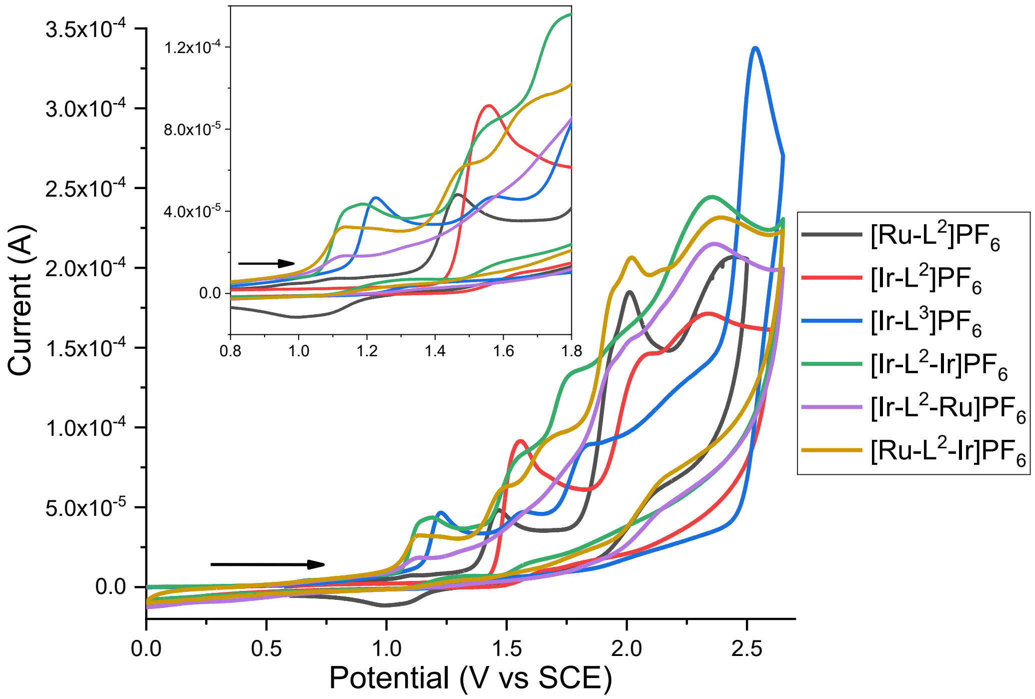

Figure S83 Comparison of cyclic voltammograms (oxidation process) of monometallic **[Ru-L^2]PF$_6$, [Ir-L^2]PF$_6$, [Ir-L^3]PF$_6$,** and bimetallic **[Ir-L^2-Ir]PF$_6$, [Ir-L^2-Ru]PF$_6$, [Ru-L^2-Ir] PF$_6$** complexes, obtained in acetonitrile, at room temperature, using Bu_4NPF_6 (0.1 M) as the supporting electrolyte, with a glassy-carbon electrode, at a scan rate of 100 mV/s.

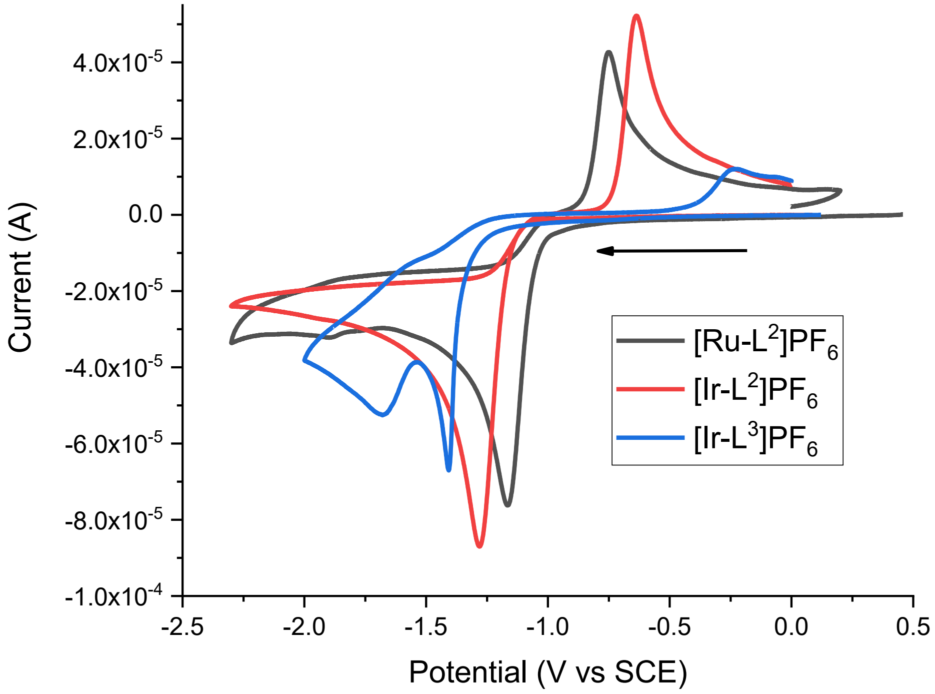

Figure S84 Cyclic voltammograms (reduction process) of monometallic **[Ru-L^2]PF$_6$, [Ir-L^2]PF$_6$, [Ir-L^3]PF$_6$** complexes, obtained in acetonitrile, at room temperature, using Bu_4NPF_6 (0.1 M) as the supporting electrolyte, with a glassy-carbon electrode, at a scan rate of 100 mV/s.

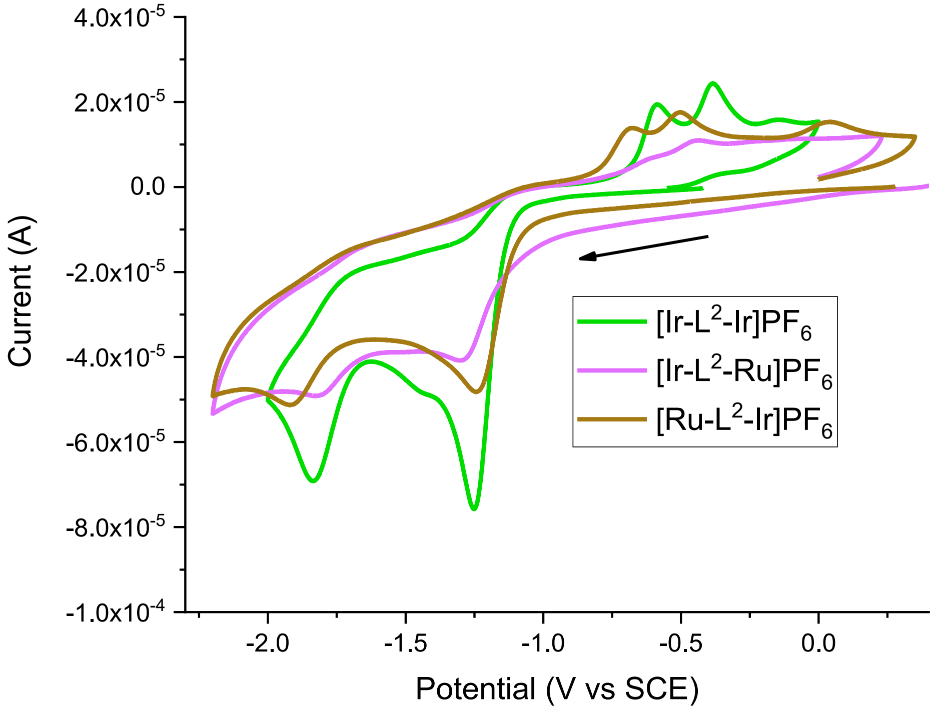

Figure S85 Cyclic voltammograms (reduction process) of bimetallic **[Ir-L^2-Ir]PF$_6$, [Ir-L^2-Ru]PF$_6$, [Ru-L^2-Ir] PF$_6$** complexes, obtained in acetonitrile, at room temperature, using Bu_4NPF_6 (0.1 M) as the supporting electrolyte, with a glassy-carbon electrode, at a scan rate of 100 mV/s.

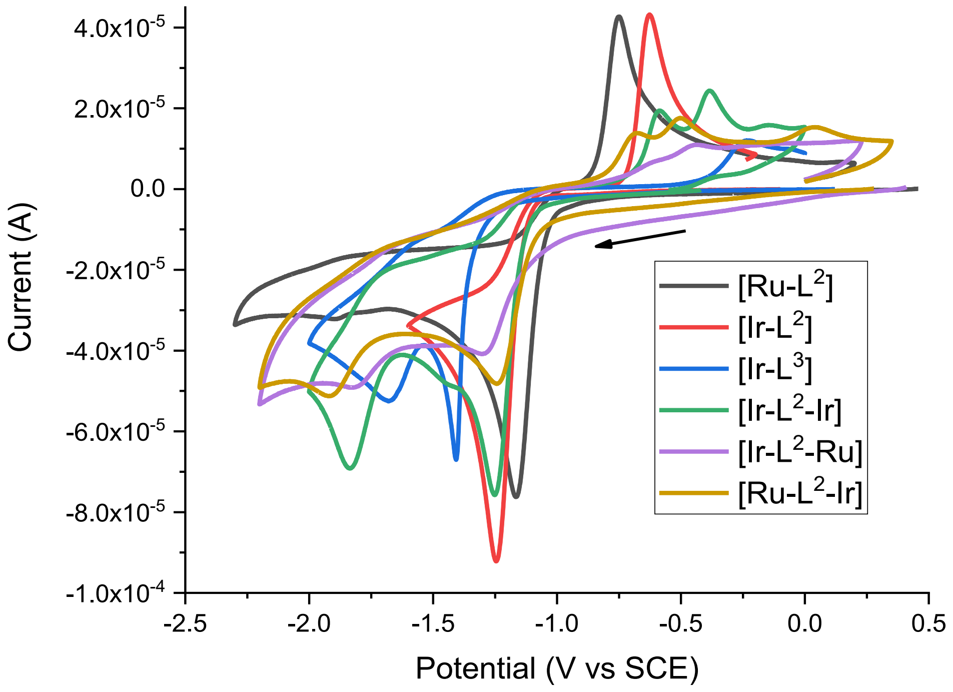

Figure S86 Comparison of cyclic voltammograms (reduction process) of monometallic **[Ru-L²]PF₆, [Ir-L²]PF₆, [Ir-L³]PF₆,** and bimetallic **[Ir-L²-Ir]PF₆, [Ir-L²-Ru]PF₆, [Ru-L²-Ir] PF₆** complexes, obtained in acetonitrile, at room temperature, using Bu_4NPF_6 (0.1 M) as the supporting electrolyte, with a glassy-carbon electrode, at a scan rate of 100 mV/s.

The ligand **[HL²]PF₆** did not show an oxidation process; however, a reduction process was detected, attributed to at least one electrochemical-chemical (EC) mechanism involving initial C2-H cleavage of the imidazolium cation followed by dimerization, consistent with literature reports.[58–61]

The mononuclear complexes **[Ru-L²]PF₆**, **[Ir-L²]PF₆** and **[Ir-L³]PF₆** exhibited a chemically irreversible oxidation process at 1.46, 1.55 and 1.22 V, respectively (Table 4). Since the free ligand does not show oxidation process, these processes must be metal based, corresponding to the $Ru^{2+/3+}$ or $Ir^{3+/4+}$ process, These values are well in agreement with potentials reported in the literature for this kind of metals.[53,54,62,63] For the Ir mononuclear complexes (Figure S87, left), the oxidation potential shifts depending on the coordination site: **[Ir-L²]PF₆** (C2-coordinated) oxidizes at 1.55 V, whereas **[Ir-L³]PF₆** (C4-coordinated) shifts to 1.22 V. All the complexes show further oxidation processes and may correspond to intermediate species or by-products generated during the initial oxidation process (Figures S81-82).

The dinuclear complexes exhibited two main chemically irreversible metal-based oxidation peaks. For **[Ir-L²-Ir]PF₆**, these processes were observed at 1.16 and 1.53 V. By comparison to the mononuclear analogs, the less positive potential (1.16 V) likely corresponds to the Ir center coordinated to the C4 site, while the second potential (1.53 V) corresponds to the C2

site. These results indicate that the C4 site is significantly more electron-donating than the C2 site,[64,65] which effectively lowers the oxidation potential of the metal center.[66,67]

In the case of heterodinuclear **[Ru-L^2-Ir]PF$_6$** two main chemically irreversible oxidation processes were also observed at 1.47 and 1.12 V. Based on the potential values obtained from the mononuclear systems, these can be attributed to the Ru$^{2+/3+}$ and Ir$^{3+/4}$ oxidation process, respectively.

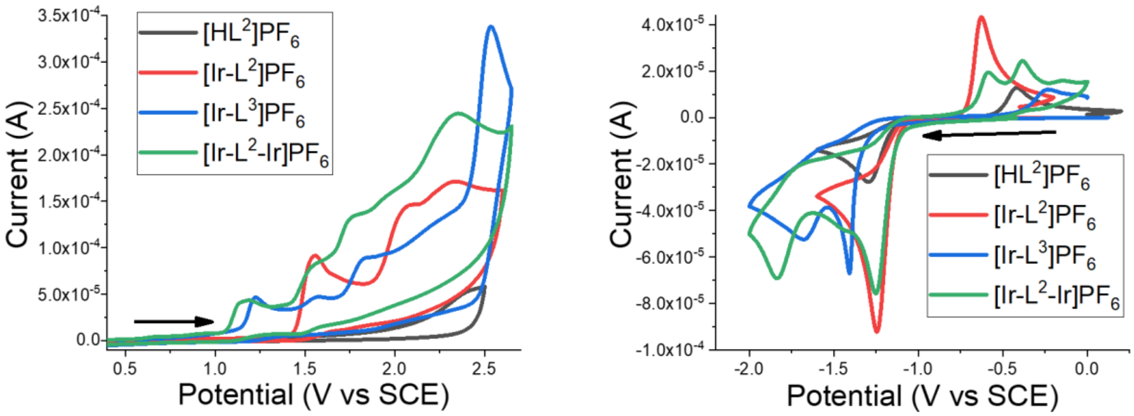

Figure S87. Comparison of cyclic voltammograms for the oxidation (left) and reduction (right) processes for **[HL^2]PF$_6$**, **[Ir-L^2]PF$_6$, [Ir-L^3]PF$_6$,** and **[Ir-L^2-Ir]PF$_6$** compounds, measure in acetonitrile, at room temperature, using Bu_4NPF_6 (0.1 M) as the supporting electrolyte, with a glassy-carbon electrode, at a scan rate of 100 mV/s.

Reliable electrochemical evaluation of electronic coupling in this type of system requires reversible or quasi-reversible behavior; therefore, it is not possible to assess electronic coupling between the metal centers presented in this work, due to the irreversible nature of the oxidation processes in all the complexes.

Regarding the reduction process, complexes. **[Ru-L^2]PF$_6$** and **[Ir-L^2]PF$_6$** exhibit a chemically irreversible reduction peak at -1.17 and -1.24 V, respectively, which could be ligand based. In contrast **[Ir-L^3]PF$_6$** shows two chemically irreversible reduction peaks at -1.41 and -1.67 V. Similarly, the dinuclear complexes **[Ir-L^2-Ir]PF$_6$**, **[Ir-L^2-Ru]PF$_6$** and **[Ru-L^2-Ir]PF$_6$** each display two chemically irreversible reduction processes; the first occurs between -1.23 to -1.29 and the second between -1.67 to -1.90 V. The second process, observed at more negative potentials, is likely associated with the reduction of a different ligand site or a chemical process following the initial electron transfer.

It should be emphasized that the irreversibility of these reduction processes precludes a straightforward comparison of peak currents. The observed variations are most likely attributable to diffusional and kinetic effects rather than differences in the number of electrons transferred.

A comparison of the CVs in anodic direction for **[HL^2]PF$_6$**, **[Ir-L^2]PF$_6$**, **[Ir-L^3]PF$_6$** and **[Ir-L^2-Ir]PF$_6$** (Figure 87, right) reveals differences in peak current intensities between the free ligand and the mono and dinuclear complexes. These variations can be ascribed to halide dissociation and ligand reduction, which can lead to an increase of electron transfer during the electrochemical process.

References

(1) Zanardi, A.; Corberán, R.; Mata, J. A.; Peris, E. Homo- And Heterodinuclear Complexes with Triazolyl-Diylidene. An Easy Approach to Tandem Catalysts. *Organometallics* **2008**, *27*, 3570–3576. DOI: 10.1021/om800411v.

(2) Zanardi, A.; Mata, J. A.; Peris, E. Well-Defined Ir/Pd Complexes with a Triazolyl-Diylidene Bridge as Catalysts for Multiple Tandem Reactions. *J. Am. Chem. Soc.* **2009**, *131* (40), 14531–14537. DOI: 10.1021/ja906028g.

(3) Sabater, S.; Mata, J. A.; Peris, E. Dual Catalysis with an Ir III-Au I Heterodimetallic Complex: Reduction of Nitroarenes by Transfer Hydrogenation Using Primary Alcohols. *Chem. Eur. J.* **2012**, *18*, 6380–6385. DOI: 10.1002/chem.201103657.

(4) Sabater, S.; Mata, J. A.; Peris, E. Hydrodefluorination of Carbon-Fluorine Bonds by the Synergistic Action of a Ruthenium-Palladium Catalyst. *Nat. Commun.* **2013**, *4*, 2553. DOI: 10.1038/ncomms3553.

(5) Grineva, A. A.; Filippov, O. A.; Canac, Y.; Sortais, J. B.; Nefedov, S. E.; Lugan, N.; César, V.; Valyaev, D. A. Experimental and Theoretical Insights into the Electronic Properties of Anionic N-Heterocyclic Dicarbenes through the Rational Synthesis of Their Transition Metal Complexes. *Inorg. Chem.* **2021**, *60*, 4015–4025. DOI: 10.1021/acs.inorgchem.1c00073.

(6) Bitzer, M. J.; Pöthig, A.; Jandl, C.; Kühn, F. E.; Baratta, W. Ru-Ag and Ru-Au Dicarbene Complexes from an Abnormal Carbene Ruthenium System. *Dalton Trans.* **2015**, *44*, 11686–11689. DOI: 10.1039/c5dt01914a.

(7) Rottschäfer, D.; Ebeler, F.; Strothmann, T.; Neumann, B.; Stammler, H. G.; Mix, A.; Ghadwal, R. S. The Viability of C5-Protonated- and C4,C5-Ditopic Carbanionic Abnormal NHCs: A New Dimension in NHC Chemistry. *Chem. Eur. J.* **2018**, *24*, 3716–3720. DOI: 10.1002/chem.201800260.

(8) Ellul, C. E.; Mahon, M. F.; Saker, O.; Whittlesey, M. K. Abnormally Bound N-Heterocyclic Carbene Complexes of Ruthenium: C-H Activation of Both C4 and C5 Positions in the Same Ligand. *Angew. Chemie - Int. Ed.* **2007**, *46*, 6343–6345. DOI: 10.1002/anie.200701930.

(9) Hu, Z.; Ma, X.; Wang, J.; Wang, H.; Han, X.; Shi, M.; Zhang, J. Six-Membered Janus-Type Ditopic N-Heterocyclic Carbene Coinage Metal Complexes. *Organometallics* **2019**, *38* (9), 2132–2137. DOI: 10.1021/acs.organomet.9b00124.

(10) Leung, J. N.; Huynh, H. V. Design of a Mesoionic Janus-Type Dicarbene. *J. Am. Chem. Soc.* **2024**, *146* (6), 3622–3626. DOI: 10.1021/jacs.3c13284.

(11) Leung, J. N.; Huynh, H. V. Mesoionic Janus-Type Dicarbene: Complexes, Adducts, and Catalytic Studies. *Chem. Eur. J.* **2024**, *30*, e202402127. DOI: 10.1002/chem.202402127.

(12) Leung, J. N.; Huynh, H. V. Dinuclear Gold(I) and Gold(III) Complexes of Janus Di-N-Heterocyclic Carbenes. *Inorg. Chem.* **2025**, *64*, 15206−15216. DOI: 10.1021/acs.inorgchem.5c02302.

(13) Boydston, A. J.; Williams, K. A.; Bielawski, C. W. A Modular Approach to Main-Chain Organometallic Polymers. *J. Am. Chem. Soc.* **2005**, *127*, 12496–12497. DOI: 10.1021/ja054029k.

(14) Tennyson, A. G.; Rosen, E. L.; Collins, M. S.; Lynch, V. M.; Bielawski, C. W. Bimetallic N-Heterocyclic Carbene-Lridium Complexes: Investigating Metal-Metal and Metal-Ligand Communication via Electrochemistry and Phosphorescence Spectroscopy. *Inorg. Chem.* **2009**, *48* (14), 6924–6933. DOI: 10.1021/ic900391q.

(15) Poyatos, M.; Peris, E. Insights into the Past and Future of Janus-Di-N-Heterocyclic Carbenes. *Dalton Trans.* **2021**, *50*, 1772. DOI: 10.1039/D1DT02035H.

(16) Cheong, Y. J.; Sung, K.; Kim, J. A.; Kim, Y. K.; Jang, H. Y. Highly Efficient Iridium-Catalyzed Production of Hydrogen and Lactate from Glycerol: Rapid Hydrogen Evolution by Bimetallic Iridium Catalysts. *Eur. J. Inorg. Chem.* **2020**, 4064–4068. DOI: 10.1002/ejic.202000670.

(17) Marcheggiani, E.; Tubaro, C.; Biffis, A.; Graiff, C.; Baron, M. Hydroalkoxylation of Terminal and Internal Alkynes Catalyzed by Dinuclear Gold(I) Complexes with Bridging Di(N-Heterocyclic Carbene) Ligands. *Catalysts* **2020**, *10* (1), 1. DOI: 10.3390/catal10010001.

(18) Zhou, Y.; Xi, Z.; Chen, W.; Wang, D. Dinickel(II) Complexes of Bis(N-Heterocyclic Carbene) Ligands Containing [Ni2(μ-OH)] Cores as Highly Efficient Catalysts for the Coupling of Aryl Chlorides. *Organometallics* **2008**, *27* (22), 5911–5920. DOI: 10.1021/om800711g.

(19) Majumder, A.; Naskar, R.; Roy, P.; Maity, R. Homo- and Heterobimetallic Complexes Bearing NHC Ligands: Applications in α-Arylation of Amide, Suzuki–Miyaura Coupling Reactions, and Tandem Catalysis. *Eur. J. Inorg. Chem.* **2019**, No. 13, 1810–1815. DOI: 10.1002/ejic.201801570.

(20) Nishad, R. C.; Kumar, S.; Rit, A. Hetero- and Homobimetallic Complexes Bridged by a Bis(NHC) Ligand: Synthesis via Selective Sequential Metalation and Catalytic Applications in Tandem Organic Transformations. *Organometallics* **2021**, *40* (7), 915–926. DOI: 10.1021/acs.organomet.1c00019.

(21) Böhmer, M.; Kampert, F.; Tan, T. T. Y.; Guisado-Barrios, G.; Peris, E.; Hahn, F. E. IrIII/AuI and RhIII/AuI Heterobimetallic Complexes as Catalysts for the Coupling of Nitrobenzene and Benzylic Alcohol. *Organometallics* **2018**, *37* (21), 4092–4099. DOI: 10.1021/acs.organomet.8b00642.

(22) Zanardi, A.; Mata, J. A.; Peris, E. An Ir-Pt Catalyst for the Multistep Preparation of Functionalized Indoles from the Reaction of Amino Alcohols and Alkynyl Alcohols. *Chem. Eur. J.* **2010**, *16*, 13109–13115. DOI: 10.1002/chem.201001180.

(23) Majumder, A.; Nath Saha, T.; Majumder, N.; Naskar, R.; Pal, K.; Maity, R. Heterobimetallic Carbene Complexes Bearing Cyclometalated IrIII/RhIII and Mixed NHC∧ Py/PPh3 Coordinated PdII Centers: Structures and Tandem Catalysis. *Eur. J. Inorg. Chem.* **2021**, *2021* (12), 1104–1110. DOI: 10.1002/ejic.202001080.

(24) Pedersen, A. K.; Fitzgerald, G. A. Preparation and Analysis of Deuterium-labeled Aspirin: Application to Pharmacokinetic Studies. *J. Pharm. Sci.* **1985**, *74* (2), 188–192. DOI: 10.1002/jps.2600740217.

(25) Smith, J. G.; Dibble, P. W.; Sandborn, R. E. The Preparation and Reactions of Naphtho[1,2-c]Furan and Naphtho[2,3-c]Furan. *J. Org. Chem.* **1986**, No. 51, 3762–3768.

(26) Meddour, A.; Courtieu, J. Achiral Deuterated Derivatizing Agent for Enantiomeric Analysis of Carboxylic Acids by NMR in a Chiral Liquid Crystalline Solvent. *Tetrahedron Asymmetry* **2000**, *11* (17), 3635–3644. DOI: 10.1016/S0957-4166(00)00321-9.

(27) Cho, B. T.; Kang, S. K.; Kim, M. S.; Ryu, S. R.; An, D. K. Solvent-Free Reduction of Aldehydes and Ketones Using Solid Acid-Activated Sodium Borohydride. *Tetrahedron* **2006**, *62* (34), 8164–8168. DOI: 10.1016/j.tet.2006.05.083.

(28) Alonso, F.; Riente, P.; Yus, M. Alcohols for the α-Alkylation of Methyl Ketones and Indirect Aza-Wittig Reaction Promoted by Nickel Nanoparticles. *Eur. J. Org. Chem.* **2008**, No. 29, 4908–4914. DOI: 10.1002/ejoc.200800729.

(29) Liang, Y. F.; Zhou, X. F.; Tang, S. Y.; Huang, Y. B.; Feng, Y. S.; Xu, H. J. Lithium Tert-Butoxide Mediated α-Alkylation of Ketones with Primary Alcohols under Transition-Metal-Free Conditions. *RSC Adv.* **2013**, *3* (21), 7739–7742. DOI: 10.1039/c3ra23221b.

(30) Kobayashi, Y.; Hayashi, N.; Tan, C.-H.; Kishi, Y. Toward the Creation of NMR Database in Chiral Solvents for Assignments of Relative and Absolute Stereochemistry: Proof of Concept. *Org. Lett.* **2001**, *2* (14), 2245–2248. DOI: 10.1021/ol010108z.

(31) Gärtner, D.; Stein, A. L.; Grupe, S.; Arp, J.; Von Wangelin, A. J. Iron-Catalyzed Cross-Coupling of Alkenyl Acetates. *Angew. Chemie - Int. Ed.* **2015**, *54* (36), 10545–10549. DOI: 10.1002/anie.201504524.

(32) George, S. R. D.; Frith, T. D. H.; Thomas, D. S.; Harper, J. B. Putting Corannulene in Its Place. Reactivity Studies Comparing Corannulene with Other Aromatic Hydrocarbons. *Org. Biomol. Chem.* **2015**, *13* (34), 9035–9041. DOI: 10.1039/c5ob01215e.

(33) Yang, D.; Sun, P.; Wei, W.; Liu, F.; Zhang, H.; Wang, H. Copper-Catalyzed Regioselective Cleavage of C−X and C−H Bonds: A Strategy for Sulfur Dioxide Fixation. *Chem. Eur. J.* **2018**, *24* (17), 4423–4427. DOI: 10.1002/chem.201705866.

(34) Kratzert, D.; Holstein, J. J.; Krossing, I. DSR: Enhanced Modelling and Refinement of Disordered Structures with SHELXL. *J. Appl. Crystallogr.* **2015**, *48*, 933–938. DOI: 10.1107/S1600576715005580.

(35) Kratzert, D.; Krossing, I. Recent Improvements in DSR. *J. Appl. Crystallogr.* **2018**, *51* (Petr 2014), 928–934. DOI: 10.1107/S1600576718004508.

(36) Spek, A. L. Single-Crystal Structure Validation with the Program PLATON. *J. Appl. Crystallogr.* **2003**, *36*, 7–13. DOI: 10.1107/S0021889802022112.

(37) Wang, D.; McBurney, R. T.; Pernik, I.; Messerle, B. A. Controlling the Selectivity and Efficiency of the Hydrogen Borrowing Reaction by Switching between Rhodium and Iridium Catalysts. *Dalton Trans.* **2019**, *48*, 13989–13999. DOI: 10.1039/c9dt02819f.

(38) Liu, J.; Li, W.; Li, Y.; Liu, Y.; Ke, Z. Selective C-Alkylation Between Alcohols Catalyzed by N-Heterocyclic Carbene Molybdenum. *Chem. - An Asian J.* **2021**, *16* (20), 3124–3128. DOI: 10.1002/asia.202100959.

(39) Kaur, M.; U Din Reshi, N.; Patra, K.; Bhattacherya, A.; Kunnikuruvan, S.; Bera, J. K. A Proton-Responsive Pyridyl(Benzamide)-Functionalized NHC Ligand on Ir Complex

for Alkylation of Ketones and Secondary Alcohols. *Chem. Eur. J.* **2021**, *27*, 10737–10748. DOI: 10.1002/chem.202101360.

(40) Martínez, R.; Ramón, D. J.; Yus, M. Easy α-Alkylation of Ketones with Alcohols through a Hydrogen Autotransfer Process Catalyzed by RuCl2(DMSO)4. *Tetrahedron* **2006**, *62* (38), 8988–9001. DOI: 10.1016/j.tet.2006.07.013.

(41) Cho, C. S.; Kim, B. T.; Kim, T. J.; Shim, S. C. An Unusual Type of Ruthenium-Catalyzed Transfer Hydrogenation of Ketones with Alcohols Accompanied by C-C Coupling. *J. Org. Chem.* **2001**, *66* (26), 9020–9022. DOI: 10.1021/jo0108459.

(42) Yang, J.; Liu, X.; Meng, D. L.; Chen, H. Y.; Zong, Z. H.; Feng, T. T.; Sun, K. Efficient Iron-Catalyzed Direct β-Alkylation of Secondary Alcohols with Primary Alcohols. *Adv. Synth. Catal.* **2012**, *354* (2–3), 328–334. DOI: 10.1002/adsc.201000907.

(43) Satyanarayana, P.; Reddy, G. M.; Maheswaran, H.; Kantam, M. L. Tris(Acetylacetonato)Rhodium(III)-Catalyzed α-Alkylation of Ketones, β-Alkylation of Secondary Alcohols and Alkylation of Amines with Primary Alcohols. *Adv. Synth. Catal.* **2013**, *355* (9), 1859–1867. DOI: 10.1002/adsc.201300061.

(44) Xu, Q.; Chen, J.; Tian, H.; Yuan, X.; Li, S.; Zhou, C.; Liu, J. Catalyst-Free Dehydrative α-Alkylation of Ketones with Alcohols: Green and Selective Autocatalyzed Synthesis of Alcohols and Ketones. *Angew. Chemie - Int. Ed.* **2014**, *53*, 225–229. DOI: 10.1002/anie.201308642.

(45) Francos, J.; Menéndez-Rodríguez, L.; Tomás-Mendivil, E.; Crochet, P.; Cadierno, V. Synthesis and Catalytic Applications of Ruthenium(II)-Phosphino-Oxime Complexes. *RSC Adv.* **2016**, *6* (45), 39044–39052. DOI: 10.1039/c6ra07015a.

(46) Das, J.; Vellakkaran, M.; Banerjee, D. Nickel-Catalyzed Alkylation of Ketone Enolates: Synthesis of Monoselective Linear Ketones. *J. Org. Chem.* **2019**, *84* (2), 769–779. DOI: 10.1021/acs.joc.8b02609.

(47) Dehury, N.; Mishra, S. R.; Laha, P.; Patra, S. Tandem α/β-Alkylation and Transfer Hydrogenation by Heterodimetallic Ruthenium-Iridium Complex. *Inorganica Chim. Acta* **2020**, *511*, 119796. DOI: 10.1016/j.ica.2020.119796.

(48) Genç, S.; Arslan, B.; Gülcemal, D.; Gülcemal, S.; Günnaz, S. Nickel-Catalyzed Alkylation of Ketones and Nitriles with Primary Alcohols. *Org. Biomol. Chem.* **2022**, *20* (48), 9753–9762. DOI: 10.1039/d2ob01787c.

(49) Jones, N. D.; James, B. R. Homo- and Heterobimetallic Precursor Catalysts for the Heck Reaction, and a Proposal for a General Catalytic Cooperativity Index. *Adv. Synth. Catal.* **2002**, *344*, 1126–1134. DOI: 10.1002/1615-4169(200212)344:10<1126::AID-ADSC1126>3.0.CO;2-5.

(50) Wong, C. M.; McBurney, R. T.; Binding, S. C.; Peterson, M. B.; Gonçales, V. R.; Gooding, J. J.; Messerle, B. A. Iridium(III) Homo- and Heterogeneous Catalysed Hydrogen Borrowing C-N Bond Formation. *Green Chem.* **2017**, *19*, 3142–3151. DOI: 10.1039/c7gc01007a.

(51) Becker, S. Understanding Cooperativity in Homo- and Heterometallic Complexes: From Basic Concepts to Design. *ChemPlusChem* **2024**, *89*, e202300619. DOI: 10.1002/cplu.202300619.

(52) Tebben, L.; Mück-Lichtenfeld, C.; Fernández, G.; Grimme, S.; Studer, A. From

Additivity to Cooperativity in Chemistry: Can Cooperativity Be Measured? *Chem. Eur. J.* **2017**, *23*, 5864–5873. DOI: 10.1002/chem.201604651.

(53) Leigh, V.; Ghattas, W.; Lalrempuia, R.; Müller-Bunz, H.; Pryce, M. T.; Albrecht, M. Synthesis, Photo-, and Electrochemistry of Ruthenium Bis(Bipyridine) Complexes Comprising a N-Heterocyclic Carbene Ligand. *Inorg. Chem.* **2013**, *52*, 5395–5402. DOI: 10.1021/ic400347r.

(54) Barbante, G. J.; Doeven, E. H.; Francis, P. S.; Stringer, B. D.; Hogan, C. F.; Kheradmand, P. R.; Wilson, D. J. D.; Barnard, P. J. Iridium(III) N-Heterocyclic Carbene Complexes: An Experimental and Theoretical Study of Structural, Spectroscopic, Electrochemical and Electrogenerated Chemiluminescence Properties. *Dalton Trans.* **2015**, *44*, 8564–8576. DOI: 10.1039/c4dt03378g.

(55) Siek, S.; Burks, D. B.; Gerlach, D. L.; Liang, G.; Tesh, J. M.; Thompson, C. R.; Qu, F.; Shankwitz, J. E.; Vasquez, R. M.; Chambers, N.; Szulczewski, G. J.; Grotjahn, D. B.; Webster, C. E.; Papish, E. T. Iridium and Ruthenium Complexes of N-Heterocyclic Carbene- and Pyridinol-Derived Chelates as Catalysts for Aqueous Carbon Dioxide Hydrogenation and Formic Acid Dehydrogenation: The Role of the Alkali Metal. *Organometallics* **2017**, *36*, 1091–1106. DOI: 10.1021/acs.organomet.6b00806.

(56) Sawkmie, M.; Bhattacharyya, M.; Banothu, V.; Kaminsky, W.; Gannon, P. M.; Majaw, S.; Kollipara, M. R. Ruthenium, Rhodium, and Iridium Complexes Featuring Fluorenyl Benzohydrazone Derivatives: Synthesis and Preliminary Investigation of Their Anticancer and Antibacterial Activity. *J. Mol. Struct.* **2023**, *1291*, 135994. DOI: 10.1016/j.molstruc.2023.135994.

(57) Zahirović, A.; Fetahović, S.; Feizi-Dehnayebi, M.; Višnjevac, A.; Bešta-Gajević, R.; Kozarić, A.; Martić, L.; Topčagić, A.; Roca, S. Dual Antimicrobial-Anticancer Potential, Hydrolysis, and DNA/BSA Binding Affinity of a Novel Water-Soluble Ruthenium-Arene Ethylenediamine Schiff Base (RAES) Organometallic. *Spectrochim. Acta - Part A Mol. Biomol. Spectrosc.* **2024**, *318*, 124528. DOI: 10.1016/j.saa.2024.124528.

(58) Ogawa, K. A.; Boydston, A. J. Electrochemical Characterization of Azolium Salts. *Chem. Lett.* **2014**, *43*, 907–909. DOI: 10.1246/cl.140162.

(59) Feroci, M.; Chiarotto, I.; D'Anna, F.; Forte, G.; Noto, R.; Inesi, A. Stability and Organocatalytic Efficiency of N-Heterocyclic Carbenes Electrogenerated in Organic Solvents from Imidazolium Ionic Liquids. *Electrochim. Acta* **2015**, *153*, 122–129. DOI: 10.1016/j.electacta.2014.11.135.

(60) Feroci, M.; Chiarotto, I.; D'Anna, F.; Gala, F.; Noto, R.; Ornano, L.; Zollo, G.; Inesi, A. N-Heterocyclic Carbenes and Parent Cations: Acidity, Nucleophilicity, Stability, and Hydrogen Bonding—Electrochemical Study and Ab Initio Calculations. *ChemElectroChem* **2016**, *3*, 1133–1141. DOI: 10.1002/celc.201600187.

(61) Schotten, C.; Bourne, R. A.; Kapur, N.; Nguyen, B. N.; Willans, C. E. Electrochemical Generation of N-Heterocyclic Carbenes for Use in Synthesis and Catalysis. *Adv. Synth. Catal.* **2021**, *363*, 3189–3200. DOI: 10.1002/adsc.202100264.

(62) Cavazzini, M.; Quici, S.; Scalera, C.; Puntoriero, F.; La Ganga, G.; Campagna, S. Synthesis, Characterization, Absorption Spectra, and Luminescence Properties of Multinuclear Species Made of Ru(II) and Ir(III) Chromophores. *Inorg. Chem.* **2009**, *48*, 8578–8592. DOI: 10.1021/ic9006108.

(63) Tripathy, S. K.; De, U.; Dehury, N.; Pal, S.; Kim, H. S.; Patra, S. Dinuclear [{(p-Cym)RuCl}2(μ-Phpy)](PF6)2 and Heterodinuclear [(Ppy)2Ir(μ-Phpy)Ru(p-Cym)Cl](PF6)2 Complexes: Synthesis, Structure and Anticancer Activity. *Dalton Trans.* **2014**, *43*, 14546–14549. DOI: 10.1039/c4dt01033g.

(64) Chianese, A. R.; Kovacevic, A.; Zeglis, B. M.; Faller, J. W.; Crabtree, R. H. Abnormal C5-Bound N-Heterocyclic Carbenes: Extremely Strong Electron Donor Ligands and Their Iridium(I) and Iridium(III) Complexes. *Organometallics* **2004**, *23*, 2461–2468. DOI: 10.1021/om049903h.

(65) Crabtree, R. H. Abnormal, Mesoionic and Remote N-Heterocyclic Carbene Complexes. *Coord. Chem. Rev.* **2013**, *257*, 755–766. DOI: 10.1016/j.ccr.2012.09.006.

(66) Schuster, O.; Mercsa, L.; Albrecht, M. The Potential of N-Heterocyclic Carbene Complexes as Components for Electronically Active Materials. *Chimia* **2010**, *64*, 184–187. DOI: 10.2533/chimia.2010.184.

(67) van Diemen, J. H.; Hage, R.; Haasnoot, J. G.; Lempers, H. E. B.; Reedijk, J.; Vos, J. G.; Cola, L. De; Barigelletti, F.; Balzani, V. Electrochemical and Photophysical Properties of New Triazole-Bridged Heterobimetallic Ruthenium-Rhodium and Ruthenium-Iridium Complexes. *Inorg. Chem.* **1992**, *31*, 3518–3522. DOI: 10.1021/ic00043a008.
